# Reductive Elimination Reactions in Gold(III) Complexes Leading to C(sp^3^)–X (X = C, N, P, O, Halogen) Bond Formation: Inner-Sphere vs S_N_2 Pathways

**DOI:** 10.1021/acs.inorgchem.2c04166

**Published:** 2023-01-20

**Authors:** Alejandro Portugués, Miguel Ángel Martínez-Nortes, Delia Bautista, Pablo González-Herrero, Juan Gil-Rubio

**Affiliations:** † Departamento de Química Inorgánica, Facultad de Química, Universidad de Murcia, Campus de Espinardo, 30100 Murcia, Spain; ‡ ACTI, Universidad de Murcia, Campus de Espinardo, 30100 Murcia, Spain

## Abstract

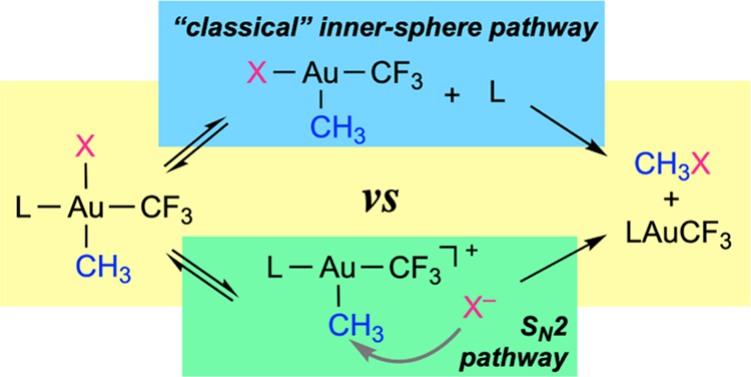

The reactions leading to the formation of C–heteroatom bonds in the coordination sphere of Au(III) complexes are uncommon, and their mechanisms are not well known. This work reports on the synthesis and reductive elimination reactions of a series of Au(III) methyl complexes containing different Au–heteroatom bonds. Complexes [Au(CF_3_)(Me)(X)(PR_3_)] (R = Ph, X = OTf, OClO_3_, ONO_2_, OC(O)CF_3_, F, Cl, Br; R = Cy, X = Me, OTf, Br) were obtained by the reaction of *trans*-[Au(CF_3_)(Me)_2_(PR_3_)] (R = Ph, Cy) with HX. The cationic complex *cis*-[Au(CF_3_)(Me)(PPh_3_)_2_]OTf was obtained by the reaction of [Au(CF_3_)(Me)(OTf)(PPh_3_)] with PPh_3_. Heating these complexes led to the reductive elimination of MeX (X = Me, Ph_3_P^+^, OTf, OClO_3_, ONO_2_, OC(O)CF_3_, F, Cl, Br). Mechanistic studies indicate that these reductive elimination reactions occur either through (a) the formation of tricoordinate intermediates by phosphine dissociation, followed by reductive elimination of MeX, or (b) the attack of weakly coordinating anionic (TfO^–^ or ClO_4_^–^) or neutral nucleophiles (PPh_3_ or NEt_3_) to the Au-bound methyl carbon. The obtained results show for the first time that the nucleophilic substitution should be considered as a likely reductive elimination pathway in Au(III) alkyl complexes.

## Abstract 2

The first experimental and computational evidence of an S_N_2 mechanism for the reductive elimination reaction from Au(III) complexes is reported. Very rare eliminations of highly electrophilic molecules such as MeOTf or MeOClO_3_ are demonstrated.

## Introduction

**Scheme 1:**
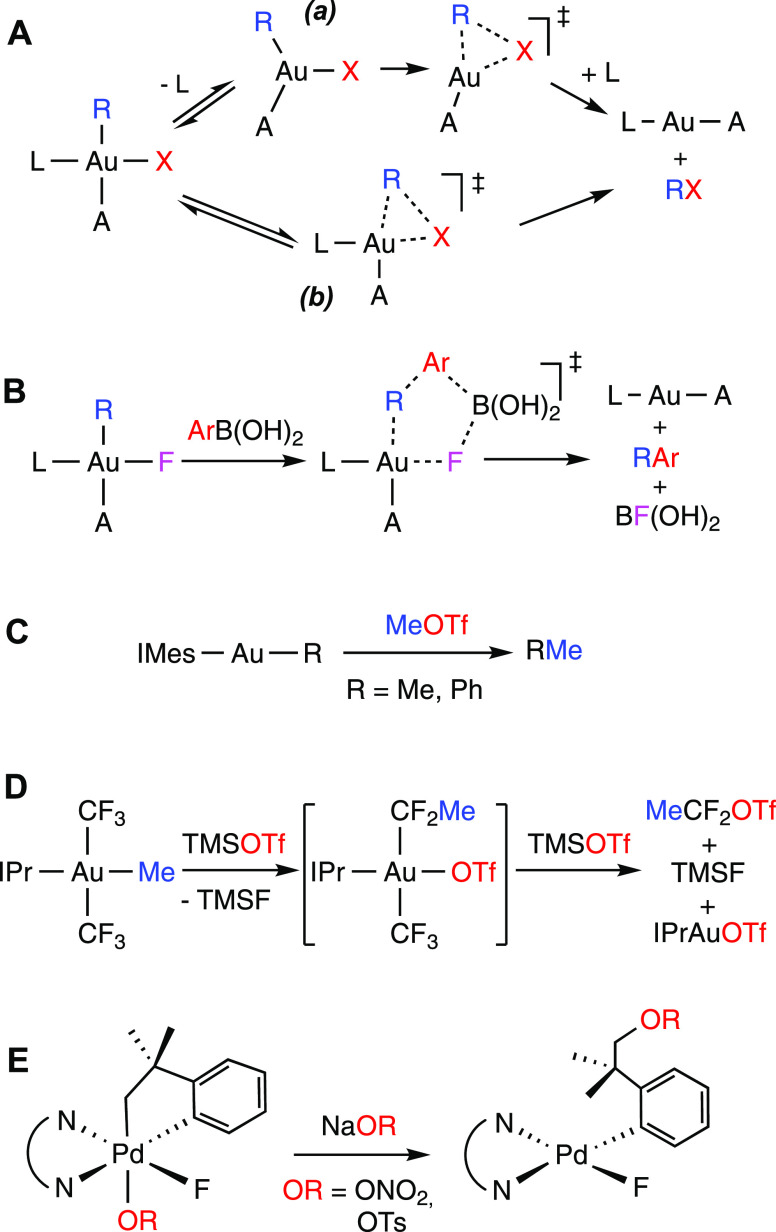
(A) and (B) Reductive Elimination Pathways in Square Planar Au(III) Complexes; (C) Alkylation of Methyl or Aryl Au(I) Complexes with MeOTf; (D and E) Reported Reductive Eliminations of Organic Esters of Strong Oxoacids IMes = 1,3-dimesityl-1*H*-imidazolydene; IPr = 1,3-bis(2,6-diisopropylphenyl)-1*H*-imidazolydene.

The advances in the field of Au(I)/Au(III) catalysis during the last decade^1−15^ have boosted research on fundamental aspects of gold redox reactions.^16−23^ Thus, the oxidative addition of substrates containing C–halogen, C–N_2_^+^, or strained C–C bonds to Au(I) complexes, regarded as the most challenging step of these catalytic cycles, has received considerable attention.^24−30^ By contrast, reductive eliminations in Au(III) complexes have been less studied, despite the fact that the degree of selectivity in this step dramatically affects the outcome of the catalytic processes.^31^ Pioneering studies carried out during the 1970s^32−34^ revealed that reductive elimination in square planar Au(III) complexes can occur (a) through tricoordinate intermediates formed after ligand dissociation or (b) directly from the tetracoordinate complexes (Scheme 1A).^24,25^ Alternatively, a concerted mechanism where C–C and B–F bonds form simultaneously has been proposed for the reaction of Au(III) alkyl fluorido complexes with arylboronic acids (Scheme 1B).^35^ Whereas the experimental evidence of pathways (a)^32−34,36−39^ and (b)^35,40−48^ is abundant for C–C coupling reactions, mechanistic data on reductive elimination processes leading to the C–heteroatom bond formation are scarce. The available information has been obtained on the dissociative reductive eliminations of alkyl,^49,50^ aryl,^38,51^ or trifluoromethyl halides^52^ and nondissociative reductive eliminations of ArX derivatives (X = NR_2_, PR_3_^+^, OR, SR, halogen).^53−59^

Alkyl esters of strong oxyacids such as triflic, perchloric, or nitric acids are powerful electrophiles, capable of alkylating organic^60−62^ or metal-based^63−66^ nucleophiles. In particular, an oxidative addition of MeOTf has been invoked to explain the formation of ethane or toluene in the reaction of [AuR(IMes)] with MeOTf (Scheme 1C).^67^ The reverse reaction, namely, the reductive elimination of organic triflates, perchlorates, or nitrates from metal complexes is thus very unlikely. Nevertheless, it has been postulated for the Bi- or Cu-catalyzed formation of aryl or vinyl triflates^68,69^ and in the reaction of [Au(CF_3_)_2_(Me)(IPr)] with Me_3_SiOTf (Scheme 1D).^70^ Direct reductive elimination of an alkyl nitrate or tosylate has been observed in cyclometalated Pd(IV) complexes (Scheme 1E).^71^

Herein, we report a study on reductive elimination reactions from Au(III) complexes of the type [Au(CF_3_)(Me)X(PR_3_)], which lead to C–C, C–N, C–P, C–O, or C–halogen bond formation. Uncommon reductive elimination products, such as methyl fluoride, triflate, perchlorate, and nitrate, have been observed. Mechanistic and computational studies show that, depending on the ligand X, the classical dissociative pathway or an S_N_2-type pathway operates in these reactions.

## Results and Discussion

### Synthesis of Au(III) Complexes

**Scheme 2:**
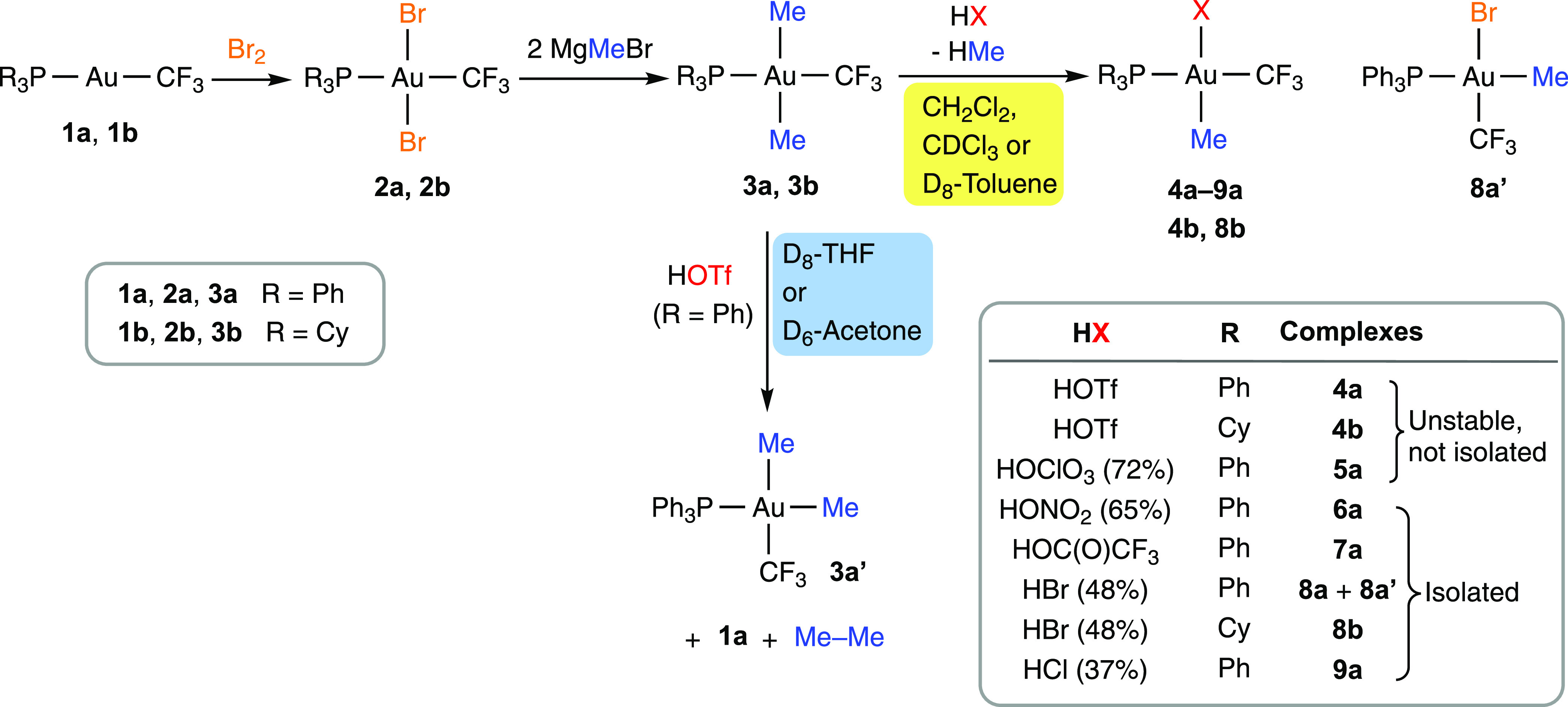
Synthesis and Reactions with Acids of Au(III) Methyl Complexes

Bromination of [Au(CF_3_)(PR_3_)] (R = Ph (**1a**), Cy (**1b**)) followed by the reaction of the resulting dibromo complexes *trans*-[AuBr_2_(CF_3_)(PR_3_)] (R = Ph (**2a**), Cy (**2b**)) with methylmagnesium bromide afforded *trans*-[Au(CF_3_)(Me)_2_(PR_3_)] (R = Ph (**3a**), Cy (**3b**)) in good yields (Scheme 2), which are stable at room temperature under ambient light.

Complex **3a** rapidly reacted in chloroform or dichloromethane with strong protic acids, such as triflic, concentrated aqueous perchloric or nitric, trifluoroacetic, hydrobromic, or hydrochloric acid to give methane and complexes [Au(CF_3_)(Me)(X)(PPh_3_)] (X = OTf (**4a**), OClO_3_ (**5a**), ONO_2_ (**6a**), OC(O)CF_3_ (**7a**), Br (**8a**), Cl (**9a**)) as the main reaction products (Scheme 2). Similarly, the reaction of **3b** with triflic or hydrobromic acid gave [Au(CF_3_)(Me)(X)(PCy_3_)] (X = OTf (**4b**), Br (**8b**)). Remarkably, only one of the methyl ligands was protonated even in the presence of an excess of acid. No significant reaction was observed between **3a** and hydrofluoric acid, acetic acid, phenol, or 4-methoxythiophenol. The protonation of the robust Au–C(sp^3^) bonds^72^ of **3a** or **3b** with acids is in line with the previous results of Kochi and co-workers on the protonolysis of complexes [Au(alkyl)_3_(phosphine)],^36^ where only one of the Au–alkyl bonds *cis* to the phosphine is protonated, in agreement with the mutually exerted large *trans* effect of these alkyl ligands.

Complexes **6a**, **7a**, **8a**, and **8b** were isolated in good yields. The isolated samples of **8a** contained small amounts (4–7%) of the corresponding isomer **8a′**, where the PPh_3_ and Me ligands are mutually *trans* (see below). In contrast, the attempts to isolate **4a**, **4b**, or **5a** gave impure oils, which we attribute to their lower stabilities (see the Supporting Information).

The outcome of the reaction of **3a** with triflic acid depended on the solvent. Thus, the Au–Me bond acidolysis was the dominant process in dichloromethane, chloroform, or toluene, whereas the isomerization to *cis*-[Au(CF_3_)(Me)_2_(PPh_3_)] (**3a′**), with the concurrent reductive elimination of ethane, was the main process in tetrahydrofuran (THF) or acetone (Scheme 2). This reaction occurs even in the presence of a substoichiometric amount of acid (10%) but not when acetic acid was used instead of triflic acid.

These observations can be rationalized by considering the influence of the solvent on the p*K*_a_ of triflic acid.^73^ Thus, the irreversible Au–Me acidolysis needs a strongly acidic medium and therefore it takes place only in those solvents where the p*K*_a_ of the acid is lowest (dichloromethane, chloroform, and toluene). In contrast, in THF or acetone, the p*K*_a_ of triflic acid would not be low enough to protonate the methyl carbon. Then, other reaction pathways would come into play, where the acidic medium facilitates the isomerization and reductive elimination reactions of the Au(III) complexes (see below).

**Scheme 3:**
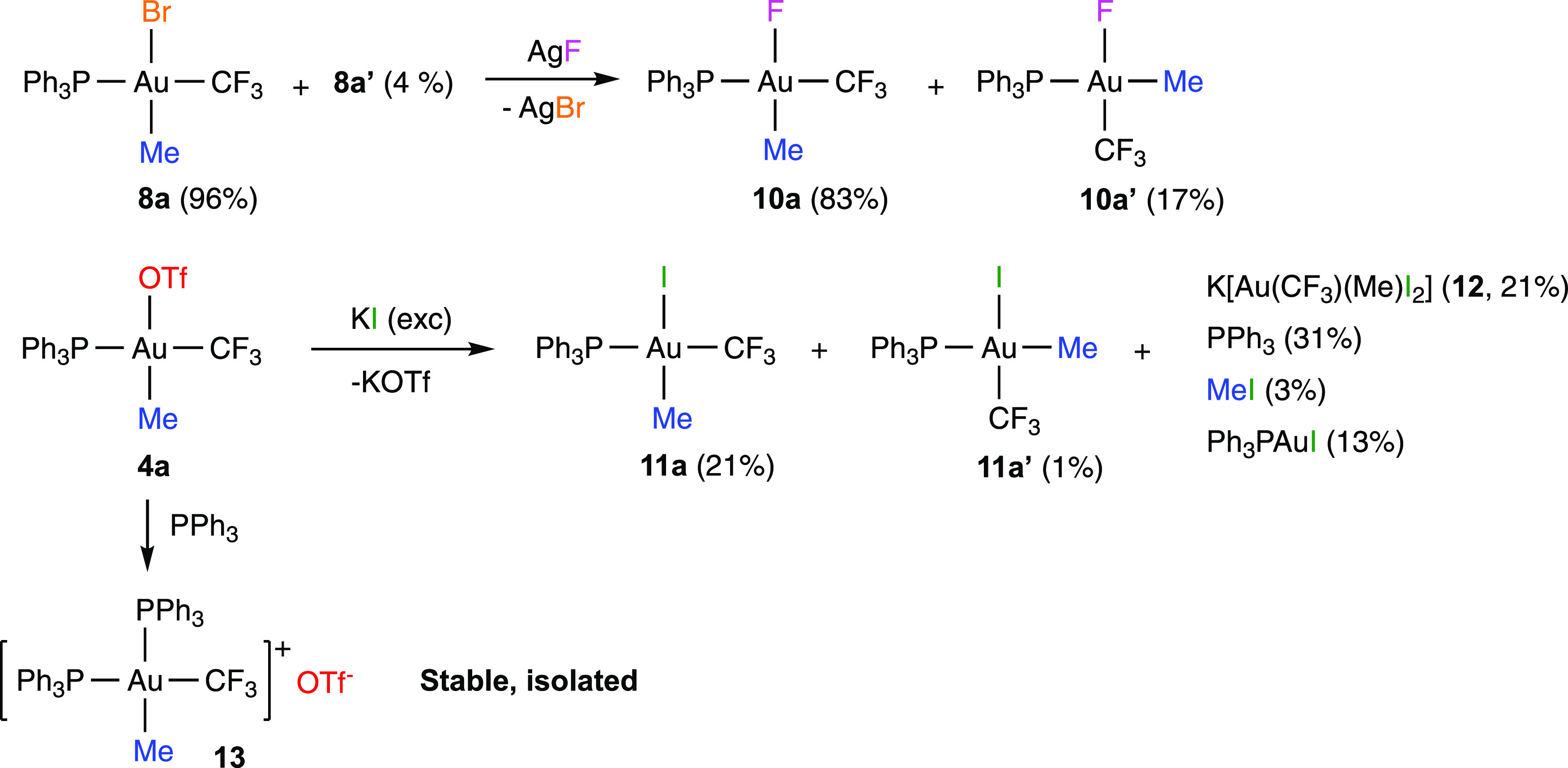
Synthesis of Au(III) Fluorido, Iodido, and Cationic Bis(phosphine) Complexes

The reaction of a mixture of isomers **8a** and **8a′** with AgF gave AgBr and the corresponding fluorido complexes **10a** and **10a′** (Scheme 3), which were unambiguously identified in solution by NMR spectroscopy, but could not be isolated in pure form. The reaction of *in situ*-generated **4a** and KI gave a mixture containing mainly PPh_3_, [Au(CF_3_)(Me)(I)(PPh_3_)] (**11a** and **11a′**), and another gold complex containing the CF_3_ and CH_3_ ligands but lacking the PPh_3_ ligand, which was tentatively identified as *cis*- or *trans*-K[Au(CF_3_)(Me)I_2_] (**12**) (Scheme 3). MeI and [AuI(PPh_3_)] were also detected by NMR spectroscopy in the reaction mixture. The reaction of **4a** with PPh_3_ cleanly gave *cis*-[Au(CF_3_)(Me)(PPh_3_)_2_] (**13**), which was isolated in good yield (Scheme 3).

**Figure 1:**
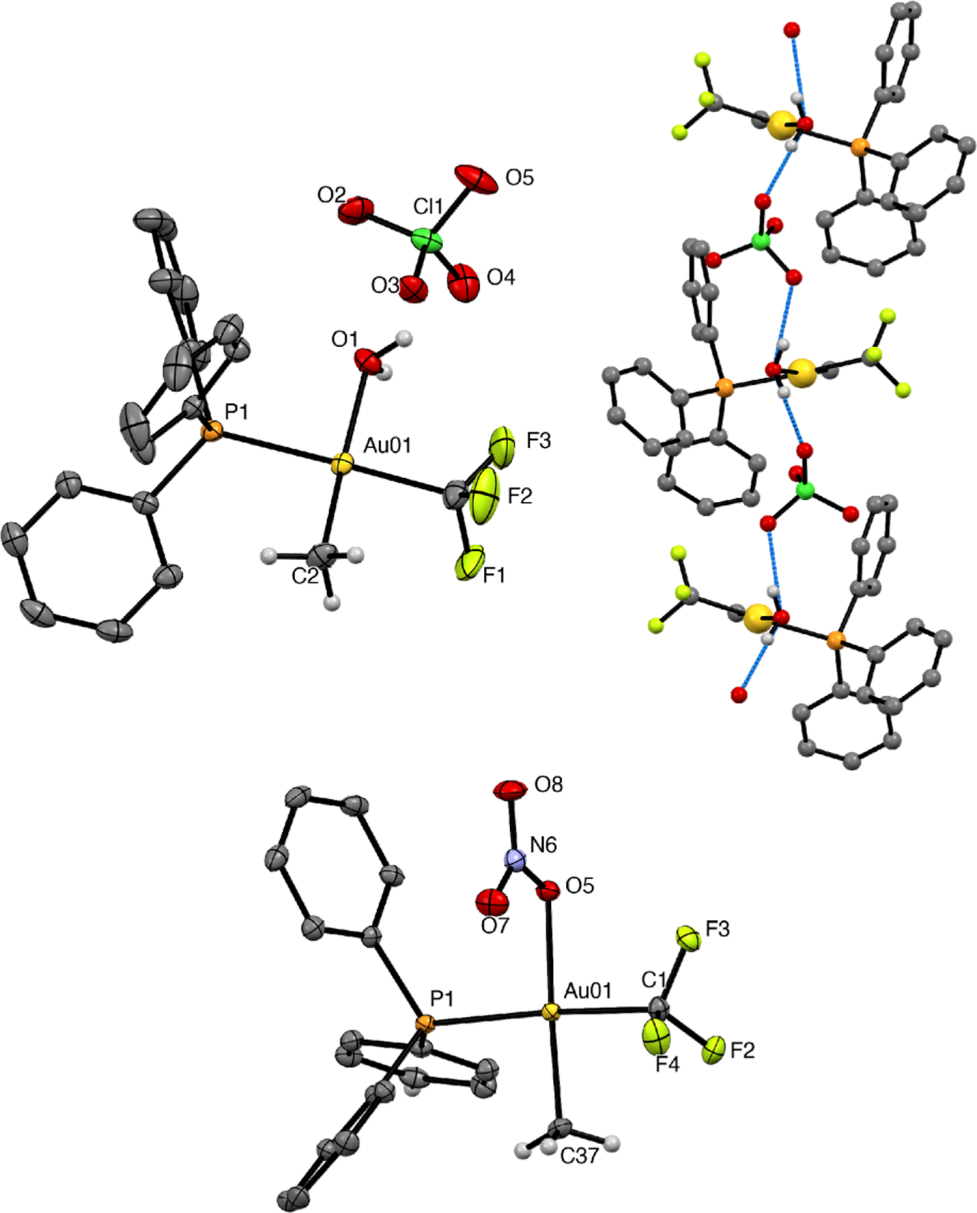
ORTEP representations (50% ellipsoids, phenylic H’s omitted) of the structures of the cation and the anion of the salt [Au(OH_2_)(CF_3_)(Me)(PPh_3_)]ClO_4_ (**5a**·H_2_O) (up, left) and complex **6a** (down). Representation of the chains of ···H–O–H···O–Cl–O··· hydrogen bonds between the ClO_4_^–^ anions and H_2_O ligands of the salt (up, right). Tables of bond lengths and angles are included in the Supporting Information.

Single crystals were obtained from an *in situ*-generated solution of **5a**. However, the X-ray diffraction analysis showed that the crystal contained the salt [Au(CF_3_)(Me)(OH_2_)(PPh_3_)]ClO_4_ (**5a**·H_2_O), instead of the expected perchlorato complex (Figure 1), suggesting that under the reaction conditions, **5a** and H_2_O are in equilibrium with **5a**·H_2_O. In the crystal structure, each ClO_4_^–^ anion is hydrogen-bonded to two coordinated water molecules to form ···O–Cl–O···H–O–H··· chains along the *a* axis. The Au–OH_2_ distance (2.1562(17) Å) is almost identical to the value found in *cis*-[Au(Me)_2_(OTf)(OH_2_)] (2.157(6) Å).^74^ The crystal structure of **6a** (Figure 1) shows that the nitrato ligand is coordinated to gold through one of the oxygen atoms. The Au–ONO_2_ distance (2.133(2) Å) is similar to that found in [Au(CH_2_COMe)(ONO_2_)(ppy)] (2.128(3) Å; ppy = 2-phenylen-2′-yl-pyridine)^75^ but longer than in [Au(CF_3_)_3_(ONO_2_)] (2.090(4) Å).^76^ In both **5a**·H_2_O and **6a**, the trifluoromethyl and phosphine ligands are in a mutual *trans* disposition.

The ^19^F and ^31^P{^1^H} NMR spectra of **3a**–**9a**, **11a**, **3b**, **4b**, and **8b** showed a doublet and a quartet, respectively, with a large ^3^*J*_PF_ value (62.4–72.8 Hz) characteristic of a mutually *trans* arrangement of the phosphine and trifluoromethyl ligands. The ^31^P{^1^H} NMR spectrum of **13** showed the presence of two inequivalent and mutually coupled ^31^P nuclei with an additional splitting due to ^31^P–^19^F coupling, indicating a *cis* configuration. The ^19^F_3_C and ^31^PPh_3_ resonances of **10a** and **10a′** show coupling with the gold-bound fluorine nucleus, and the ^19^F NMR spectrum of the mixture displays two signals at −223.7 (**10a**) and −246.6 ppm (**10a′**), which fall in the same region as those of previously reported Au(III) fluorido complexes.^49,77,78^

### Reductive Elimination and Isomerization Reactions

**Scheme 4:**
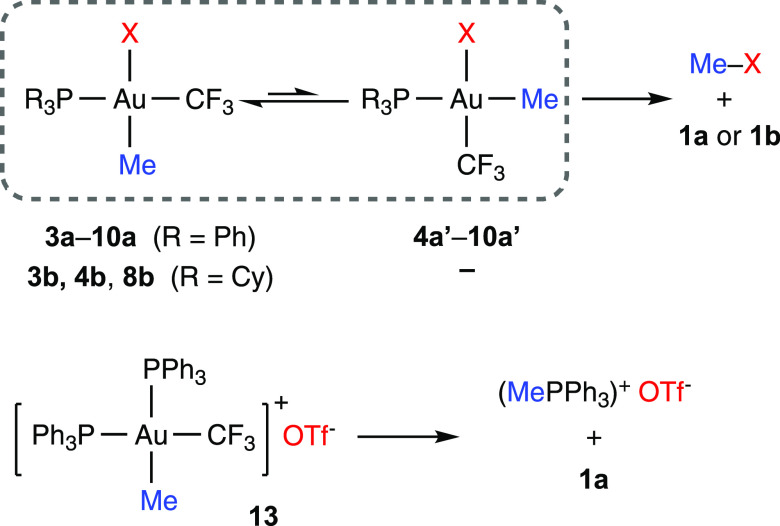
Thermal Decomposition and Isomerization of the Au(III) Complexes

Heating of solutions of the Au(III) complexes led to the reductive elimination of MeX (X = Me, OTf, OClO_3_, ONO_2_, OC(O)CF_3_, F, Cl, Br) as the major process, with the formation of [Au(CF_3_)(PR_3_)] (R = Ph (**1a**) or Cy (**1b**)) (Scheme 4 and Table 1). Thermal isomerization was observed as a secondary process in most cases. Upon heating, the concentration of isomers **5a′**–**10a′** increased rapidly and then remained steady at the equilibrium proportions shown in Table 1, until both isomers completely transformed into the corresponding reductive elimination products. Exceptions to this behavior were the triflato complex **4a**, which gave very small amounts of its isomer **4a′**, and the PCy_3_ complexes **3b**, **4b**, and **8b**, for which no isomerization was observed. The fastest decompositions were observed for the triflato and perchlorato complexes (**4a**, **4b**, and **5a**), which were consumed in 0.5–4.5 h at 50 °C. In contrast, the decomposition of **6a**, **7a**, **8a**, **9a**, or **10a** required higher temperatures and longer times. Finally, the decomposition of the PCy_3_ complexes **3b** and **8b** required more energetic conditions compared to their PPh_3_ counterparts **3a** and **8a**.

**Table 1:** Decomposition of the Au(III) Complexes: Reaction Temperatures and Times, Isomerization Degree, and Observed Secondary Products^a^

complexes	X	*T* (°C)	*t* (h)^b^	isom. (%)^c^	MeX yield (%)^f^	secondary products
**3a**	Me	100	27		>95^g^	
**3b**	Me	140	>14^e^			
**4a**, **4a′**^d^	OTf	50	3.5	<4	70	[Au(PPh_3_)_2_]X, Au
**4b**^d^	OTf	50	4.5		70	[Au(PCy_3_)_2_]X, [AuX(PCy_3_)], Au
**5a**, **5a′**^d^	OClO_3_	50	0.5	5	50	[Au(PPh_3_)_2_]X, Au
**6a**, **6a′**	ONO_2_	80	6	12	78	[Au(PPh_3_)_2_]X, Au, unknown
**7a**, **7a′**	OCOCF_3_	100	17.5	20	83^g^	(PMePh_3_)X (5%)
**10a**, **10a′**	F	110	14	17	h	[Au(PPh_3_)_2_]X, ethane
**9a**, **9a′**	Cl	80	5	16	h	Ph_3_PAuCl, (PMePh_3_)Cl (5%)
**8a**, **8a′**	Br	80	4.3	18	79^g^	Ph_3_PAuBr, (PMePh_3_)Br (5%)
**8b**	Br	110	20		h	

a All experiments were carried out in CDCl_3_ except the decompositions of **3a**, **3b**, and **7a**, which were carried out in *D*_8_-toluene (all spectra are given in the Supporting Information).

b Necessary time for the consumption of at least 95% of the starting Au(III) complex.

c Equilibrium proportion of isomers **4a′**–**10a′**.

d *In situ*-generated from **3a** or **3b** and HX.

e The conversion was 6.6% after 14 h.

f Determined by integration of the ^1^H NMR spectra measured before and after the reaction.

g Indirectly estimated from the resulting concentration of **1a**.

h The yield was not accurately determined in these cases.

Au(I) complexes [Au(PR_3_)_2_]^+^ (R = Ph or Cy) were formed as secondary products in most cases. These products arise from hydrolysis or decomposition of the resulting Au(I) complexes **1a** and **1b** (see the Supporting Information). Small amounts (5%) of phosphonium salts (PMePh_3_)X (X = OC(O)CF_3_, Cl, Br) were observed in the decompositions of **7a**, **8a**, and **9a**. Similarly, complex **13** underwent a quantitative reductive elimination of (PMePh_3_)OTf after heating for 2 h at 80 °C or 4 h at 60 °C (Scheme 4).

The configurational assignment of isomers **3a′**–**11a′** is supported by their smaller ^3^*J*_PF*cis*_ (9.1–11.8 Hz) and larger ^3^*J*_PH*trans*_ (8.9–9.7 Hz) values with respect to those of **3a**–**11a** (^3^*J*_PF*trans*_ 62.4–72.8 Hz; ^3^*J*_PH*cis*_ 5.8–6.6 Hz), as well as by the larger ^31^P–^13^CH_3_ coupling constant of **7a′** (^2^*J*_PC*trans*_ = 91.6 Hz) compared to that of **7a** (^2^*J*_PC*cis*_ = 3.8 Hz).^79^

### Mechanistic Studies

The concentrations of the reactants and products during the thermal decomposition of representative complexes (**3a**, **8a**, and **13**) were monitored by NMR spectroscopy. In addition, the effect of the presence of an additional amount of PPh_3_ was studied.

**Figure 2:**
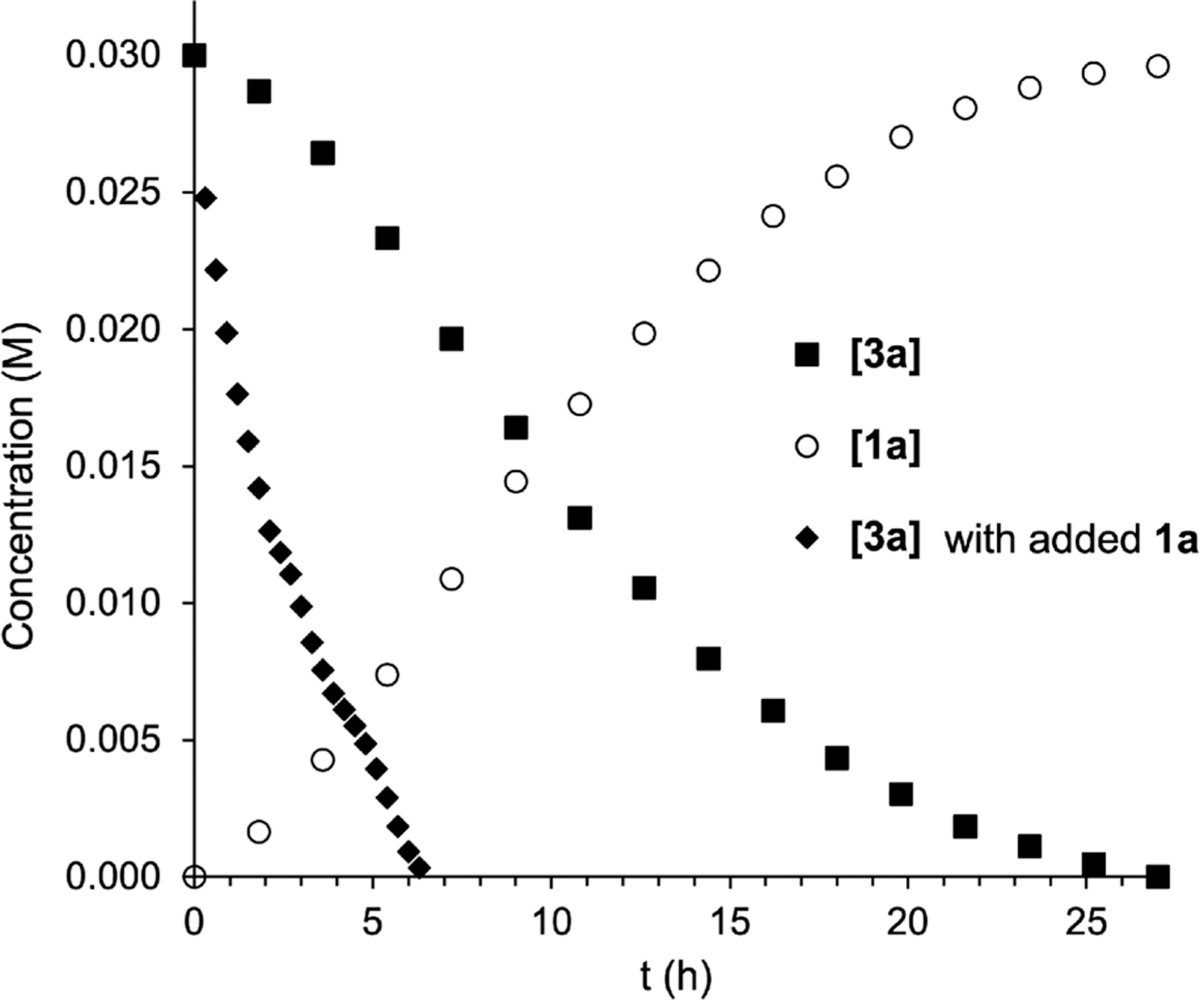
Thermal decomposition of **3a** (100 °C, *D*_8_-toluene): [**3a**] and [**1a**] vs time; [**3a**] vs time in the presence of added **1a** (4.6 equiv). Concentrations were determined by integration of the ^19^F NMR spectra using PhCF_3_ as the internal standard.

**Scheme 5:**
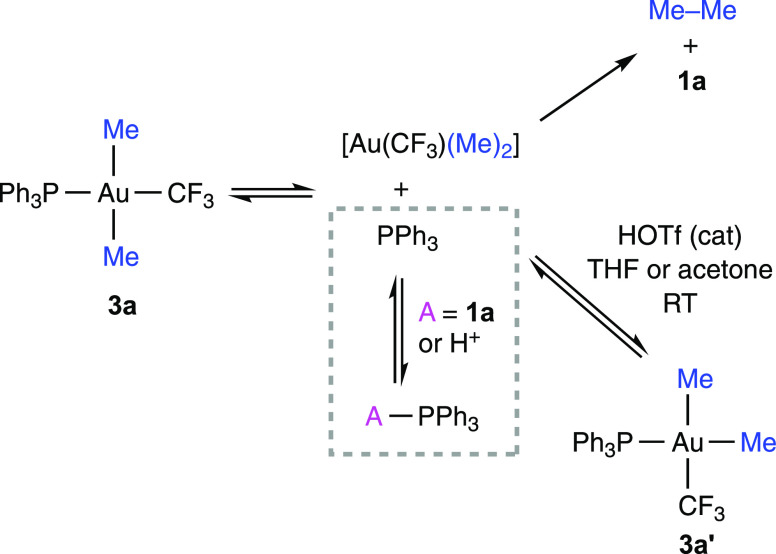
Proposed Dissociative Mechanism for the Decomposition and Isomerization of **3a** Effect of **1a** or HOTf on the PPh_3_ dissociation equilibrium.

The decomposition of the dimethyl complex **3a** was monitored at 100 °C (Figure 2). The formation of ethane from **3a** was drastically inhibited by the addition of PPh_3_ (0.2 or 1 equiv), which is in agreement with a mechanism where PPh_3_ dissociation gives the tricoordinate intermediate [Au(CF_3_)(Me)_2_], which decomposes to give ethane (Scheme 5). Remarkably, the rate of consumption of **3a** increased with time until approximately half-conversion, suggesting the acceleration of the reaction by one of the reaction products. Indeed, the reaction was faster in the presence of added **1a** (Figure 2). The exchange between coordinated and free PPh_3_ in a sample containing **1a** and PPh_3_ was observed, in agreement with **1a** and PPh_3_ being in equilibrium with [Au(CF_3_)(PPh_3_)_2_] (see Figures S86 and S103). This would decrease the concentration of free PPh_3_, increasing the amount of the tricoordinate intermediate (Scheme 5).

The proposed dissociative mechanism also explains the observed catalytic effect of triflic acid on the decomposition and isomerization of **3a** in acetone or THF. Thus, the protonation of free PPh_3_ would shift the phosphine dissociation equilibrium toward the tricoordinate intermediate at room temperature (Scheme 5). This intermediate could undergo the elimination of ethane or coordinate a molecule of PPh_3_ to give **3a′**. In the absence of an acid, achieving a significant PPh_3_ dissociation degree would require a higher temperature, which would produce the decomposition of the tricoordinate intermediate to give ethane and **1a**.

**Figure 3:**
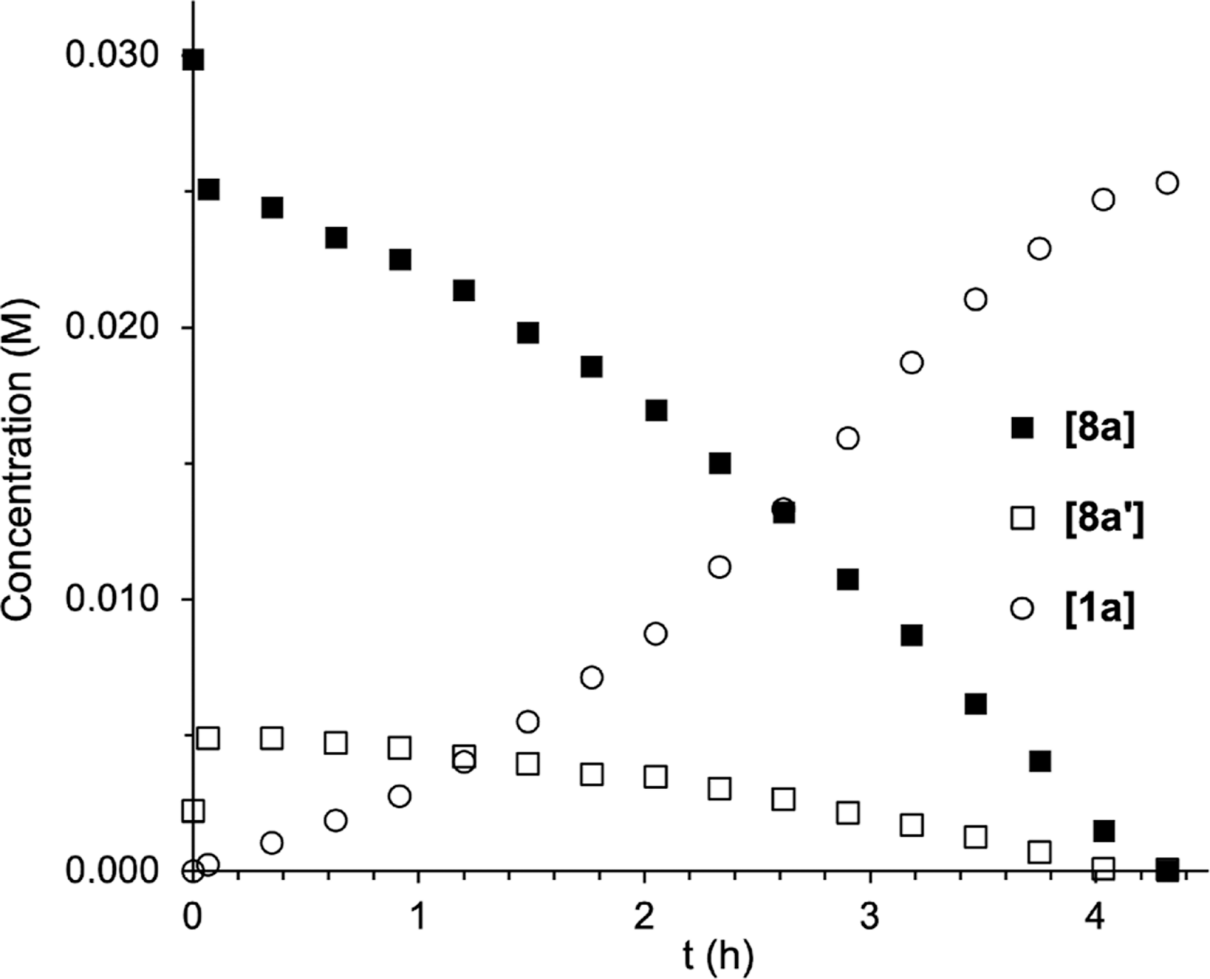
[**8a**], [**8a′**], and [**1a**] vs time (80 °C, CDCl_3_). Concentrations were determined by integration of the ^19^F NMR spectra using PhCF_3_ as the internal standard.

**Scheme 6:**
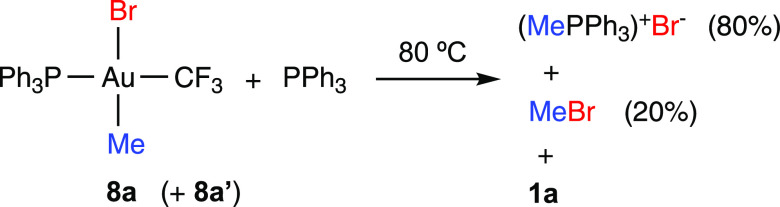
Decomposition of **8a** and **8a′** in the Presence of Added PPh_3_

Heating of a solution of the bromido complexes **8a** (96%) and **8a′** (4%) at 80 °C led to the rapid equilibration of both isomers at an 82:18 ratio, respectively, which remained constant during the reaction (Figure 3). As observed for **3a**, the rate of formation of **1a** increased with time until half-conversion, suggesting the acceleration of the reaction by a reaction product. In the presence of one additional equivalent of PPh_3_, both the consumption of **8a** and **8a′** and the formation of MeBr were slower, in agreement with a PPh_3_-dissociative pathway (Figure S83). However, in these conditions, the major reaction product was (PMePh_3_)Br (Scheme 6). Similarly, the heating of [Au(CF_3_)(Me)(Cl)(PPh_3_)] (**9a**) in the presence of PPh_3_ (5 equiv) gave mainly (PMePh_3_)Cl.

The slower reductive eliminations of the tricyclohexylphosphine complexes **3b** and **8b** are also in line with phosphine dissociation being the rate-determining step and are attributed to the higher donor ability of PCy_3_ compared with that of PPh_3_.

**Figure 4:**
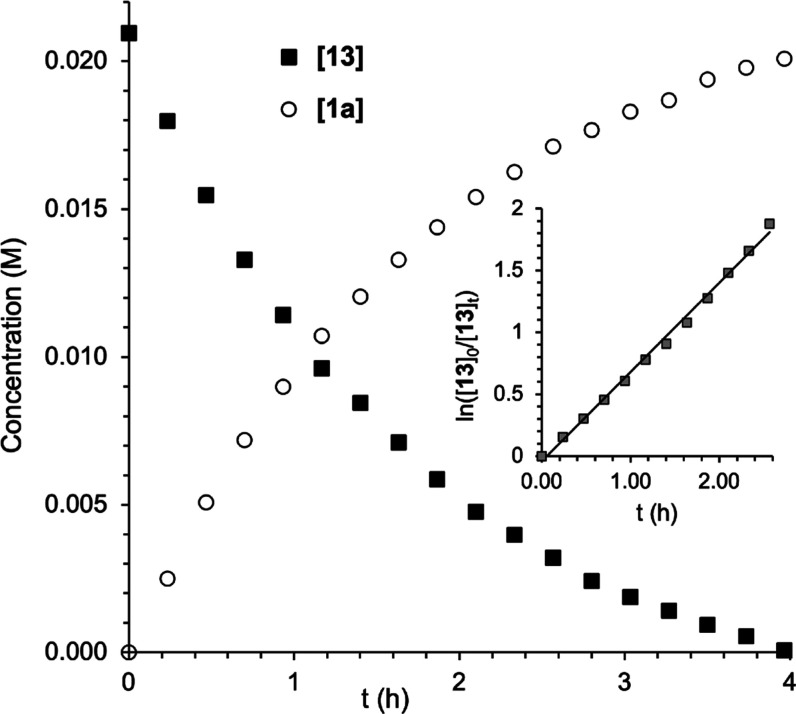
Thermal decomposition of **13** (60 °C in CDCl_3_): [**13**] and [**1a**] vs time; (inset) plot of ln([**13**]_0_/[**13**]*_t_*) vs time illustrating the first-order decay of [**13**] up to a conversion of 85% (*t* = 2.57 h). Concentrations were determined by integration of the ^19^F NMR spectra using PhCF_3_ as the internal standard.

The decomposition of **13** into **1a** and (PMePh_3_)OTf was monitored at 60 °C in CDCl_3_ (Figure 4). In marked contrast with the behavior of **3a** and **8a**, the depletion of **13** showed a first-order dependence until an 85% conversion and, importantly, was accelerated by the added PPh_3_ (the completion time decreased from 4 to 0.25 h in the presence of 5 equiv of PPh_3_). Since this PPh_3_-induced reaction acceleration is not compatible with a phosphine-dissociative mechanism, the concerted reductive elimination from a pentacoordinate intermediate or the nucleophilic attack of PPh_3_ on the methylic carbon was considered as alternative pathways.

**Scheme 7:**
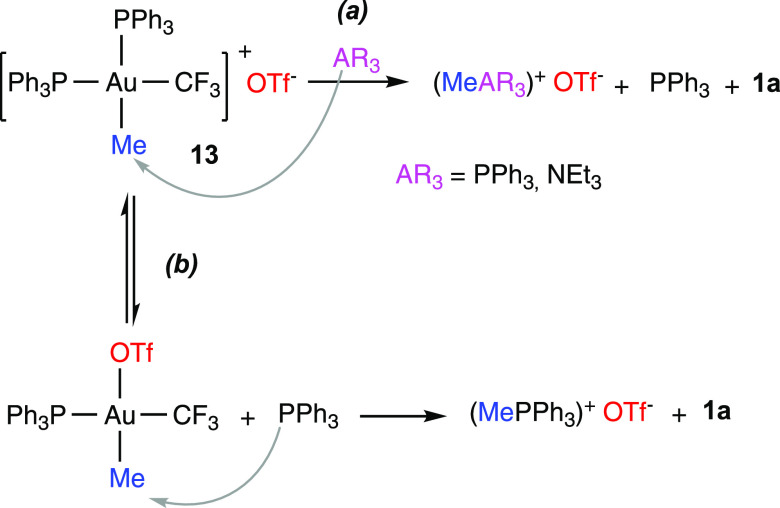
Nucleophilic Attack on the Methyl Carbon of (a) Added PPh_3_ or NEt_3_ and (b) Dissociated PPh_3_

The room-temperature ^31^P NMR spectrum of a mixture of **13** and PPh_3_ did not show evidence of pentacoordinated species. Instead, only three sharp signals corresponding to **13** and free PPh_3_ were observed (Figure S103). To test the possibility of a nucleophilic attack, **13** was reacted with 2 equivalents of NEt_3_ at 50 °C. In these conditions, **1a** and a mixture of (PMePh_3_)OTf and (NEt_3_Me)OTf in a 0.78:1 molar ratio formed (Scheme 7a). Besides, no signs of NEt_3_ coordination or substitution of PPh_3_ by NEt_3_ were found in the NMR spectra. Overall, these observations point to the external attack of PPh_3_ or NEt_3_ on the methyl carbon being the main reaction pathway.

In the absence of added phosphine, the formation of (PMePh_3_)OTf from **13** could possibly occur through the dissociation of PPh_3_ followed by nucleophilic substitution on the methyl carbon (Scheme 7b). In agreement with this, the reductive elimination of the phosphonium salt was faster in solvents with a higher coordinating ability (reaction times at 60 °C: 1.5 h in acetone, 0.75 h in acetonitrile). The fast dissociation of PPh_3_ was evidenced by the ^31^P{^1^H} NMR spectrum of **13** at 60 °C as a broadening of the signal of the ^31^P *trans* to the methyl ligand, with the loss of P–P and P–F couplings (Figure S100). In addition, the observed first-order dependence of the consumption rate of **13** agrees with this mechanism if a steady concentration of the intermediate is assumed (Figure S96).

The formation of (PMePh_3_)X (X = Cl, Br) from **8a** or **9a** in the presence of PPh_3_ could follow a similar pathway, although in this case a fraction of the observed phosphonium salts could be originated by the reaction of the formed MeBr or MeCl with PPh_3_. The formation of small amounts of phosphonium salts during the thermal decompositions of **7a**, **8a**, and **9a** (Table 1) suggests that the S_N_2 pathway could be a secondary reductive elimination route in these cases.

The conversion of complexes **6a**, **7a**, **9a**, or **10a** into **1a** and MeONO_2_, MeOC(O)CF_3_, MeCl, or MeF, respectively, needed reaction times (5–20 h) and temperatures (80–100 °C) comparable to those of **8a**. The equilibrium amount of their respective isomers **6a′**, **7a′**, **9a′**, or **10a′** (12–20%) was similar to that of **8a′** (18%). Considering these similarities, we propose that they mainly proceed through a phosphine-dissociative mechanism similar to that of **8a**.

In marked contrast, the reductive eliminations of MeOTf or MeOClO_3_ from **4a**, **4b**, or **5a** were significantly faster, suggesting a different reaction pathway. In addition, complexes **4a** and **4b** show comparable decomposition times, meaning that phosphine dissociation is not rate-determining in these cases. Considering the low coordinating abilities of OTf^–^ and ClO_4_^–^, the degree of anion dissociation is expected to be higher for **4a**, **4b**, or **5a** compared with the rest of the studied complexes and thus could reasonably be the starting point of the observed reactivity.

### Computational Study

**Figure 5:**
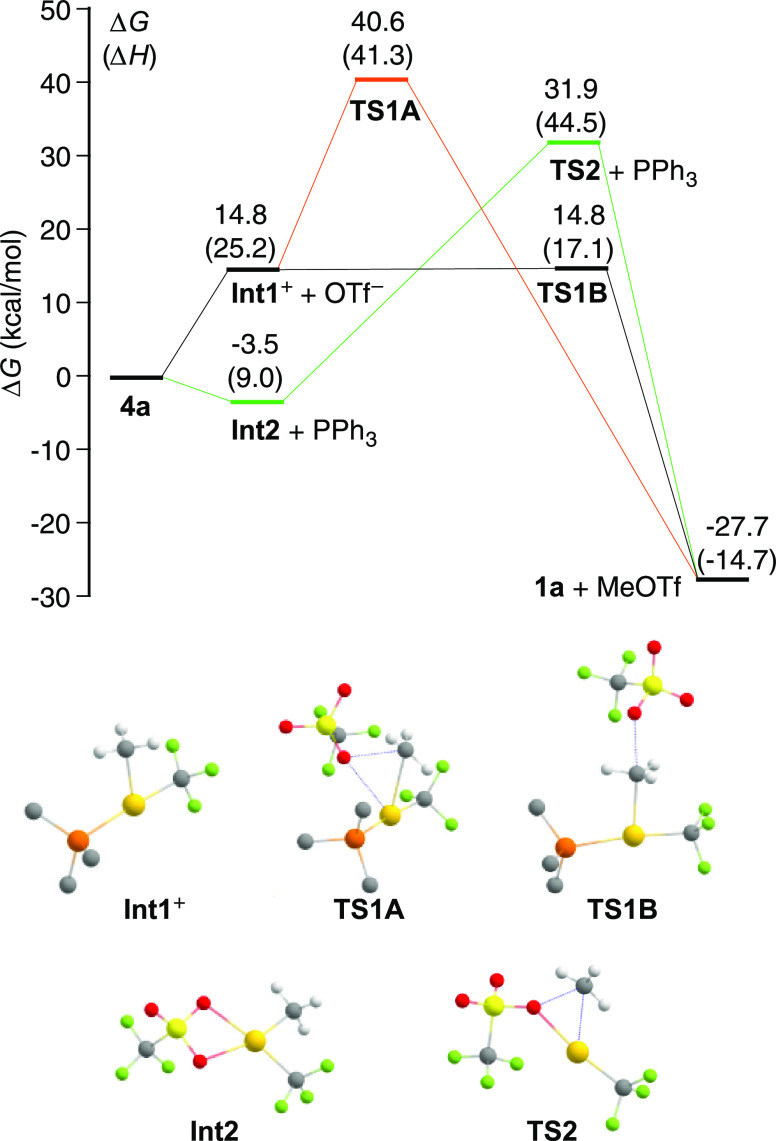
Free-energy profiles (in kcal/mol) of the modeled reaction pathways for the reductive elimination of MeOTf from complex **4a** and structures of intermediate species and transition states. Enthalpies are given in parentheses.

For an understanding of the decomposition reactions of the Au(III) complexes bearing a labile anionic ligand, the reductive elimination of MeOTf from **4a** was modeled using DFT calculations at the B3LYP/(6-31G**+LANL2DZ) level in chloroform solution (see details in the Supporting Information). Different pathways involving the dissociation of PPh_3_ or OTf^–^ as the first step were calculated and compared (Figure 5). In both cases, the relaxed scan of the potential energy as the Au–P or Au–O distance was elongated to 4.5 or 3.2 Å, respectively, produced a Morse-type curve with a gradual increase in energy as the ligands are separated from the metal (Figure S110). Therefore, no transition state can be located using this theoretical model. These scans appear to indicate a higher activation barrier for the dissociation of PPh_3_ compared to that of OTf^–^. However, the calculated free-energy change for the dissociation of OTf^–^ to give the cationic tricoordinate intermediate [Au(CF_3_)(Me)(PPh_3_)]^+^ (**Int1**^**+**^) is significantly higher relative to the dissociation of PPh_3_ to give [Au(CF_3_)(Me)(OTf)] (**Int2**), the latter being slightly exergonic. This can be explained by a higher entropy increase upon the dissociation of PPh_3_ and, more importantly, an additional stabilization of the **Int2** fragment due to the chelating coordination of the OTf^–^ ligand.

The C–O coupling step could occur from **Int1**^**+**^ through two different mechanisms. The first one involves a three-centered transition state with a pseudotetrahedral coordination around the gold atom (**TS1A**), having a very high free energy. The second one involves an S_N_2-like transition state resulting from the nucleophilic attack of the OTf^–^ anion on the methyl ligand (**TS1B**) and provides a much more favorable pathway because it has virtually the same free energy as the previous dissociation step. On the other hand, the C–O coupling from **Int2** would occur through a highly energetic tricoordinate transition state (**TS2**), which makes the PPh_3_ dissociation pathway clearly unfavorable. The attempts to model the concerted reaction pathways through pseudotetrahedral or planar transition states formed from **4a** or **4a′**, respectively, converged to a transition state very similar to **TS1A**.

**Scheme 8:**
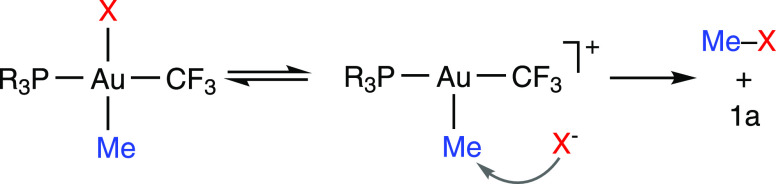
Decomposition of Triflate or Perchlorate Complexes by Anion Dissociation Followed by Nucleophilic Attack

Thus, on the basis of these calculations and the above-presented experimental data, the C–O reductive couplings from **4a**, **4b**, or **5a** occur most likely via dissociation of the labile anionic ligand and subsequent nucleophilic attack on the metal-bound methyl group (Scheme 8). Previous studies have revealed that this is a feasible pathway for the reductive elimination of Csp^3^–X (X = N, O, Cl, Br, I) coupling products in Rh(III),^80,81^ Pd(II),^82^ Pd(IV),^71,83,84^ and Pt(IV)^85−88^ alkyl complexes. Interestingly, the studies on Pt(IV) complexes showed that reductive elimination is faster for more electron-deficient anions,^86^ despite their lower nucleophilicity.^89^ The S_N_2 reductive elimination mechanism has been previously considered for Au(III) complexes, but to the best of our knowledge, no experimental evidence has been provided. Thus, in a computational study of the Au-catalyzed methane oxidation, Periana and co-workers proposed that the formation of the O–CH_3_ bond could take place through the nucleophilic attack of a free HSO_4_^–^ anion on an intermediate Au(III) methyl complex.^90^ Later, Bercaw and co-workers discarded an S_N_2-type pathway for the reductive elimination of MeI from [Au(Me)(I)_2_(IPr)].^50^

**Figure 6:**
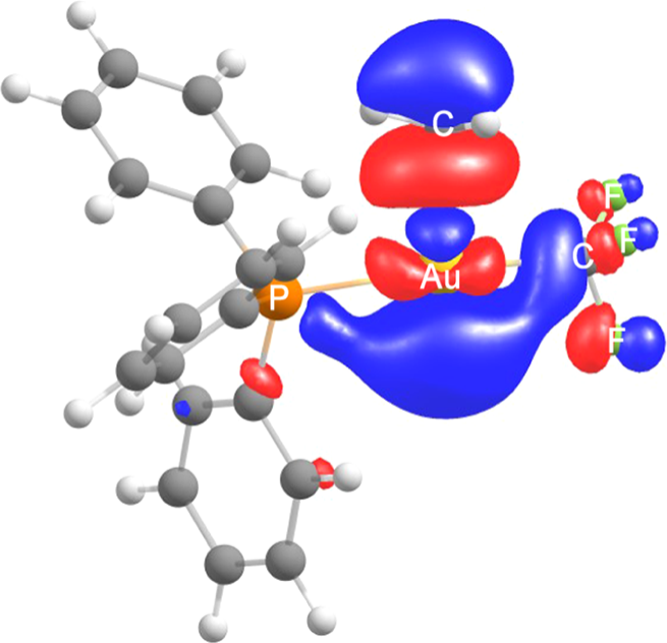
LUMO isosurface (0.035 e/bohr^3^) for the cationic species **Int1**^**+**^.

The lowest unoccupied molecular orbital (LUMO) isosurface of **Int1**^+^ (Figure 6) provides additional support for the proposed mechanism. It is a σ* orbital mostly distributed along the Au–C bond, with large external lobes, implying that both the C and Au atoms can display Lewis-acidic behavior. The electrophilicity of the Me group in this species is also supported by the above-discussed nucleophilic attacks of PPh_3_ or NEt_3_ on this group.

## Conclusions

The thermal decomposition of complexes [Au(CF_3_)(Me)(X)(PR_3_)] leads to [Au(CF_3_)(PR_3_)] and C(sp^3^)–X (X = C, P, O, F, Cl, Br) coupling products. The observed reactions include rare examples of reductive eliminations of methyl fluoride or highly electrophilic molecules such as methyl triflate or perchlorate. Reductive elimination is accompanied by the partial isomerization of the corresponding Au(III) complexes.

Mechanistic studies indicate that the reductive eliminations of ethane or methyl bromide from [Au(CF_3_)(Me)(X)(PR_3_)] (X = Me, Br) take place from a tricoordinate intermediate formed by phosphine dissociation. In contrast, in the cases of the analogous complexes with X = OTf or ClO_4_, and the cationic complex *cis*-[Au(CF_3_)(Me)(PPh_3_)_2_]OTf, experimental evidence and computational modeling are consistent with a reaction pathway involving X^–^ or PPh_3_ dissociation (respectively) followed by nucleophilic attack to the gold-bound carbon. This S_N_2 reductive elimination mechanism has not been documented before in Au(III) complexes and should be considered as a potential pathway in Au(I)/Au(III)-catalyzed reactions, in particular, in those leading to C(sp^3^)–heteroatom coupling products.

## Supporting Information Available

Synthetic procedures, characterization data, NMR spectra, decomposition and mechanistic experiments, crystallographic data, and details of the computational study (PDF)
