# Supplementary material for: Reductive Elimination Reactions in Gold(III) Complexes Leading to C(sp3)–X (X = C, N, P, O, Halogen) Bond Formation: Inner-Sphere vs SN2 Pathways
Source: Inorg Chem. 2023 Jan 20;62(4):1708–18. doi: 10.1021/acs.inorgchem.2c04166 (PMC9890567; doi:10.1021/acs.inorgchem.2c04166)
Supplement: Supplementary file 1 — ic2c04166_si_001.pdf [file ic2c04166_si_001.pdf]

# SUPPORTING INFORMATION

# Reductive Elimination Reactions in Gold(III) Complexes Leading to Csp$^3$–X (X = C, N, P, O, Halogen) Bond Formation: Inner-Sphere vs S$_N$2 Pathways

*Alejandro Portugués,[a] Miguel Ángel Martínez-Nortes,[a] Delia Bautista,[b] Pablo González-Herrero,[a] Juan Gil-Rubio*[a]*

[a] Departamento de Química Inorgánica. Facultad de Química. Universidad de Murcia. Campus de Espinardo, 30100 Murcia (Spain). E-mail: jgr@um.es.

[b] ACTI, Universidad de Murcia. Campus de Espinardo, 30100 Murcia (Spain).

**Table of contents**


1. General considerations, materials and methods ........................................... S2
2. Synthesis, characterization and spectra ........................................... S2
    - [Au($CF_3$)($PPh_3$)] (**1a**) ........................................... S2
    - [Au($CF_3$)($PCy_3$)] (**1b**) ........................................... S2
    - *trans*-[Au($CF_3$)$Br_2$($PPh_3$)] (**2a**) ........................................... S2
    - *trans*-[Au($CF_3$)$Br_2$($PCy_3$)] (**2b**) ........................................... S3
    - *trans*-[Au($CF_3$)$Me_2$($PPh_3$)] (**3a**) ........................................... S5
    - *trans*-[Au($CF_3$)$Me_2$($PCy_3$)] (**3b**) ........................................... S7
    - *SP-4-3*- and *SP-4-4*-[Au($CF_3$)(Me)(OTf)($PPh_3$)] (**4a** and **4a'**) ................ S9
    - *SP-4-3*-[Au($CF_3$)(Me)(OTf)($PCy_3$)] (**4b**) ........................................... S11
    - *SP-4-3*- and *SP-4-4*-[Au($CF_3$)(Me)($OClO_3$)($PPh_3$)] (**5a** and **5a'**) ............ S12
    - *SP-4-3*- and *SP-4-4*-[Au($CF_3$)(Me)($ONO_2$)($PPh_3$)] (**6a** and **6a'**) ............ S14
    - *SP-4-3*- and *SP-4-4*-[Au($CF_3$)(Me)($OCOCF_3$)($PPh_3$)] (**7a** and **7a'**) ........ S16
    - *SP-4-4*- and *SP-4-3*-[Au($CF_3$)(Me)Br($PPh_3$)] (**8a** and **8a'**) ..................... S21
    - *SP-4-4*-[Au($CF_3$)(Me)Br($PCy_3$)] (**8b**) ........................................... S23
    - *SP-4-4*- and *SP-4-3*-[Au($CF_3$)(Me)Cl($PPh_3$)] (**9a** and **9a'**) ..................... S25
    - *SP-4-3* and *SP-4-4*-[Au($CF_3$)(Me)F($PPh_3$)] (**10a** and **10a'**) ................... S28
    - Reaction of **3a** with HOTf and KI ........................................... S30
    - *cis*-[Au($CF_3$)(Me)$(PPh_3)_2$]TfO (**13**) ........................................... S31
    - Formation of complexes of the types [Au$(PR_3)_2$]X and [AuX($PR_3$)] in some acidolysis and reductive elimination reactions ...................................... S34
    - Table S1. Selected NMR data of the Au(III) complexes ........................ S35
3. Reductive elimination reactions ........................................... S36
    - 3.1. Reductive elimination of ethane from **3a** and **3b** ............................... S37
    - 3.2. Reaction of **3a** with HOTf in THF. Isomerization to *cis*-[Au($CF_3$)$(Me)_2$($PPh_3$)] (**3a'**) and reductive elimination of ethane ......... S39
    - 3.3. Reductive elimination of MeOTf from **4a** or **4b** ................................... S41
    - 3.4. Reductive elimination of $MeOClO_3$ from **5a** ........................................ S45
    - 3.5. Reductive elimination of $MeONO_2$ from **6a** and **6a'** ............................ S47
    - 3.6. Reductive elimination of MeOC(O)$CF_3$ from **7a** and **7a'** ..................... S49
    - 3.7. Reductive elimination of MeBr from **8a** and **8a'** or from **8b** ................ S51
    - 3.8. Reductive elimination of MeCl from **9a** and **9a'** ................................. S56
    - 3.9. Reductive elimination of MeF from **10a** and **10a'** ............................... S58
    - 3.10. Reductive elimination of ($PMePh_3$)(OTf) from **13** ................................ S60
    - 3.11. Reaction of **13** with $NEt_3$. Formation of ($NMeEt_3$)(OTf) ....................... S66
4. Crystal structures ........................................... S67
5. Computational methods and data ........................................... S72
6. References ........................................... S76

## 1. General considerations, materials and methods

$Ph_3PAuMe$ was prepared as previously reported.[1] Unless otherwise stated, reactions were carried out at room temperature and under a $N_2$ atmosphere by using standard Schlenk techniques. Inhibitor-free HPLC grade $CH_2Cl_2$ (Fisher) or $CDCl_3$ (Eurisotop) were used as solvents in preparative-scale or NMR-tube reactions. Both were passed through an activated basic alumina column, degassed by $N_2$ bubbling or by three freeze-pump-thaw cycles ($CDCl_3$) and stored over 4 Å molecular sieves in a $N_2$ atmosphere and protected of light. Unless otherwise stated, the complexes were isolated in an air atmosphere using commercial solvents (HPLC or analytical grade). Irradiations were performed using a fluorescent lamp ($\lambda_{max}$ = 310 nm, 36 W).

NMR spectra were measured on Bruker Avance 200, 300, 400, 600 or Avance Neo 400 spectrometers. $^1H$ and $^{13}C\{^1H\}$ NMR spectra were referenced on the solvent signals.[2] $^{19}F$ and $^{31}P\{^1H\}$ spectra were referenced against external $CFCl_3$ or $H_3PO_4$, respectively.

Elemental analyses were carried out with a LECO CHNS-932 microanalyzer. Infrared spectra were recorded in the range 4000–200 cm$^{-1}$ on Perkin-Elmer Spectrum 65 FT-IR or Jasco FT-IR 4600 spectrometers using the ATR (Attenuated Total Reflection) technique. Melting points were determined on a Reichert apparatus in an air atmosphere.

Reductive elimination products MeX (X = Me, F, Cl, Br, I, $OC(O)CF_3$, $SC_6H_4OMe$-4) were identified by comparison of their NMR data with bibliography data or with NMR data of samples prepared in our laboratory. References for the NMR data of the uncommon methane derivatives with X = OTf, $OClO_4$ or $ONO_2$ are given in the corresponding sections.

### 2. Synthesis, Characterization and Spectra

**[Au(CF$_3$)(PPh$_3$)] (1a)**. $ICF_3$ (g) (1.02 mmol) was introduced by means of a syringe equipped with a needle in a septum-capped Schlenk tube containing a precooled (ca. -70 ºC) solution of $Ph_3PAuMe$ (432 mg, 0.911 mmol) in $CH_2Cl_2$ (20 mL). Then, the mixture was allowed to warm at room temperature with stirring and irradiated at 310 nm for 10 min. The colorless solution was evaporated to dryness under vacuum. The resulting colorless residue was stirred with with *n*-hexane (2 mL). The suspension was filtered and the colorless solid was washed with with *n*-hexane (2 × 2 mL) and air dried. Yield: 417 mg, 87%. $^1H$ NMR (200.1 MHz, $CDCl_3$): δ 7.57-7.46 (m, 15H, Ph). $^{19}F$ NMR (188.3 MHz, $CDCl_3$): δ -29.1 (d, $^3J_{PF}$ = 45.2 Hz). $^{31}P\{^1H\}$ NMR (81.0 MHz, $CDCl_3$): δ 38.8 (q, $^3J_{PF}$ = 45.1 Hz). The NMR data of the obtained complex were in agreement with those previously reported.[3]

**[Au(CF$_3$)(PCy$_3$)] (1b)**. $ICF_3$ (g) (2.5 mmol) was introduced by means of a syringe equipped with a needle in a Schlenk tube containing a solution of $Cy_3PAuMe$ (887 mg, 1.8 mmol) in $CH_2Cl_2$ (20 mL) at ca. -70 ºC. Then, the mixture was allowed to warm at room temperature with stirring and irradiated at 310 nm for 10 min. The colorless solution was evaporated to dryness under vacuum. The resulting colorless solid was washed with *n*-hexane (3 × 2 mL) and air dried. Yield: 875 mg, 89%. $^1H$ NMR (300.1 MHz, $CDCl_3$): δ 2.04-1.25 (several m, 33 H, Cy).$^{19}F$ NMR (282.4 MHz, $CDCl_3$): δ -29.9 (d, $^3J_{PF}$ = 41.2 Hz). $^{31}P\{^1H\}$ NMR (121.5 MHz, $CDCl_3$): δ 53.1 (q, $^3J_{PF}$ = 41.2 Hz). The NMR data of the obtained complex were in agreement with those previously reported.[4]

***trans*-[Au(CF$_3$)Br$_2$(PPh$_3$)] (2a)**. It was prepared by a modified literature method. A solution of **1a** (206 mg, 0.390 mmol) in $CH_2Cl_2$ (5 mL) was cooled at ca. -70 ºC,

protected from light and magnetically stirred. $Br_2$ (20 μL, 0.39 mmol) was added to the solution and the mixture was allowed to warm at room temperature. Then, a layer of *n*-hexane (10 mL) was carefully added on the resulting orange solution and the two phases were allowed to slowly mix at 5 °C during 16 h in the dark. The resulting orange crystals were washed with *n*-hexane (3 × 2 mL) and air dried. Yield: 195 mg, 73.8 %. Solutions of **2a** decomposed to [AuBr($PPh_3$)] and $CBrF_3$ when they were exposed to ambient light. $^1$H NMR (200.1 MHz, $CDCl_3$): δ 7.76-7.26 (m, 15H, Ph). $^{19}$F NMR (188.3 MHz, $CDCl_3$): δ -17.3 (d, $^3J_{PF}$ = 87.8 Hz). $^{31}$P{$^1$H} NMR (81.0 MHz, $CDCl_3$): δ 23.6 (q, $^3J_{PF}$ = 87.8 Hz). An useful $^{13}$C NMR spectrum was not obtained because the complex partially decomposed during the measurement. The NMR data of the complex agree with those previously reported.[5]

***trans*-[Au($CF_3$)$Br_2$($PCy_3$)] (2b)**. A solution of **1b** (495 mg, 0.906 mmol) in $CH_2Cl_2$ (5 mL) was cooled at ca. -70 °C, protected from light and magnetically stirred. $Br_2$ (46.4 μL, 0.906 mmol) was added to the solution and the mixture was allowed to warm at room temperature. The mixture was evaporated to dryness under vacuum. The crude was chromatographed on a silica gel column using $CH_2Cl_2$/*n*-hexane (2:1) as eluent. The collected orange fraction (Rf = 0.88) was evaporated to dryness and the residue was washed with *n*-pentane (3 × 2 mL), to give an orange solid, which was dried under vacuum. Yield: 545 mg, 85 %. Solutions of **2b** decomposed to [AuBr($PCy_3$)] and $CBrF_3$ when they were exposed to ambient light. M.p. 233–235 °C (d). Anal. Calcd for $C_{19}H_{33}AuBr_2F_3P$: C, 32.31; H, 4.71. Found: C, 32.14; H, 4.83. IR (cm$^{-1}$): 1176, 1064, 1046, 1003 ν(C–F). $^1$H NMR (300.1 MHz, $CD_2Cl_2$): δ 2.74 (m, 3 H, PCH), 1.99-1.32 (several m, 30H, Cy). $^{13}$C{$^1$H} NMR (150.9 MHz, $CD_2Cl_2$): δ 137.6 (qd, $^1J_{FC}$ = 377.6 Hz, $^2J_{PC}$ = 274.6 Hz, $CF_3$), 32.5 (d, $^1J_{PC}$ = 21.8 Hz, Cy), 29.6 (d, $^3J_{PC}$ = 2.8 Hz, Cy), 27.8 (d, $^2J_{PC}$ = 11.4 Hz, Cy), 26.4 (s, Cy).$^{19}$F NMR (282.4 MHz, $CD_2Cl_2$): δ -20.6 (d, $^3J_{PF}$ = 82.7 Hz). $^{31}$P{$^1$H} NMR (121.5 MHz, $CD_2Cl_2$): δ 35.4 (q, $^3J_{PF}$ = 82.7 Hz).

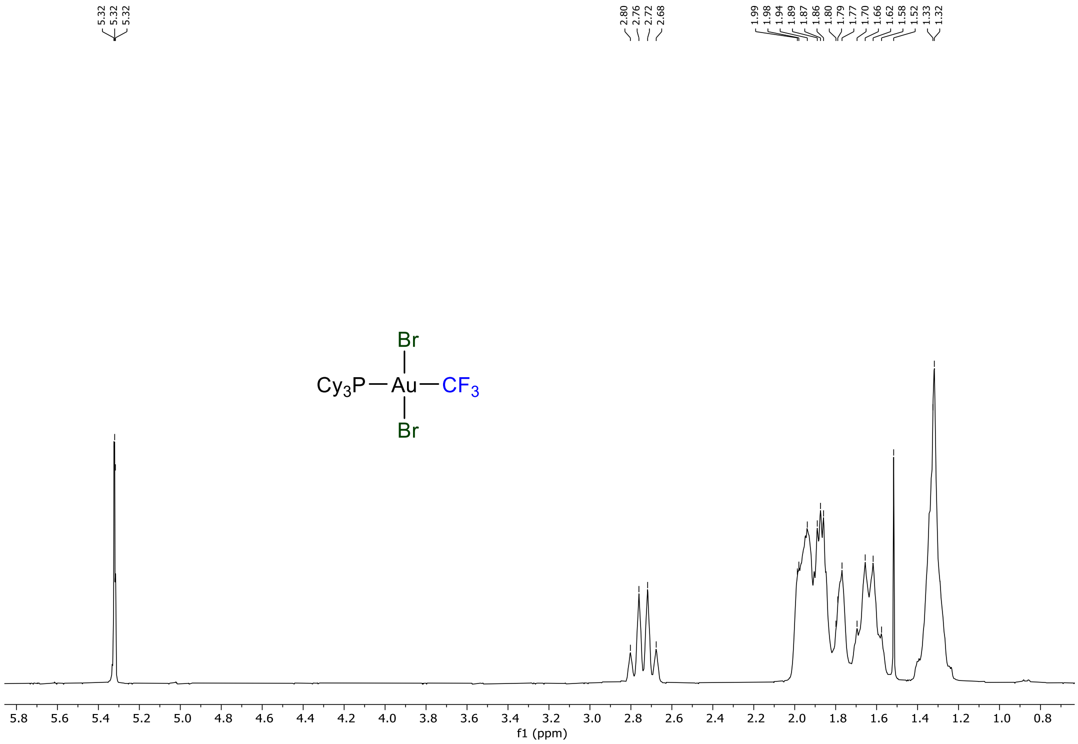


**Figure S1**. $^1$H NMR spectrum (300.1 MHz, $CD_2Cl_2$) of **2b**.

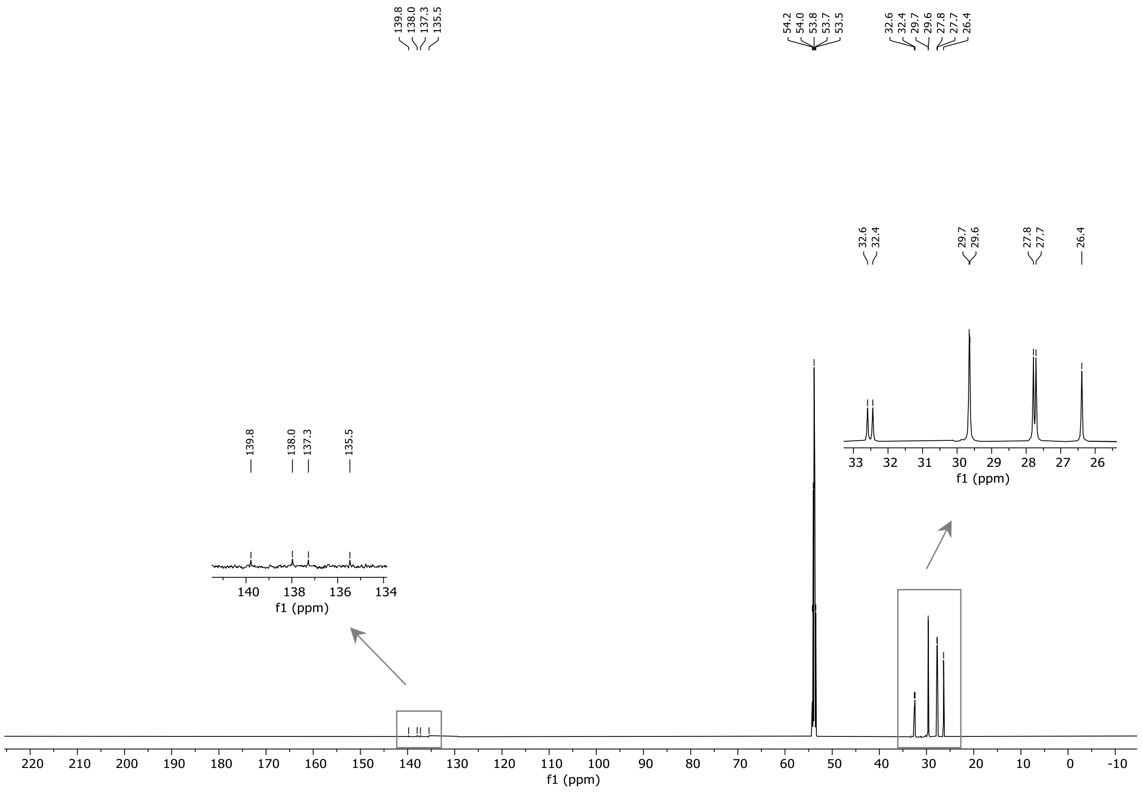


**Figure S2**. $^{13}C\{^{1}H\}$ NMR spectrum (150.9 MHz, $CD_2Cl_2$) of **2b**.

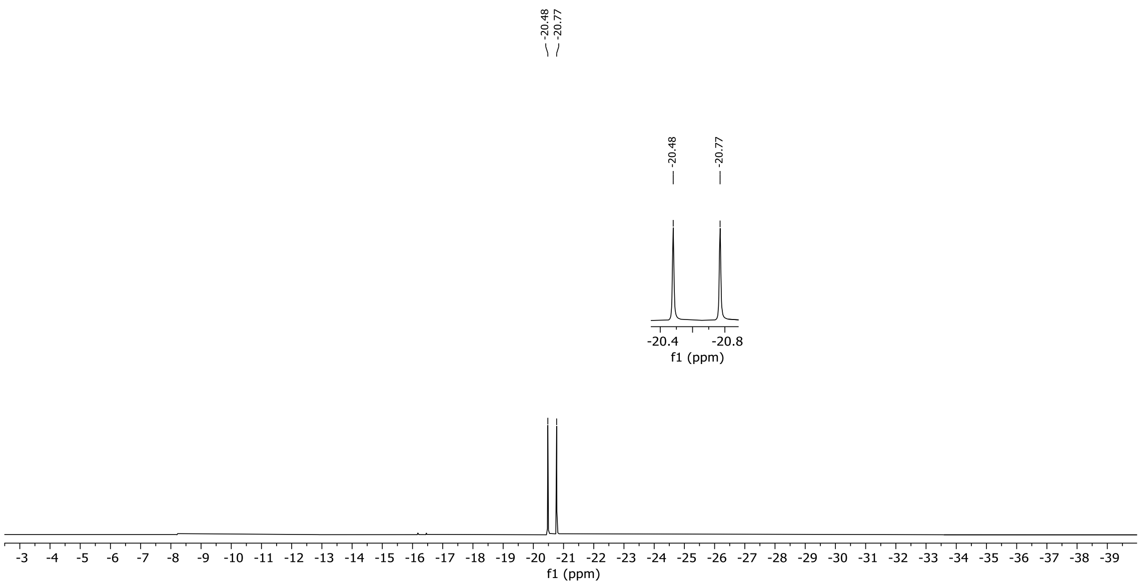


**Figure S3**. $^{19}F$ NMR spectrum (282.4 MHz, $CD_2Cl_2$) of **2b**.

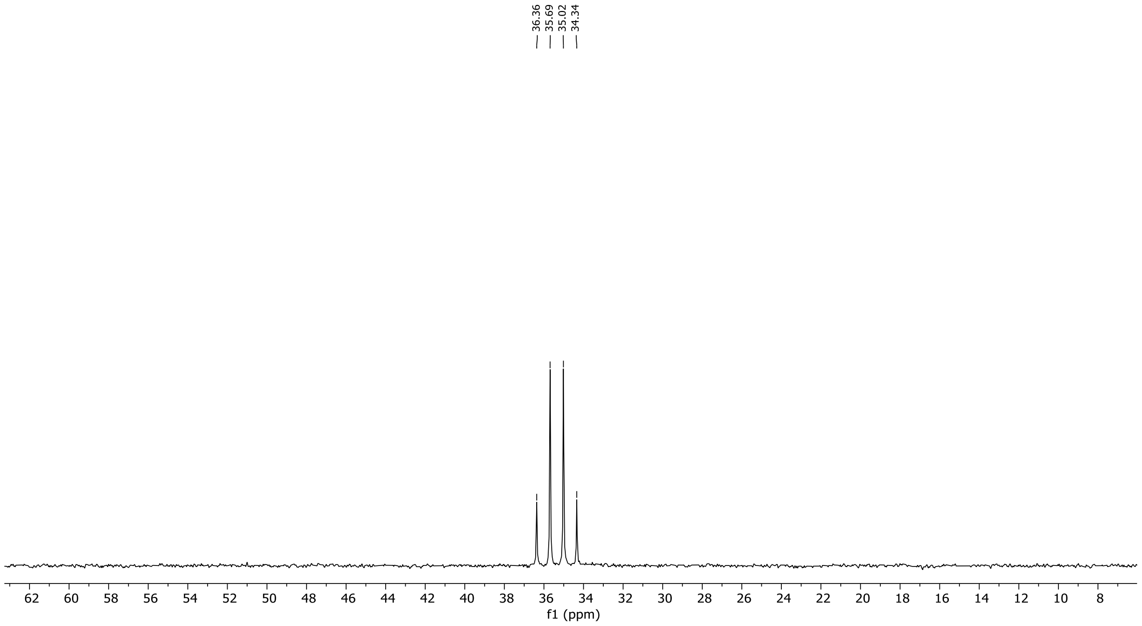


**Figure S4**. $^{31}P\{^1H\}$ NMR spectrum (121.5 MHz, $CD_2Cl_2$) of **2b**.

***trans*-[Au($CF_3$)$Me_2$($PPh_3$)] (3a)**. MgMeBr (3.0 M solution in $Et_2O$, 0.42 mmol) was added to a solution of **2a** (94 mg, 0.14 mmol) in THF (15 mL) at ca. -70 ºC with protection from light. The mixture was allowed to warm at room temperature and stirred for 30 min in the dark. Then, $H_2O$ (0.1 mL) was added and the mixture was stirred for 15 min and evaporated to dryness under vacuum. The residue was stirred with $CH_2Cl_2$ (20 mL) and excess of $MgSO_4$ for 40 min. The suspension was filtered over celite. The solid material was extracted with more $CH_2Cl_2$ (30 mL) and the combined $CH_2Cl_2$ solutions were evaporated to dryness under vacuum. The residue was suspended in *n*-pentane (3 mL) and the suspension was filtered. The colorless solid was washed with *n*-pentane (2 × 3 mL) and air dried. Yield: 55 mg, 72 %. M.p. 165–167 ºC (d). Anal. Calcd for $C_{21}H_{21}AuF_3P$: C, 45.18; H, 3.79. Found: C, 45.27; H, 3.73. IR ($cm^{-1}$): 1098, 1043, 1022, 999 ν(C–F). $^1H$ NMR (300.1 MHz, $CDCl_3$): δ 7.55–7.47 (m, 15H, Ph), 0.19 (dq, $^3J_{PH}$ = 5.4 Hz, $^4J_{FH}$ = 0.8 Hz, 6H, Me). $^{13}C\{^1H\}$ NMR (150.9 MHz, $CD_2Cl_2$): δ 138.1 (qd, $^1J_{FC}$ = 356.3 Hz, $^2J_{PC}$ = 250.6 Hz, $CF_3$), 134.7 (d, $J_{PC}$ = 10.8 Hz, *o*-Ph), 132.2 (d, $J_{PC}$ = 2.8 Hz, *p*-Ph), 129.3 (d, $J_{PC}$ = 11.2 Hz, *m*-Ph), 126.9 (d, $J_{PC}$ = 57.3 Hz, *i*-Ph), 12.4 (m, Me). $^{19}F$ NMR (282.4 MHz, $CDCl_3$): δ -32.6 (d, $^3J_{PF}$ = 64.2 Hz). $^{31}P$ NMR (121.5 MHz, $CDCl_3$): δ 27.5 (q, $^3J_{PF}$ = 64.2 Hz).

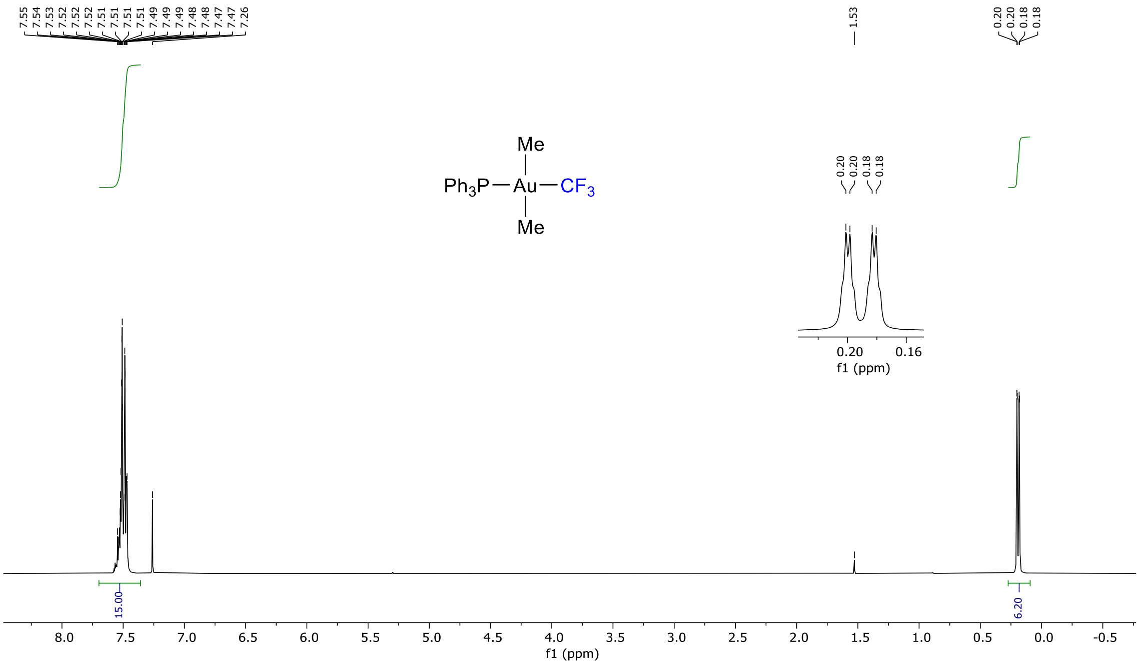


**Figure S5**. $^{1}$H NMR spectrum (300.1 MHz, $CDCl_3$) of **3a**.

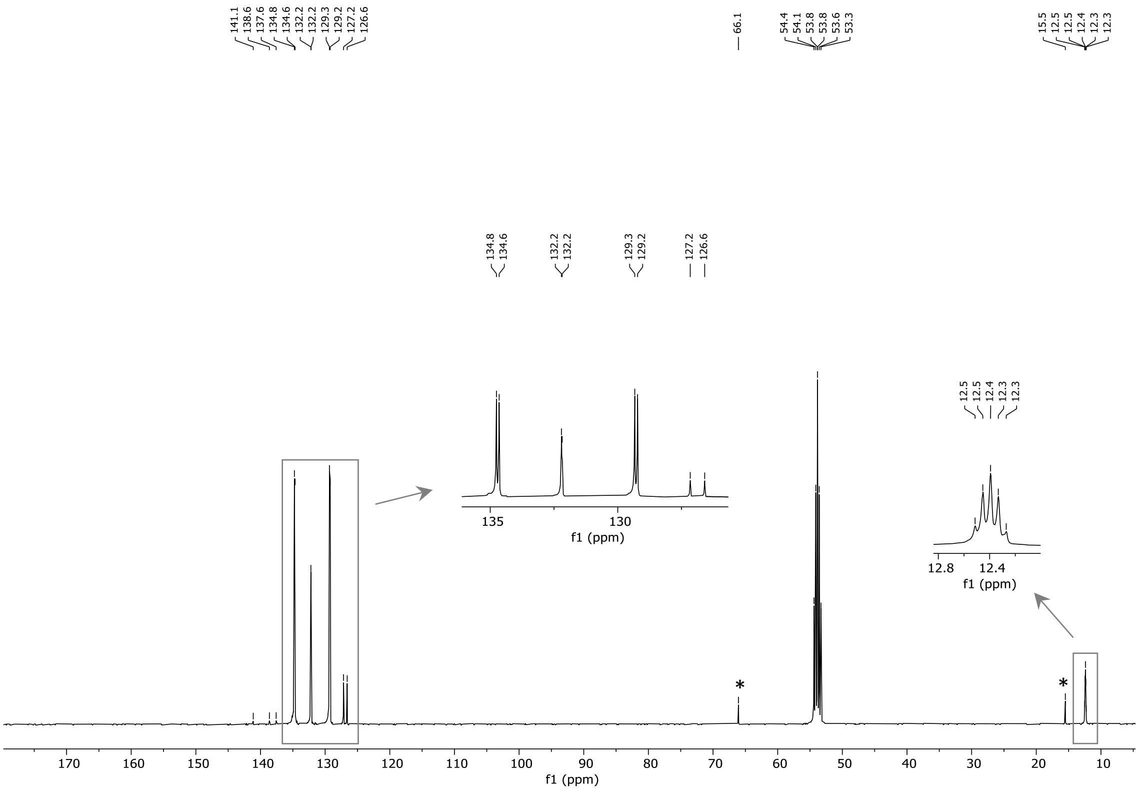


**Figure S6**. $^{13}C\{^{1}H\}$ NMR spectrum (100.8 MHz, $CDCl_3$) of **3a**. The signals marked with an asterisk correspond to $Et_2O$.

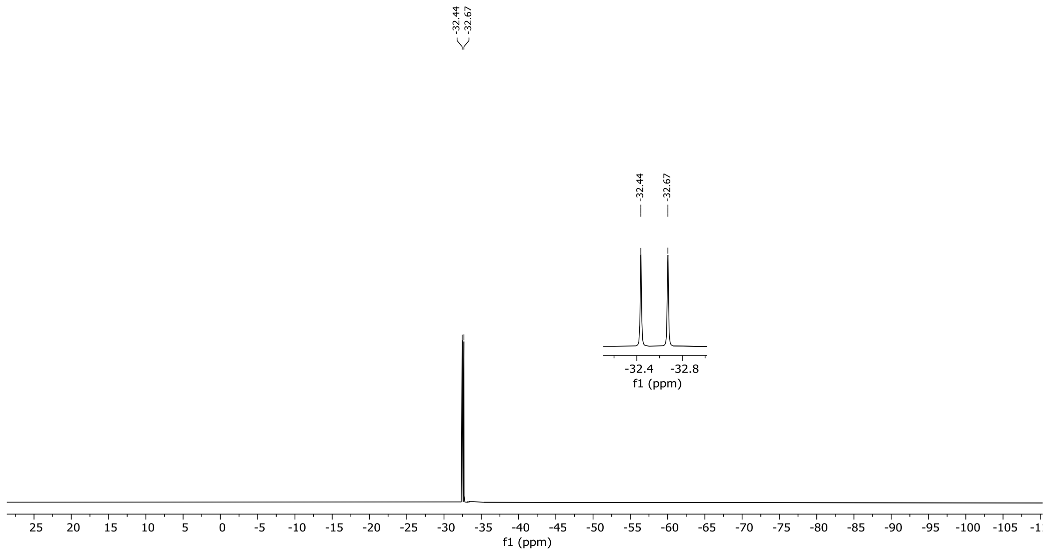


**Figure S7**. $^{19}$F NMR spectrum (282.4 MHz, $CDCl_3$) of **3a**.

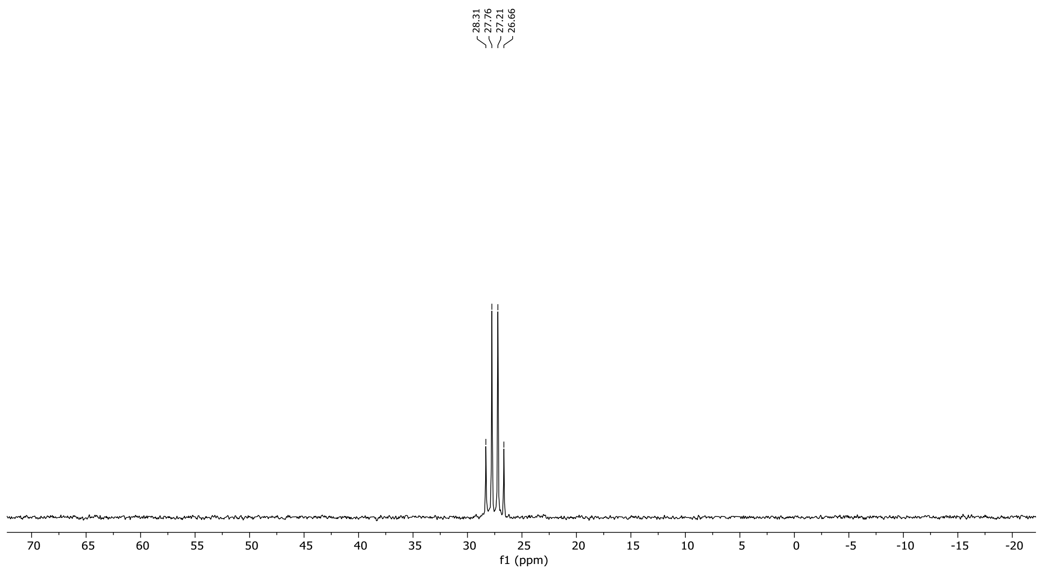


**Figure S8**. $^{31}$P{$^{1}$H} NMR spectrum (121.5 MHz, $CDCl_3$) of **3a**.

***trans*-[Au(CF$_3$)Me$_2$(PCy$_3$)]** (**3b**). MgMeBr (3.0 M solution in $Et_2O$, 1.0 mmol) was added to a solution of **2b** (240 mg, 0.34 mmol) in THF (15 mL) at ca. -70 ºC protected from light. The mixture was allowed to warm at room temperature and stirred for 30 min in the dark. The resulting solution was cooled at 0 ºC and hydrolyzed by addition $H_2O$ (15 mL). $Et_2O$ (2 × 25 mL) was added and the mixture was stirred for 2 min. The organic layer was separated and the aqueous one was extracted with $Et_2O$ (2 × 10 mL). The combined organic extracts were washed with saturated NaCl solution (3 × 25 mL) and dried with $Na_2SO_4$. The extract was filtered and evaporated to dryness under vacuum. The residue was chromatographed on a silica gel column using $CH_2Cl_2$/*n*-hexane (1:4) as eluent. The collected colourless fraction (Rf = 0.38) was evaporated to dryness and the residue was washed with *n*-pentane (3 × 2 mL), to give a colorless solid, which was air dried. Yield: 165 mg, 84 %. M.p. 215 ºC. Anal. Calcd for $C_{21}H_{39}AuF_3P$: C, 43.75; H, 6.82. Found: C, 43.65; H, 6.85. IR (cm-1): 1176, 1120, 1100, 1041, 1004, 978 ν(C–F). $^{1}$H NMR (300.1 MHz, $CDCl_3$): δ 2.43 (m, 3 H, PCH), 1.89-1.24 (several m, 30 H, Cy), 0.30 (dd, $^{3}J_{PH}$ = 3.7 Hz, $^{4}J_{FH}$ = 1.0 Hz, 6H, Me). $^{13}$C{$^{1}$H} NMR (75.5 MHz, $CDCl_3$): δ

140.1 (qd, ${}^{1}J_{FC}$ = 359.6 Hz, ${}^{2}J_{PC}$ = 233.1 Hz, $CF_3$), 31.8 (d, ${}^{1}J_{PC}$ = 25.6 Hz, Cy), 29.5 (d, ${}^{3}J_{PC}$ = 1.9 Hz, Cy), 27.6 (d, ${}^{2}J_{PC}$ = 11.0 Hz, Cy), 26.3 (d, ${}^{4}J_{PC}$ = 1.6 Hz, Cy), 7.9 (m, Me).${}^{19}$F NMR (282.4 MHz, $CDCl_3$): δ -34.7 (d, ${}^{3}J_{PF}$ = 62.4 Hz). ${}^{31}$P{${}^{1}$H} NMR (121.5 MHz, $CDCl_3$): δ 24.4 (q, ${}^{3}J_{PF}$ = 62.4 Hz).

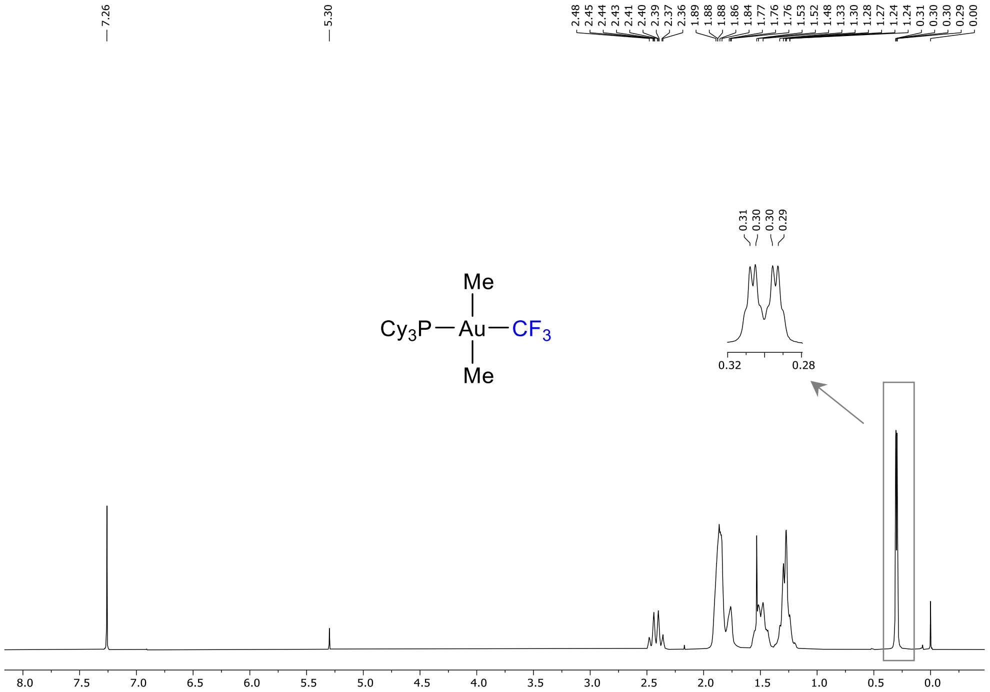


**Figure S9**. ${}^{1}$H NMR spectrum (300.1 MHz, $CDCl_3$) of **3b**.

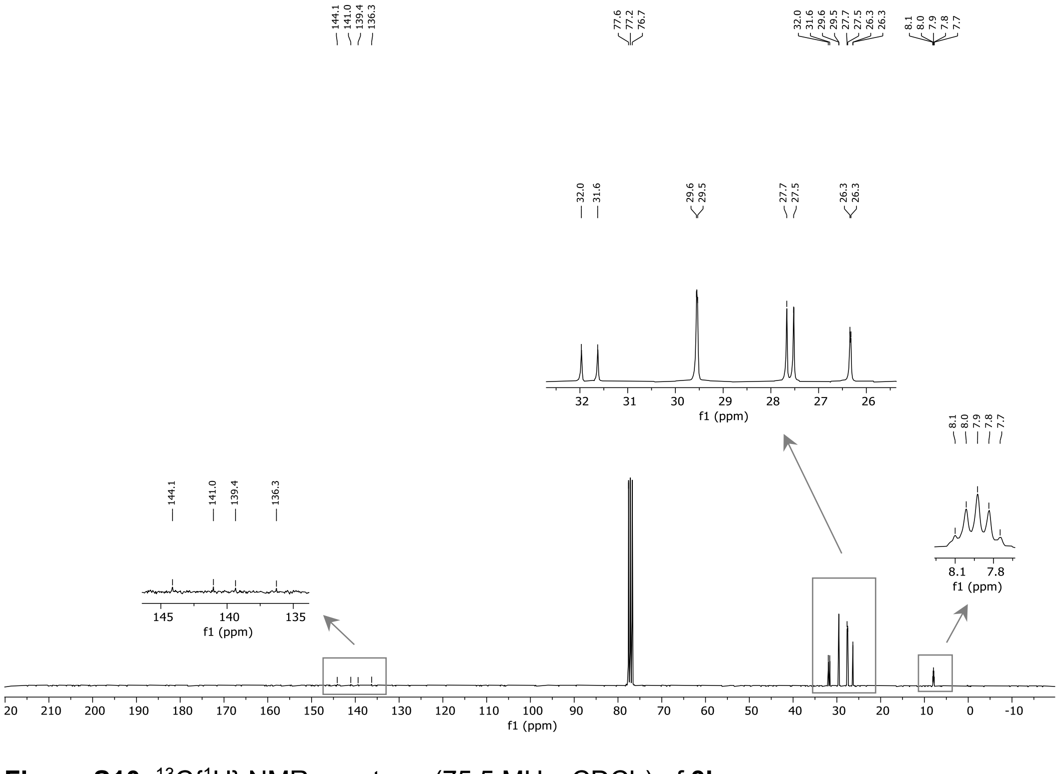


**Figure S10**. ${}^{13}$C{${}^{1}$H} NMR spectrum (75.5 MHz, $CDCl_3$) of **3b**.

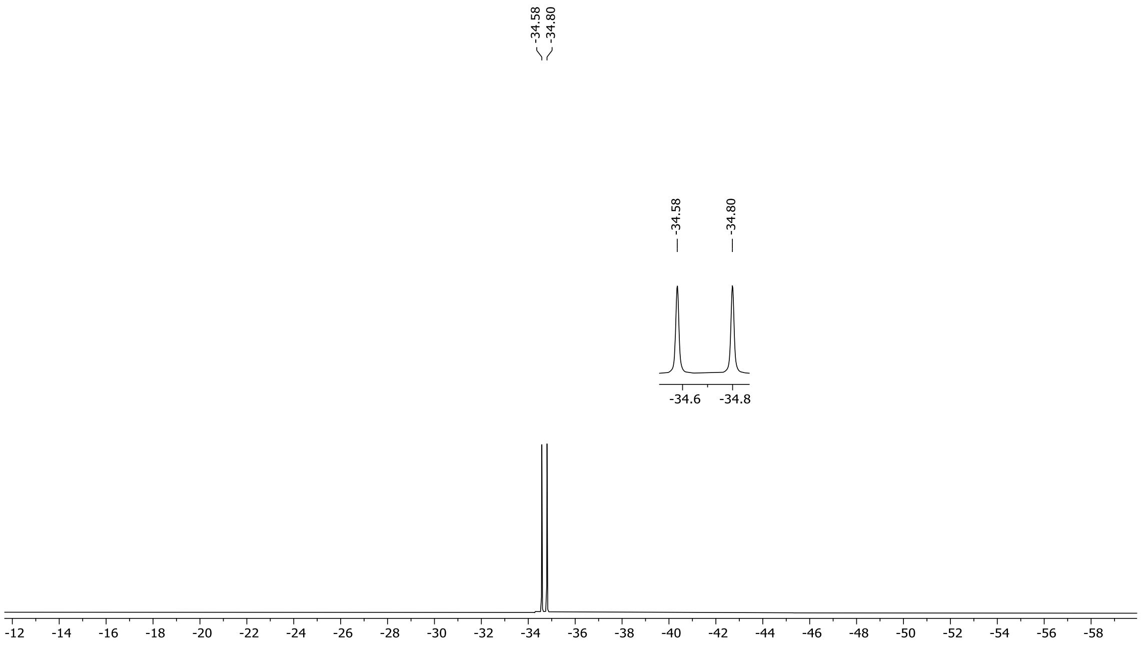


**Figure S11**. $^{19}$F NMR spectrum (282.4 MHz, $CDCl_3$) of **3b**.

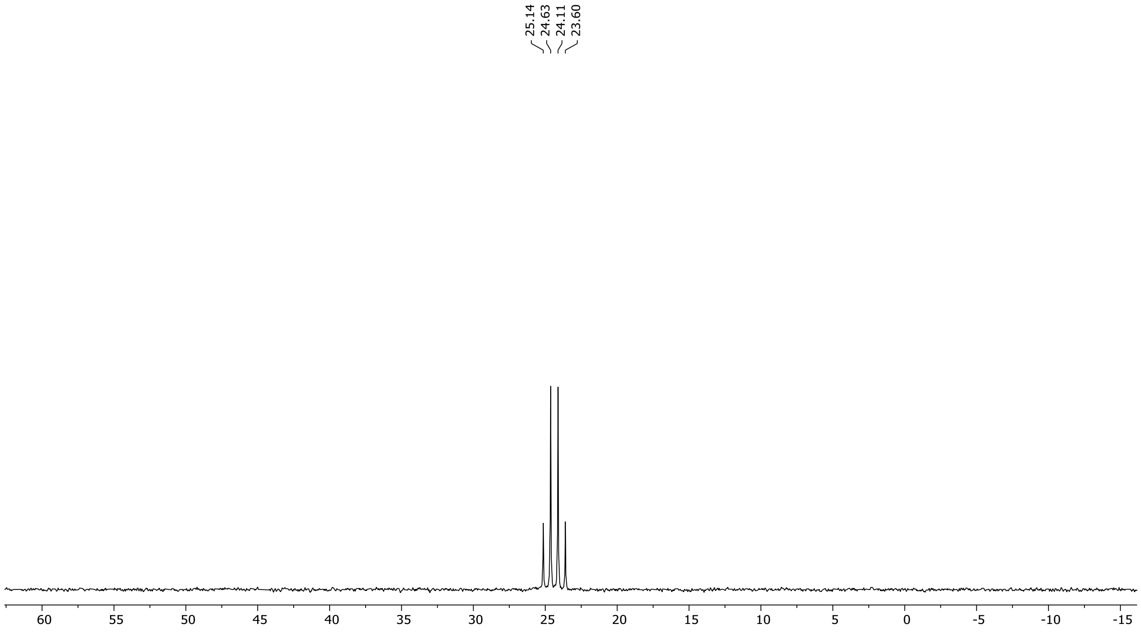


**Figure S12**. $^{31}$P{$^{1}$H} NMR spectrum (121.5 MHz, $CDCl_3$) of **3b**.

***SP-4-3*-[Au($CF_3$)(Me)(OTf)($PPh_3$)] (4a) and *SP-4-4*-[Au($CF_3$)(Me)(OTf)($PPh_3$)] (4a')**. HOTf (2 µL, 0.023 mmol) was added to a solution of **3a** (13 mg, 0.023 mmol) in $CDCl_3$ (0.5 mL) at room temperature. The NMR tube was capped and shaken. After 20 min the NMR spectra showed formation of **4a** (main product), together with smaller amounts of metallic gold and the decomposition products [Au($PPh_3$)$_2$](OTf) and MeOTf. NMR data of **4a**: $^{1}$H NMR (300.1 MHz, $CDCl_3$): δ 7.65–7.56 (m, 15H, Ph), 1.56 (d, $^{3}J_{PH}$ = 6.2 Hz, 3H, Me).$^{19}$F NMR (282.4 MHz, $CDCl_3$): δ -34.0 (d, $^{3}J_{PF}$ = 71.4 Hz, Au$CF_3$), -77.3 (s, TfO). $^{31}$P NMR (121.5 MHz, $CDCl_3$): δ 31.8 (q, $^{3}J_{PF}$ = 71.4 Hz).

Very small amounts of **4a'** (< 4%) were observed in some samples. Representative NMR data of **4a'** are given in Table S2.

The attempts to isolate **4a** from larger-scale reactions in $CH_2Cl_2$ gave rise to impure oils containing large amounts of $[Au(PPh_3)_2]OTf$.

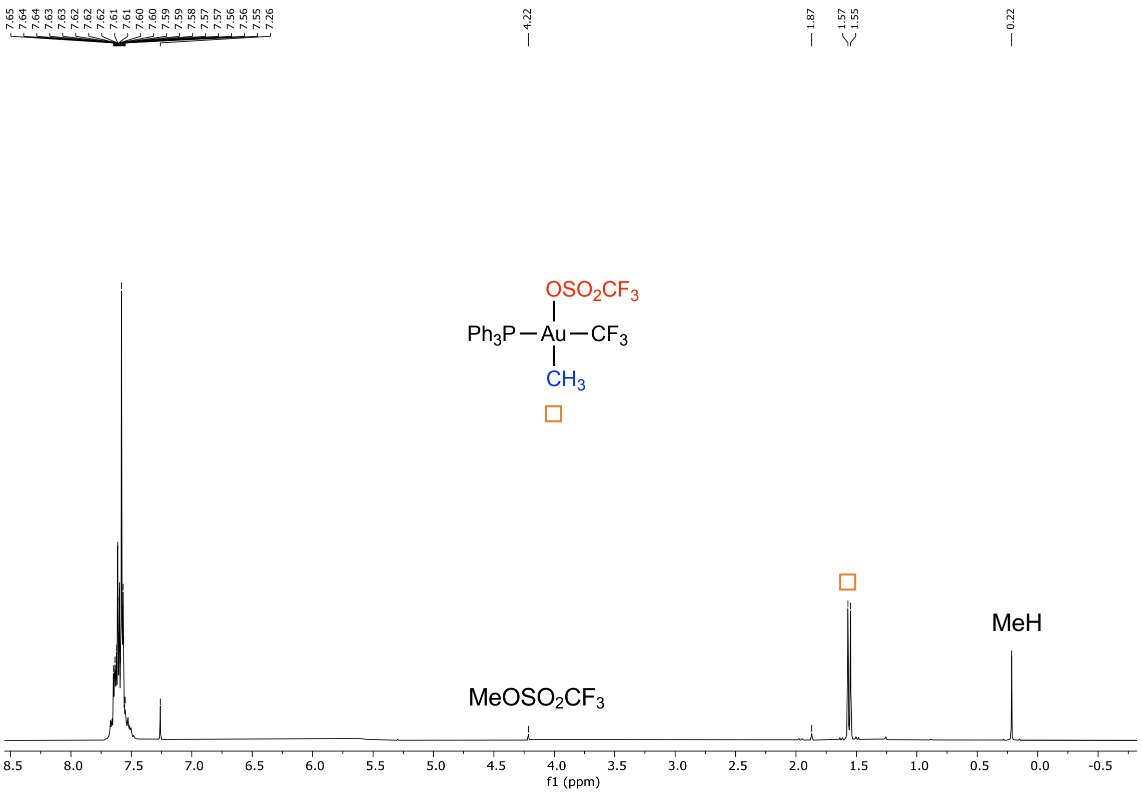


**Figure S13**. $^{1}H$ NMR spectrum (300.1 MHz, $CDCl_3$) of in situ generated **4a**.

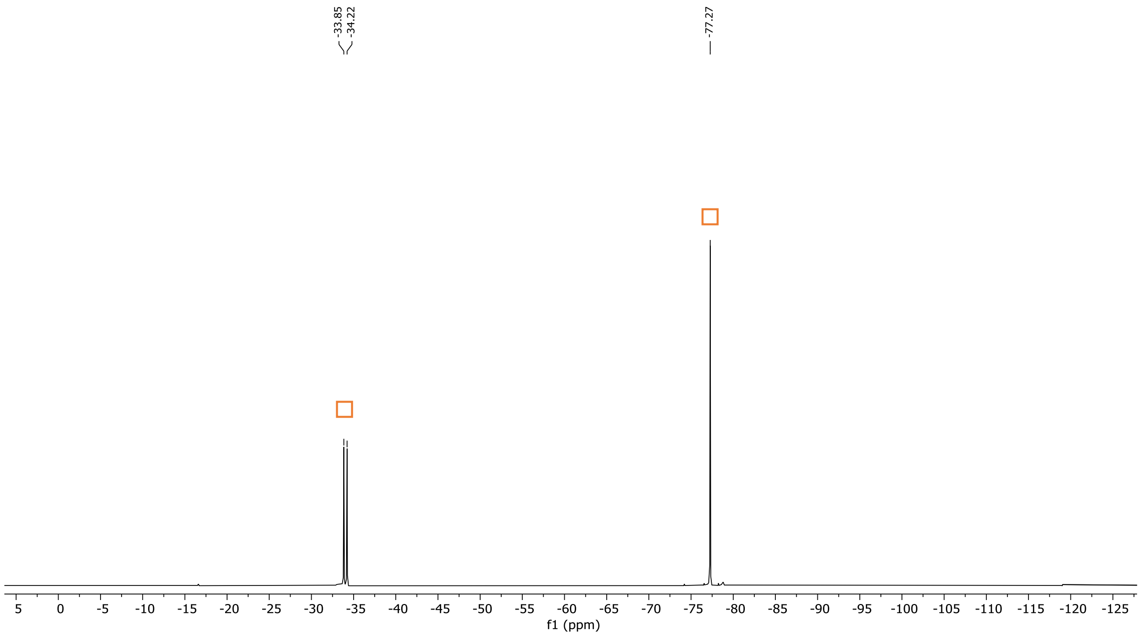


**Figure S14**. $^{19}F$ NMR spectrum (282.4 MHz, $CDCl_3$) of in situ generated **4a**.

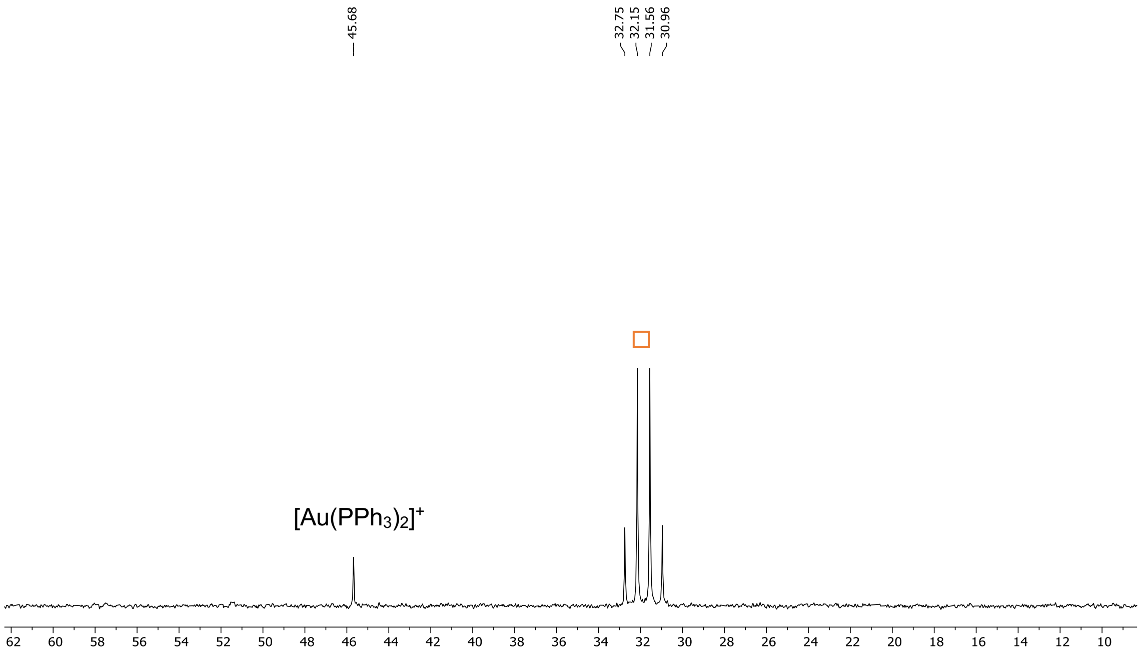


**Figure S15**. $^{31}P\{^1H\}$ NMR spectrum (121.5 MHz, $CDCl_3$) of in situ generated **4a**.

***SP-4-3*-[Au(CF$_3$)(Me)(OTf)(PCy$_3$)] (4b)**. HOTf (2.0 µL, 0.02 mmol) was added to a solution of **3b** (13 mg, 0.02 mmol) in $CDCl_3$ (0.5 mL) at room temperature. The NMR tube was closed and shaken. After 20 min the NMR spectra showed formation of **4b** (main product), together with smaller amounts of metallic gold and decomposition products [Au(PCy$_3$)$_2$](OTf) and MeOTf. NMR data of **4b**: $^1H$ NMR (300.1 MHz, $CDCl_3$): δ 2.50 (m, 3 H, PCH), 1.95-1.33 (several m, 30 H, Cy), 1.72 (d, $^3J_{PH}$ = 4.6 Hz, 3H, Me). $^{19}F$ NMR (282.4 MHz, $CDCl_3$): δ -36.8 (d, $^3J_{PF}$ = 65.2 Hz, AuCF$_3$), -76.5 (s, TfO). $^{31}P$ NMR (121.5 MHz, $CDCl_3$): δ 40.1 (q, $^3J_{PF}$ = 65.2 Hz).

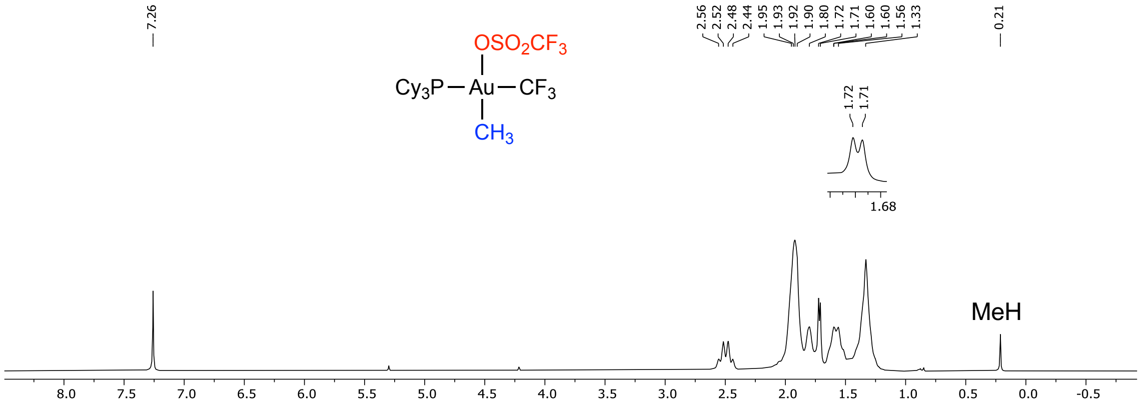


**Figure S16**. $^1H$ NMR spectrum (300.1 MHz, $CDCl_3$) of in situ generated **4b.**

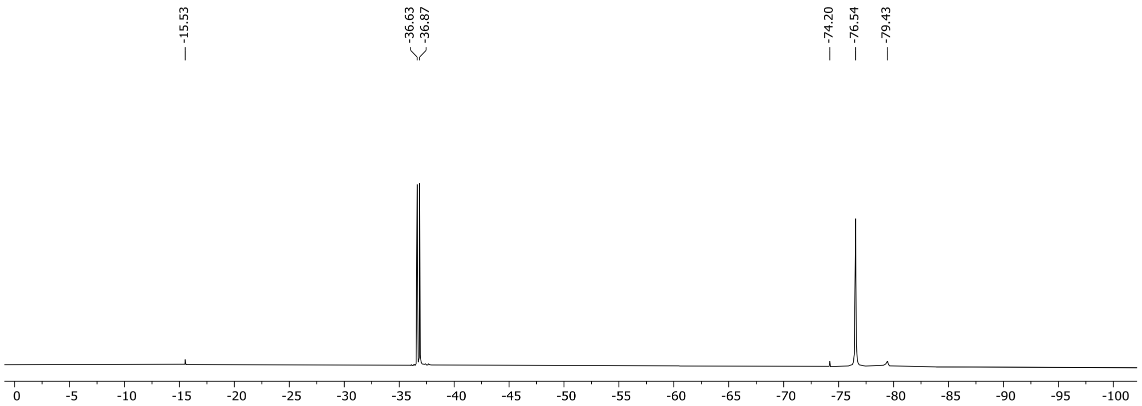


**Figure S17**. $^{19}F$ NMR spectrum (282.4 MHz, $CDCl_3$) of in situ generated **4b**.

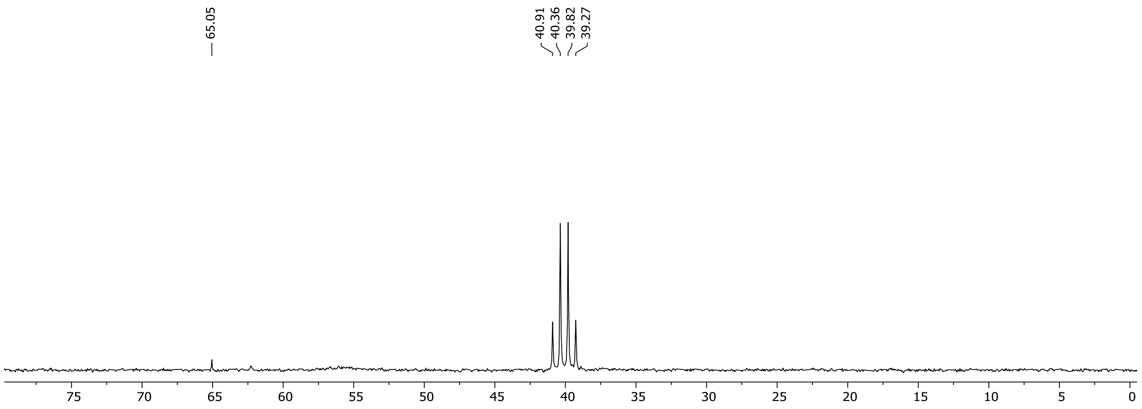


**Figure S18**. $^{31}P\{^1H\}$ NMR spectrum (121.5 MHz, $CDCl_3$) of in situ generated **4b**.

***SP-4-3*-[Au(CF$_3$)(Me)(OClO$_3$)(PPh$_3$)] (5a) and *SP-4-4*-[Au(CF$_3$)(Me)(OClO$_3$)(PPh$_3$)] (5a')**. 72% Aqueous $HClO_4$ (3 µL, 0.04 mmol) was added to a solution of **3a** (14 mg, 0.025 mmol) in $CDCl_3$ (0.5 mL) at room temperature. The NMR tube was capped and shaken. After 20 min the NMR spectra showed formation of **5a** (main product), together with smaller amounts of metallic gold and decomposition products $[Au(PPh_3)_2](ClO_4)$ and $MeOClO_3$. NMR data of **5a**: $^1H$ NMR (300.1 MHz, $CDCl_3$): δ 7.65–7.54 (m, 15H, Ph), 1.61 (dd, $^3J_{PH}$ = 6.0 Hz, $^4J_{FH}$ = 0.5 Hz, 3H, Me).$^{19}F$ NMR (282.4 MHz, $CDCl_3$): δ -34.4 (d, $^3J_{PF}$ = 72.8 Hz, $AuCF_3$). $^{31}P$ NMR (121.5 MHz, $CDCl_3$): δ 32.3 (q, $^3J_{PF}$ = 72.8 Hz).

Partial isomerization of **5a** to **5a'** was observed when solutions of **5a** were left at room or higher temperature. The amount of **5a'** increased up to a maximum value of 5 %. Representative NMR data of **5a'** are given in Table S2.

The attempts to isolate **5a** from larger-scale reactions in $CH_2Cl_2$ gave rise to impure oils containing large amounts of $[Au(PPh_3)_2]ClO_4$.

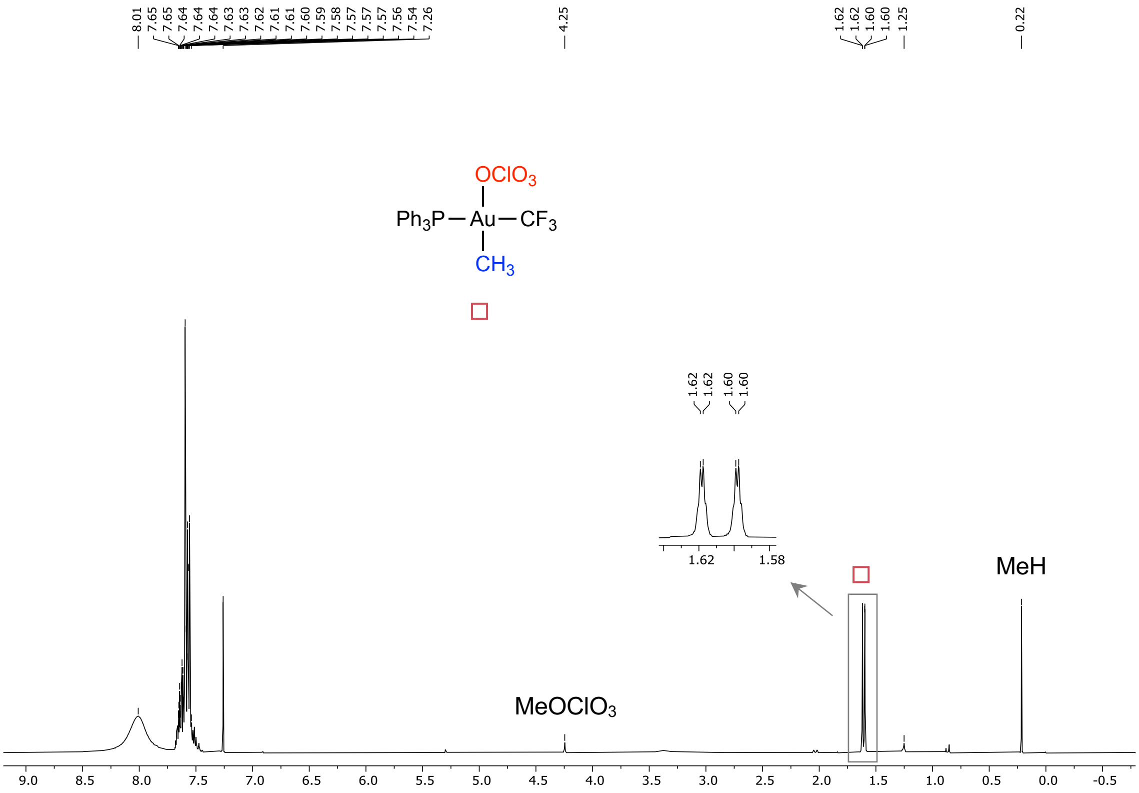


**Figure S19**. $^{1}H$ NMR spectrum (300.1 MHz, $CDCl_3$) of in situ generated **5a**.

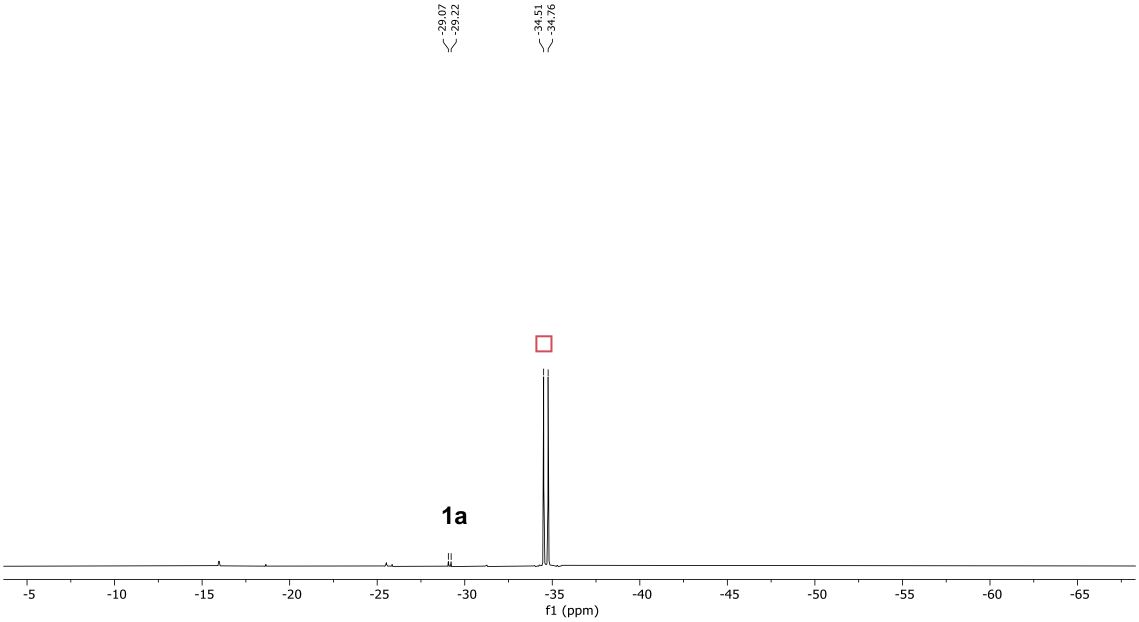


**Figure S20**. $^{19}F$ NMR spectrum (282.4 MHz, $CDCl_3$) of in situ generated **5a**.

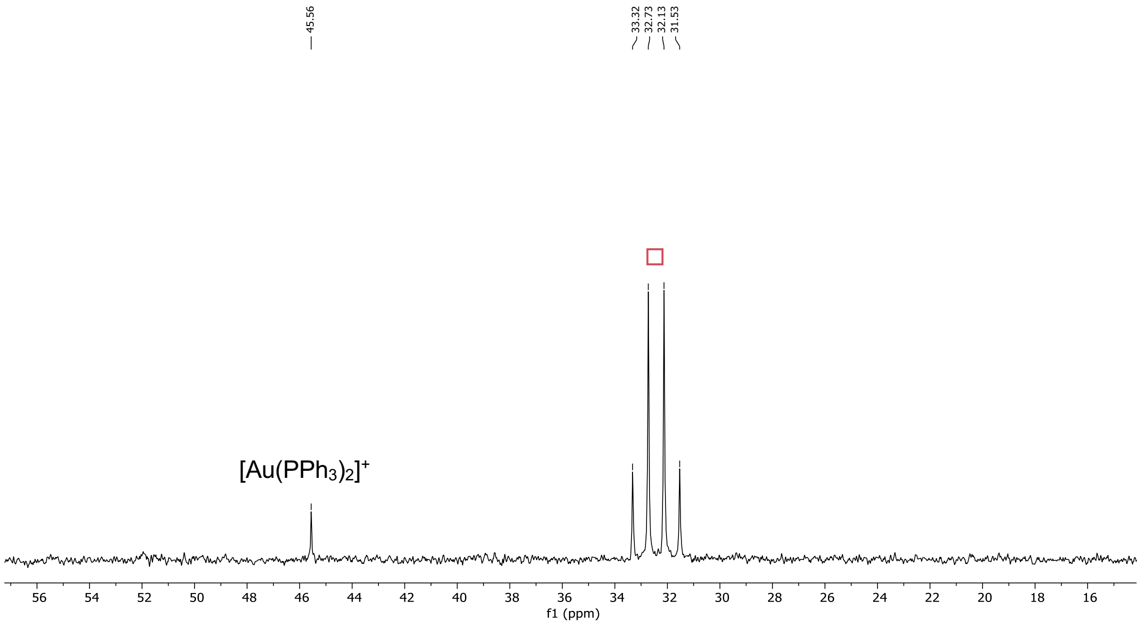


**Figure S21**. $^{31}P\{^1H\}$ NMR spectrum (121.5 MHz, $CDCl_3$) of in situ generated **5a**.

***SP-4-3*-[Au(CF$_3$)(Me)(ONO$_2$)(PPh$_3$)] (6a) and *SP-4-4*-[Au(CF$_3$)(Me)(ONO$_2$)(PPh$_3$)] (6a')**. 65% aqueous $HNO_3$ (13 µL, 0.19 mmol) was added to a solution of **3a** (70 mg, 0.13 mmol) in $CH_2Cl_2$ (5 mL) at room temperature. The mixture was stirred for 30 min and evaporated to dryness under vacuum. The residue was suspended in *n*-pentane (2 mL) and the suspension was filtered. The solid was washed with *n*-pentane (2 × 2 mL) and air dried to give **6a** as a colorless solid. Yield: 57 mg, 75 %. M.p. 148–150 ºC (d). Anal. Calcd for $C_{20}H_{18}AuNO_3F_3P$: C, 39.69; H, 3.00; N, 2.31. Found: C, 39.68; H, 2.98; N, 2.09. IR (cm$^{-1}$): 1273, 1241, 1100, 1068, 1043, 966 ν(C–F). $^1H$ NMR (600.1 MHz, $CDCl_3$): δ 7.64–7.53 (m, 15H, Ph), 1.38 (d, $^3J_{PH}$ = 5.9 Hz, 3H, Me). $^{13}C\{^1H\}$ NMR (150.9 MHz, $CDCl_3$): δ 139.0 (qd, $^1J_{FC}$ = 365.7 Hz, $^2J_{PC}$ = 242.3 Hz, $CF_3$), 134.3 (d, $J_{PC}$ = 11.0 Hz, *o*-Ph), 132.9 (d, $J_{PC}$ = 2.8 Hz, *p*-Ph), 129.7 (d, $J_{PC}$ = 11.1 Hz, *m*-Ph), 124.3 (d, $J_{PC}$ = 56.0 Hz, *i*-Ph), 8.3 (m, Me).$^{19}F$ NMR (282.4 MHz, $CDCl_3$): δ -36.4 (d, $^3J_{PF}$ = 71.7 Hz). $^{31}P$ NMR (121.5 MHz, $CDCl_3$): δ 29.0 (q, $^3J_{PF}$ = 71.7 Hz).

Partial isomerization of **6a** to **6a'** was observed when solutions of **6a** were left at room or higher temperature. The amount of **6a'** increased up to a maximum value of 12 %. Representative NMR data of **6a'** are given in Table S2.

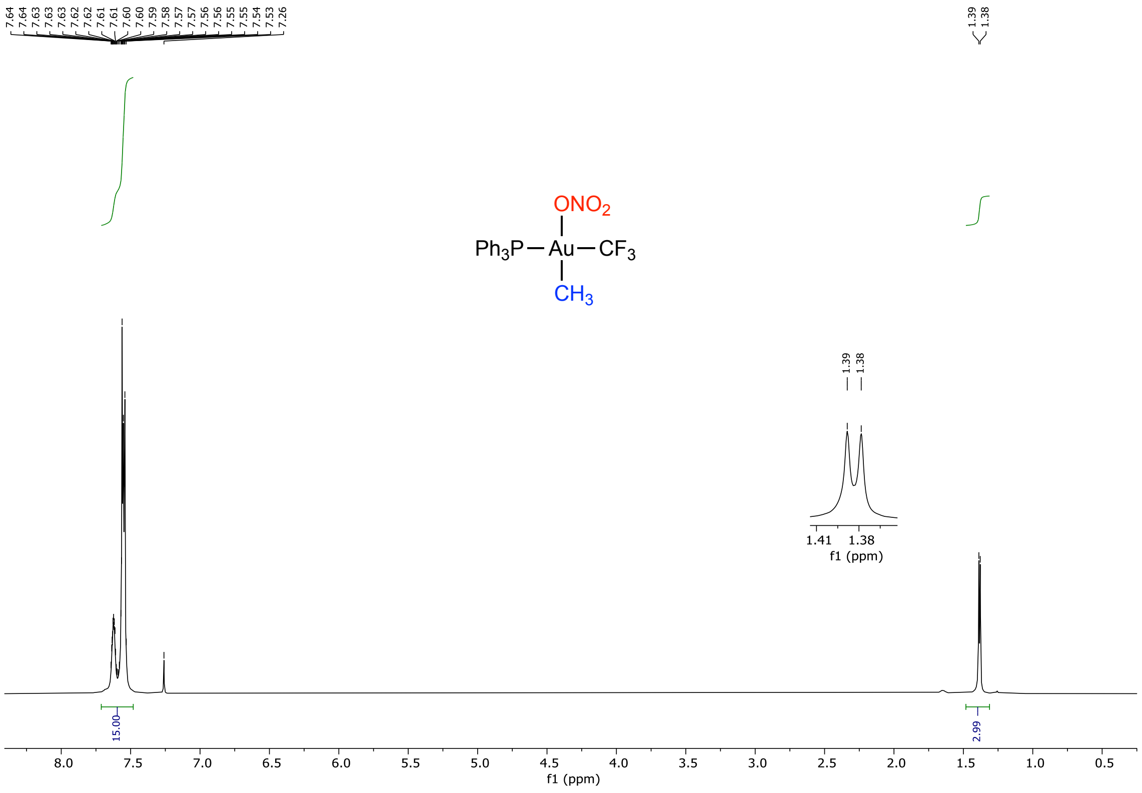

**Figure S22**. $^{1}$H NMR spectrum (600.1 MHz, $CDCl_3$) of **6a**.

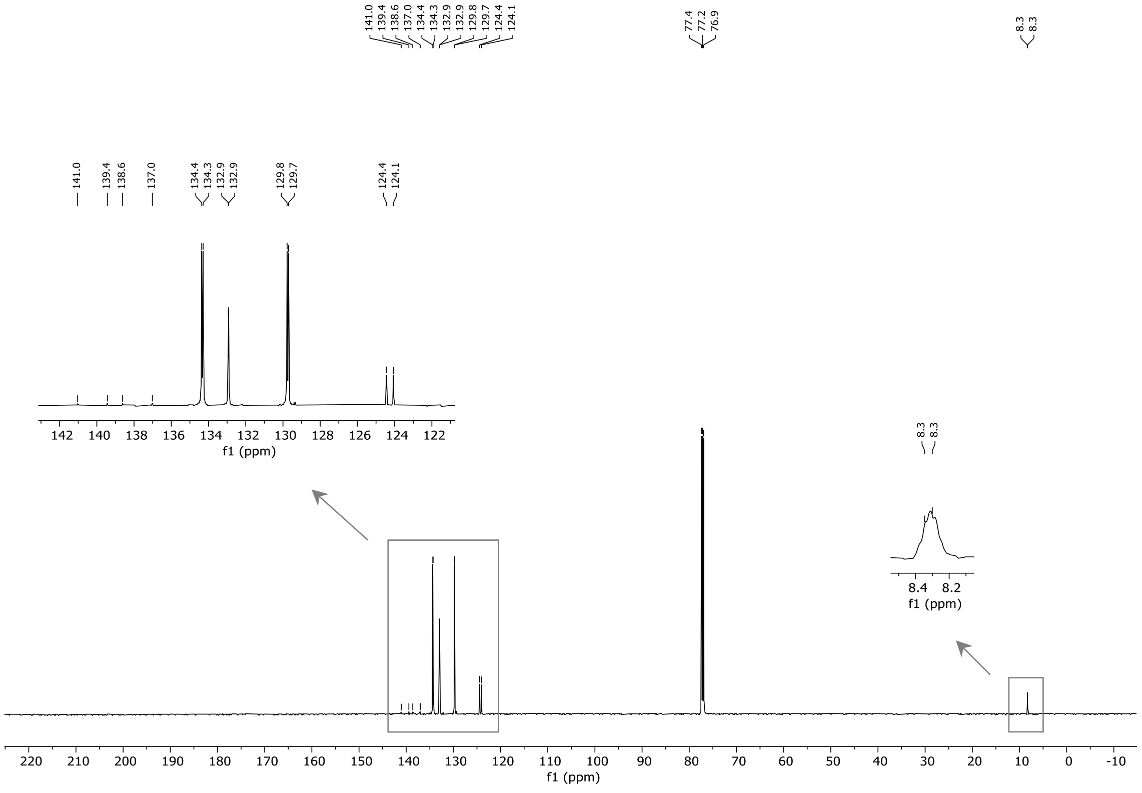

**Figure S23**. $^{13}C\{^{1}H\}$ NMR spectrum (150.9 MHz, $CDCl_3$) of **6a**.

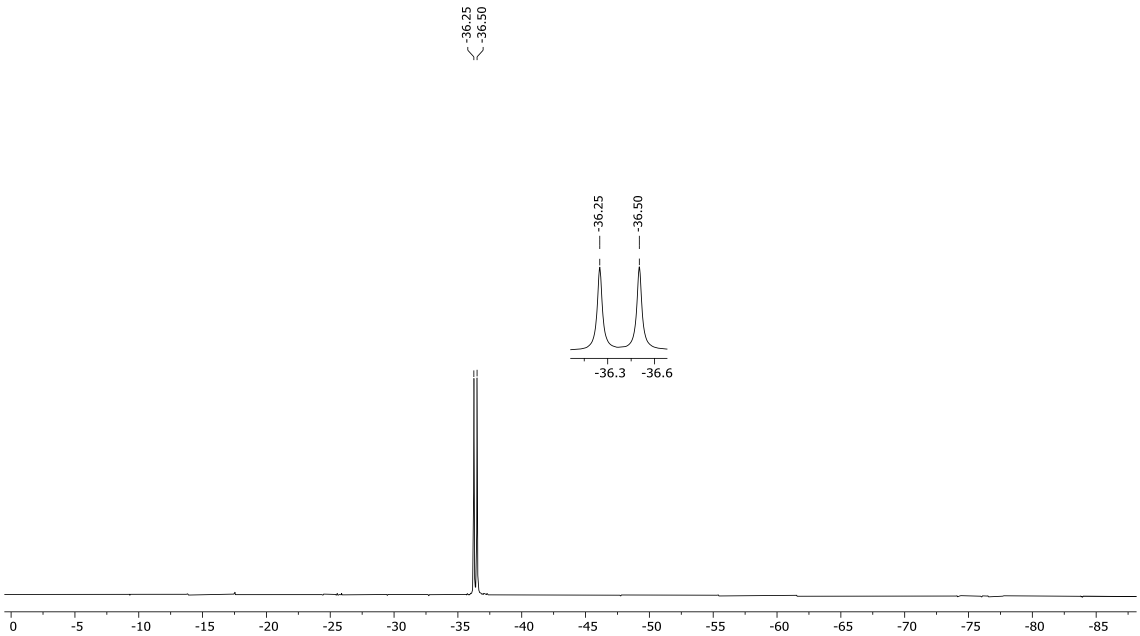


**Figure S24**. $^{19}$F NMR spectrum (282.4 MHz, $CDCl_3$) of **6a**.

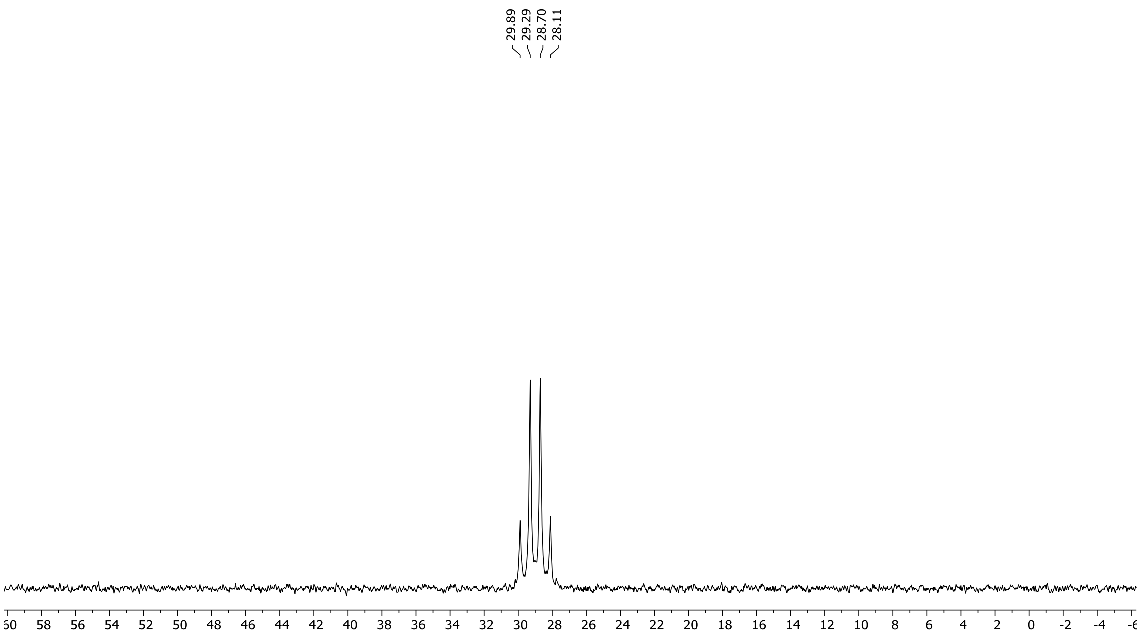


**Figure S25**. $^{31}$P{$^{1}$H} NMR spectrum (121.5 MHz, $CDCl_3$) of **6a**.

***SP-4-3*-[Au($CF_3$)(Me)($OCOCF_3$)($PPh_3$)] (7a) and *SP-4-4*-[Au($CF_3$)(Me)($OCOCF_3$)($PPh_3$)] (7a')**. Trifluoroacetic acid (17 µL, 0.22 mmol) was added to a solution of **3a** (120 mg, 0.215 mmol) in $CH_2Cl_2$ (5 mL) at room temperature. The mixture was stirred for 30 min and evaporated to dryness under vacuum. The residue was suspended in *n*-pentane (2 mL) and the suspension was filtered. The colorless solid was washed with *n*-pentane (2 × 2 mL) and air dried to give **7a**. Yield: 126 mg, 89.3 %. M.p. 147–149 ºC. Anal. Calcd for $C_{22}H_{18}AuO_2F_6P$: C, 40.26; H, 2.76. Found: C, 40.26; H, 2.77. IR ($cm^{-1}$): 1186, 1144, 1114, 1100, 1074, 1047, 998 ν(C–F).

$^1H$ NMR (300.1 MHz, $CDCl_3$): δ 7.64–7.49 (m, 15H, Ph), 1.33 (dd, $^3J_{PH}$ = 5.9 Hz, $^4J_{FH}$ = 0.5 Hz, 3H, Me). $^{13}C\{^1H\}$ NMR (150.9 MHz, $CDCl_3$): δ 160.8 (q, $^2J_{FC}$ = 37.0 Hz, $F_3C$-*C*OO), 139.0 (qd, $^1J_{FC}$ = 364.5 Hz, $^2J_{PC}$ = 243.0 Hz, $F_3C$-Au-P), 134.4 (d, $J_{PC}$ = 11.1 Hz, *o*-Ph), 132.8 (s, *p*-Ph), 129.6 (d, $J_{PC}$ = 11.7 Hz, *m*-Ph), 124.4 (d, $J_{PC}$ = 55.8 Hz, *i*-Ph), 117.9 (q, $^1J_{FC}$ = 292.5 Hz, $F_3$*C*-COO), 8.5 (dq, $J_{PC}$ = 3.8, $J_{FC}$ = 5.4 Hz,Me). $^{19}F$ NMR (282.4 MHz, $CDCl_3$): δ -36.3 (d, $^3J_{PF}$ = 71.4 Hz, $AuCF_3$), -75.0 (s, $OCOCF_3$). $^{31}P\{^1H\}$ NMR (121.5 MHz, $CDCl_3$): δ 29.4 (q, $^3J_{PF}$ = 71.4 Hz).

Partial isomerization of **7a** to **7a'** was observed when solutions of **7a** were left at room temperature or heated. The amount of **7a'** increased up to a maximum value of 20 %. Representative NMR data of **7a'** are given in Table S2.

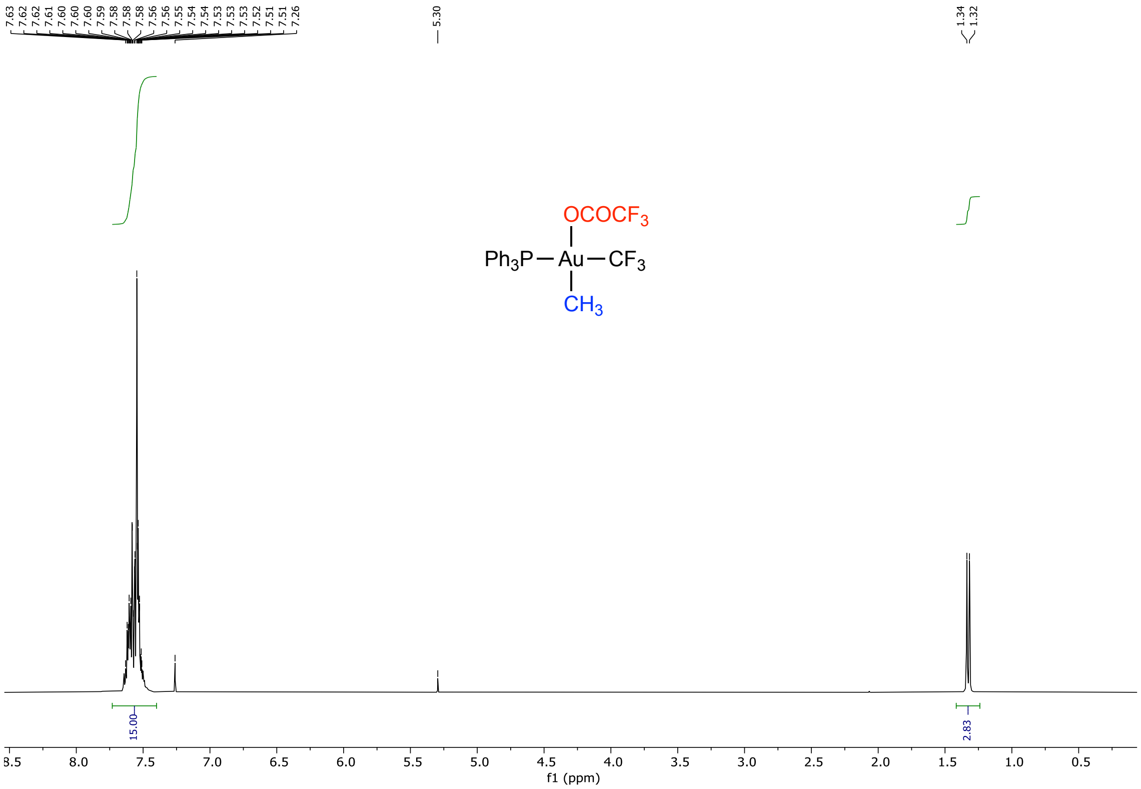


**Figure S26**. $^1H$ NMR spectrum (300.1 MHz, $CDCl_3$) of **7a**.

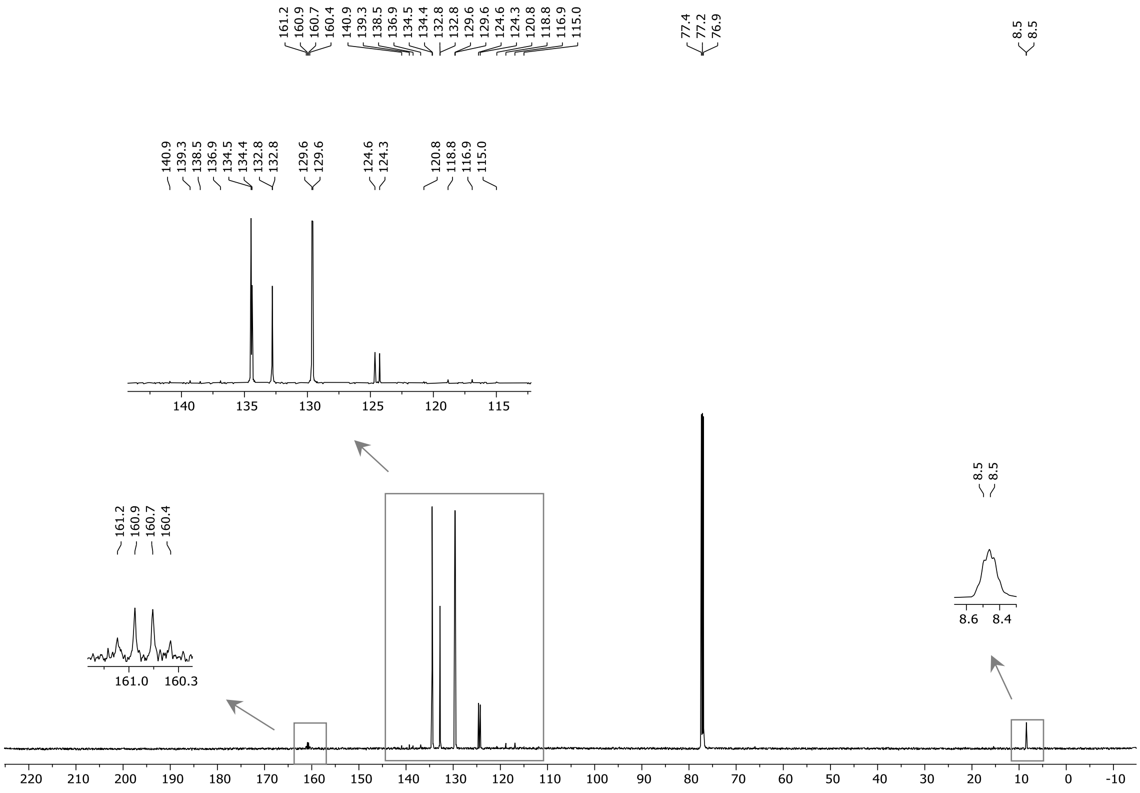

**Figure S27**. $^{13}C\{^{1}H\}$ NMR spectrum (150.9 MHz, $CDCl_3$) of **7a**.

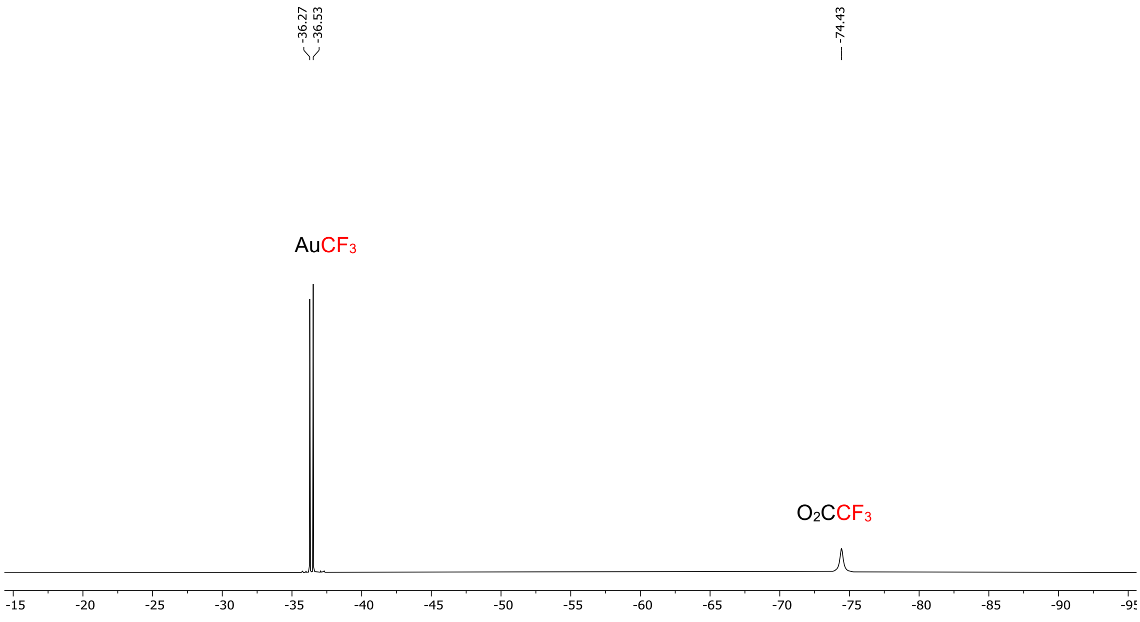


**Figure S28**. $^{19}F$ NMR spectrum (282.4 MHz, $CDCl_3$) of **7a**.

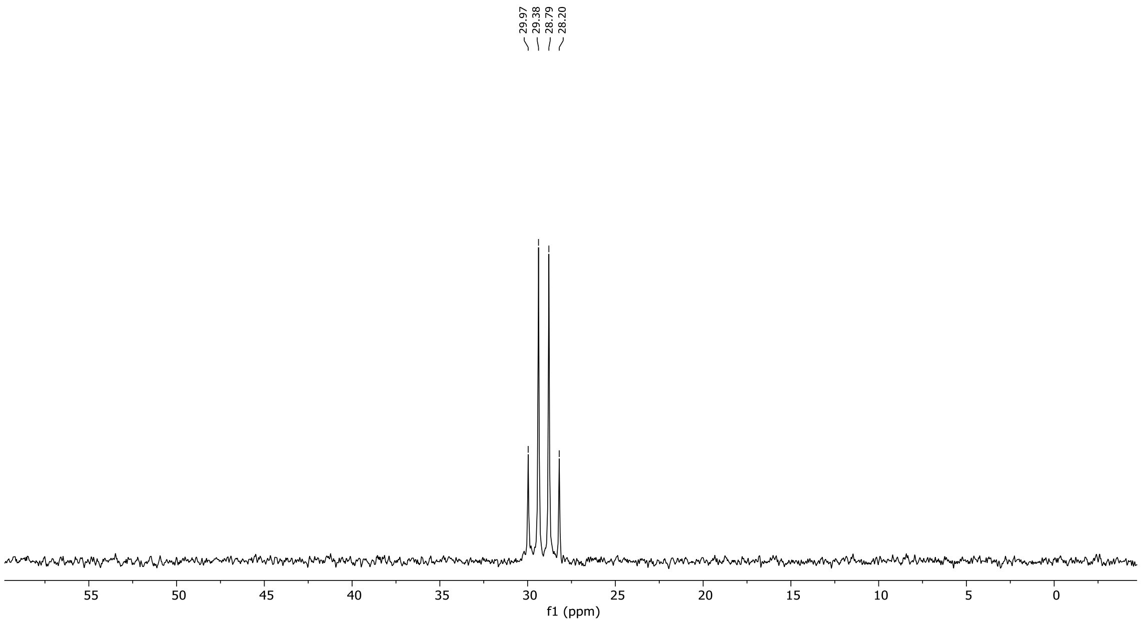

**Figure S29**. $^{31}P\{^1H\}$ NMR spectrum (121.5 MHz, $CDCl_3$) of **7a**.

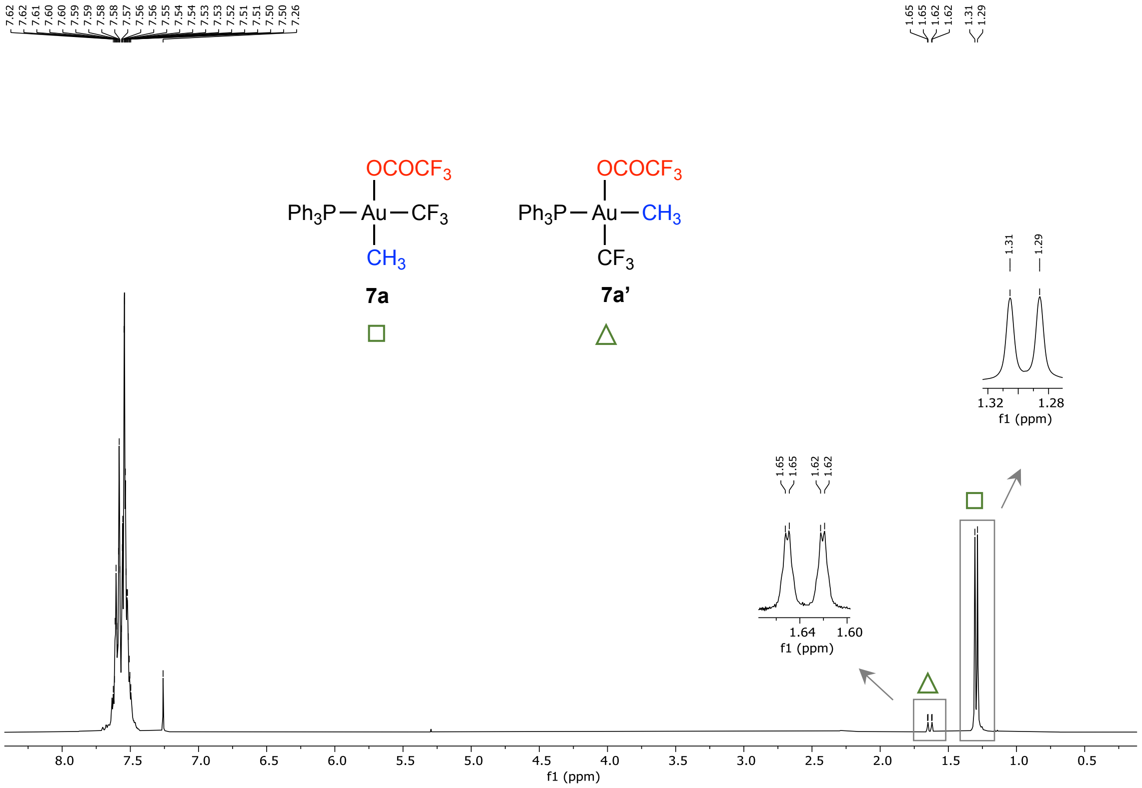

**Figure S30**. $^1H$ NMR spectrum (300.1 MHz, $CDCl_3$) of a mixture of **7a** and **7a'**.

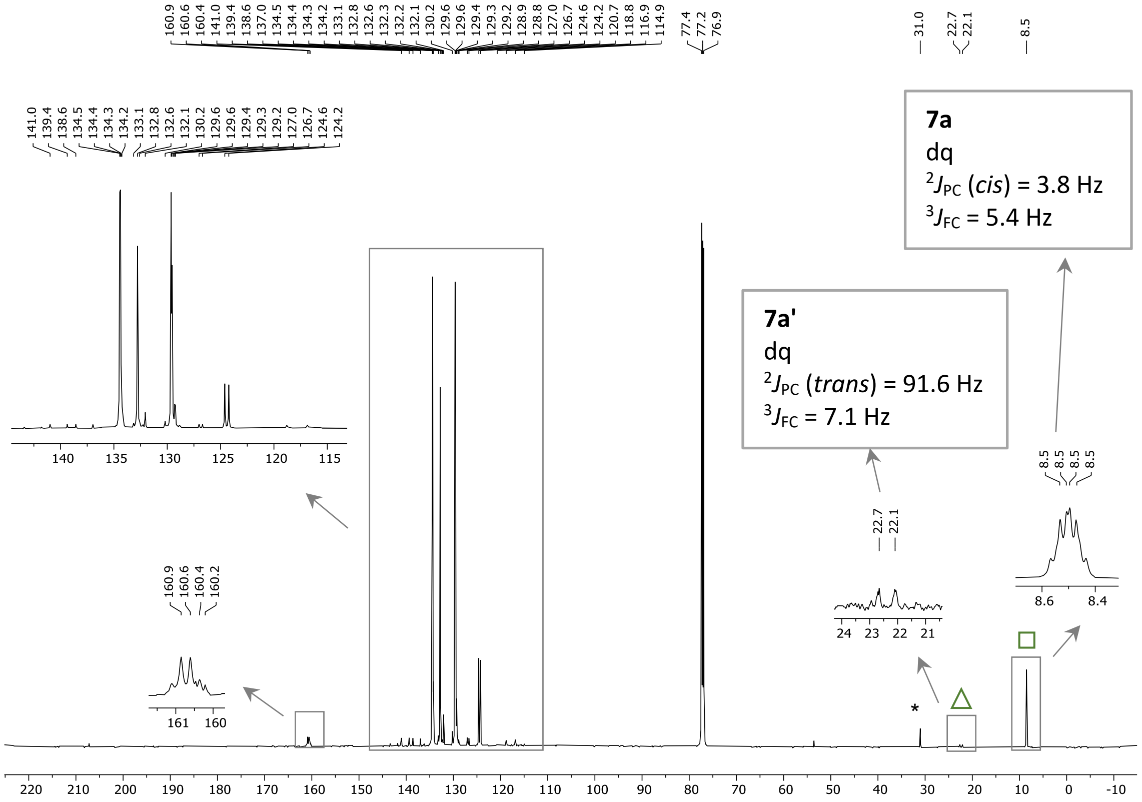


**Figure S31**. $^{13}C\{^1H\}$ NMR spectrum (150.9 MHz, $CDCl_3$) of a mixture of **7a** and **7a'**. The signal marked with an asterisk corresponds to $(CH_3)_2CO$.

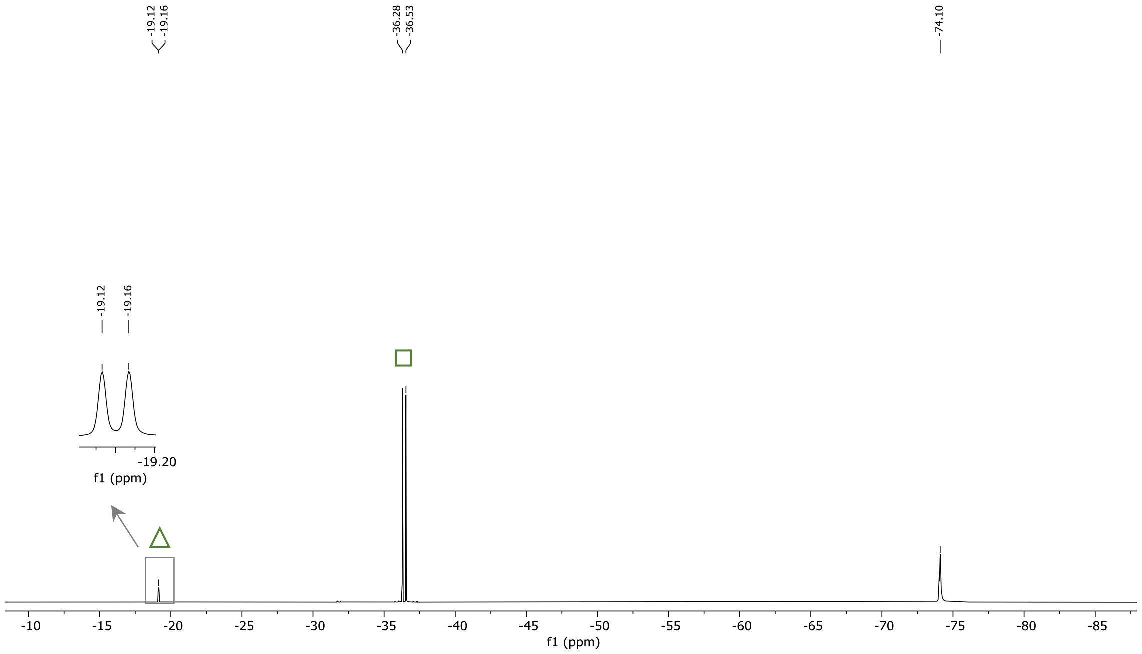


**Figure S32**. $^{19}F$ NMR spectrum (282.4 MHz, $CDCl_3$) of a mixture of **7a** and **7a'**.

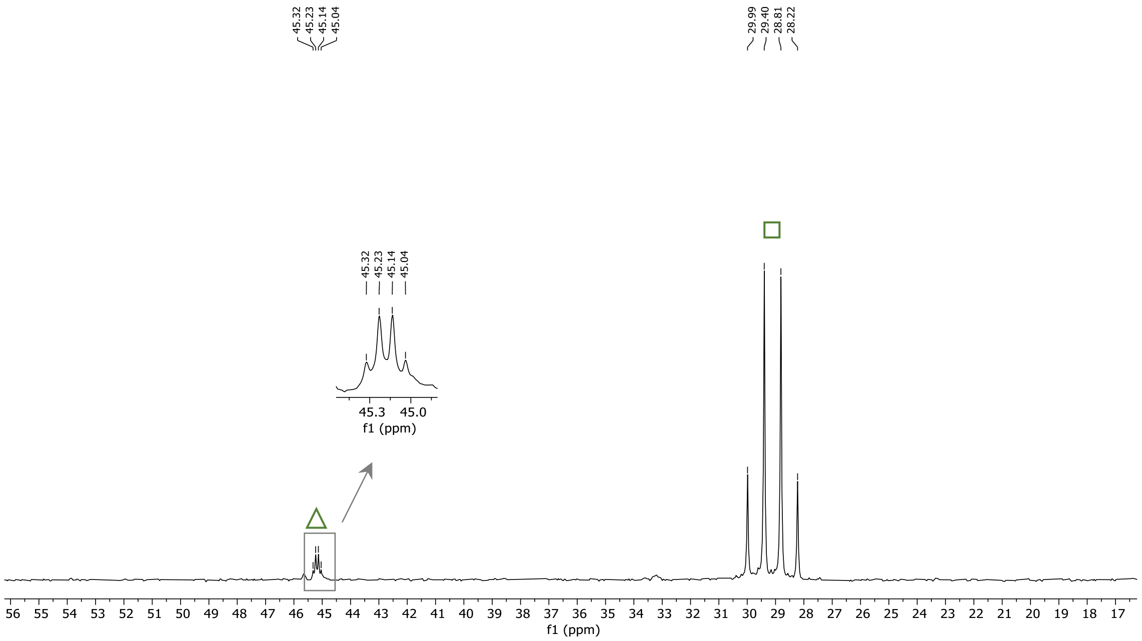


**Figure S33**. $^{31}P\{^1H\}$ NMR spectrum (121.5 MHz, $CDCl_3$) of a mixture of **7a** and **7a'**.

***SP-4-4*-[Au($CF_3$)(Me)Br($PPh_3$)] (8a) and *SP-4-3*-[Au($CF_3$)(Me)Br($PPh_3$)] (8a')**. 48 % Hydrobromic acid (30.0 µL, 0.27 mmol of HBr) was added to a solution of **3a** (101 mg, 0.181 mmol) in $CH_2Cl_2$ (5 mL) at room temperature. The mixture was stirred for 30 min and evaporated to dryness under vacuum. The crude was chromatographed on a silica gel column using $CH_2Cl_2$/*n*-hexane (2:1) as eluent. The collected pale-orange fraction (Rf = 0.36) was evaporated to dryness and the residue was washed with *n*-pentane (3 × 2 mL), to give a pale-orange solid, which was dried under vacuum. Yield: 83 mg, 73 %. The isolated solid was a 96:4 mixture of isomers **8a** and **8a'**. The proportion of **8a'** increased up to a maximum value of 18 % when solutions of **8a** were left at room temperature or heated. M.p. 150–152 ºC. Anal. Calcd for $C_{20}H_{18}AuBrF_3P$: C, 38.55; H, 2.91. Found: C, 38.56; H, 2.81. IR ($cm^{-1}$): 1225, 1104, 1063, 1036, 997 ν(C–F). NMR data of **8a**: $^1H$ NMR (300.1 MHz, $CDCl_3$): δ 7.65-7.48 (m, 15H, Ph), 1.28 (dd, $^3J_{PH}$ = 5.9 Hz, $^4J_{FH}$ = 0.7 Hz, Me). $^{13}C\{^1H\}$ NMR (75.5 MHz, $CDCl_3$): δ 137.8 (qd, $^1J_{FC}$ = 357.7 Hz, $^2J_{PC}$ = 257.2 Hz, $CF_3$), 134.8 (d, $J_{PC}$ = 10.7 Hz, *o*-Ph), 132.4 (d, $J_{PC}$ = 2.8 Hz, *p*-Ph), 129.2 (d, $J_{PC}$ = 11.8 Hz, *m*-Ph), 125.9 (d, $J_{PC}$ = 57.3 Hz, *i*-Ph), 20.3 (m, Me).$^{19}F$ NMR (282.4 MHz, $CDCl_3$): δ -28.9 (d, $^3J_{PF}$ = 70.1 Hz). $^{31}P\{^1H\}$ NMR (121.5 MHz, $CDCl_3$): δ 26.7 (q, $^3J_{PF}$ = 70.1 Hz). The NMR data of **8a'** are given in Table S2.

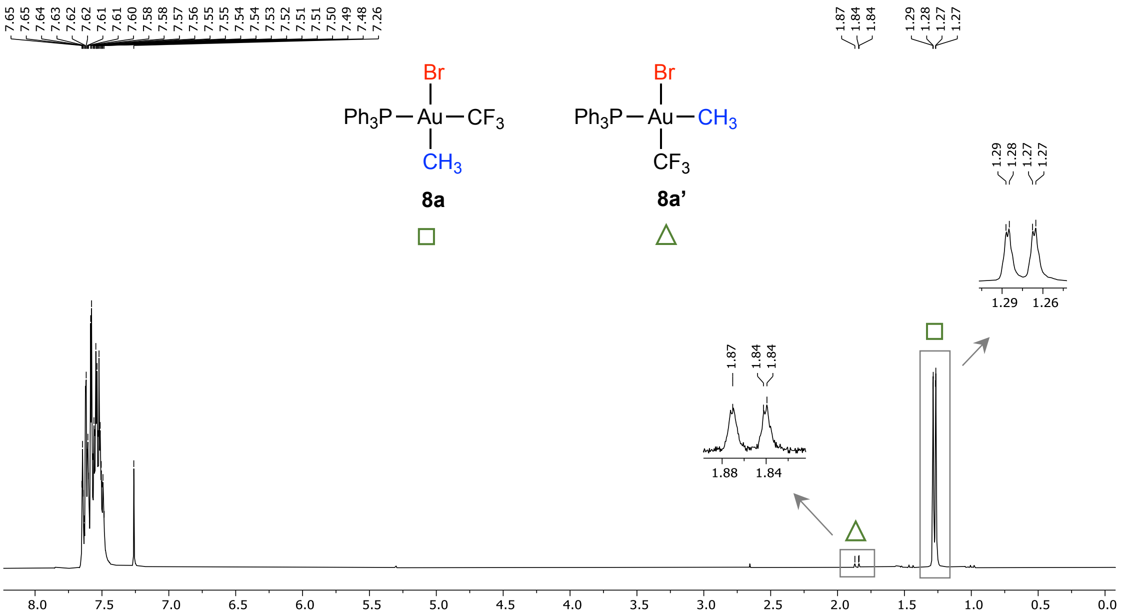


**Figure S34**. $^{1}H$ NMR spectrum (300.1 MHz, $CDCl_3$) of **8a** and **8a'**.

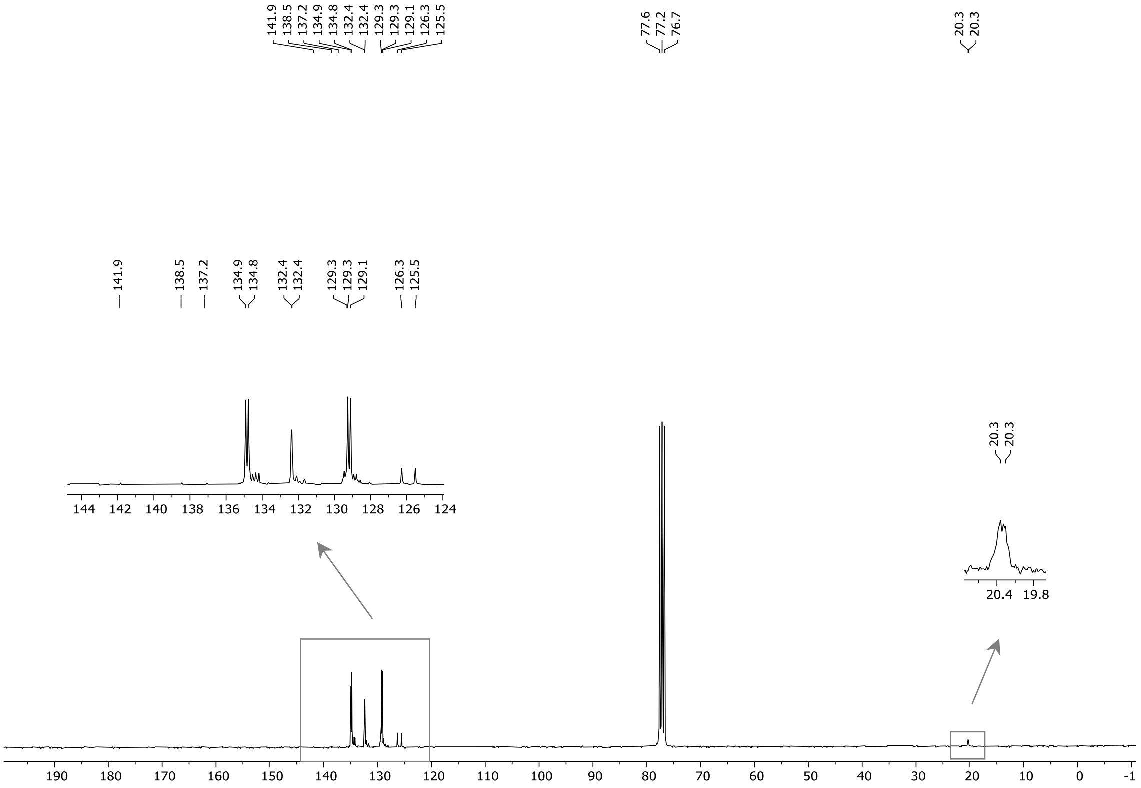


**Figure S35**. $^{13}C\{^{1}H\}$ NMR spectrum (75.5 MHz, $CDCl_3$) of **8a** and **8a'**.

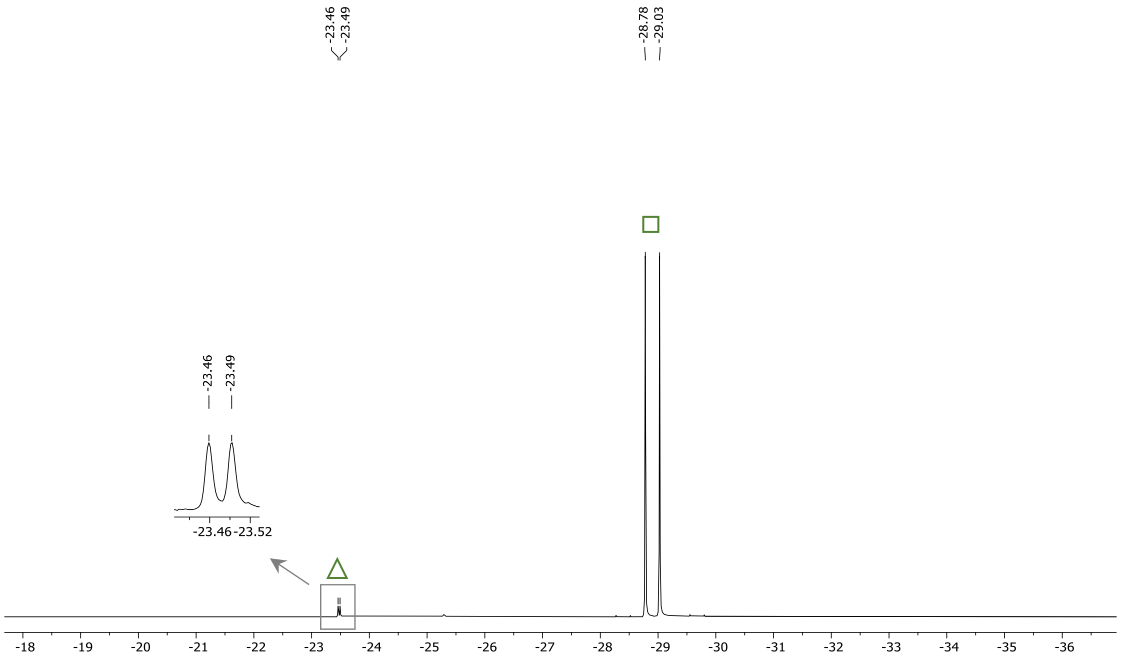


**Figure S36**. $^{19}F$ NMR spectrum (282.4 MHz, $CDCl_3$) of **8a** and **8a'**.

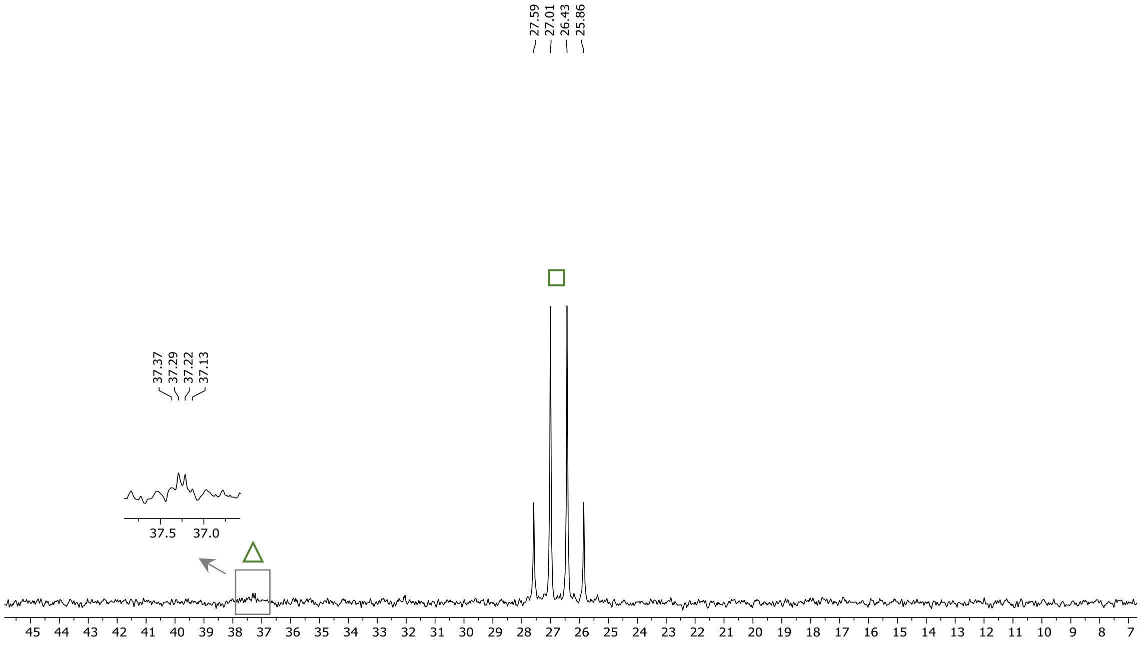


**Figure S37**. $^{31}P\{^1H\}$ NMR spectrum (121.5 MHz, $CDCl_3$) of **8a** and **8a'**.

***SP-4-4*-[Au(CF$_3$)(Me)Br(PCy$_3$)] (8b)**. 48 % Hydrobromic acid (35 µL, 0.31 mmol of HBr) was added to a solution of **3b** (130 mg, 0.226 mmol) in $CH_2Cl_2$ (5 mL) at room temperature. The mixture was stirred for 30 min and evaporated to dryness under vacuum. The crude was chromatographed on a silica gel column using $CH_2Cl_2$/*n*-hexane (1:2) as eluent. The collected colourless fraction (Rf = 0.13) was evaporated to dryness and the residue was washed with *n*-pentane (3 × 2 mL), to give a colorless solid, which was dried under vacuum. Yield: 93 mg, 64 %. M.p. 194–196 ºC. Anal. Calcd for $C_{20}H_{36}AuBrF_3P$: C, 37.46; H, 5.66. Found: C, 37.18; H, 5.81. IR (cm$^{-1}$): 1220,

1176, 1029, 1004 ν(C–F). $^{1}$H NMR (300.1 MHz, $CDCl_3$): δ 2.68 (m, 3 H, PCH), 1.95-1.30 (several m, 30 H, Cy), 1.44 (dd, $^{3}J_{PH}$ = 4.2 Hz, $^{4}J_{FH}$ = 0.7 Hz, 3H, Me). $^{13}$C{$^{1}$H} NMR (150.9 MHz, $CDCl_3$): δ 141.5 (qd, $^{1}J_{FC}$ = 364.5 Hz, $^{2}J_{PC}$ = 232.8 Hz, $CF_3$), 32.5 (d, $^{1}J_{PC}$ = 24.5 Hz, Cy), 29.7 (d, $^{3}J_{PC}$ = 2.4 Hz, Cy), 27.5 (d, $^{2}J_{PC}$ = 11.1 Hz, Cy), 26.2 (d, $^{4}J_{PC}$ = 1.4 Hz, Cy), 13.7 (m, Me).$^{19}$F NMR (282.4 MHz, $CDCl_3$): δ -31.0 (d, $^{3}J_{PF}$ = 65.6 Hz). $^{31}$P{$^{1}$H} NMR (121.5 MHz, $CDCl_3$): δ 30.2 (q, $^{3}J_{PF}$ = 65.6 Hz).

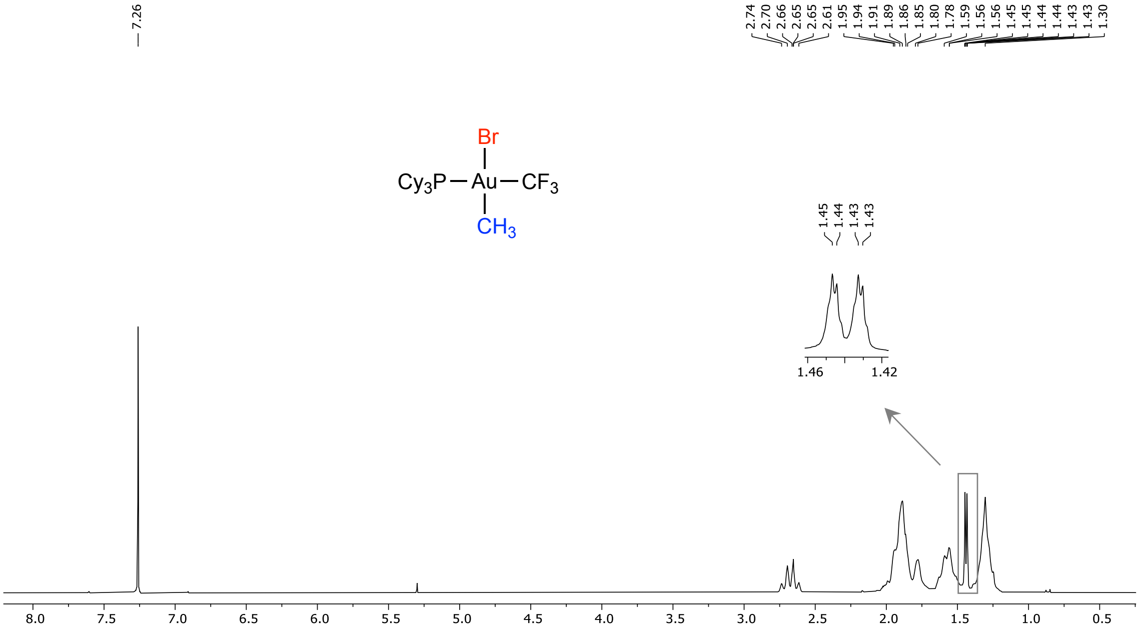


**Figure S38**. $^{1}$H NMR spectrum (300.1 MHz, $CDCl_3$) of **8b**.

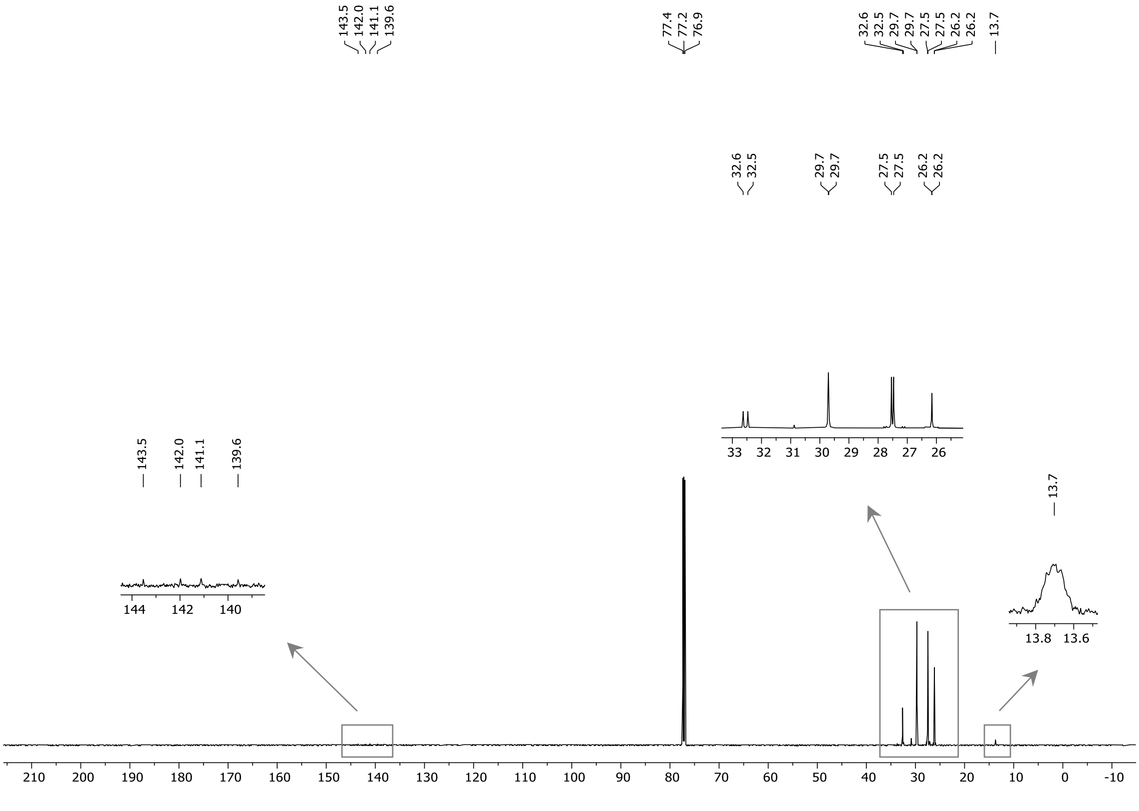

**Figure S39**. $^{13}$C{$^{1}$H} NMR spectrum (150.9 MHz, $CDCl_3$) of **8b**.

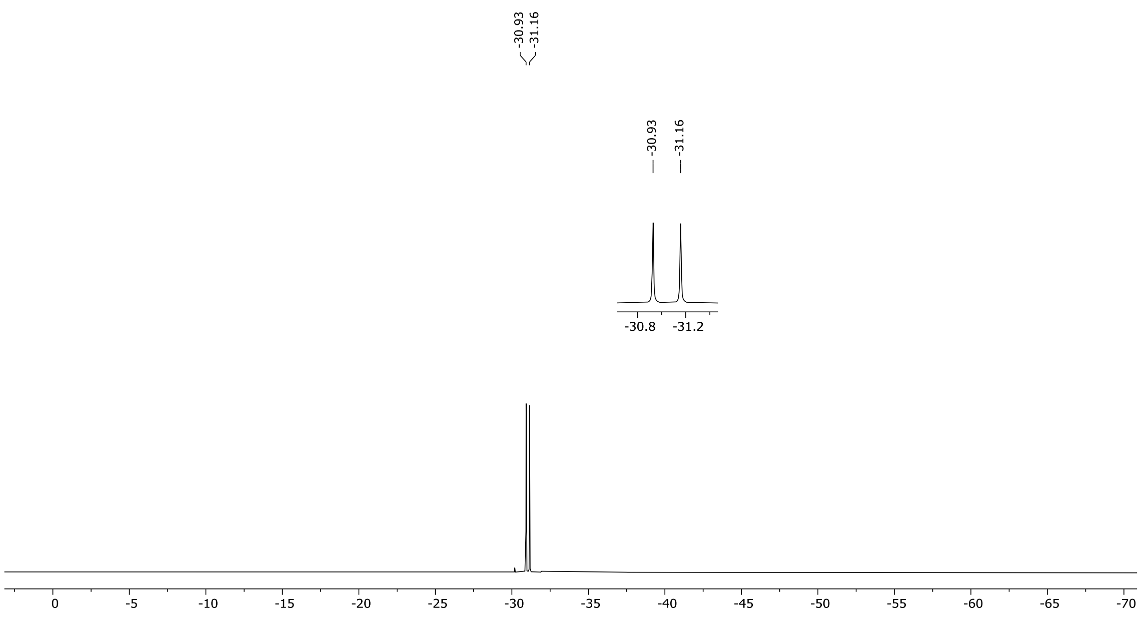


**Figure S40**. $^{19}F$ NMR spectrum (282.4 MHz, $CDCl_3$) of **8b**.

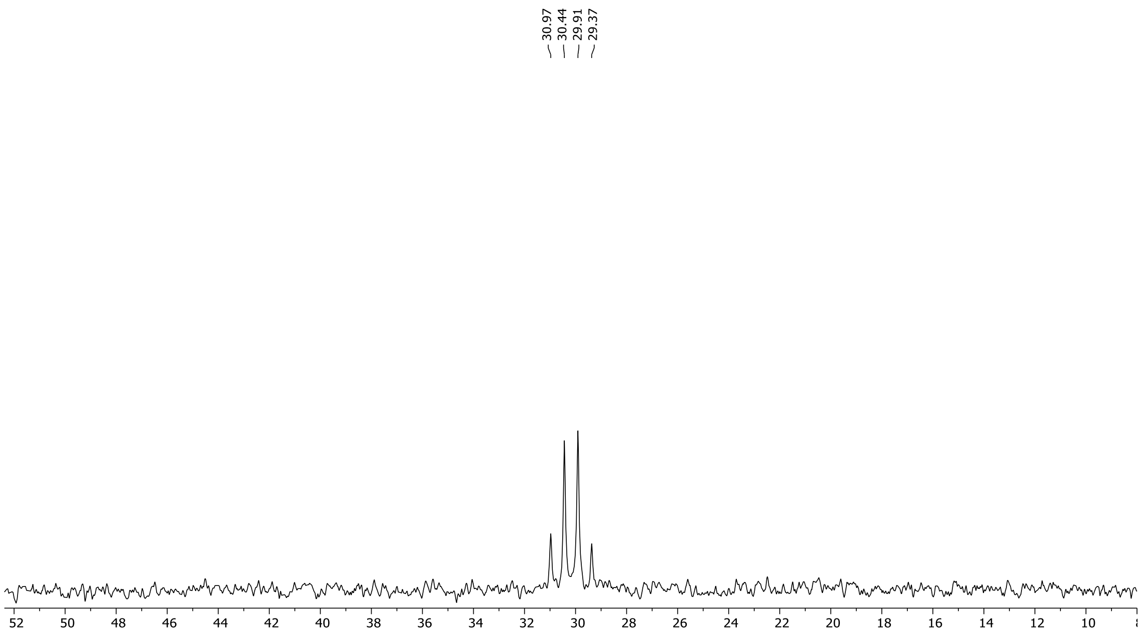


**Figure S41**. $^{31}P\{^1H\}$ NMR spectrum (121.5 MHz, $CDCl_3$) of **8b**.

***SP-4-4*-[Au(CF$_3$)(Me)Cl(PPh$_3$)] (9a) and *SP-4-3*-[Au(CF$_3$)(Me)Cl(PPh$_3$)] (9a')**. 37% Hydrochloric acid (18 µL, 0.22 mmol of HCl) was added to a solution of **3a** (80 mg, 0.14 mmol) in $CH_2Cl_2$ (5 mL) at room temperature. The mixture was stirred for 30 min and evaporated to dryness under vacuum. The crude was chromatographed on a silica gel column using $CH_2Cl_2$/*n*-hexane (2:1) as eluent. The collected colorless fraction (Rf = 0.16) was evaporated to dryness and the residue was washed with *n*-pentane (3 × 2 mL) to give **9a** as a colorless solid, which was dried under vacuum. Yield: 58 mg, 70 %. M.p. 151–153 ºC. Anal. Calcd for $C_{20}H_{18}AuClF_3P$: C, 41.51; H, 3.14. Found: C, 41.46; H, 2.95. IR (cm$^{-1}$): 1232, 1104, 1063, 1036, 997 ν(C–F). $^1H$ NMR (300.1 MHz, $CDCl_3$):

δ 7.65-7.49 (m, 15H, Ph), 1.15 (dd, $^3J_{PH}$ = 6.0 Hz, $^4J_{FH}$ = 0.6 Hz, Me). $^{13}C\{^1H\}$ NMR (75.5 MHz, $CD_2Cl_2$): δ 139.1 (qd, $^1J_{FC}$ = 360.2 Hz, $^2J_{PC}$ = 256.9 Hz, $CF_3$), 135.1 (d, $J_{PC}$ = 11.8 Hz, *o*-Ph), 132.7 (d, $J_{PC}$ = 2.8 Hz, *p*-Ph), 129.5 (d, $J_{PC}$ = 11.0 Hz, *m*-Ph), 125.7 (d, $J_{PC}$ = 56.6 Hz, *i*-Ph), 17.1 (m, Me).$^{19}F$ NMR (282.4 MHz, $CDCl_3$): δ -31.8 (d, $^3J_{PF}$ = 71.1 Hz). $^{31}P\{^1H\}$ NMR (121.5 MHz, $CDCl_3$): δ 27.7 (q, $^3J_{PF}$ = 71.1 Hz).

Partial isomerization of **9a** to **9a'** was observed when solutions of **9a** were left at room or higher temperature. The amount of **9a'** increased up to a maximum value of 16 %. Representative NMR data of **9a'** are given in Table S2.

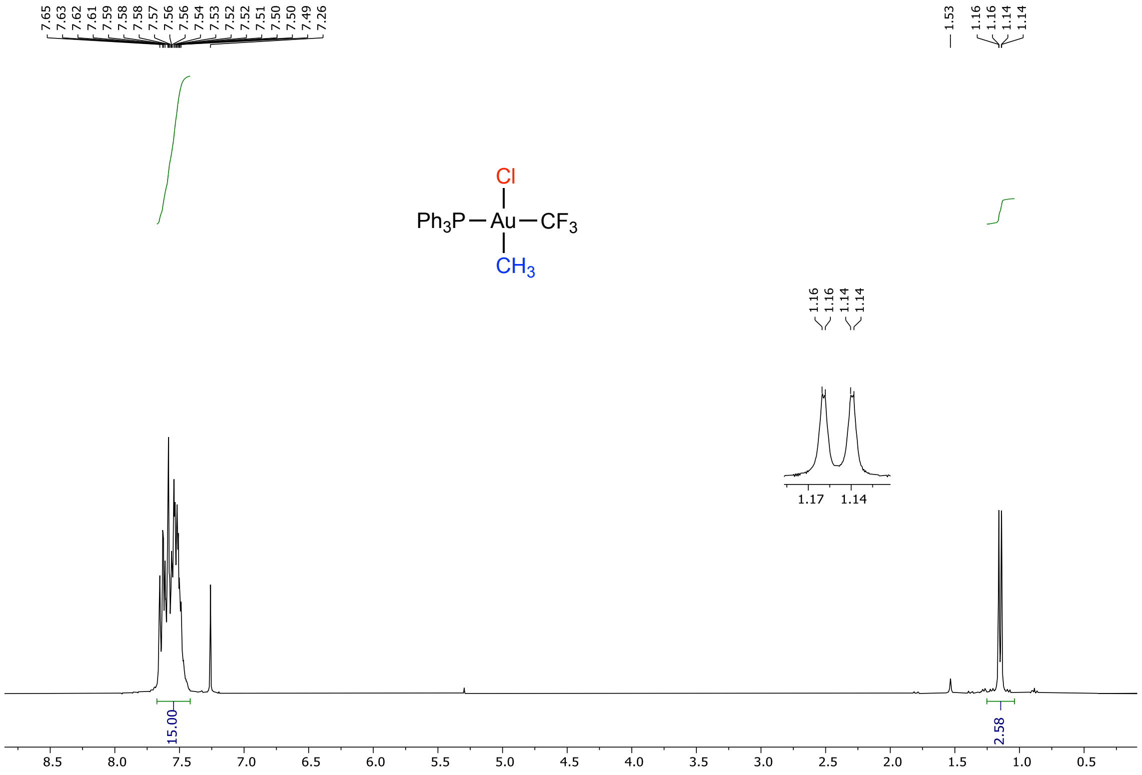


**Figure S42**. $^1H$ NMR spectrum (300.1 MHz, $CDCl_3$) of **9a**.

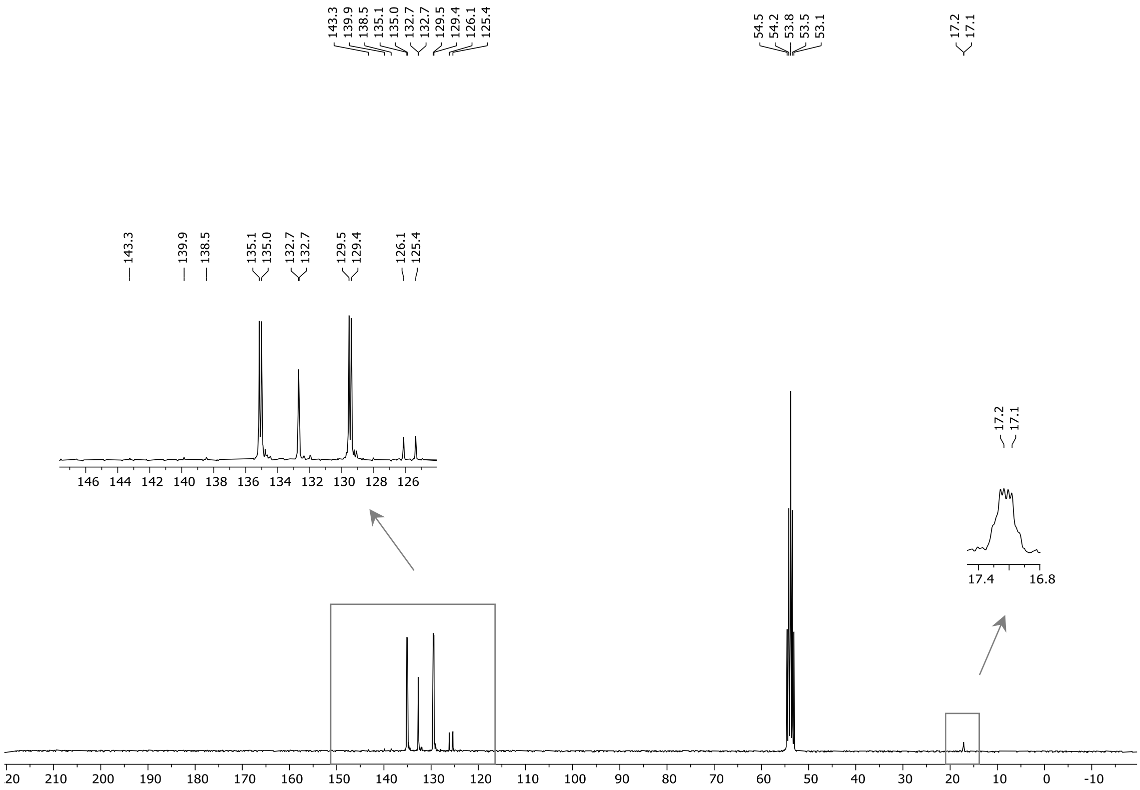


**Figure S43**. $^{13}C\{^1H\}$ NMR spectrum (75.5 MHz, $CD_2Cl_2$) of **9a**.

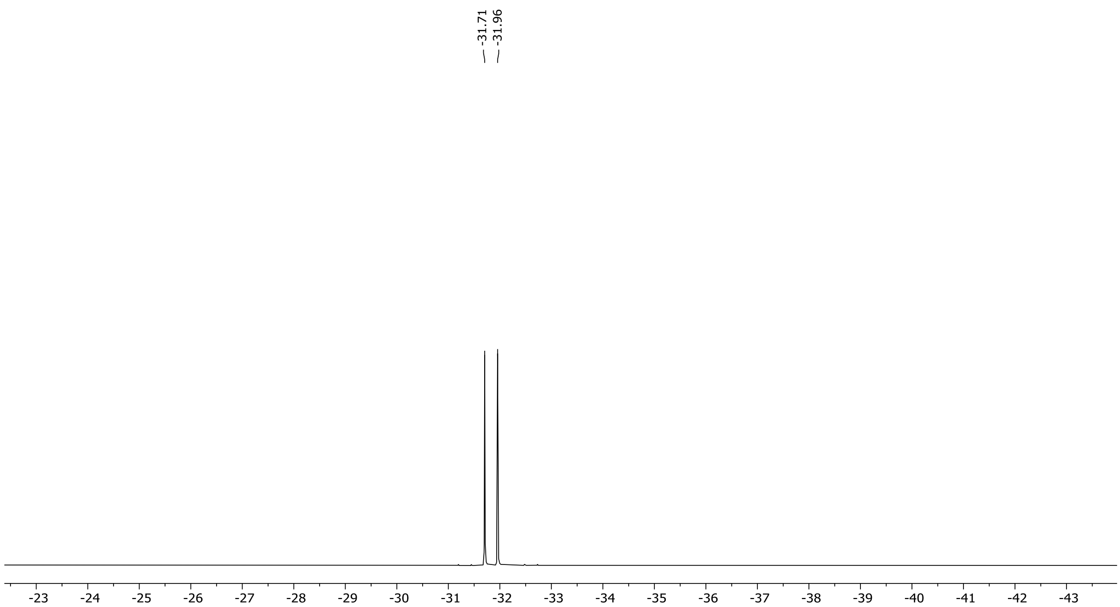


**Figure S44**. $^{19}F$ NMR spectrum (282.4 MHz, $CDCl_3$) of **9a**.

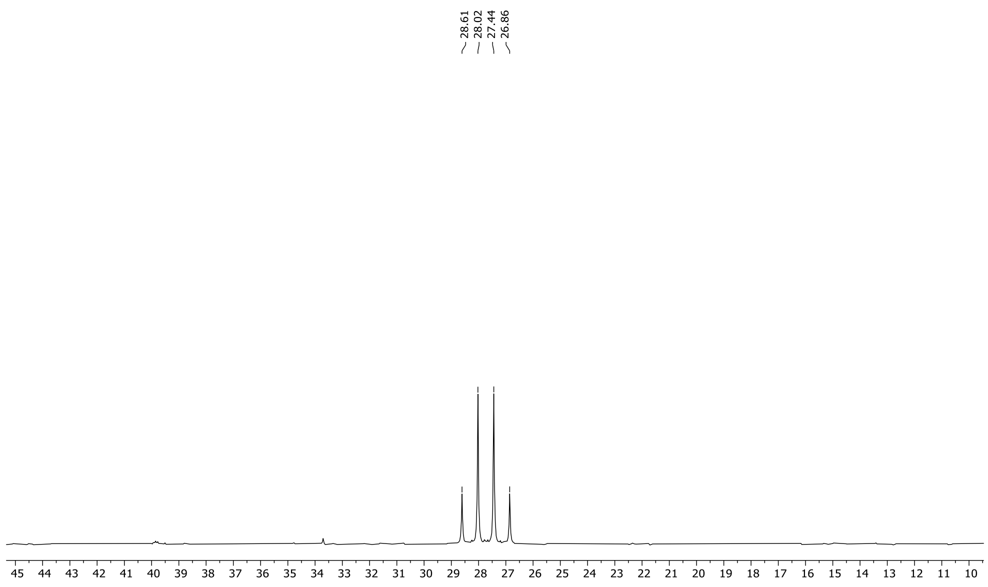


**Figure S45**. $^{31}P\{^1H\}$ NMR spectrum (121.5 MHz, $CDCl_3$) of **9a**.

***SP-4-3*-[Au($CF_3$)(Me)F($PPh_3$)] (10a) and *SP-4-4*-[Au($CF_3$)(Me)F($PPh_3$)] (10a')**. AgF (20 mg, 0.16 mmol) was added to a solution of **8a** and **8a'** (33 mg, 0.053 mmol; 96 % of **4a**) in $CDCl_3$ (0.5 mL) in a FEP tube with a PTFE stopper protected from light. The mixture was sonicated at room temperature for 2 h. The NMR spectra showed formation of a mixture of **10a** (83 %) and **10a'** (17 %). NMR data of **10a** and **10a'**: $^1H$ NMR (300.1 MHz, $CDCl_3$): δ 7.64-7.51 (m, 15H, Ph), 1.75 (tq, $^3J_{PH}$ = $^3J_{FH}$ = 9.4 Hz, $^4J_{FH}$ = 1.1 Hz, Me of **10a'**), 1.02 (d, $^3J_{PH}$ = 6.0 Hz, Me of **10a**). $^{19}F$ NMR (282.4 MHz, $CDCl_3$): δ -19.6 (dd, $^3J_{FF}$ = 51.4 Hz, $^3J_{PF}$ = 9.3 Hz, $CF_3$ of **10a'**), -37.7 (dd, $^3J_{PF}$ = 72.3, $^3J_{FF}$ = 12.7 Hz, $CF_3$ of **10a**), -223.8 (dq, $^2J_{PF}$ = 25.4, $^3J_{FF}$ = 12.7 Hz, AuF of **10a**), -246.8 (dqq, $^3J_{FF}$ = 51.4 Hz, $^2J_{PF}$ = 46.3 Hz, $^3J_{FH}$ = 9.0 Hz, AuF of **10a'**). $^{31}P\{^1H\}$ NMR (121.5 MHz, $CDCl_3$): δ 43.5 (dq, $^2J_{PF}$ = 46.8 Hz, $^3J_{PF}$ = 9.4 Hz, **10a'**), 25.5 (dq, $^3J_{PF}$ = 72.4, $^2J_{PF}$ = 25.3 Hz, **10a'**).

The following spectra were measured at a reaction time of 1 h and therefore still contain a 20 % of unreacted **8a** and **8a'**. These spectra were chosen because they show an excellent resolution and all the coupling patterns were resolved. At a reaction time of 2 h the signals of **8a** and **8a'** have disappeared, but the signals of **10a** and **10a'** appear broadened (see Figures S93–S95).

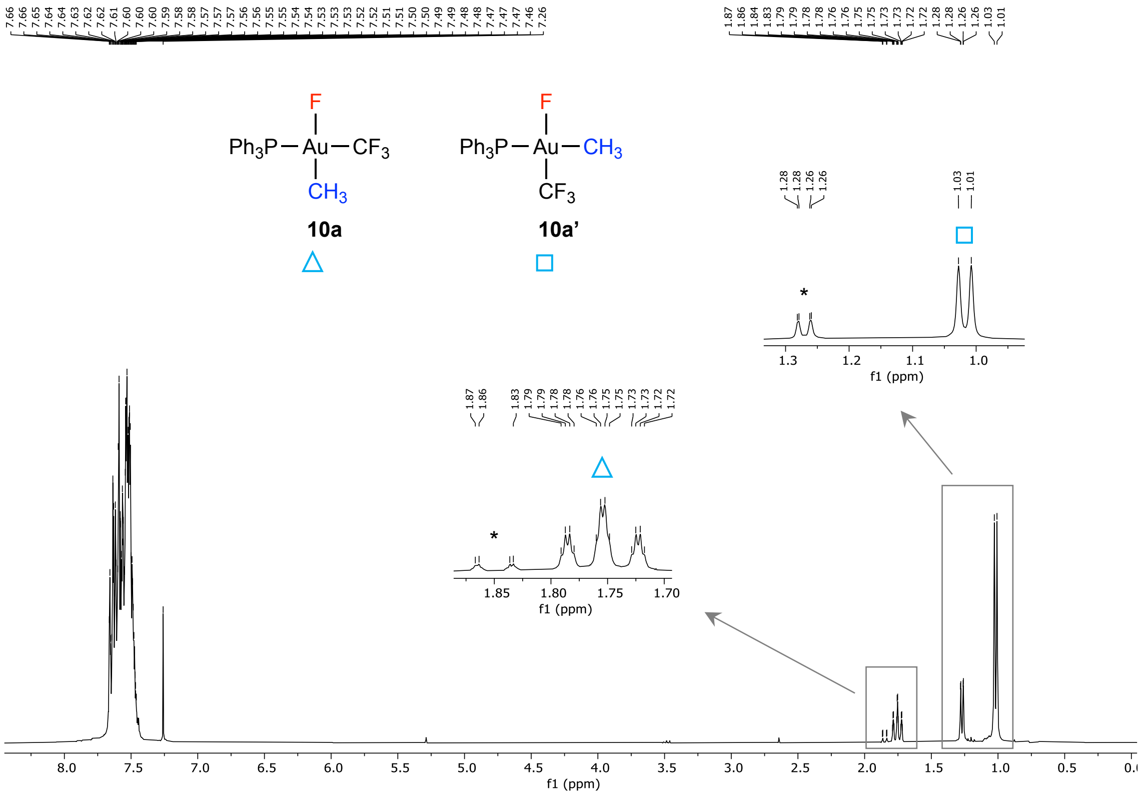

**Figure S46**. $^{1}$H NMR spectrum (300.1 MHz, $CDCl_3$) of in situ generated **10a** and **10a'** (the reaction mixture was sonicated for 1h). The signals marked with asterisks correspond to unreacted **8a** and **8a'**.

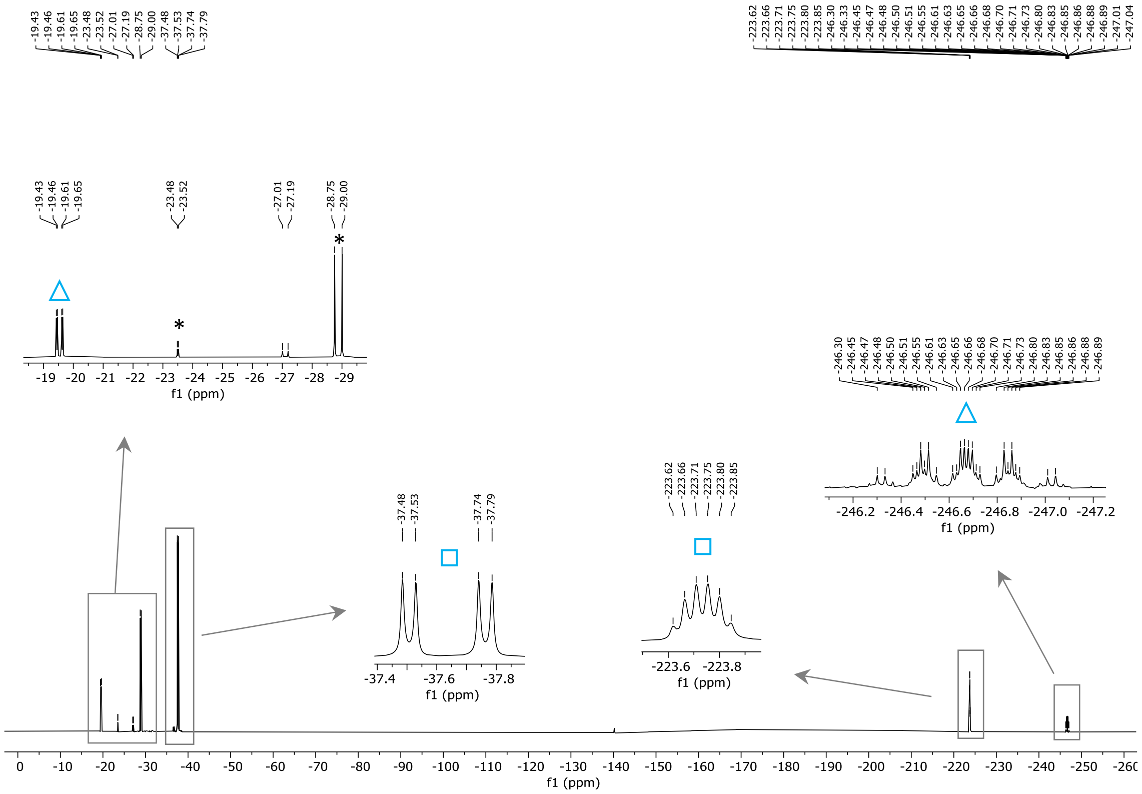

**Figure S47**. $^{19}$F NMR spectrum (121.5 MHz, $CDCl_3$) of in situ generated **10a** and **10a'**. The doublets marked with asterisk correspond to unreacted **8a** and **8a'**.

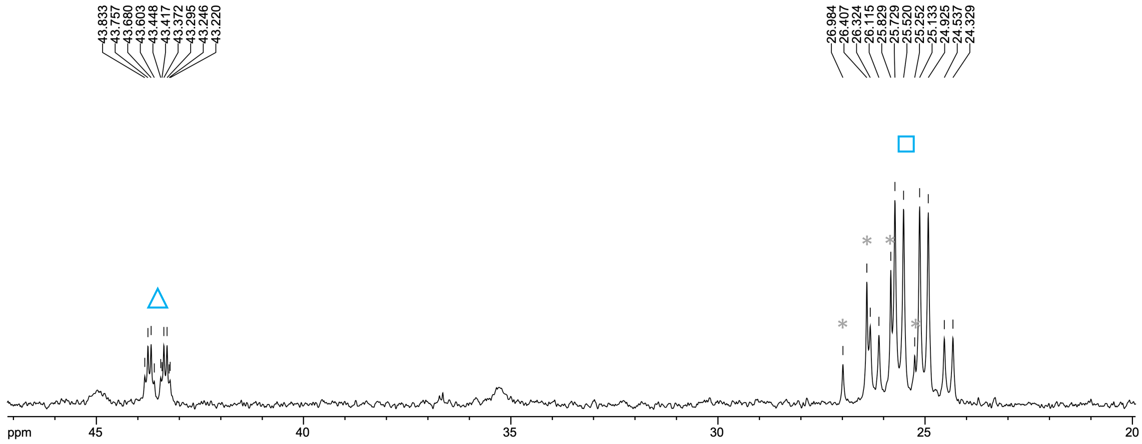


**Figure S48**. $^{31}P\{^{1}H\}$ NMR spectrum (121.5 MHz, $CDCl_3$) of in situ generated **10a** and **10a'**. The quartet labeled with asterisks corresponds to unreacted **8a**.

**Reaction of 3a with HOTf and KI**. HOTf (2.0 µL, 0.02 mmol) was added to a solution of **3a** (12.0 mg, 0.02 mmol) in $CDCl_3$. After 10 min, KI (4.0 mg, 0.02 mmol) was added and the NMR tube was shaken. After 20 min, NMR spectra were measured, showing formation of *SP-4-4*-[Au($CF_3$)(Me)(I)($PPh_3$)] (**11a**) and *SP-4-3*-[Au($CF_3$)(Me)(I)($PPh_3$)] (**11a'**) and K[Au($CF_3$)(Me)$I_2$] (**12**) as main products. Representative NMR data of **11a**, **11a'** and **12** are given in Tables S1 and S2.

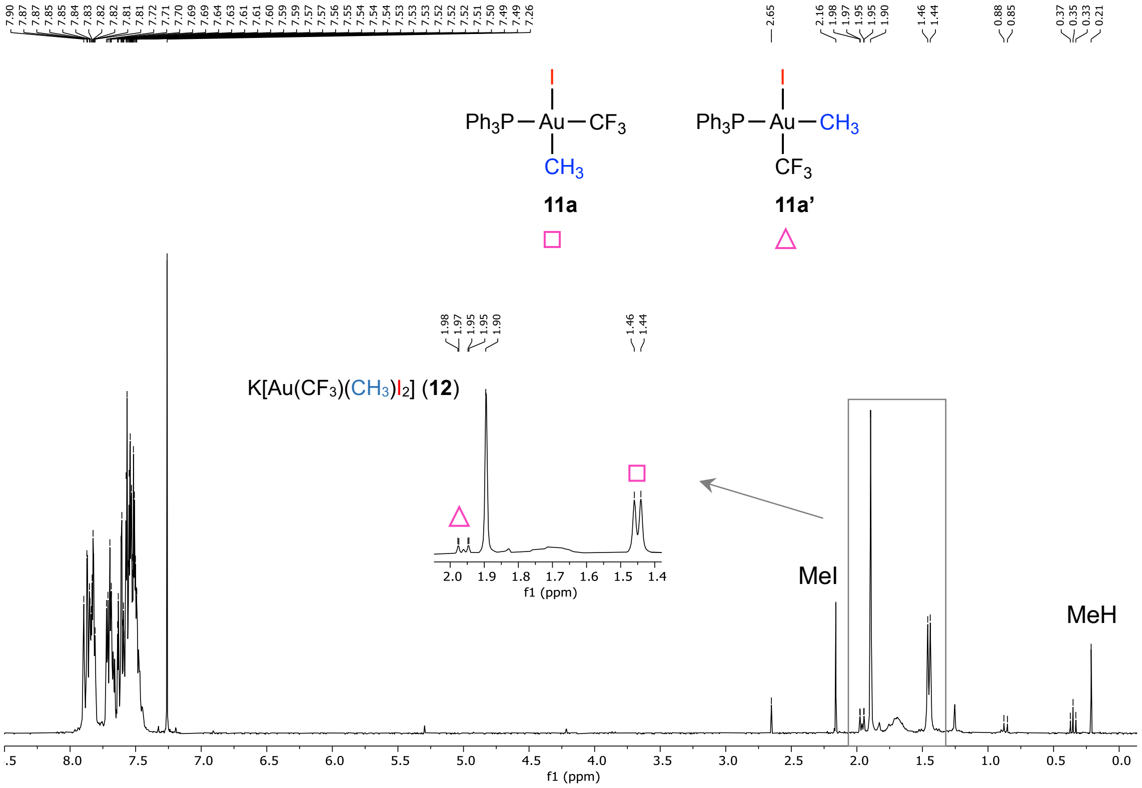


**Figure S49**. $^{1}H$ NMR spectrum (300.1 MHz, $CDCl_3$) of the reaction mixture of **3a** with HOTf and KI.

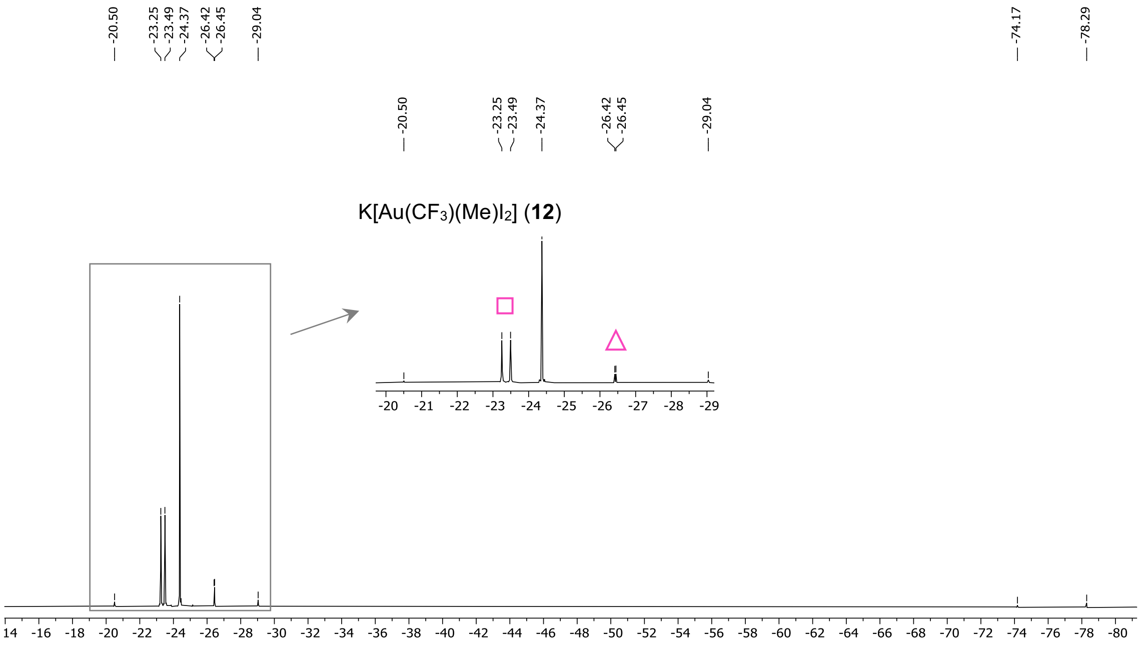


**Figure S50**. $^{19}$F NMR spectrum (282.4 MHz, $CDCl_3$) of the reaction mixture of **3a** with HOTf and KI.

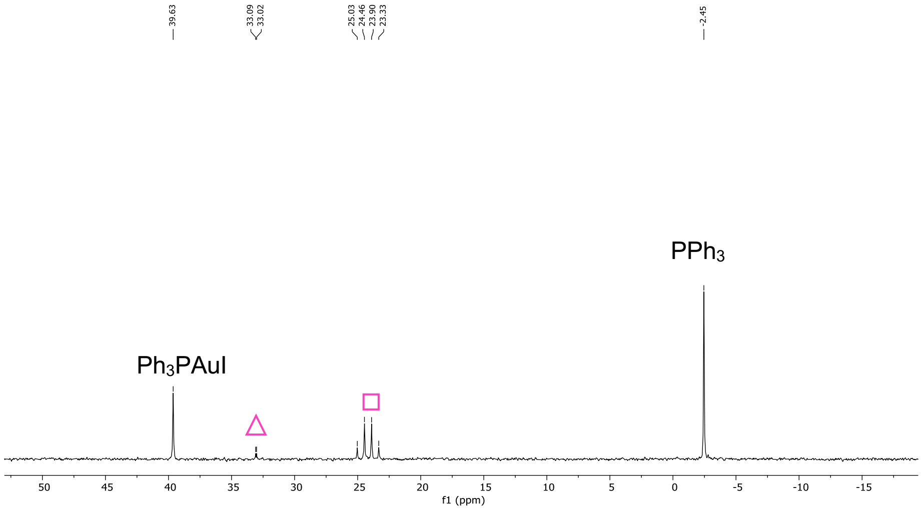


**Figure S51**. $^{31}$P{$^1$H} NMR spectrum (121.5 MHz, $CDCl_3$) of the reaction mixture of **3a** with HOTf and KI.

***cis*-[Au(CF$_3$)(Me)(PPh$_3$)$_2$]$^+$ (TfO)$^-$ (13)**. A solution of **3a** (53 mg, 0.095 mmol) in $CH_2Cl_2$ (5 mL) was treated with HOTf (9 µL, 0.1 mmol) at room temperature with stirring for 30 min. Then, $PPh_3$ (26 mg, 0.1 mmol) was added with stirring for 30 min. The resulting colourless solution was concentrated to ca. 0.5 mL under vacuum, layered with $Et_2O$ (10 mL) and the two phases were allowed to slowly mix at 5 ºC for 16 h. The crystals were separated from the mother liquor, washed with $Et_2O$ (3 × 2 mL) and air dried. Yield: 75 mg, 83 %. M.p. 146–148 ºC. Anal. Calcd for $C_{39}H_{33}AuF_6P_2SO_3$: C, 49.07; H, 3.48; S, 3.36. Found: C, 49.29; H, 3.45; S, 3.47. IR (cm$^{-1}$): 1278, 1254, 1222, 1154, 1091, 1024, 996 ν(C–F). $^1$H NMR (300.1 MHz, $CDCl_3$): δ 7.61-7.11 (m, 30H, Ph), 1.5 (t, $^3J_{PH}$ = 7.0 Hz, Me). $^{13}$C{$^1$H} NMR (100.8 MHz, $CDCl_3$): δ 134.1 (d, $J_{PC}$ = 10.9 Hz, *o*-Ph), δ 134.1 (d, $J_{PC}$ = 11.2 Hz, *o*-Ph), 133.5 (d, $J_{PC}$ = 2.9 Hz, *p*-Ph), 132.7 (d, $J_{PC}$ = 2.9 Hz, *p*-Ph), 132.0 (d, $J_{PC}$ = 11.8 Hz, *m*-Ph), 129.5 (d, $J_{PC}$ = 11.4 Hz, *m*-Ph), 126.8 (d, $J_{PC}$ =

51.2 Hz, *i*-Ph), 124.0 (d, $J_{PC}$ = 58.5 Hz, *i*-Ph), 27.4 (dm, $J_{PC}$ = 80.2 Hz, Me); the $CF_3$ signals were not observed. $^{19}$F NMR (282.4 MHz, $CDCl_3$): δ -25.7 (dd, $^3J_{PFtrans}$ = 67.8 Hz, $^3J_{PFcis}$ = 8.0 Hz, $AuCF_3$), -77.9 (s, $TfO^-$). $^{31}P\{^1H\}$ NMR (121.5 MHz, $CDCl_3$): δ 34.8 (m, $^3J_{PFcis}$ = 8.1 Hz, $^2J_{PP}$ = 17.2 Hz, P cis to $CF_3$), 28.9 (qd, $^3J_{PFtrans}$ = 67.7 Hz, $^2J_{PP}$ = 17.4 Hz, P trans to $CF_3$).

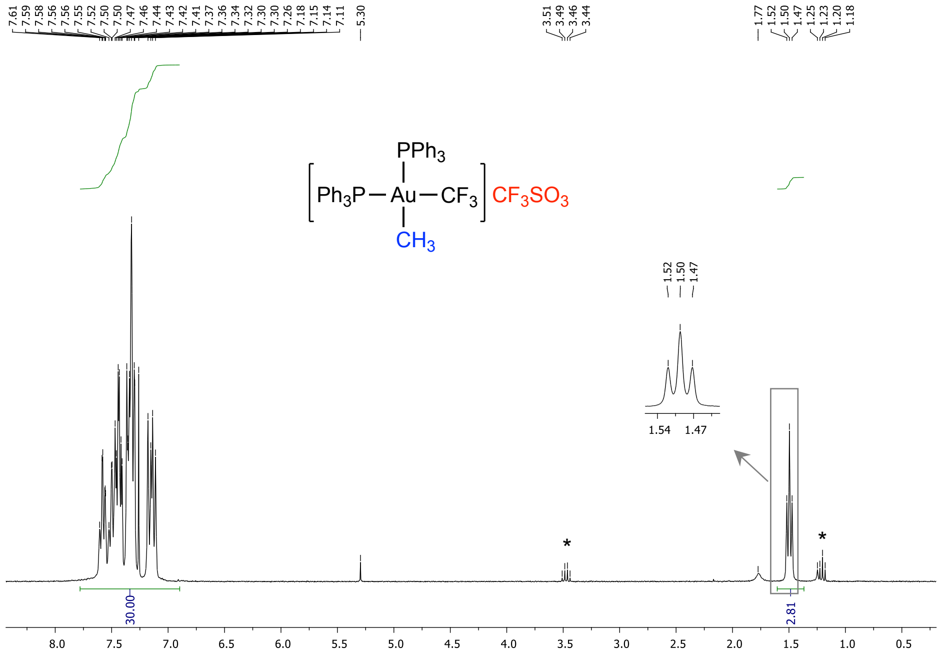


**Figure S52**. $^1$H NMR spectrum (300.1 MHz, $CDCl_3$) of **13**. $Et_2O$ signals marked with an asterisk.

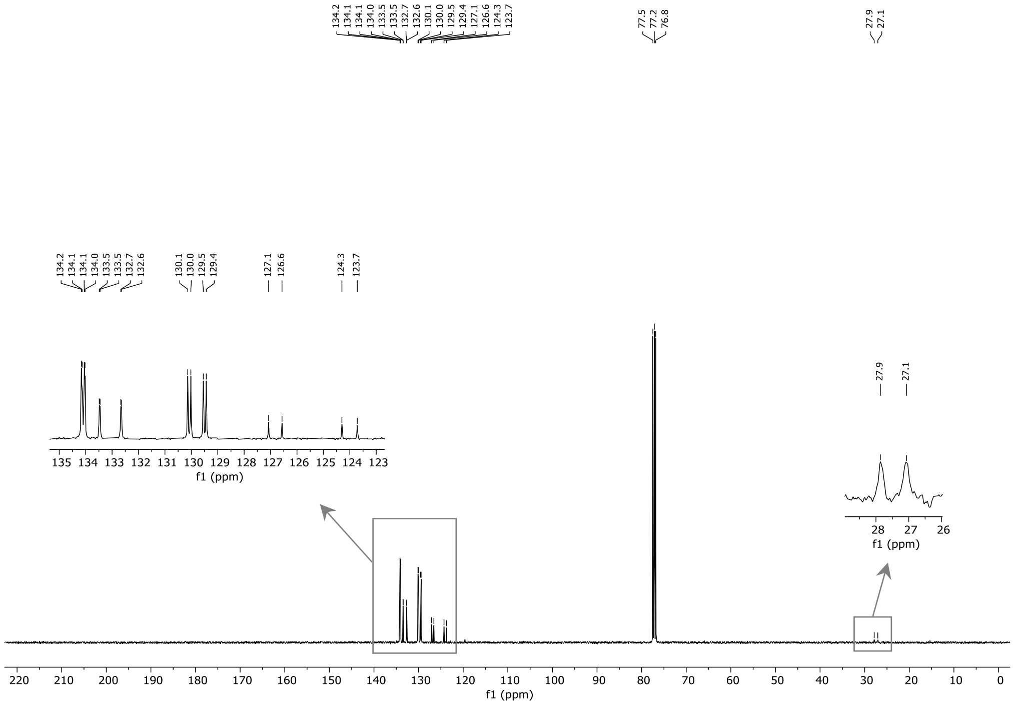


**Figure S53**. $^{13}C\{^1H\}$ NMR spectrum (100.8 MHz, $CDCl_3$) of **13**.

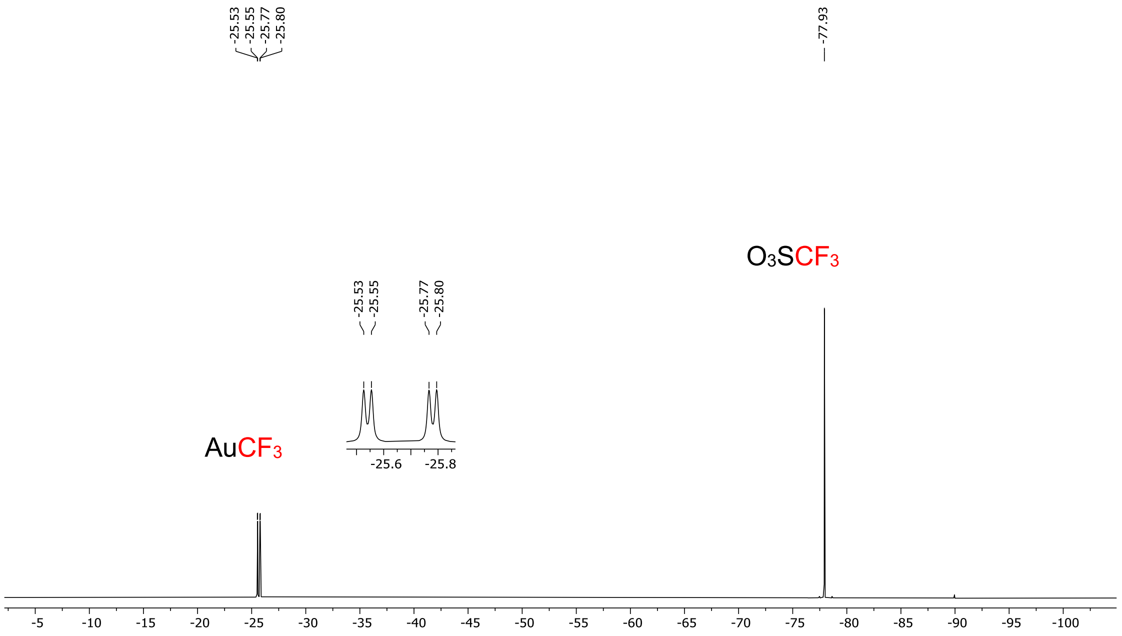


**Figure S54**. $^{19}$F NMR spectrum (282.4 MHz, $CDCl_3$) of **13**.

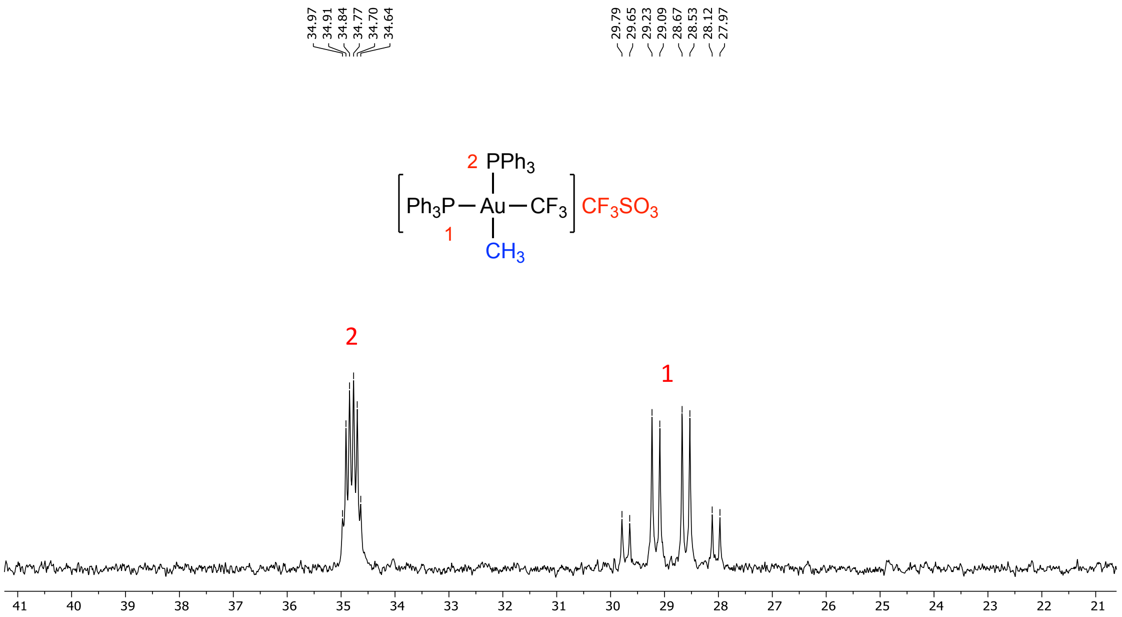


**Figure S55**. $^{31}$P{$^{1}$H} NMR spectrum (121.5 MHz, $CDCl_3$) of **13**.

**Formation of complexes of the types [Au($PR_3$)$_2$]X and [AuX($PR_3$)] in some acidolysis and reductive elimination reactions**

Complexes [Au($PR_3$)$_2$]X (R = Ph, X = OTf, $ClO_4$; R = Cy, X = OTf), were observed as secondary products during the acidolysis of **3a** with HOTf or $HClO_4$, or **3b** with HOTf. This is attributed to incipient decomposition of the unstable Au(III) complexes formed (**4a**, **4b** or **5a**) to give the corresponding MeX and [Au($CF_3$)($PR_3$)$_2$] (R = Ph (**1a**), Cy (**1b**)). In the presence of residual $H_2O$, **1a** and **1b** would undergo C–F hydrolysis to give HF and the unstable Au(I) carbonyls [Au(CO)($PR_3$)]$^+$,[6] which would then decompose to CO, [Au($PR_3$)$_2$]$^+$ and Au(0). This hydrolysis process is accelerated by the acidic medium.

In agreement with this, [Au($PPh_3$)$_2$]OTf and Au(0) were quantitatively formed when **1a** was treated with HOTf in $CDCl_3$. It was identified by comparison of its $^{31}P\{^1H\}$ NMR chemical shift (δ($^{31}P$) = 45.6 ppm) with the literature value,[7] and isolated (see below). The reaction of **1b** with HOTf in $CDCl_3$ gave Au(0) and a mixture of [Au($PCy_3$)$_2$]OTf (δ($^{31}P$) = 65.1 ppm)[8] and another complex, which was temptatively identified as [Au(OTf)($PCy_3$)] (δ($^{31}P$) = 58.2 ppm). We have not found $^{31}P$ NMR data for this compound in the literature, but we observed that it was formed when [Au(Me)($PCy_3$)] was treated with HOTf in $CDCl_3$.

Complexes [Au($PR_3$)$_2$]$^+$ were also observed as secondary products during the thermal decompositions of **4a**, **4b**, **5a**, **6a**, and **10a**. In these cases, the hydrolysis of **1a** or **1b** is accelerated by the acidic medium used to generate **4a**, **4b** and **5a**, or by the high temperatures necessary to promote the decompositions of **6a** (80 ºC) and **10a** (110 ºC).

Similarly, the formation of [AuX($PPh_3$)] (X = Cl or Br) as byproducts in the reactions of **3a** with HCl or HBr, or during the decompositions of **9a** or **8a**, could take place by reaction of the hydrolysis product [Au(CO)($PPh_3$)]$^+$ with the $Cl^-$ or $Br^-$ ions present in the reaction mixture.

**Isolation of [Au($PPh_3$)$_2$]OTf**. HOTf (6.0 µL, 0.07 mmol) was added to a solution of **1a** (75 mg, 0.14 mmol) in $CH_2Cl_2$ (5 mL) at room temperature. The mixture was stirred for 20 min, filtered and evaporated to dryness under vacuum. The resulting residue was stirred with $Et_2O$ (2 mL) and the suspension was filtered. The gray solid was washed with $Et_2O$ (2 × 2 mL) and air dried. Yield: 50 mg, 0.056 mmol, 80 %. $^1H$ NMR (200.1 MHz, $CDCl_3$): δ 7.66-7.47 (m, 30H, Ph). $^{19}F$ NMR (188.3 MHz, $CDCl_3$): δ -77.9 (s, $TfO^-$ ). $^{31}P\{^1H\}$ NMR (81.0 MHz, $CDCl_3$): δ 45.6 (s).

**Table S1**. Selected NMR data of the Au(III) complexes ($CDCl_3$, δ/ppm, J/Hz).

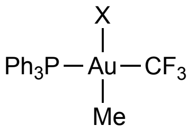

**3a–11a**

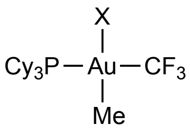

**3b, 4b, 8b**

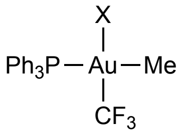

**3a'–11a'**

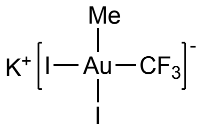

**12**[b]

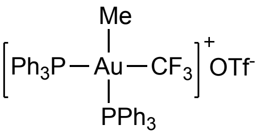

**13**

| | X | $AuCH_3$ δ($^1H$) | $AuCH_3$ $J_{PH}$ | δ($^{19}F$) | δ($^{31}P$) | $J_{PF}$ |
|---|---|---|---|---|---|---|
| **3a** | Me | 0.19 | 5.4 | -32.6 | 27.5 | 64.2 |
| **3b** | Me | 0.30 | 3.7 | -34.7 | 24.4 | 62.4 |
| **4a** | OTf | 1.6 | 6.2 | -32.9 ($AuCF_3$)<br>-77.1 (OTf) | 31.7 | 72.4 |
| **4b** | OTf | 1.72 | 4.6 | 40.1 | -36.8 | 65.2 |
| **5a** | $OClO_3$ | 1.6 | 6.0 | -34.4 | 32.3 | 72.8 |
| **6a** | $ONO_2$ | 1.4 | 5.9 | -37.2 | 29.0 | 71.7 |
| **7a** | $OC(O)CF_3$ | 1.3 | 5.8 | -36.3 ($AuCF_3$)<br>-75.0 ($CF_3CO$) | 29.4 | 71.4 |
| **10a** | F | 1.0 | 6.0 | -37.6 ($AuCF_3$)<br>-223.7 (AuF) | 25.8 | 72.3 ($^3J_{PFtrans}$)<br>25.3 ($^2J_{PFcis}$) |
| **9a** | Cl | 1.1 | 6.0 | -31.8 | 27.7 | 71.1 |
| **8a** | Br | 1.3 | 5.9 | -28.9 | 26.7 | 70.1 |
| **8b** | Br | 1.48 | 4.2 | -31.0 | 30.2 | 65.6 |
| **11a** | I | 1.5 | 5.8 | -23.4 | 24.1 | 68.9 |
| **13** | – | 1.5 | 7.0 | -25.7 | 28.9 (*trans* to $CF_3$)<br>34.8 | 67.8 ($^3J_{PFtrans}$)<br>8.0 ($^3J_{PFcis}$) |
| **3a'** | Me | 1.3<br>0.2 | 8.1<br>8.6 | -31.2 | 31.4 | 6.6 |
| **4a'** | OTf | 2.0 | 8.9 | -16.5 ($AuCF_3$)<br>-77.1 (OTf) | – [a] | 10.7 |
| **5a'** | $OClO_3$ | 2.0 | 9.0 | -16.0 | – [a] | 11.8 |
| **6a'** | $ONO_2$ | 1.7 | 8.9 | -17.9 | 45.6 | 11.4 |
| **7a'** | $OOCCF_3$ | 1.6 | 8.9 | -19.1 ($AuCF_3$)<br>-75.0 ($CF_3CO$) | 45.2 | 11.6 |
| **10a'** | F | 1.7 | 9.5 | -19.5 ($AuCF_3$)<br>-246.6 (AuF) | 44.1 | 9.4 ($^3J_{PFcis}$)<br>46.8 ($^2J_{PFtrans}$) |
| **9a'** | Cl | 1.8 | 9.4 | -22.4 | 39.9 | 9.3 |
| **8a'** | Br | 1.9 | 9.2 | -23.5 | 37.3 | 9.2 |
| **11a'** | I | 2.0 | 9.7 | -26.4 | 33.1 | 9.1 |
| **12** | – | 1.9 | – | -24.4 | – | – |

[a] The signals were not detected because of their low intensity. [b] A *cis* configuration is proposed for **12** on the basis of the stronger *trans* influence of $CF_3$ and Me compared to I.

## 3. Reductive elimination reactions

### General procedure

A solution of the corresponding Au(III) complex in the indicated solvent was heated in a NMR tube under a $N_2$ atmosphere at the indicated temperature. The progress of the reaction was monitored by $^{1}H$, $^{19}F$ and $^{31}P\{^{1}H\}$ NMR spectroscopy. For kinetic measurements, the concentrations of the complexes were measured by integration of the $^{19}F$-NMR spectra using trifluoromethylbenzene as internal standard. The yields of the formed MeX products with X = OTf, $OClO_3$, $ONO_2$ (Table 1 of the article) were determined by integration of the methyl signals of the Au(III) complex and the formed MeX in the $^{1}H$ NMR spectra before and after the reactions. The total integral of the phosphine protons was used as standard value. This method underestimated the amount of formed MeX for those methyl derivatives having a low boiling point (X = Me, F, Cl, Br and $OCOCF_3$), because a significant fraction of the formed MeX was in the head space of the NMR tube as a gas. For this reason, the yield of the MeX products with X = Me, Br and $OCOCF_3$ were indirectly estimated from the concentration of formed Au(I) complex (**1a**), which was accurately determined in the reaction monitoring experiments.

### 3.1. Reductive elimination of ethane from 3a or 3b

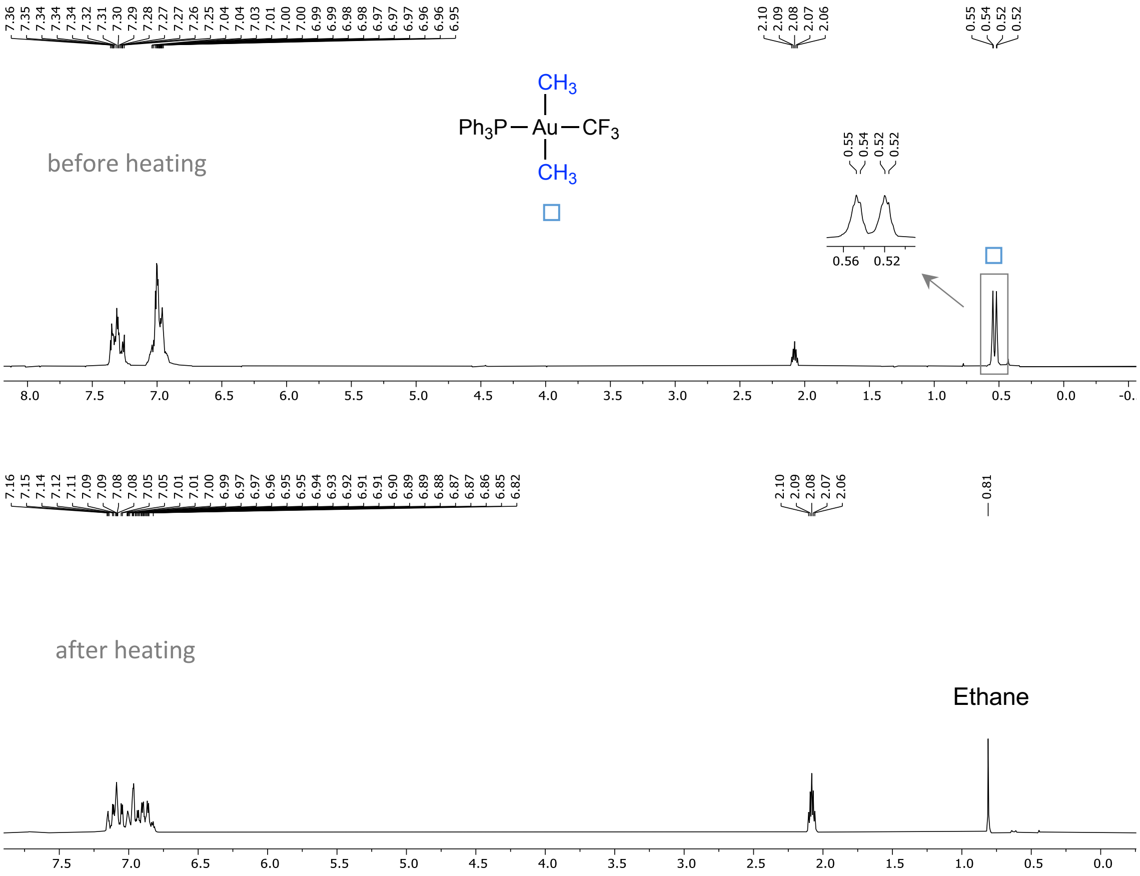


**Figure S56**. $^{1}H$ NMR spectrum (200.1 MHz, $D_8$-Toluene) of **3a** before and after heating for 27 h at 100 ºC.

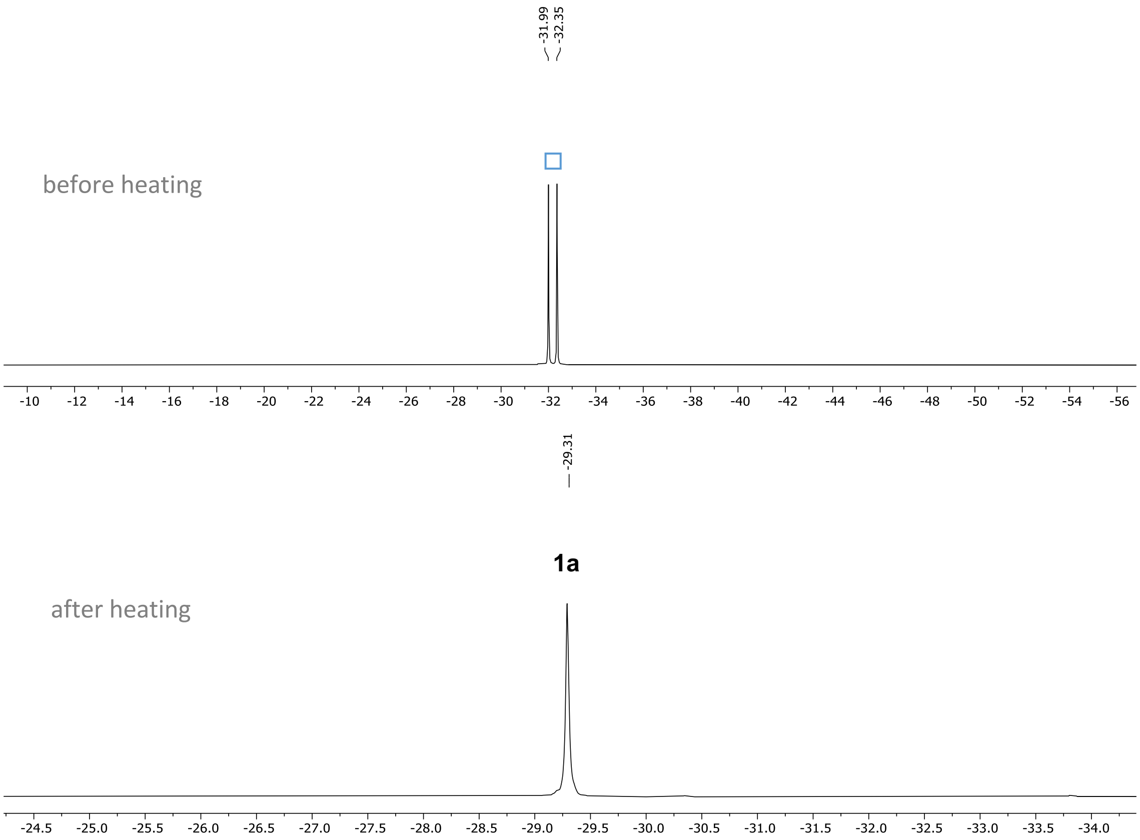


**Figure S57**. $^{19}F$ NMR spectrum (188.3 MHz, $D_8$-Toluene) of **3a** before and after heating for 27 h at 100 ºC.

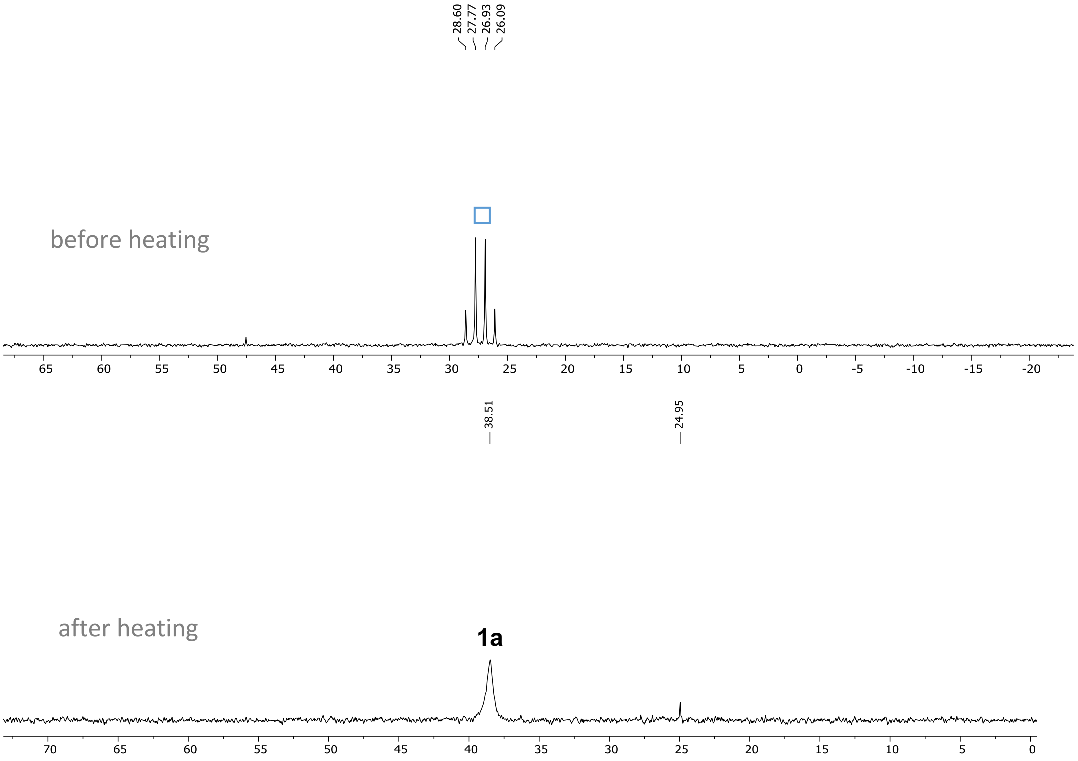


**Figure S58**. $^{31}P\{^1H\}$ NMR spectrum (81.0 MHz, $D_8$-Toluene) of **3a** before (top) and after (bottom) heating for 27 h at 100 ºC.

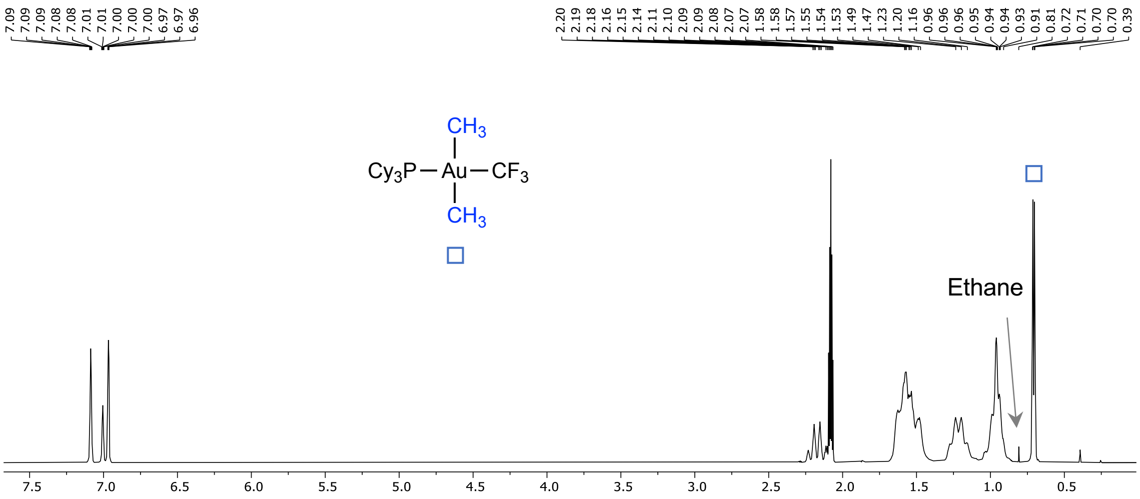


**Figure S59**. $^{1}H$ NMR spectrum (300.1 MHz, $D_8$-Toluene) of **3b** after heating for 14 h at 140 ºC.

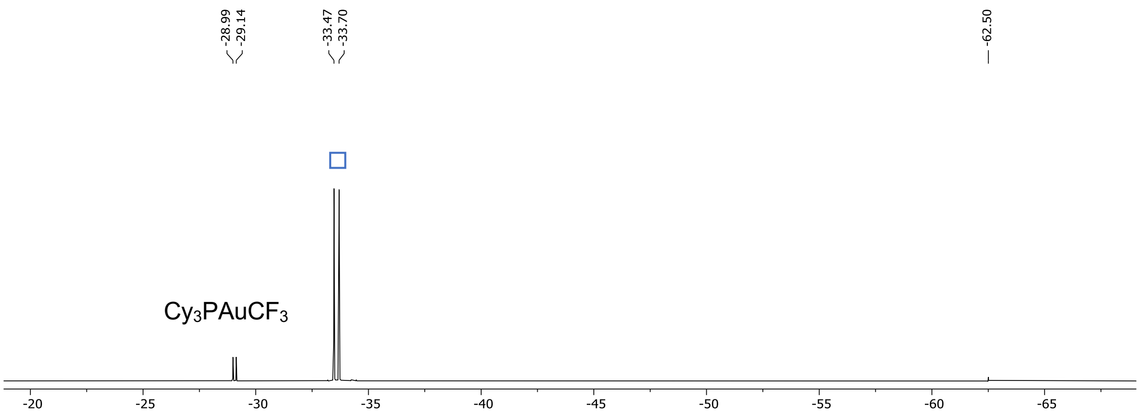


**Figure S60**. $^{19}F$ NMR spectrum (282.4 MHz, $D_8$-Toluene) of **3b** after heating for 14 h at 140 ºC.

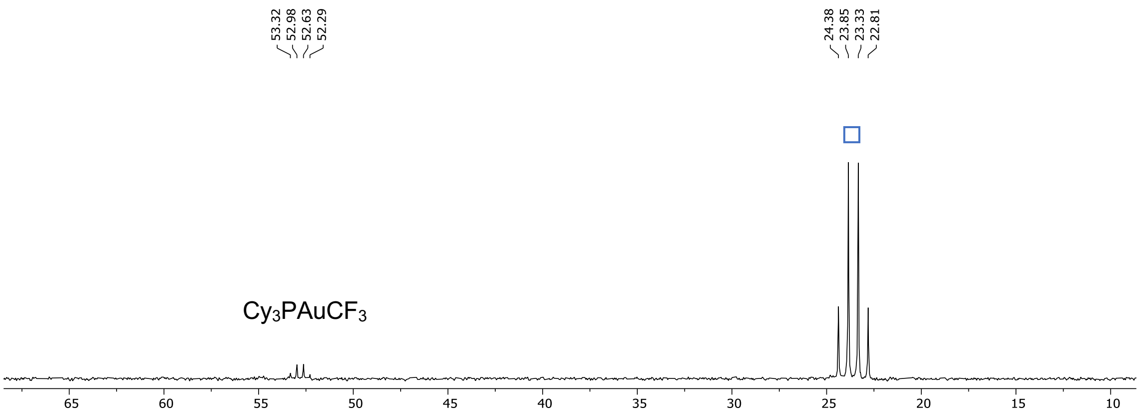


**Figure S61**. $^{31}P\{^{1}H\}$ NMR spectrum (121.5 MHz, $D_8$-Toluene) of **3b** after heating for 14 h at 140 ºC.

### 3.2. Reaction of 3a with HOTf in THF. Isomerization to *cis*-[Au($CF_3$)(Me)$_2$($PPh_3$)] (3a') and reductive elimination of ethane.

HOTf (2 µL, 0.02 mmol) was added to a solution of **3a** (13 mg, 0.023 mmol) in $D_8$-THF (0.5 mL). The NMR spectra of the mixture showed gradual conversion to **3a'**, ethane and **1a**. The NMR data of **3a'** are given in Table S1.

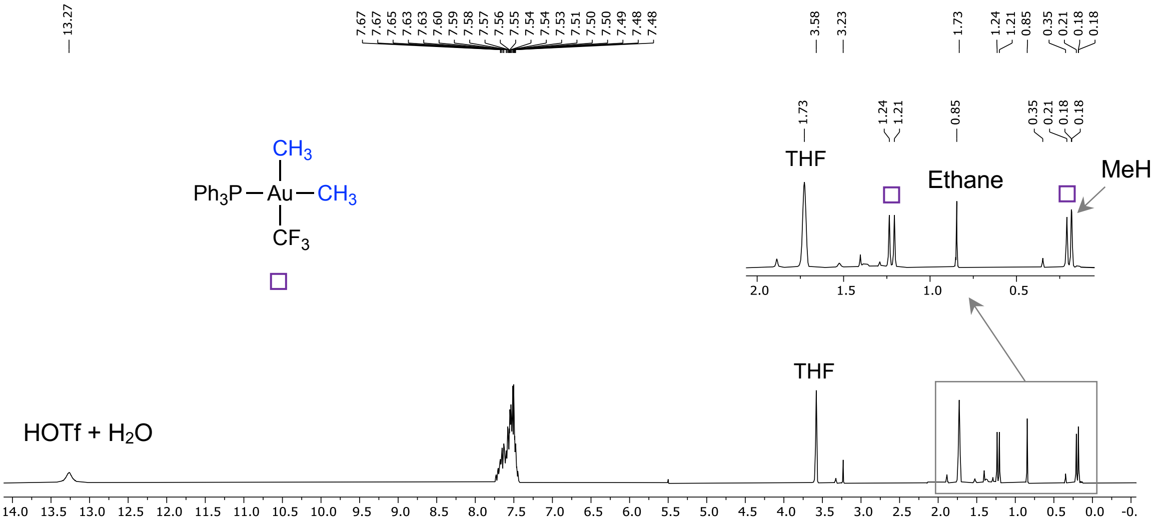


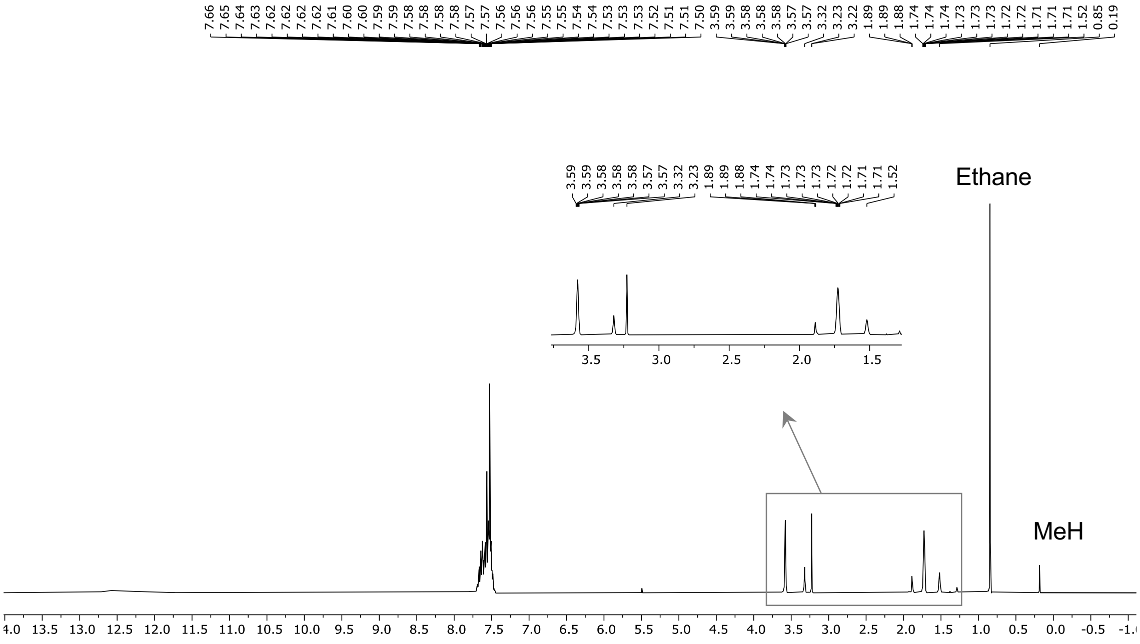


**Figure S62**. $^{1}$H NMR spectrum (300.1 MHz, $D_8$-THF) of the reaction mixture of **3a** with HOTf after 2 (top) and 19 h (bottom) of the addition of acid at room temperature. The signals at 3.58 and 1.72 ppm correspond to $D_7$-THF. The signals at 3.32, 3.23, 1.89 and 1.52 ppm are attributed to products derived from the reactions between THF, HOTf, MeOTf and residual water. Acidolysis of **3a'** could be a secondary reaction pathway giving rise to the observed methane and [Au($CF_3$)(Me)(OTf)($PPh_3$)] (isomer of **4a**), which would decompose to MeOTf and **1a**.

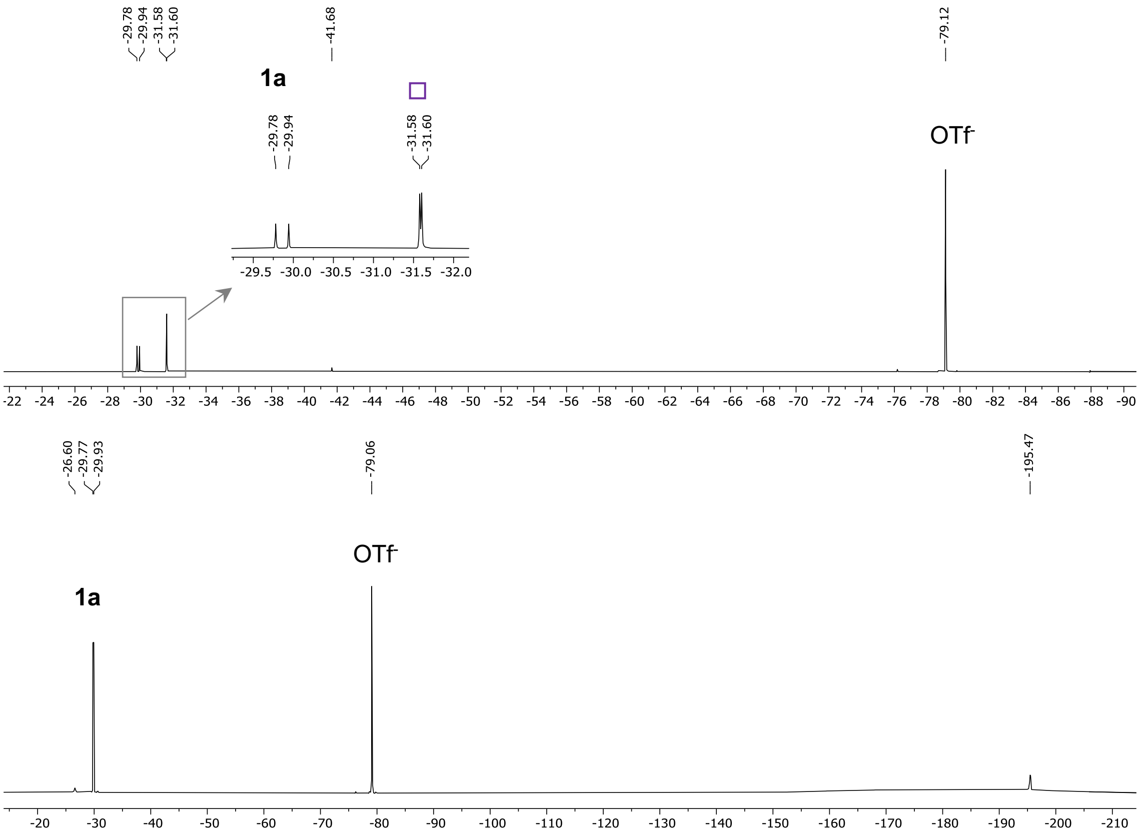


**Figure S63**. $^{19}F$ NMR spectrum (282.4 MHz, $D_8$-THF) of the reaction mixture of **3a** with HOTf after 2 (top) and 19 h (bottom) of the addition of acid at room temperature.

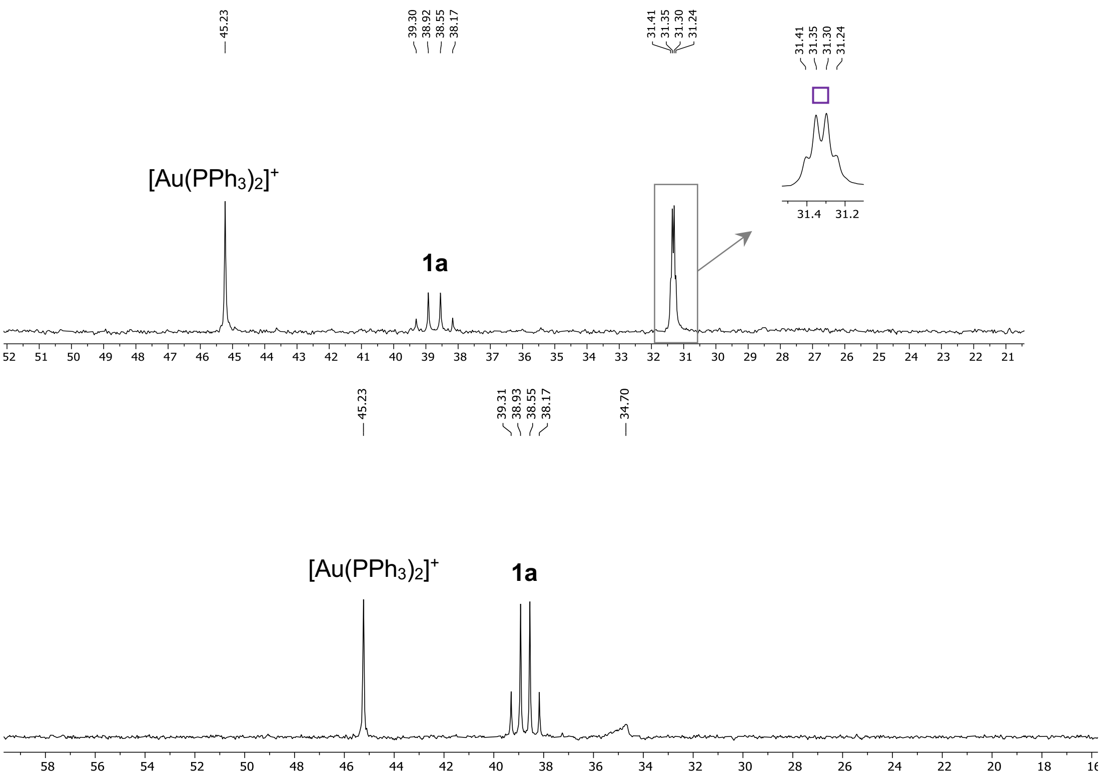


**Figure S64**. $^{31}P\{^1H\}$ NMR spectrum (121.5 MHz, $D_8$-THF) of the reaction mixture of **3a** with HOTf after 2 (top) and 19 h (bottom) of the addition of acid at room temperature.

### 3.3. Reductive elimination of MeOTf from 4a or 4b

Solutions of complexes **4a** or **4b** were in situ generated by addition of a small excess of HOTf to a solution of **3a** or **3b** in $CDCl_3$. These mixtures were heated at 50 °C and monitored by NMR spectroscopy. MeOTf was identified by comparing the chemical shifts of the observed signals with previously reported data ($\delta(^1H)$ = 4.22 ppm; $\delta(^{19}F)$ = -74.1 ppm).[9] The reaction products $[Au(CF_3)L]$ (L = $PPh_3$ (**1a**) or $PCy_3$ (**1b**)) were not observed because in the acidic reaction conditions they undergo C–F hydrolysis by residual water to give [Au(CO)L]OTf and products derived from the reaction of the fluoride with the glass NMR tubes. These unstable Au(I) carbonyls decompose to Au(0), $[AuL_2]OTf$ (L = $PPh_3$, $PCy_3$) and [Au(OTf)L] (only for L = $PCy_3$, see Fig. S70). Formation of these products by reaction of **1a** or **1b** and HOTf in $CDCl_3$ has been confirmed (see page S34).

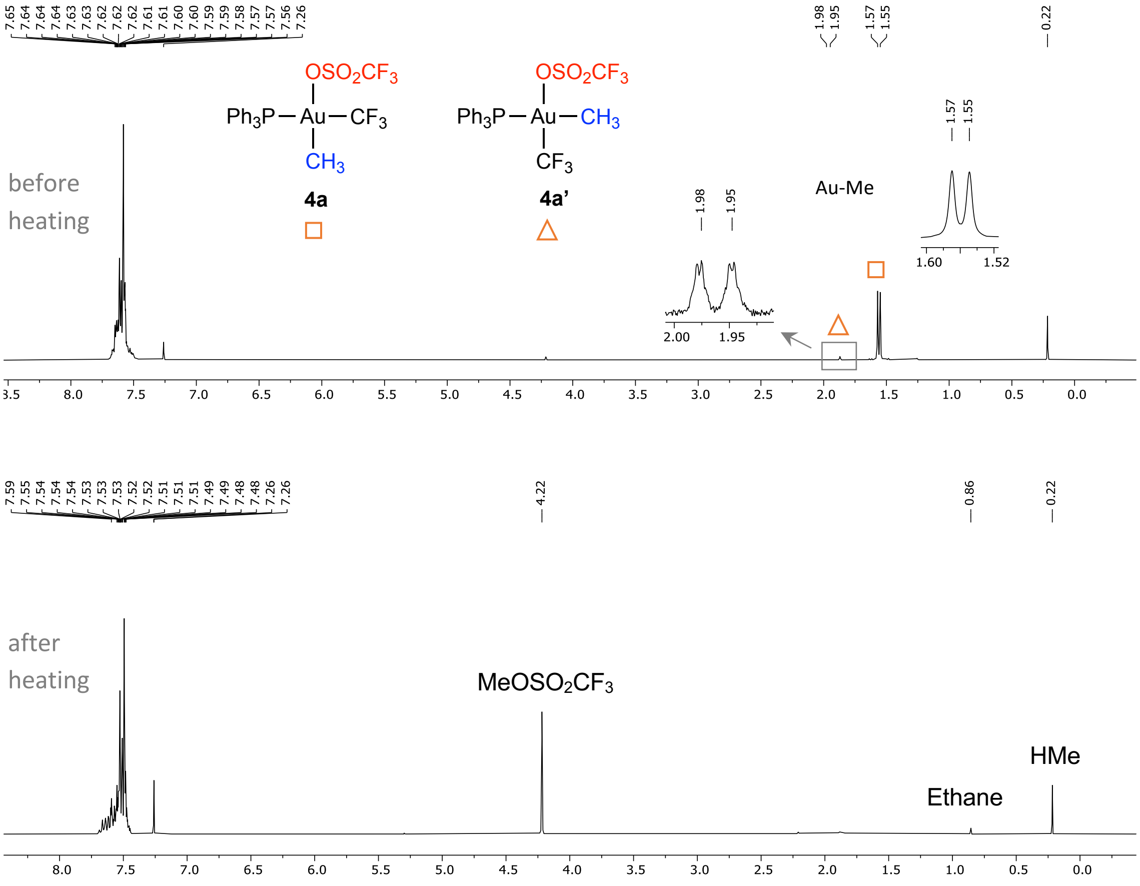


**Figure S65**. $^1H$ NMR spectrum (300.1 MHz, $CDCl_3$) of in situ generated **4a** before and after heating for 3.5 h at 50 °C.

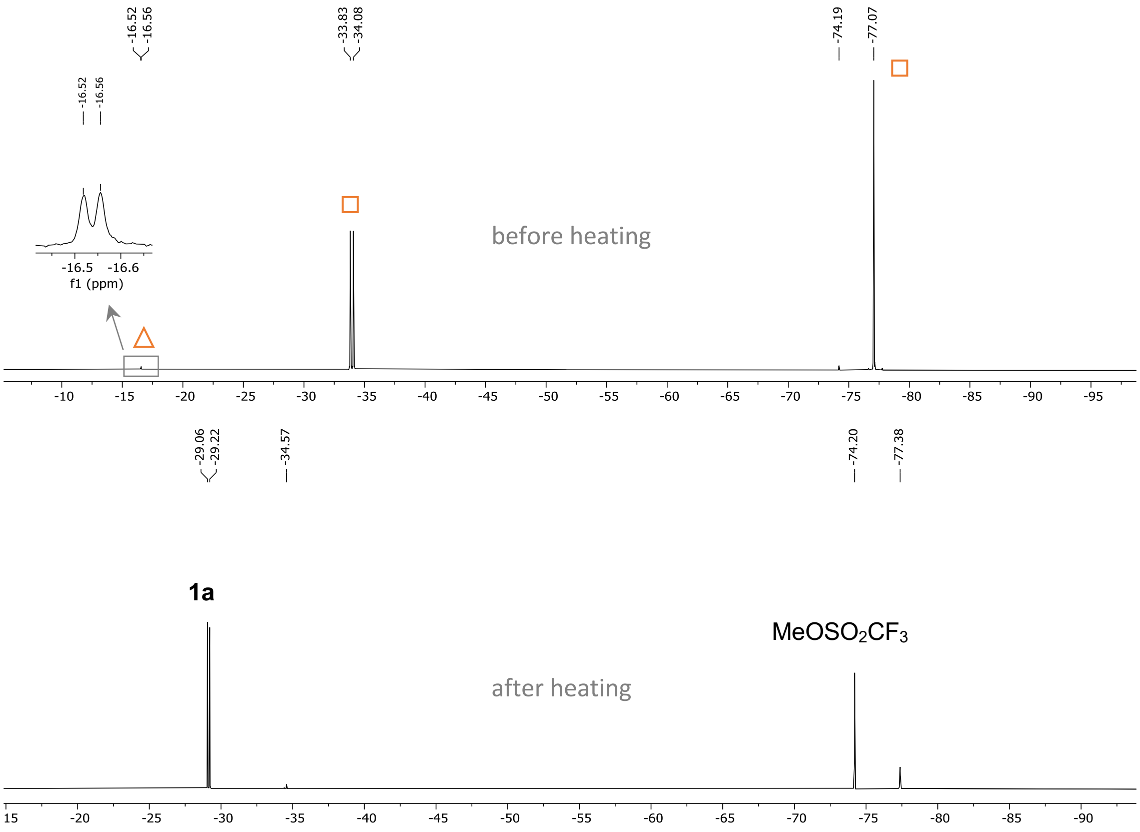


**Figure S66**. $^{19}$F NMR spectrum (282.4 MHz, $CDCl_3$) of in situ generated **4a** before and after heating for 3.5 h at 50 ºC.

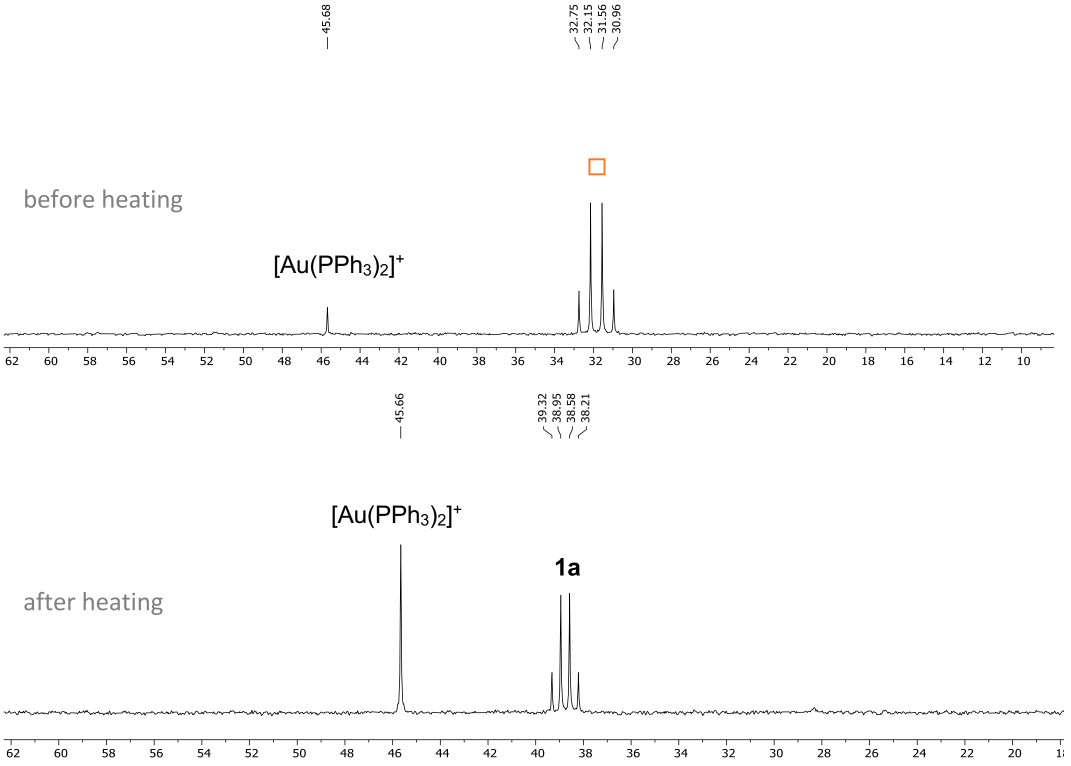


**Figure S67**. $^{31}P\{^1H\}$ NMR spectrum (121.5 MHz, $CDCl_3$) of in situ generated **4a** before and after heating for 3.5 h at 50 ºC.

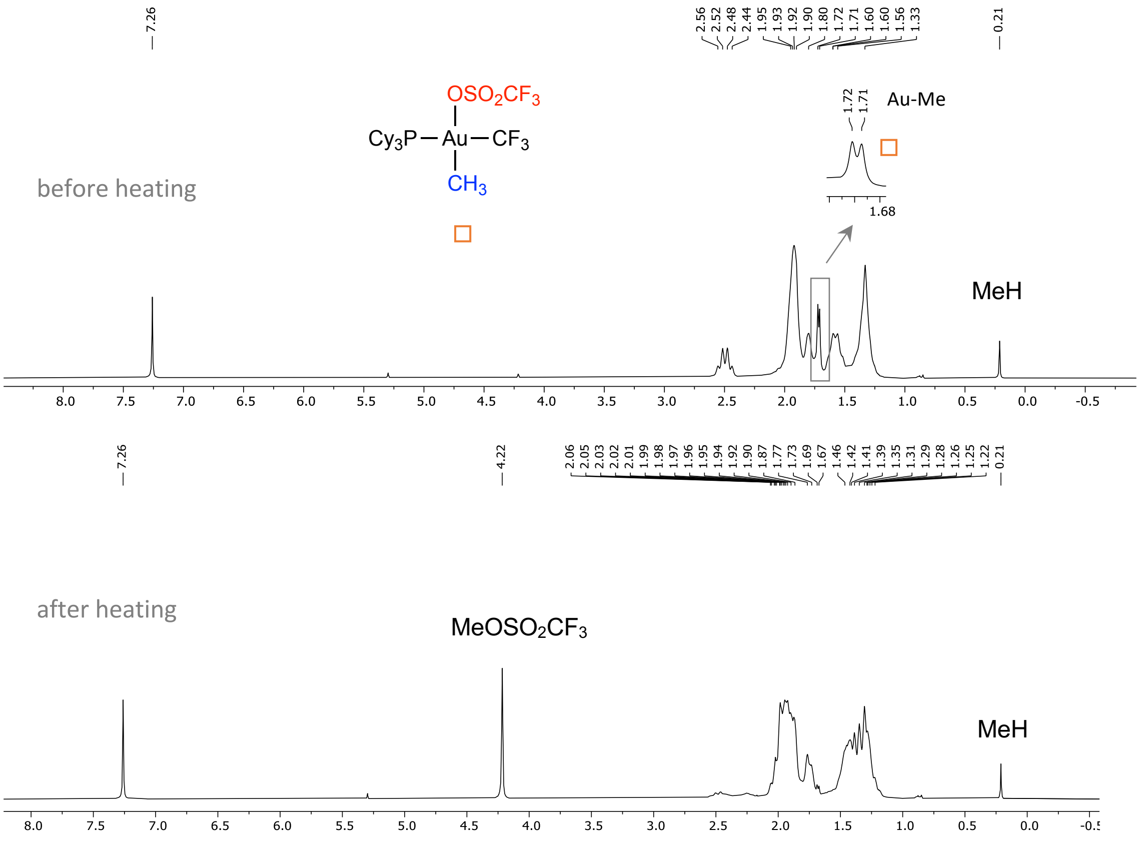


**Figure S68**. $^1H$ NMR spectrum (300.1 MHz, $CDCl_3$) of in situ generated **4b** before and after heating for 4.5 h at 50 ºC.

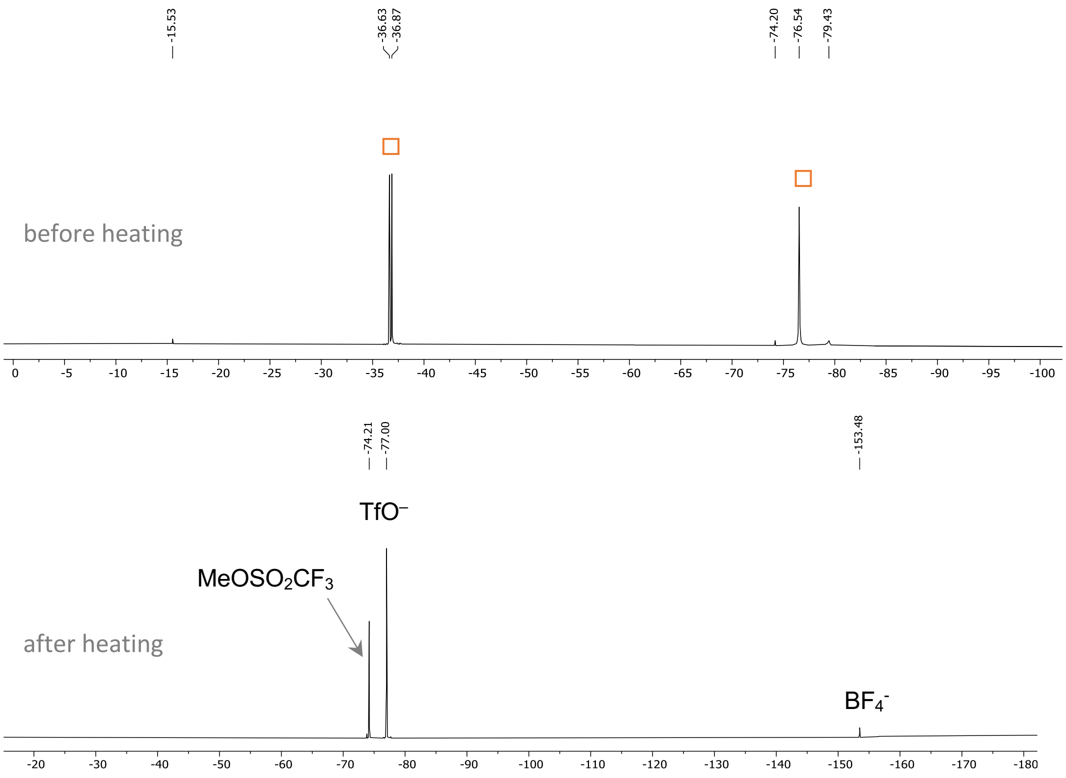


**Figure S69**. $^{19}F$ NMR spectrum (282.4 MHz, $CDCl_3$) of in situ generated **4b** before and after heating for 4.5 h at 50 ºC.

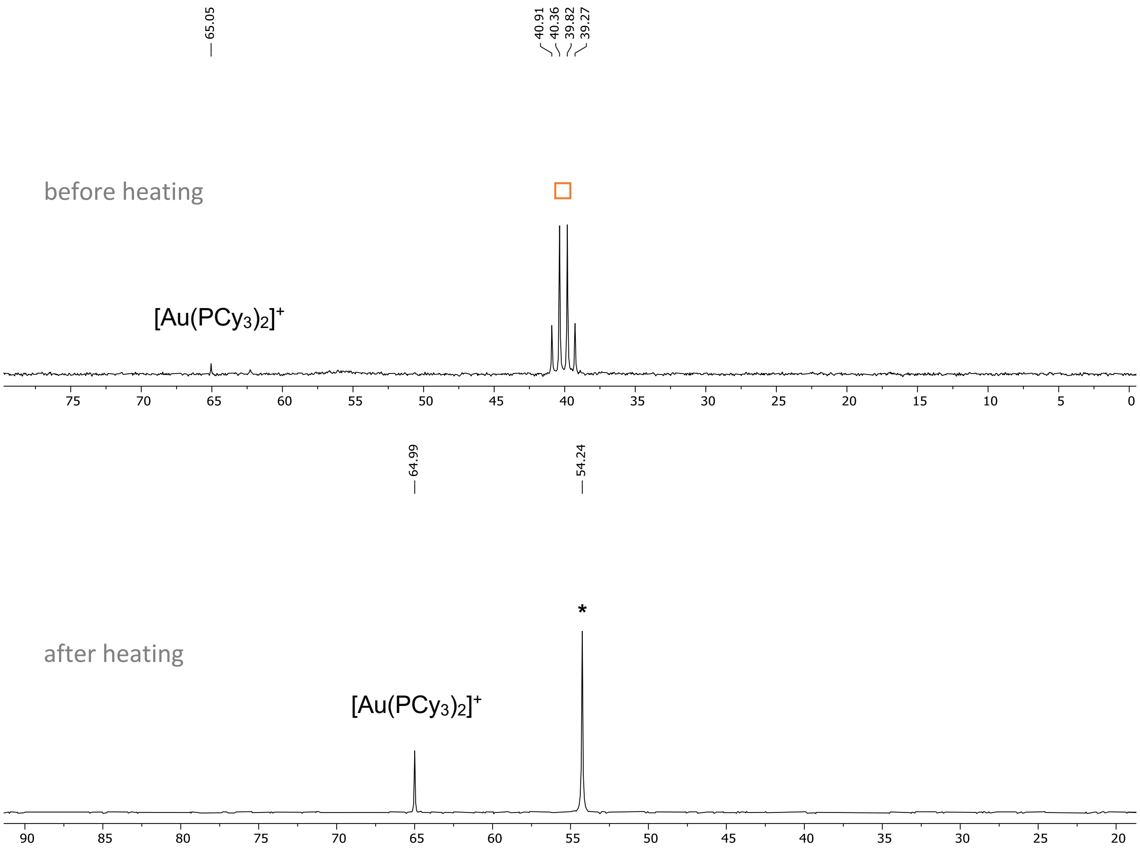


**Figure S70**. $^{31}P\{^1H\}$ NMR spectrum (121.5 MHz, $CDCl_3$) of in situ generated **4b** before and after heating for 4.5 h at 50 ºC. The chemical shift of $[Au(PCy_3)]_2^+$ is in agreement with the literature value.[8] The signal labeled with an asterisk is assigned to $[Au(OTf)(PCy_3)]$. Both complexes (and metallic gold) were formed when $[AuMe(PCy_3)]$ or $[Au(CF_3)(PCy_3)]$ were treated with HOTf in $CDCl_3$ (see page S34).

### 3.4. Reductive elimination of $MeOClO_3$ from 5a.

$MeOClO_3$ was identified by comparing the chemical shift of the observed $^1H$ NMR signal with the previously reported chemical shift value ($\delta(^1H)$ = 4.25 ppm).[9] As in the decomposition of **4a**, most of the formed **1a** was transformed into $[Au(PPh_3)_2]ClO_4$.

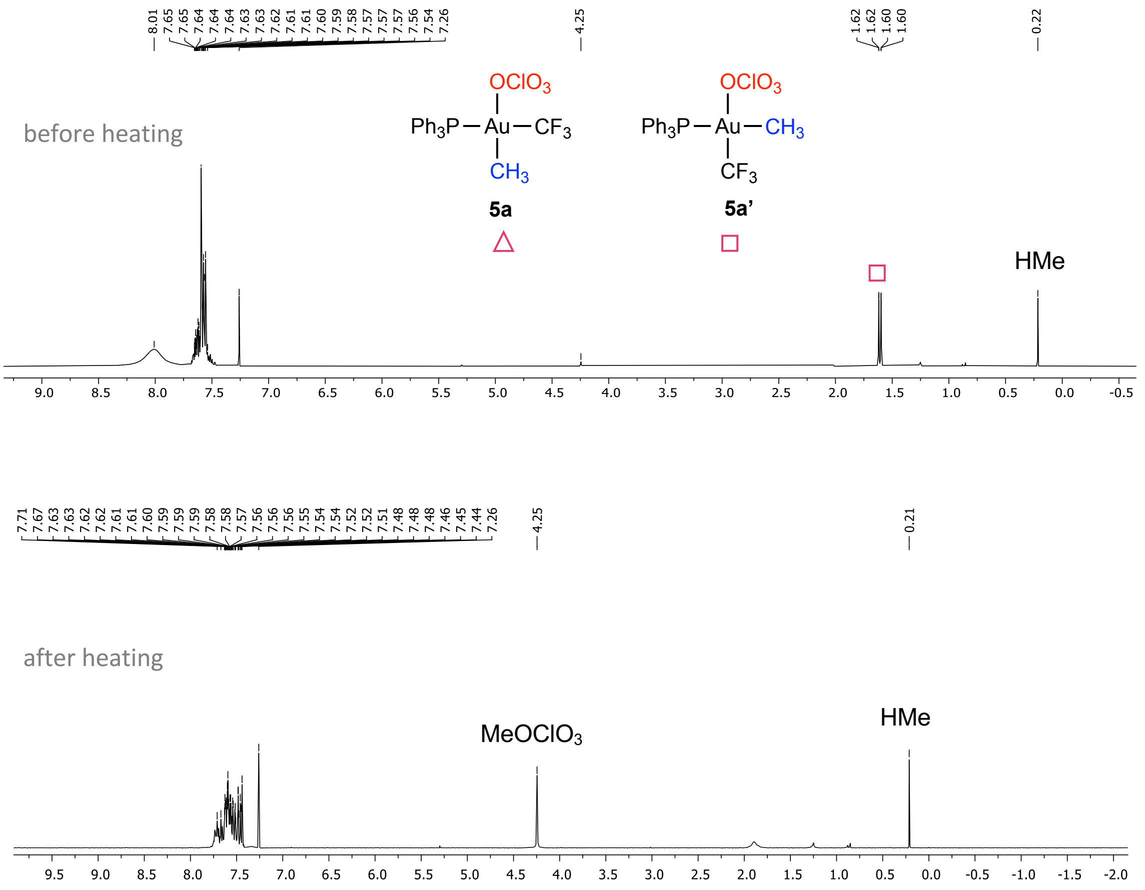


**Figure S71**. $^1H$ NMR spectrum (300.1 MHz, $CDCl_3$) of in situ generated **5a** (from **3a** and 72 % $HClO_4$) before and after heating for 1 h at 50 ºC.

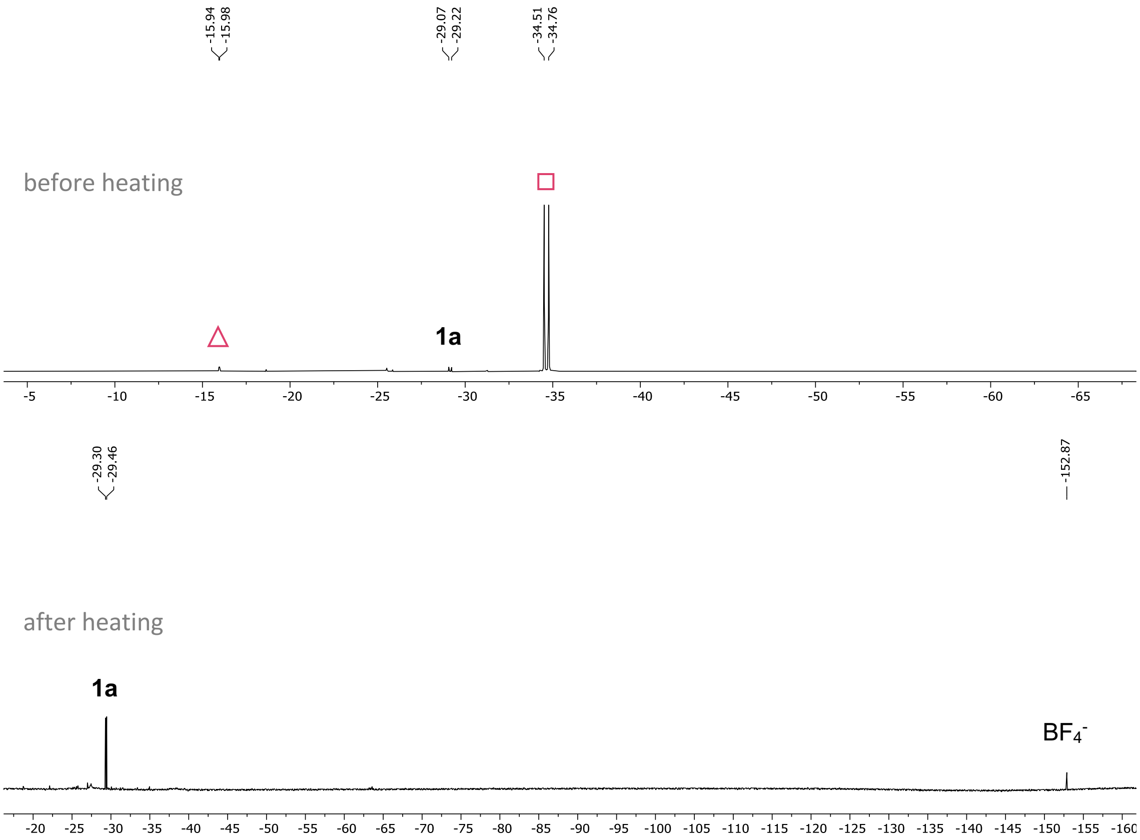


**Figure S72**. $^{19}$F NMR spectrum (282.4 MHz, $CDCl_3$) of in situ generated **5a** (from **3a** and 72 % $HClO_4$) before and after heating for 0.5 h at 50 ºC.

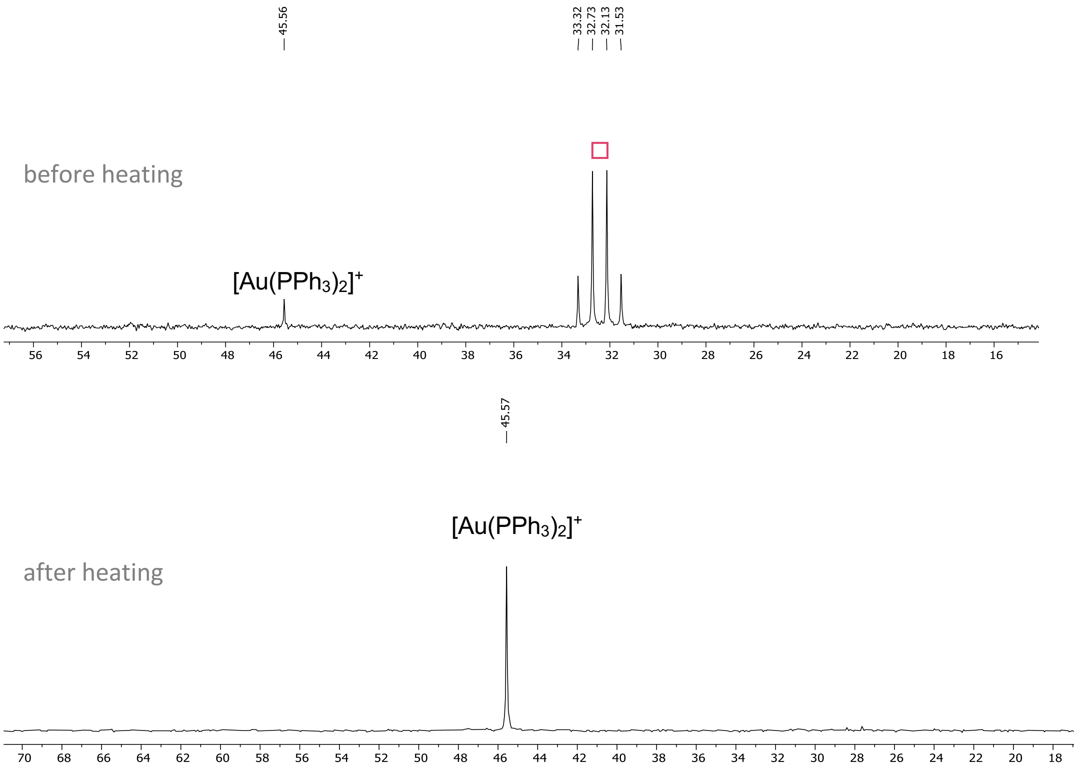


**Figure S73**. $^{31}P\{^1H\}$ NMR spectrum (121.5 MHz, $CDCl_3$) of in situ generated **5a** (from **3a** and excess 72 % $HClO_4$) before and after heating for 0.5 h at 50 ºC.

### 3.5. Reductive elimination of $MeONO_2$ from 6a and 6a'

$MeONO_2$ was identified by comparing the chemical shift of the observed $^1H$ NMR signal with: (a) the previously reported chemical shift value ($\delta(^1H)$ = 4.10 ppm in $CDCl_3$);[11] (b) the chemical shift of an independently-prepared sample ($\delta(^1H)$ = 4.10 ppm in $CDCl_3$) by the reaction of $AgNO_3$ with MeI.

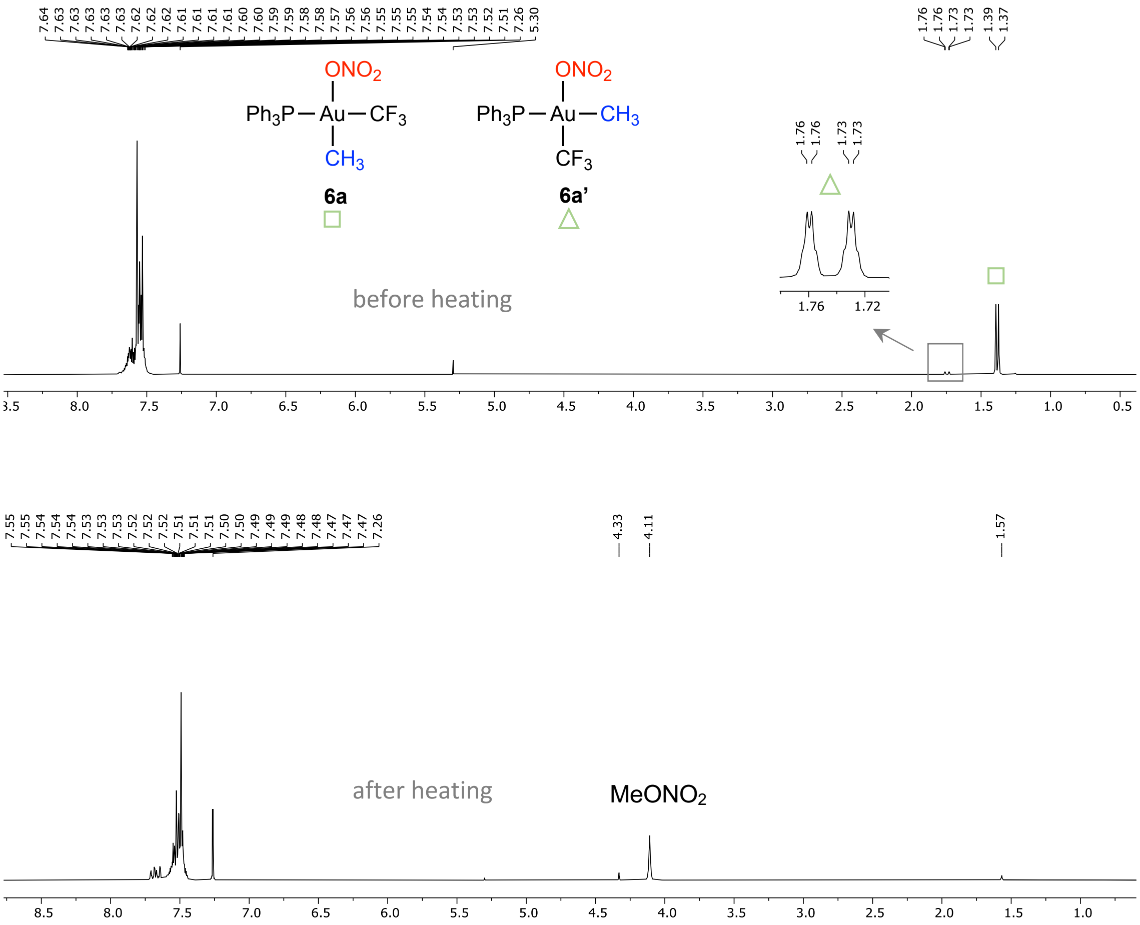


**Figure S74**. $^1H$ NMR spectra (300.1 MHz, $CDCl_3$) of a mixture of **6a** and **6a'** before and after heating for 6 h at 80 ºC.

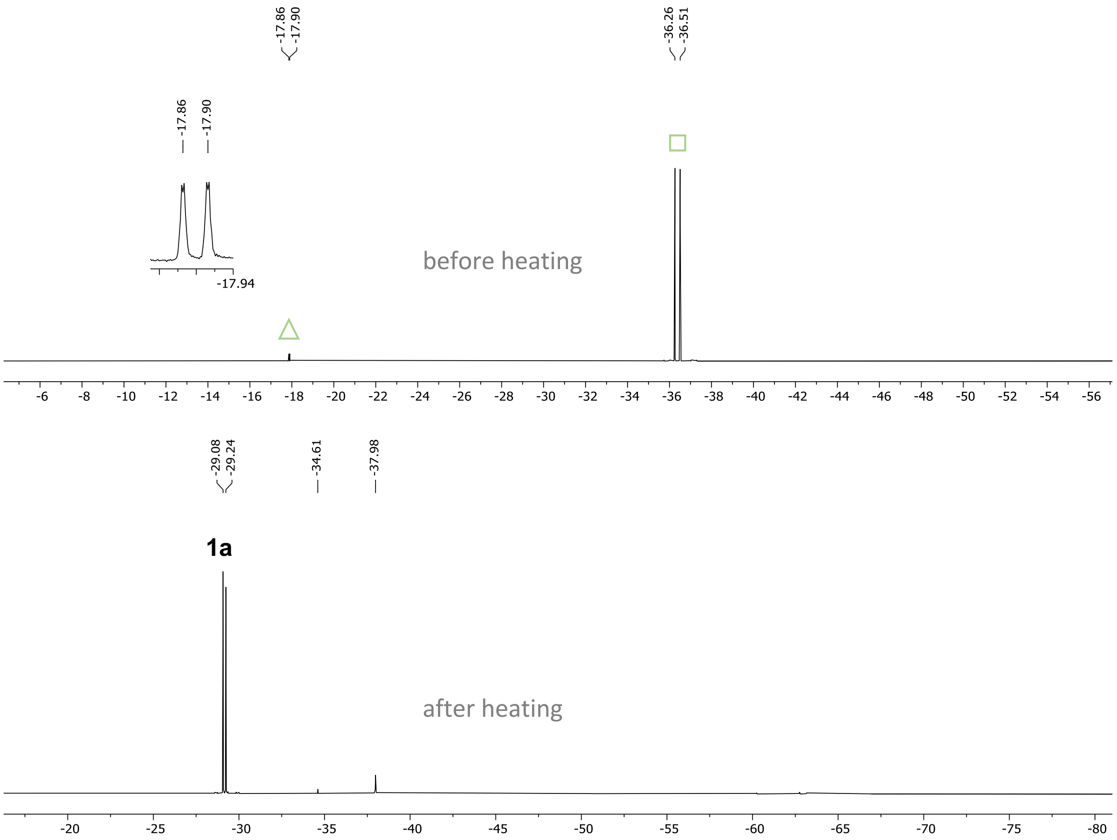


**Figure S75**. $^{19}F$ NMR spectrum (282.4 MHz, $CDCl_3$) of a mixture of **6a** and **6a'** before and after heating for 6 h at 80 ºC.

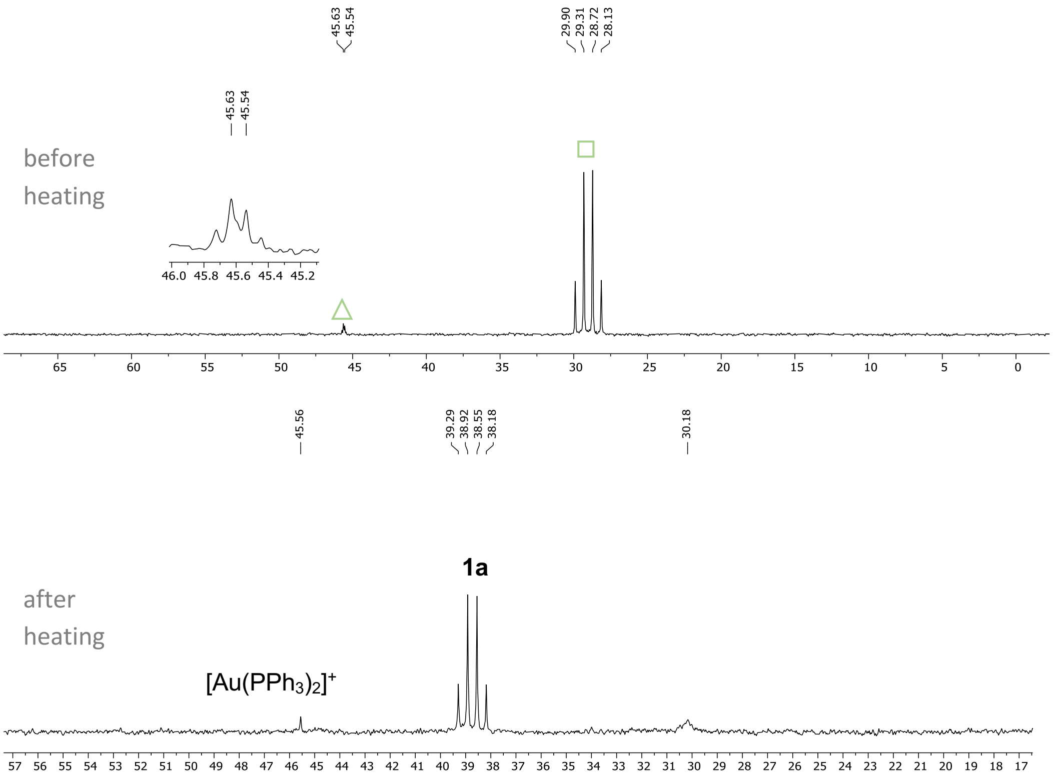


**Figure S76**. $^{31}P\{^1H\}$ NMR spectrum (121.5 MHz, $CDCl_3$) of a mixture of **6a** and **6a'** before and after heating for 6 h at 80 ºC.

### 3.6. Reductive elimination of $MeOC(O)CF_3$ from 7a and 7a'

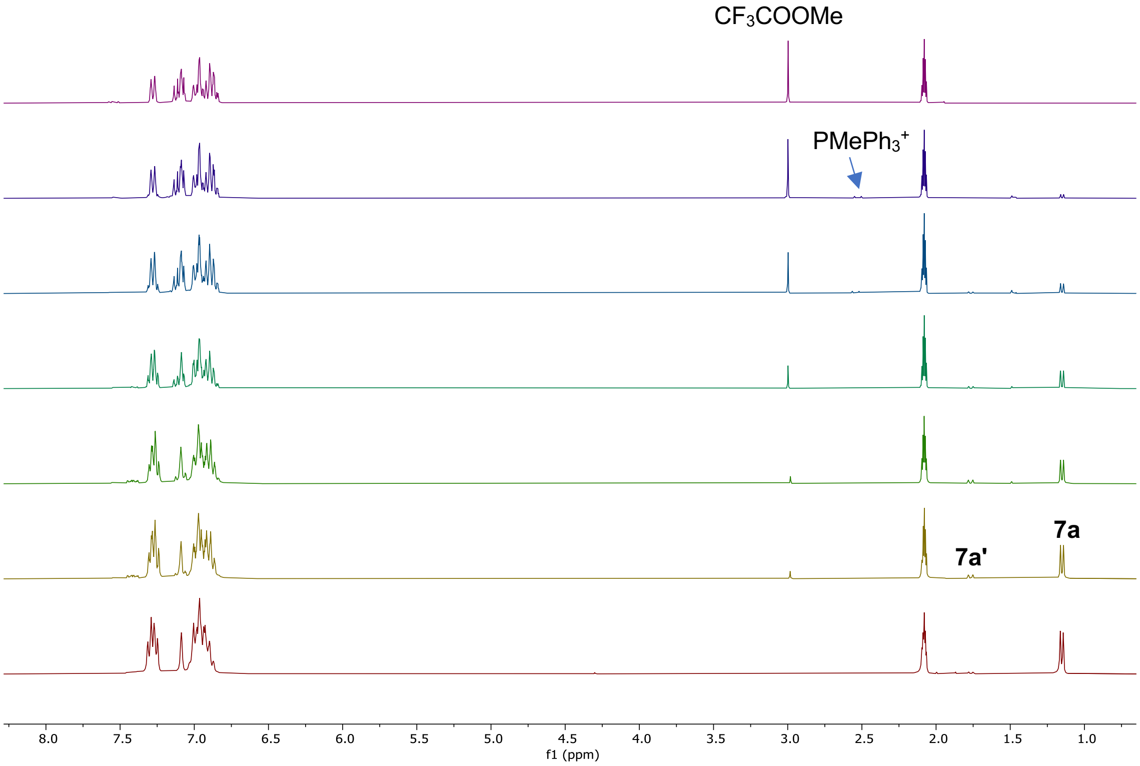


**Figure S77**. $^{1}H$ NMR spectra (300.1 MHz, $D_8$-Toluene) showing the course of the decomposition of **7a** at 100°C. From bottom to top, time = 0, 3.5, 6.25, 9.5, 12.5, 15 and 17.5 h.

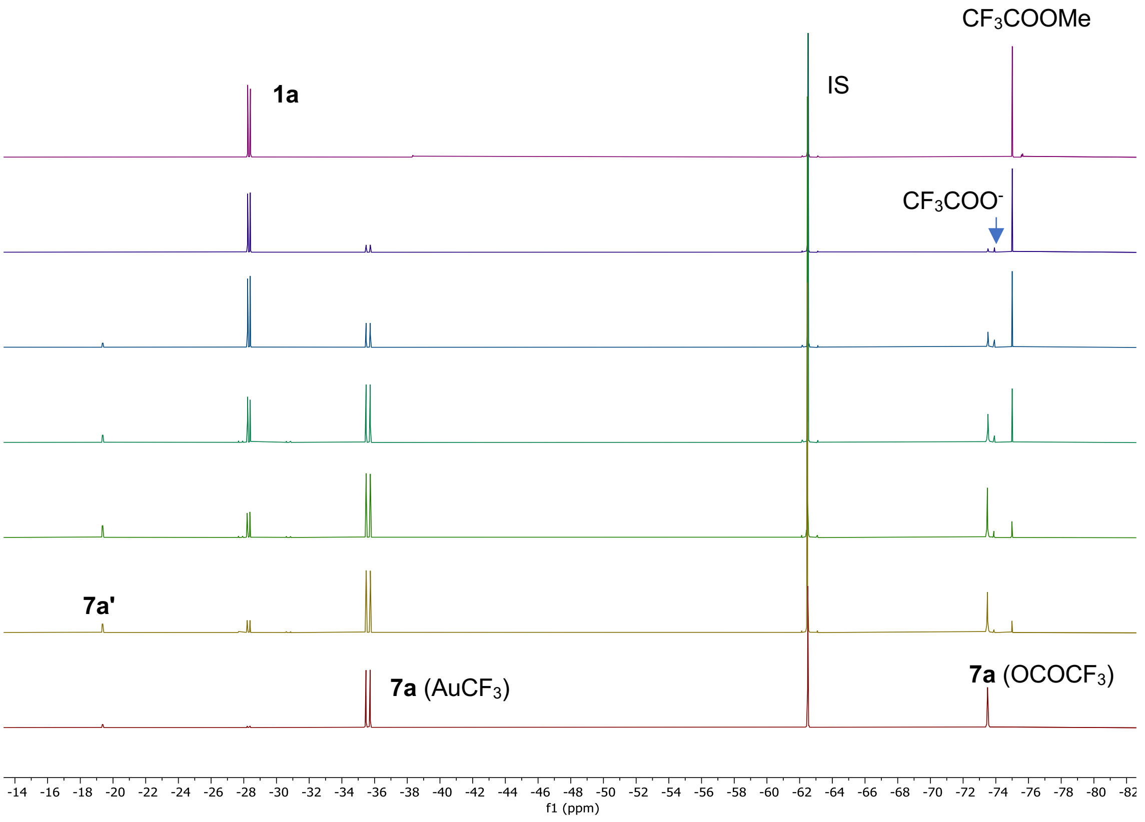


**Figure S78.** $^{19}F$ NMR spectra (282.4 MHz, $D_8$-Toluene) showing the course of the decomposition of **7a** at 100°C. From bottom to top, time = 0, 3.5, 6.25, 9.5, 12.5, 15 and 17.5 h. IS = Internal standard (trifluoromethylbenzene).

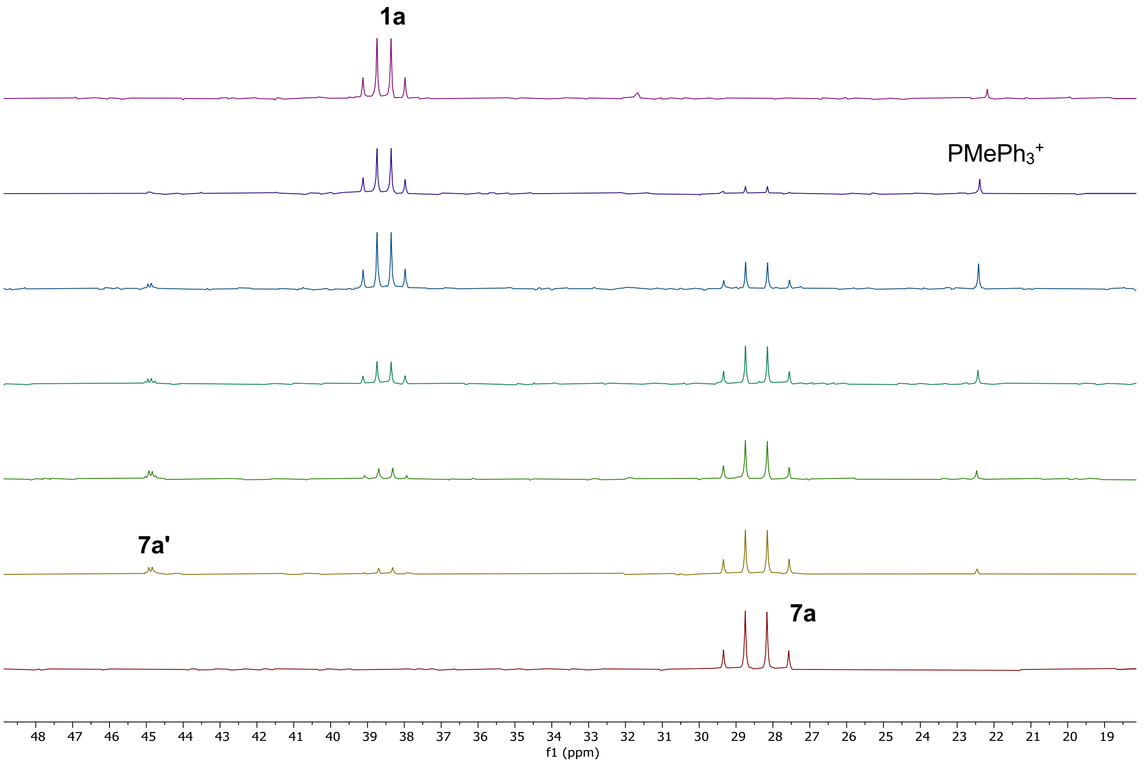


**Figure S79.** $^{31}P\{^1H\}$ NMR spectra (121.5 MHz, $D_8$-Toluene) showing the course of the decomposition of **7a** at 100°C. From bottom to top, time = 0, 3.5, 6.25, 9.5, 12.5, 15 and 17.5 h.

### 3.7. Reductive elimination of MeBr from 8a and 8a' or from 8b

a) Reductive elimination of MeBr from **8a** and **8a'** without added $PPh_3$

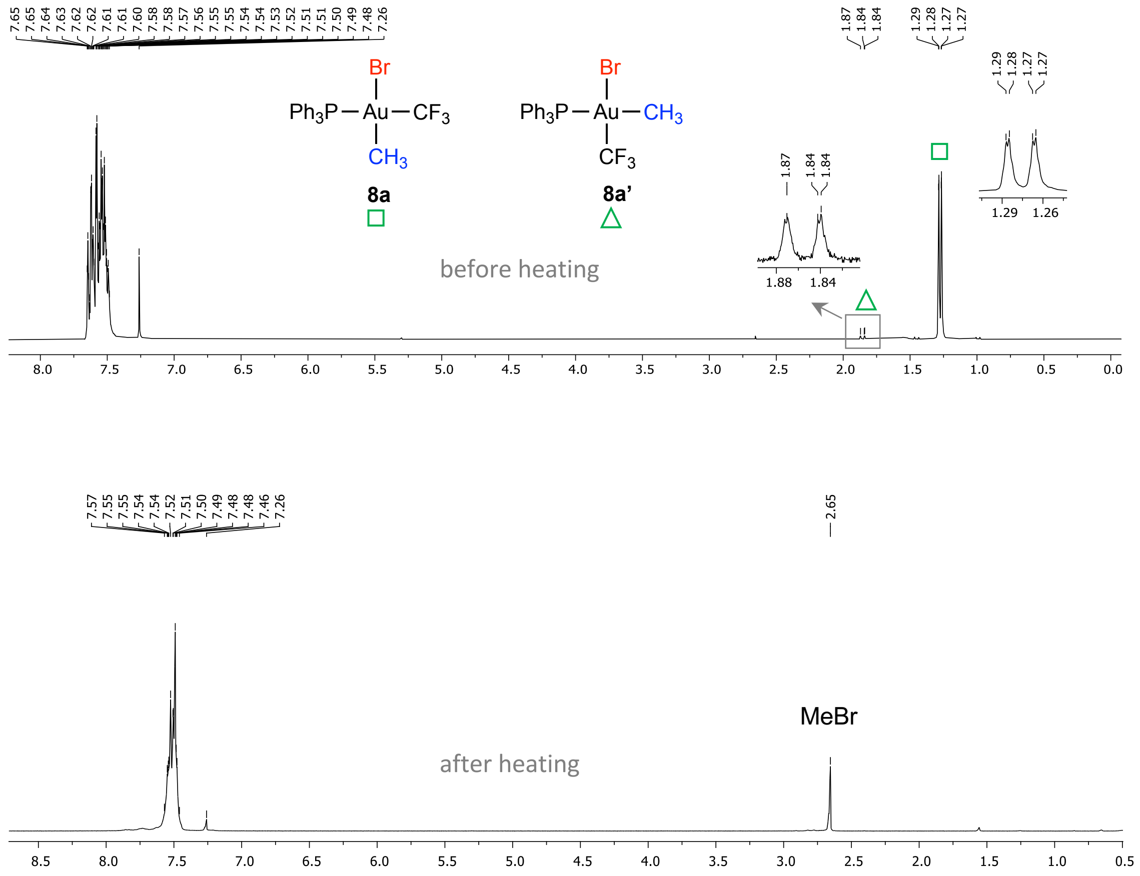


**Figure S80**. $^{1}H$ NMR spectrum (300.1 MHz, $CDCl_3$) of a mixture of **8a** and **8a'** before and after heating for 4.3 h at 80 ºC.

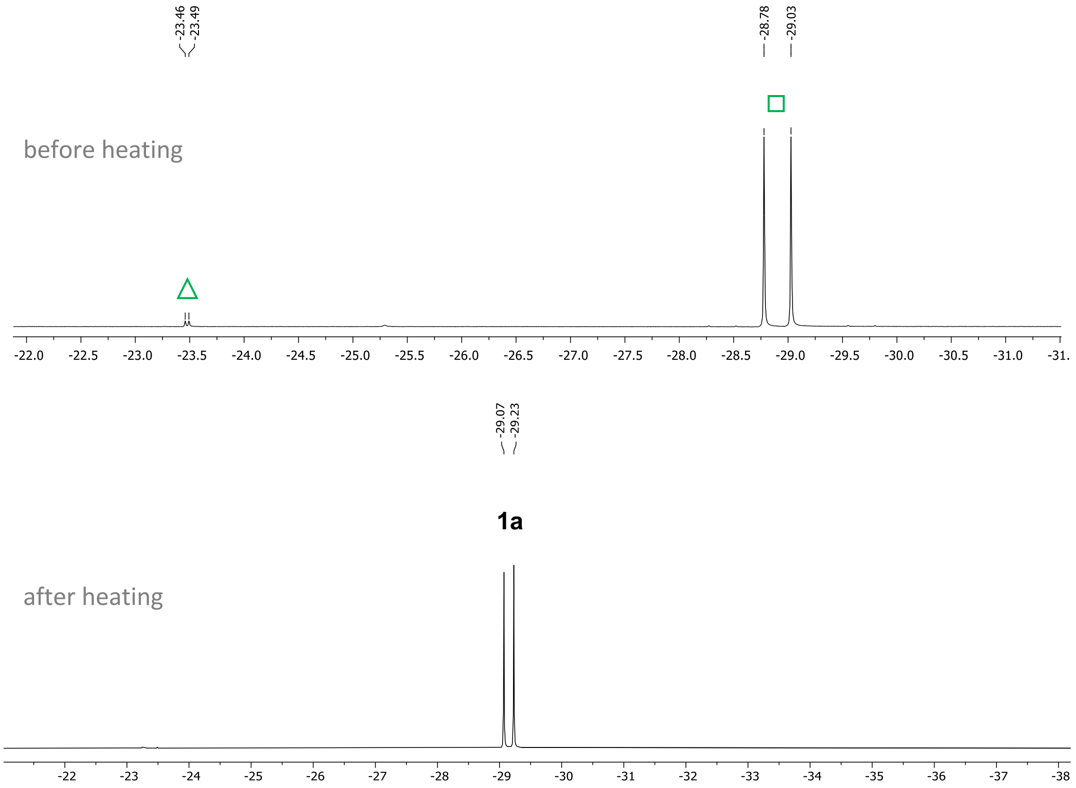


**Figure S81**. $^{19}$F NMR spectrum (282.4 MHz, $CDCl_3$) of a mixture of **8a** and **8a'** before and after heating for 4.3 h at 80 ºC.

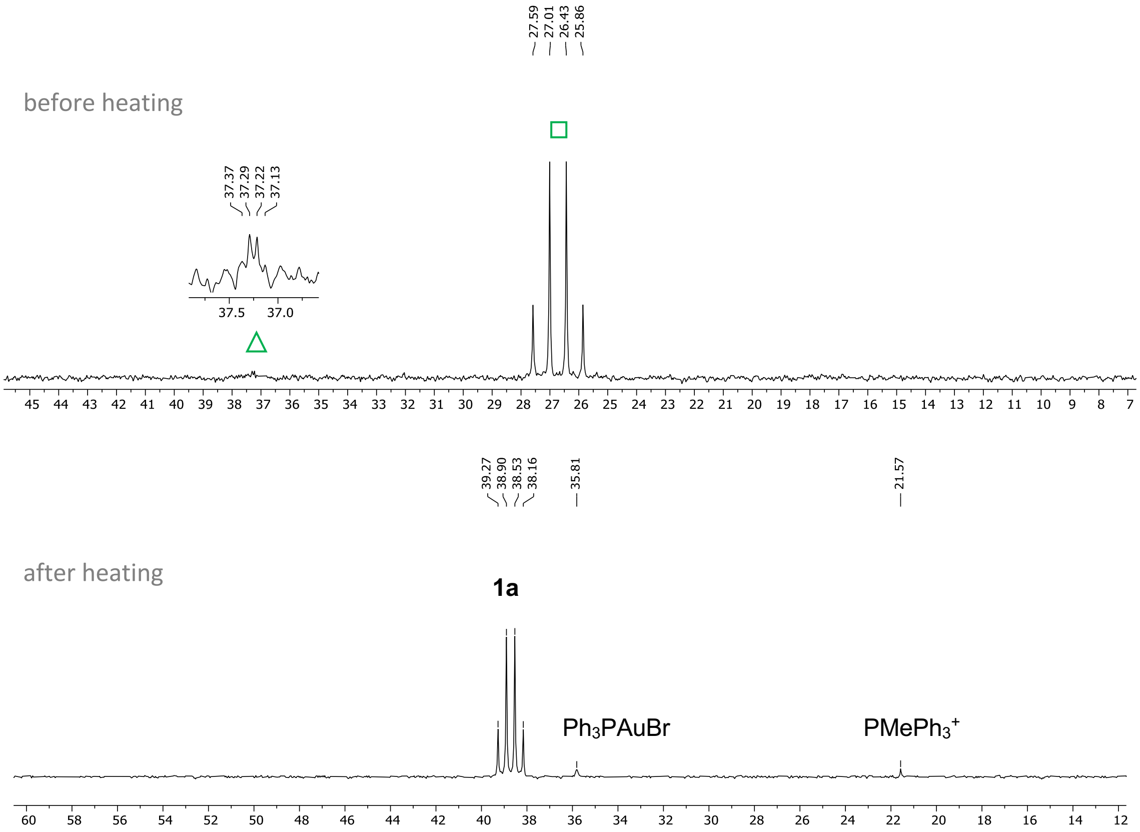


**Figure S82**. $^{31}$P{$^{1}$H} NMR spectrum (121.5 MHz, $CDCl_3$) of a mixture of **8a** and **8a'** before and after heating for 4.3 h at 80 ºC.

b) Reductive elimination of MeBr from **8a** and **8a'** with added $PPh_3$

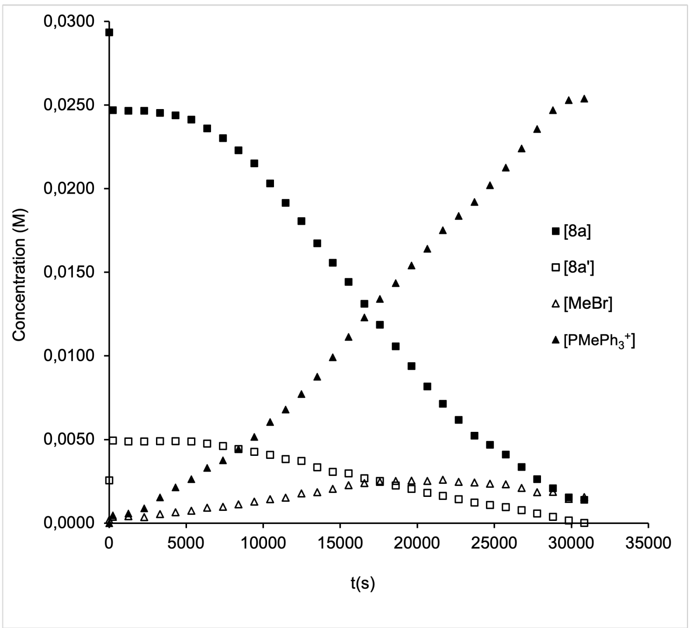


**Figure S83**. Thermal decomposition (80 ºC, $CDCl_3$) of a mixture of **8a** and **8a'** and $PPh_3$ (1 equiv). The decrease in [MeBr] observed at the end of the reactions is attributed to the reaction of MeBr with free $PPh_3$. Concentrations were determined by integration of the $^1$H-NMR spectra using 1,3,5-trimethoxybenzene as internal standard.

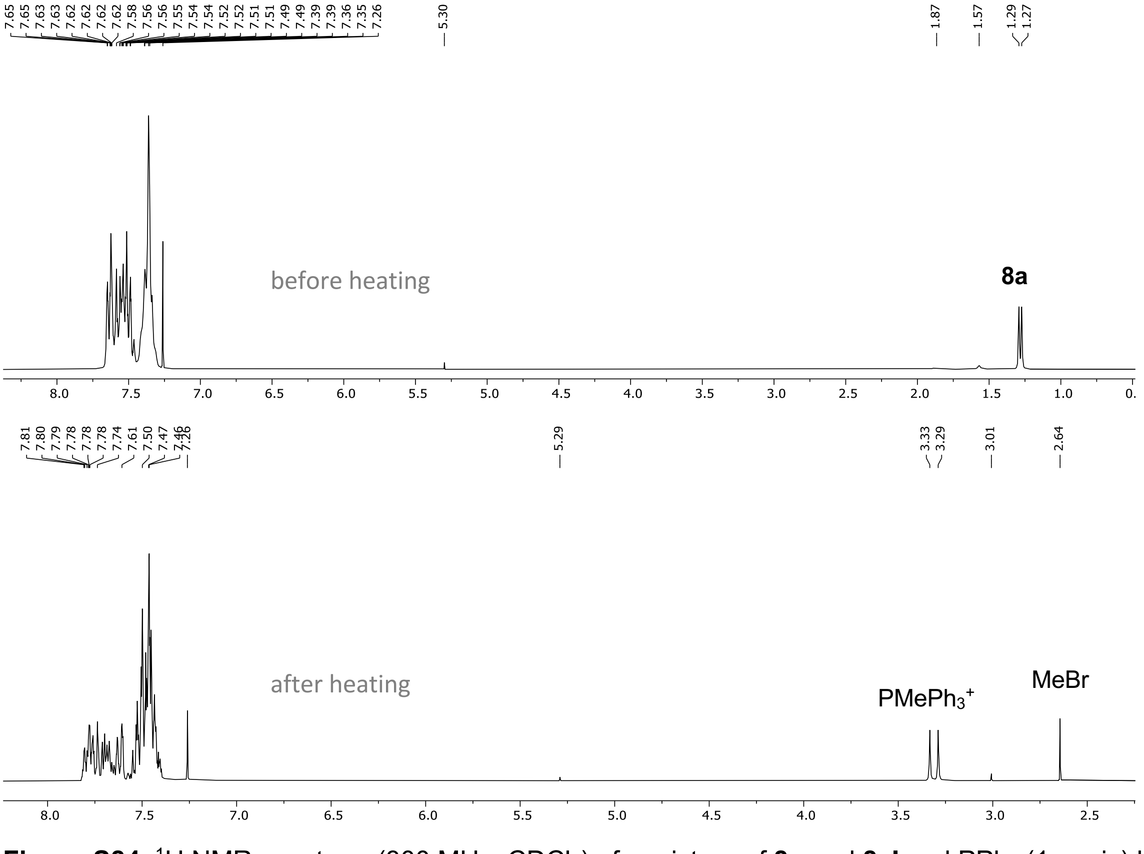


**Figure S84**. $^1$H NMR spectrum (300 MHz, $CDCl_3$) of a mixture of **8a** and **8a'** and $PPh_3$ (1 equiv) before and after heating for 8.6 h at 80 ºC.

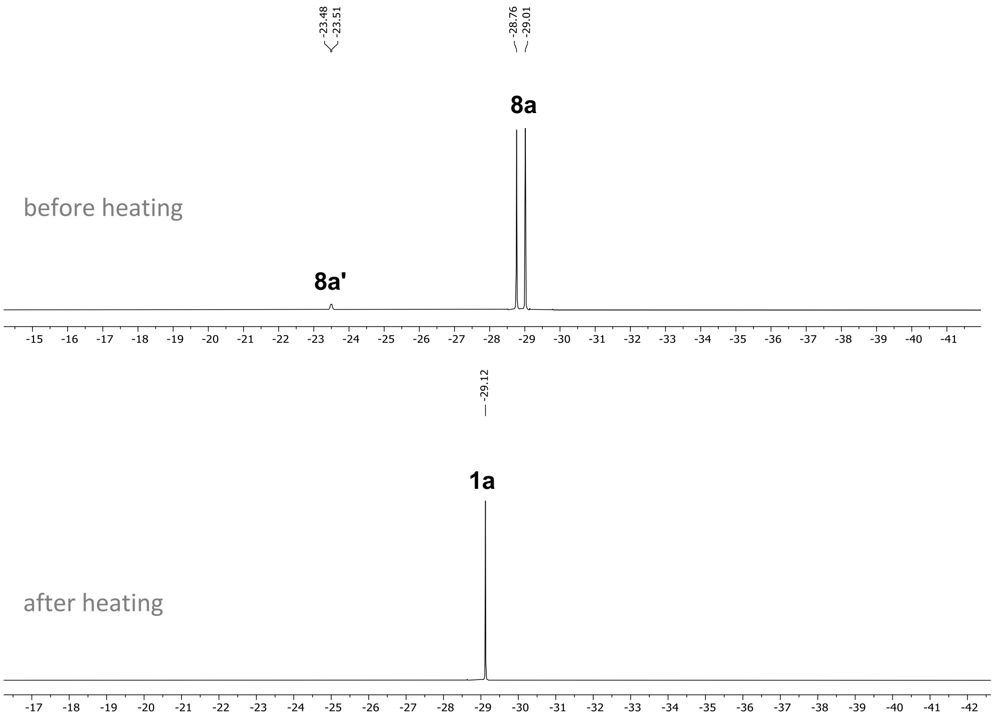


**Figure S85**. $^{19}F$ NMR spectrum (282.4 MHz, $CDCl_3$) of a mixture of **8a**, **8a'** and $PPh_3$ (1 equiv) before and after heating for 8.6 h at 80 ºC.

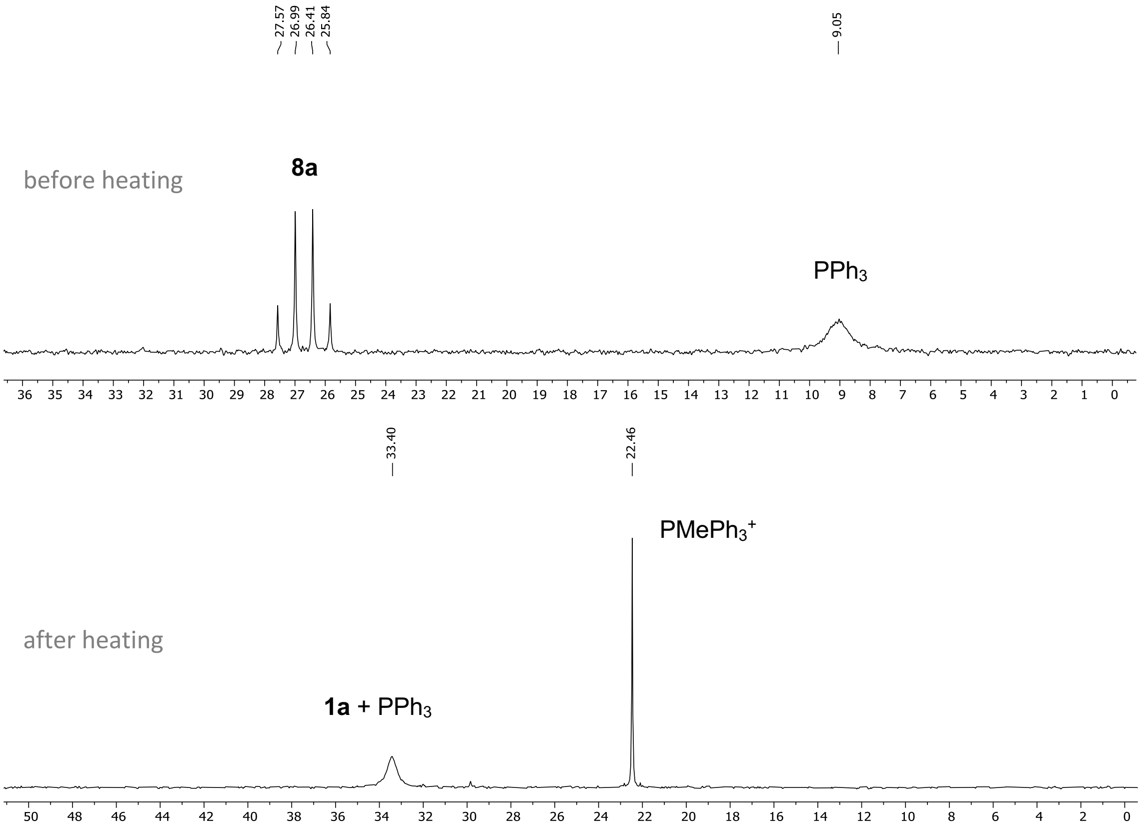


**Figure S86**. $^{31}P\{^1H\}$ NMR spectrum (121.5 MHz, $CDCl_3$) of a mixture of **8a**, **8a'** and $PPh_3$ (1 equiv) before and after heating for 8.6 h at 80 ºC. The $Ph_3P$ signal in the initial spectrum is broad and shifted respect to that of free $PPh_3$ (-6 ppm).

c) Reductive elimination of MeBr from **8b**

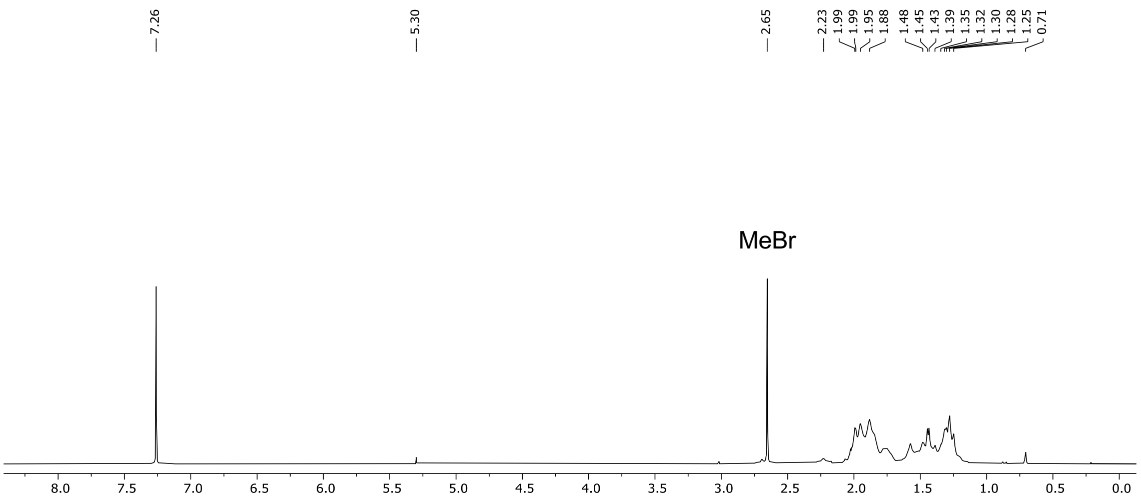


**Figure S87**. $^{1}H$ NMR spectrum (300.1 MHz, $CDCl_3$) of [AuBr($CF_3$)($CH_3$)Br($PCy_3$)] (**8b**) after heating for 20 h at 110 ºC.

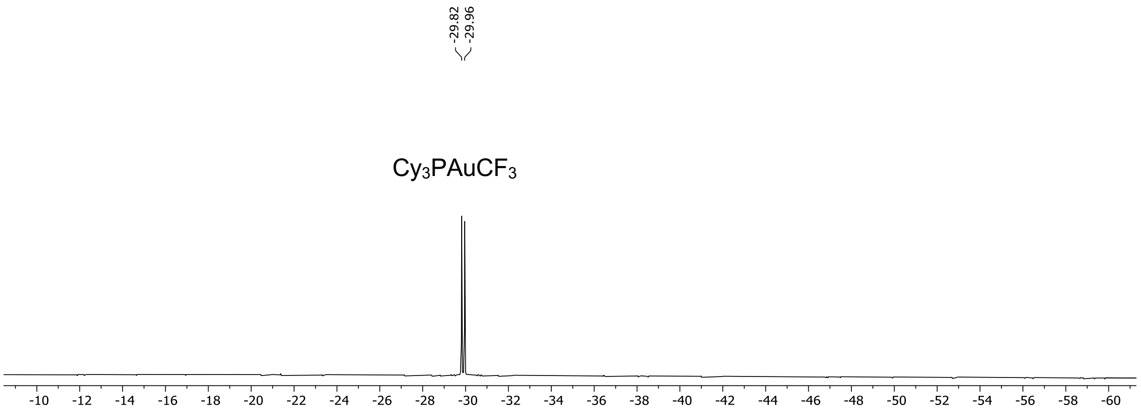


**Figure S88**. $^{19}F$ NMR spectrum (282.4 MHz, $CDCl_3$) of [Au($CF_3$)($CH_3$)Br($PCy_3$)] (**8b**) after heating for 20 h at 110 ºC.

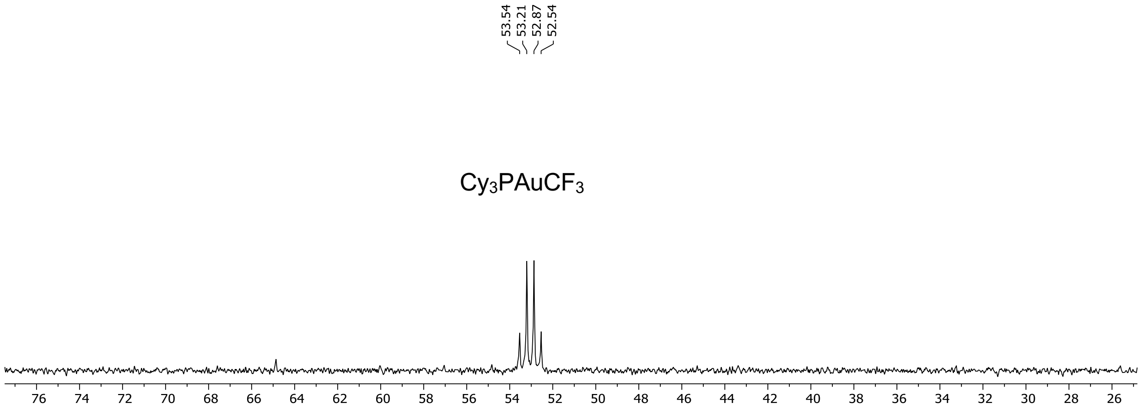


**Figure S89**. $^{31}P\{^{1}H\}$ NMR spectrum (121.5 MHz, $CDCl_3$) of [Au($CF_3$)($CH_3$)Br($PCy_3$)] (**8b**) after heating for 20 h at 110 ºC.

### 3.8. Reductive elimination of MeCl from 9a and 9a'

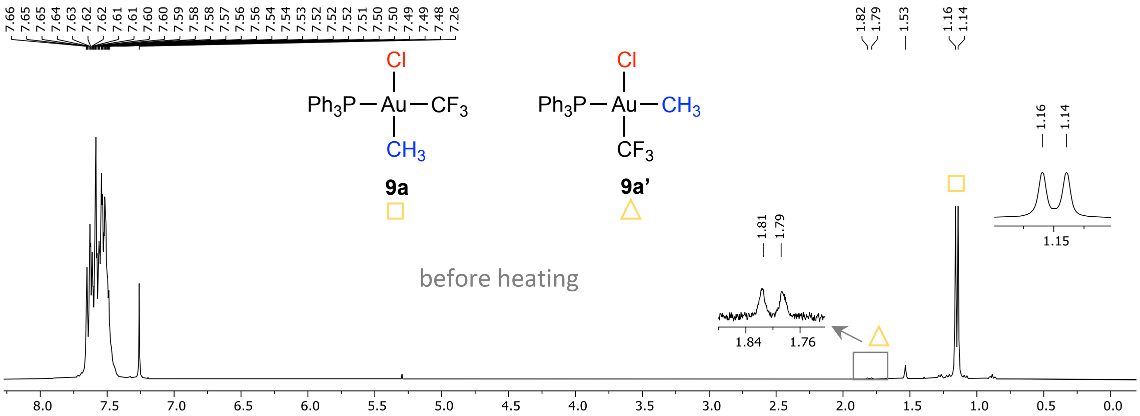


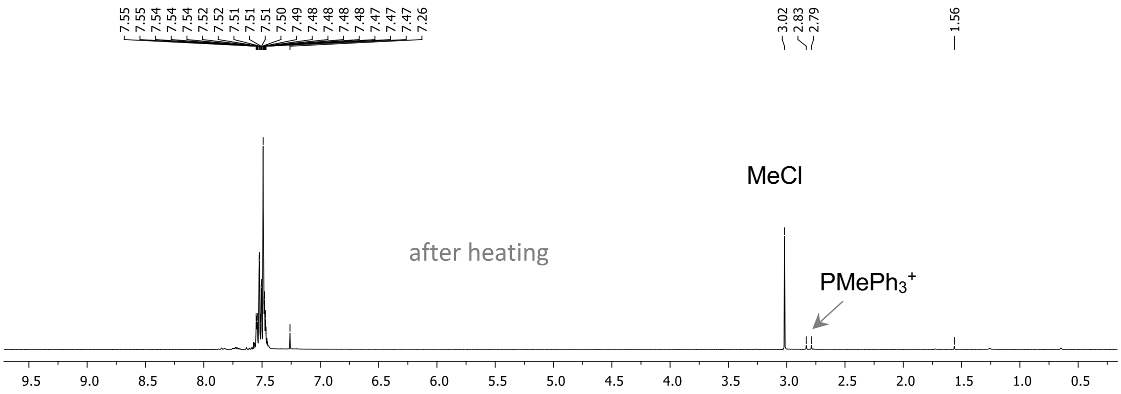


**Figure S90**. $^{1}$H NMR spectrum (300.1 MHz, $CDCl_3$) of **9a** and **9a'** before and after heating for 5 h at 80 ºC.

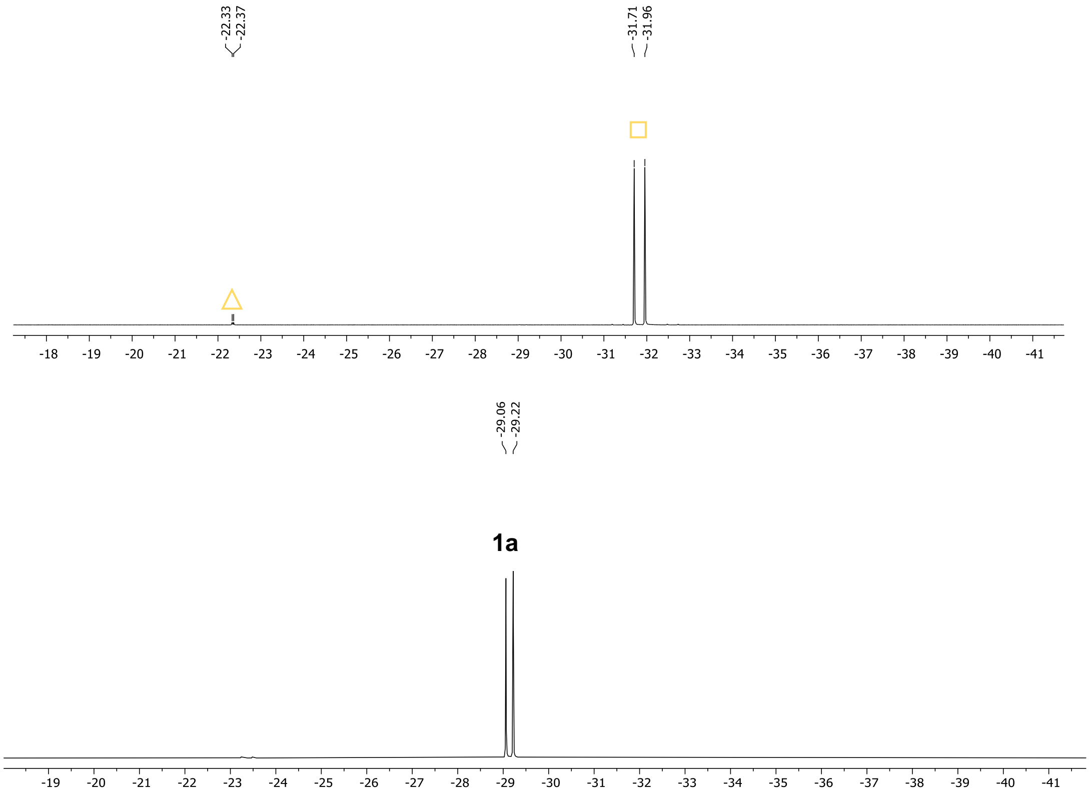


**Figure S91**. $^{19}F$ NMR spectrum (282.4 MHz, $CDCl_3$) of **9a** and **9a'** before and after heating for 5 h at 80 ºC.

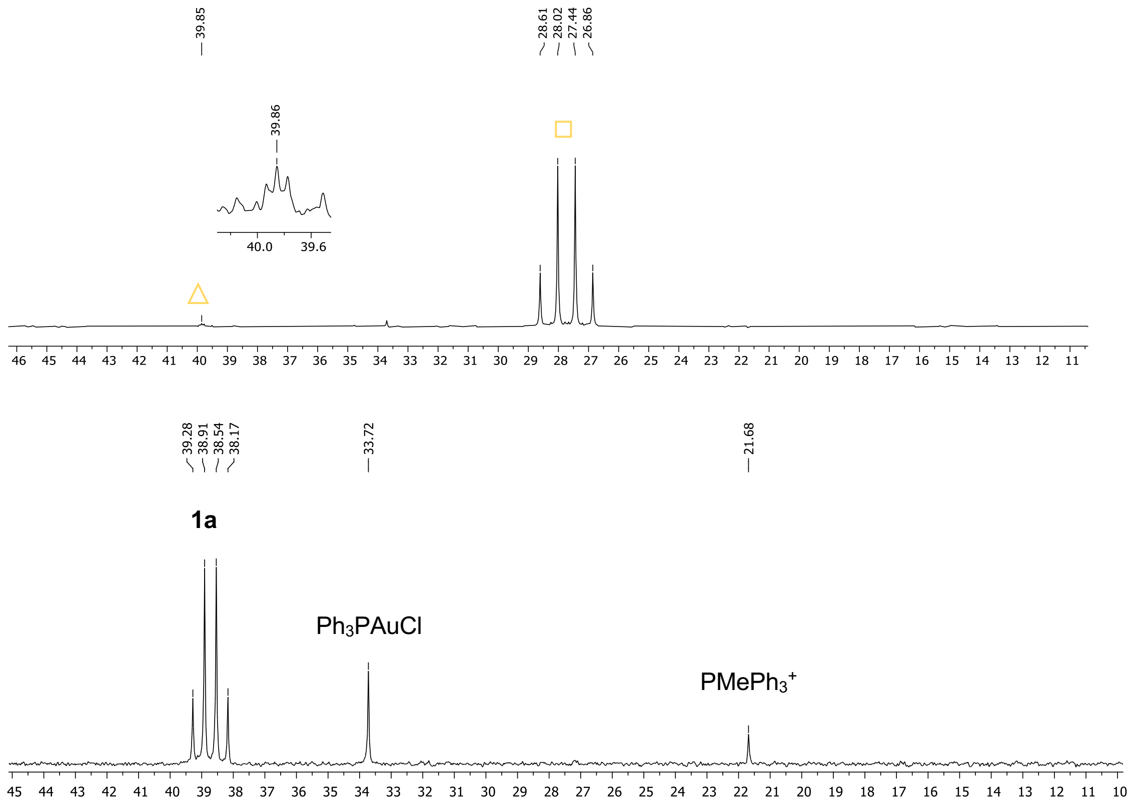


**Figure S92**. $^{31}P\{^1H\}$ NMR spectrum (121.5 MHz, $CDCl_3$) of **9a** and **9a'** before and after heating for 5 h at 80 ºC.

### 3.9. Reductive elimination of MeF from 10a and 10a'

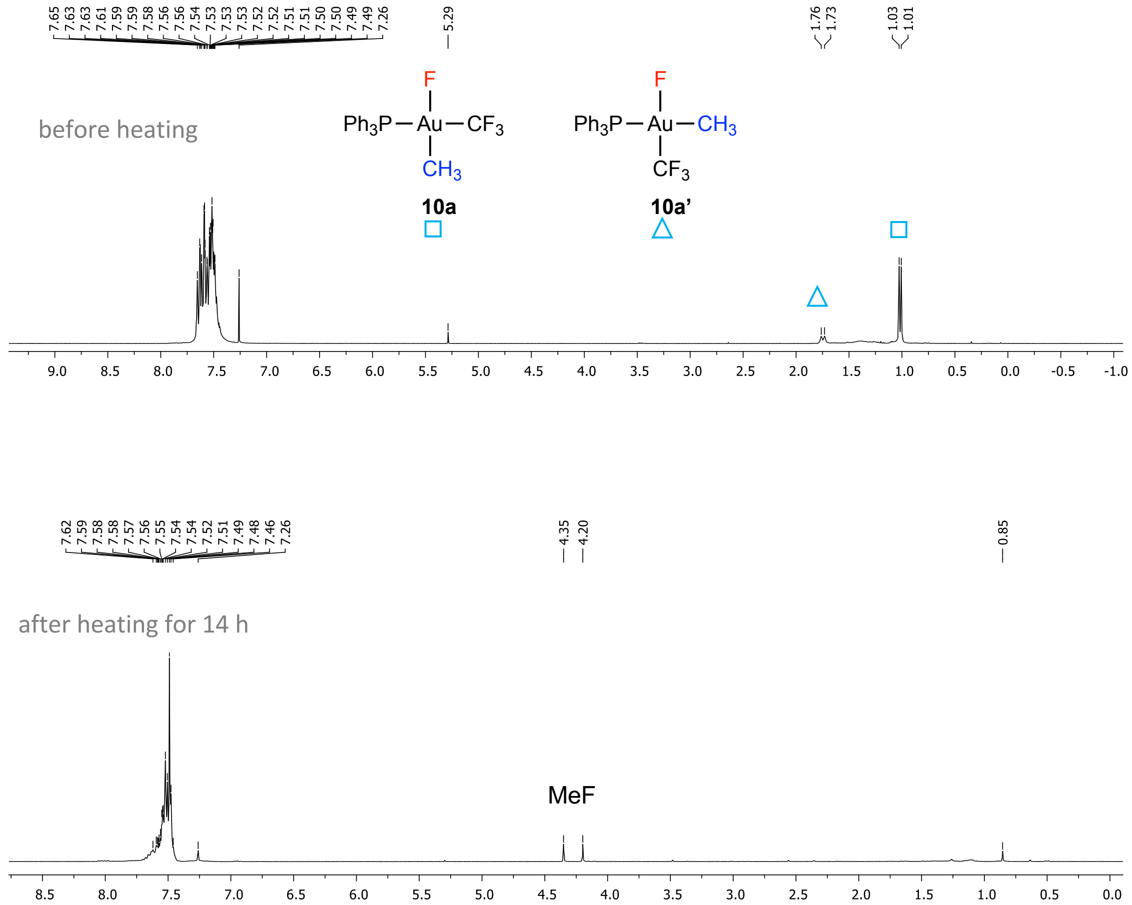


**Figure S93**. $^1$H NMR spectra (300.1 MHz, $CDCl_3$) of an in situ generated mixture of **10a** and **10a'** before and after heating at 110 ºC in a FEP tube with a PTFE stopper.

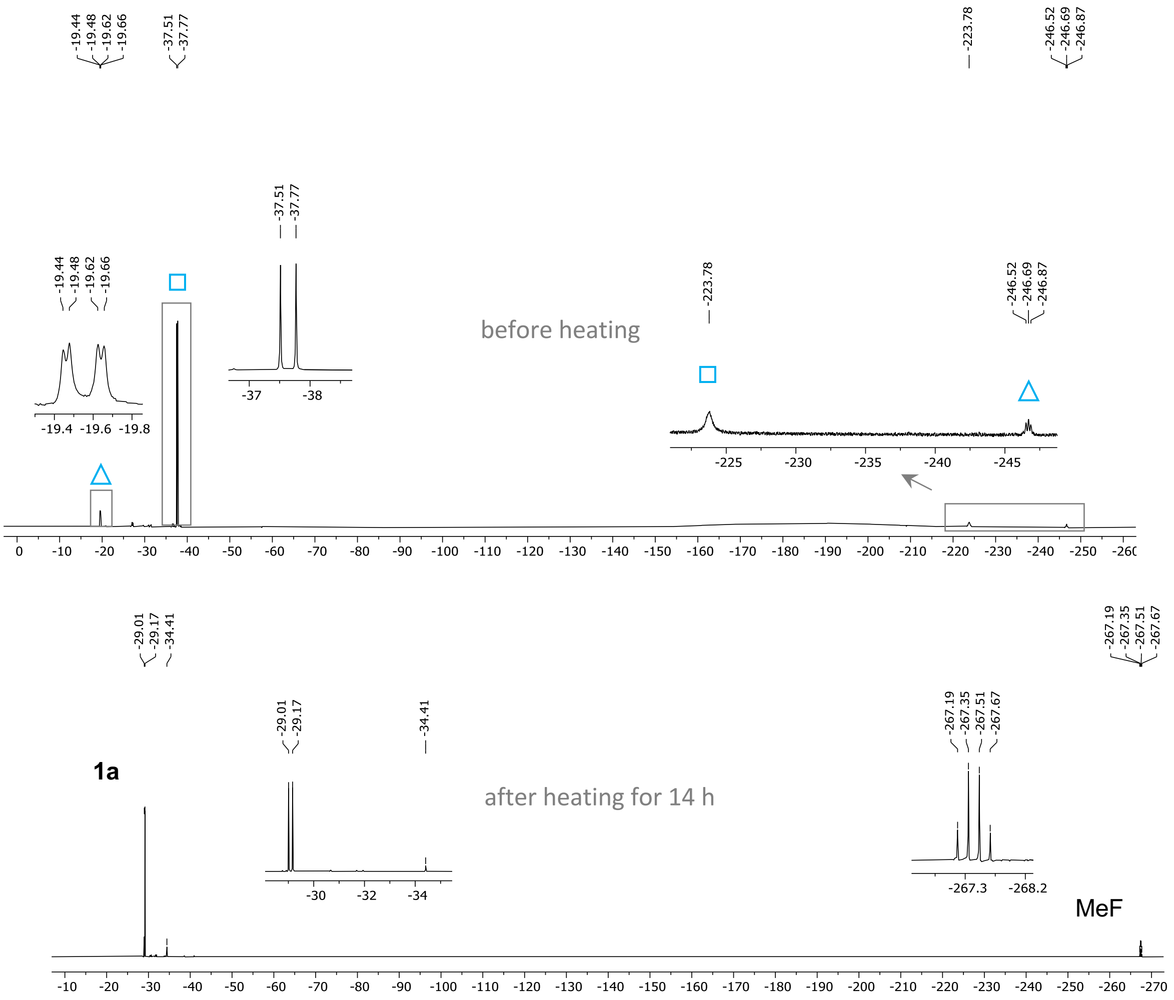


**Figure S94**. $^{19}F$ NMR spectrum (282.4 MHz, $CDCl_3$) of an in situ generated mixture of **10a** and **10a'** before and after heating at 110 ºC in a FEP tube with a PTFE stopper.

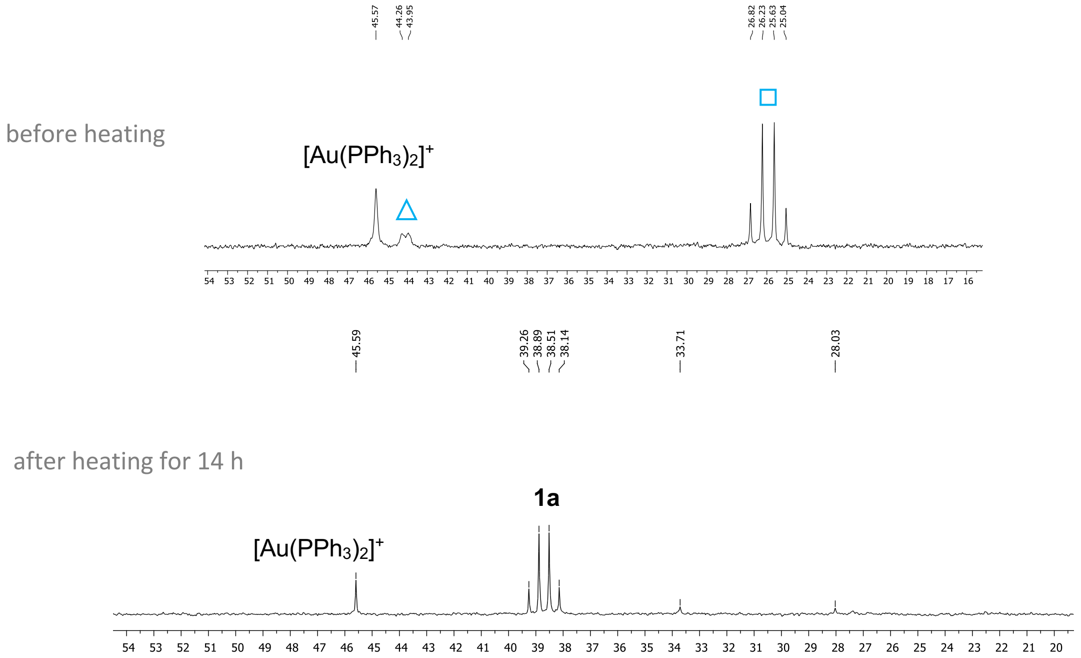


**Figure S95**. $^{31}P\{^1H\}$ NMR spectrum (121.5 MHz, $CDCl_3$) of an in situ generated mixture of **10a** and **10a'** before and after heating at 110 ºC in a FEP tube with a PTFE stopper.

**3.10. Reductive elimination of $(PMePh_3)^+(OTf)^-$ from 13**

a) Reductive elimination without added $PPh_3$.

The decay of [**13**] shows a first order dependence up to a conversion of 85%, which is in agreement with the following mechanism, by assuming a steady-state approximation, where the intermediate **4a** does not accumulate at an appreciable level compared to **13** or **1a**.

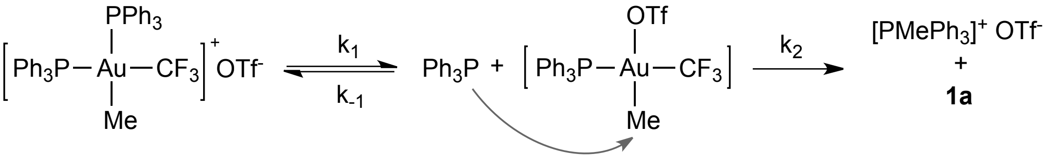


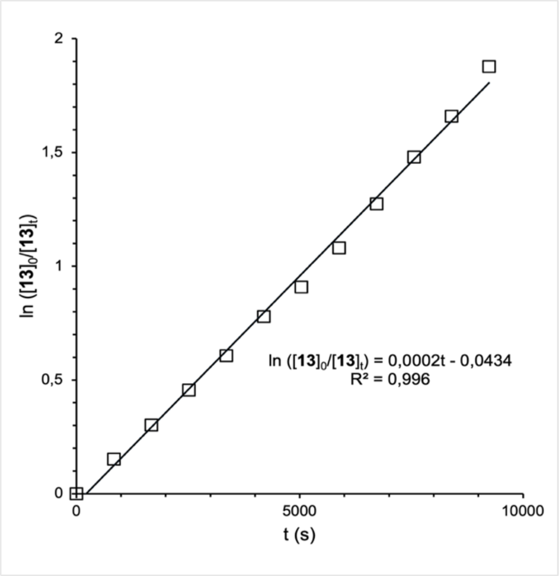


**Figure S96**. First-order dependence of the decay of complex **13**.

$$\frac{d[\mathbf{13}]}{dt} = -k_1[\mathbf{13}] + k_{-1}[\mathbf{4a}][PPh_3] \qquad S1$$

$$\frac{d[\mathbf{4a}]}{dt} = k_1[\mathbf{13}] - k_{-1}[\mathbf{4a}][PPh_3] - k_2[\mathbf{4a}][PPh_3] \qquad S2$$

$$\frac{d[\mathbf{1a}]}{dt} = k_2[\mathbf{4a}][PPh_3] \qquad S3$$

In the steady state region:

$$\frac{d[\mathbf{4a}]}{dt} = 0. \qquad S4$$

$$[\mathbf{4a}]_{SS} = \frac{k_1[\mathbf{13}]}{k_{-1} + k_2[PPh_3]} \qquad S5$$

Then,

$$\frac{d[\mathbf{13}]}{dt} = -k_1[\mathbf{13}] + k_{-1}[\mathbf{4a}]_{SS}[PPh_3] = \frac{-k_1k_2[\mathbf{13}]}{k_{-1} + k_2} \qquad S6$$

$$\frac{d[\mathbf{13}]}{dt} = -k_{SS}[\mathbf{13}] \qquad S7$$

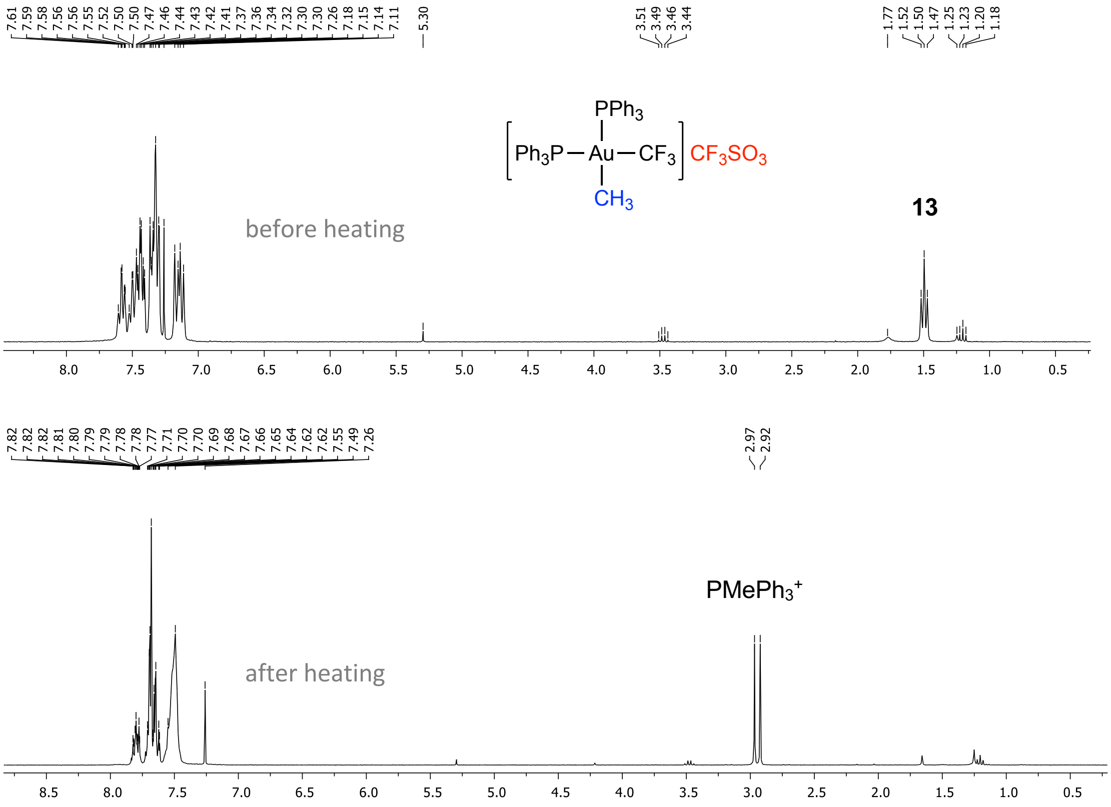


**Figure S97**. $^{1}H$ (300.1 MHz) NMR spectra of a solution of **13** in $CDCl_3$ before and after heating at 60 ºC for 4 h.

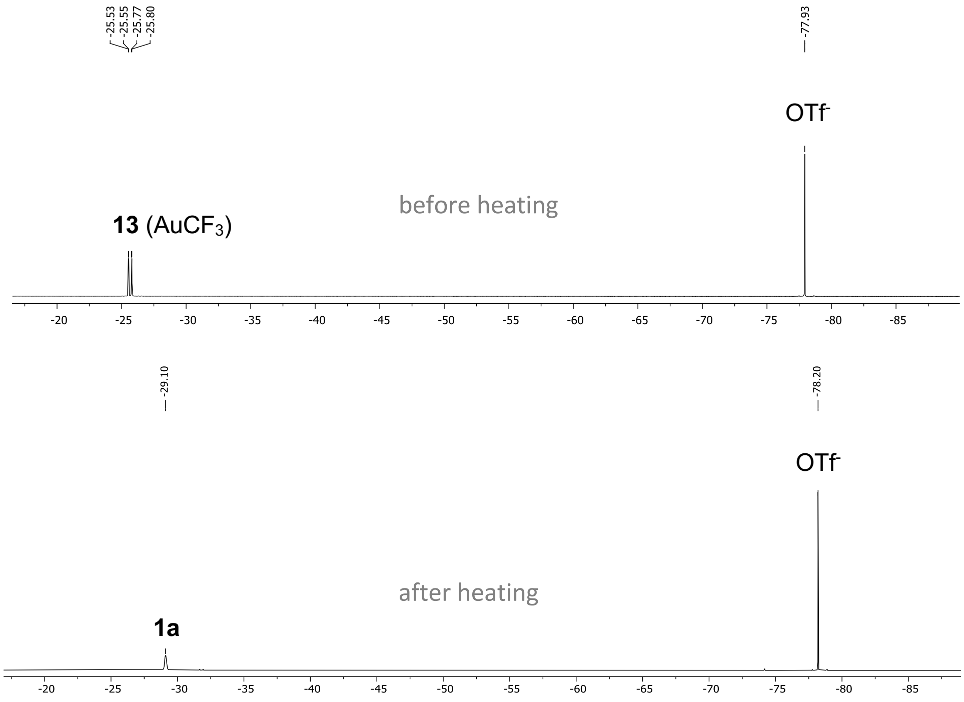


**Figure S98**. $^{19}F$ (282.4 MHz) NMR spectra of a solution of **13** in $CDCl_3$ before and after heating at 60 ºC for 4 h. The signal of **1a** is broad and does not show P-F coupling because of fast exchange with small amounts of free phosphine.

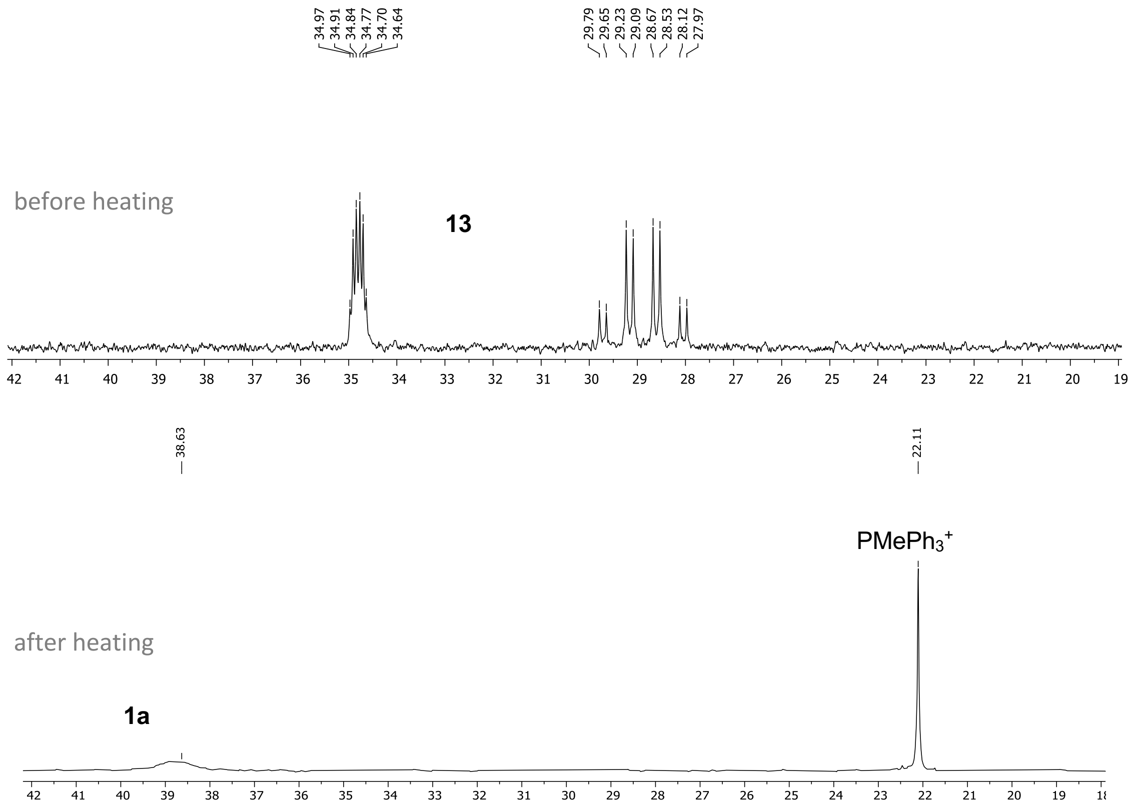


**Figure S99**. $^{31}P\{^{1}H\}$ (121.5 MHz) NMR spectra of a solution of **13** in $CDCl_3$ before (up) and after (down) heating at 60 ºC for 4 h. The signal of **1a** is broad and does not show P-F coupling because of fast exchange with small amounts of free phosphine.

b) $^{31}P\{^1H\}$ NMR spectra of **13** at 60 ºC showing the effect of fast phosphine dissociation

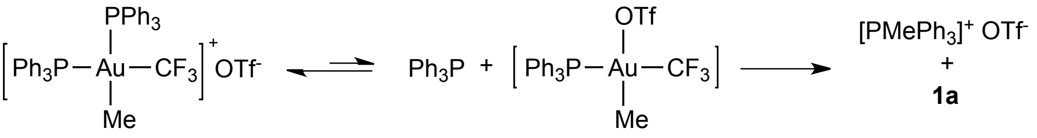

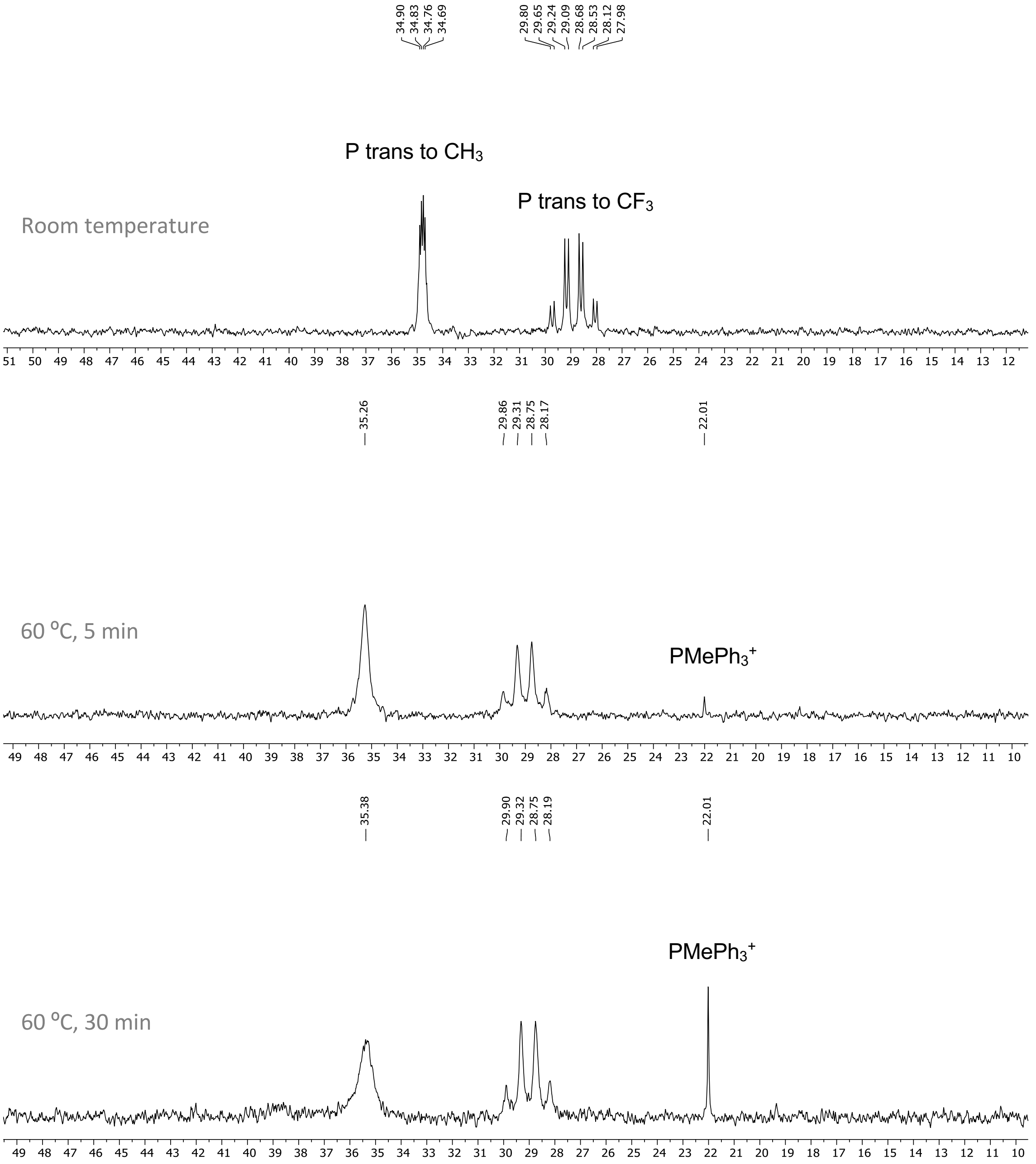


**Figure S100**. $^{31}P\{^1H\}$ NMR spectrum (121.5 MHz, $CDCl_3$) of **13** at room or 60 ºC. The lack of P-P coupling and broadening of the signals suggest fast $PPh_3$ dissociation at 60 ºC in the NMR time-scale. The dissociated phosphine reacts with **13** to give $PMePh_3^+$.

c) Reductive elimination with added $PPh_3$

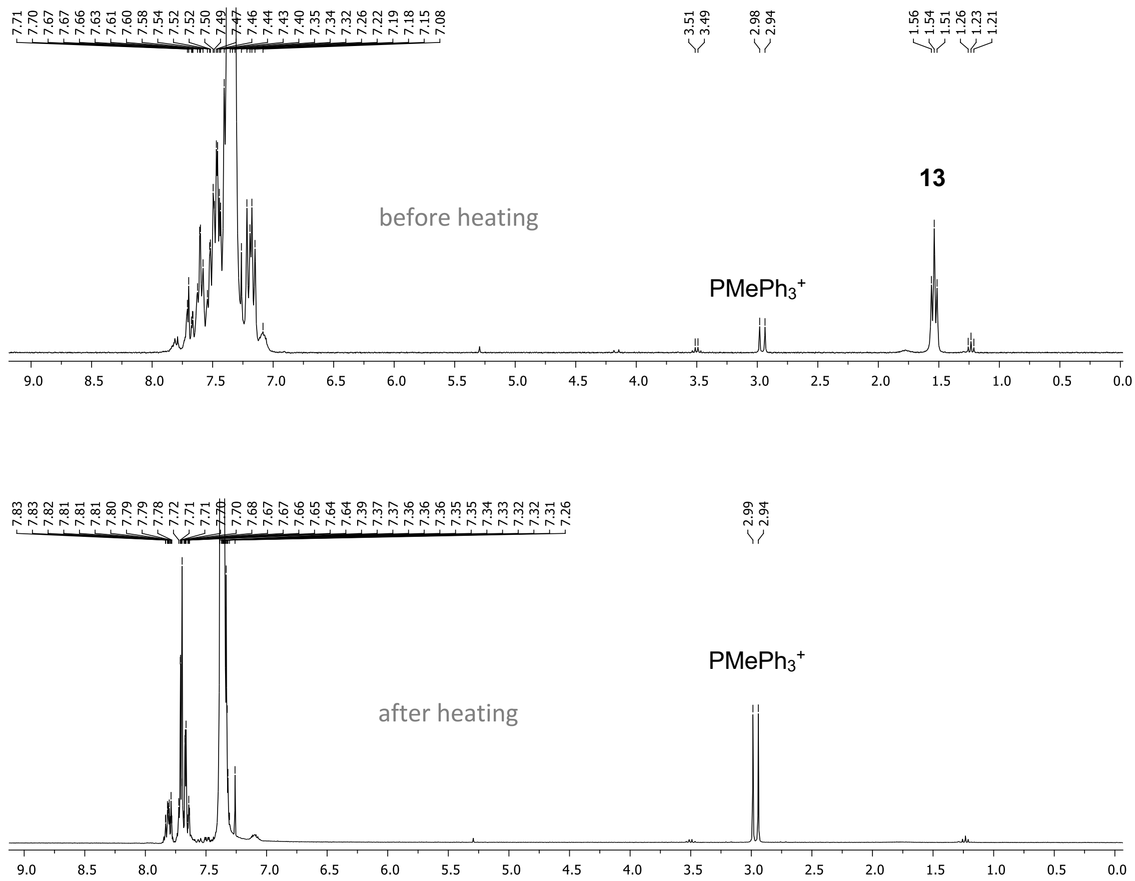


**Figure S101**. $^1$H (300.1 MHz) NMR spectra of a solution of **13** and 5 equiv of $PPh_3$, in $CDCl_3$ before and after heating at 60 ºC for 15 min.

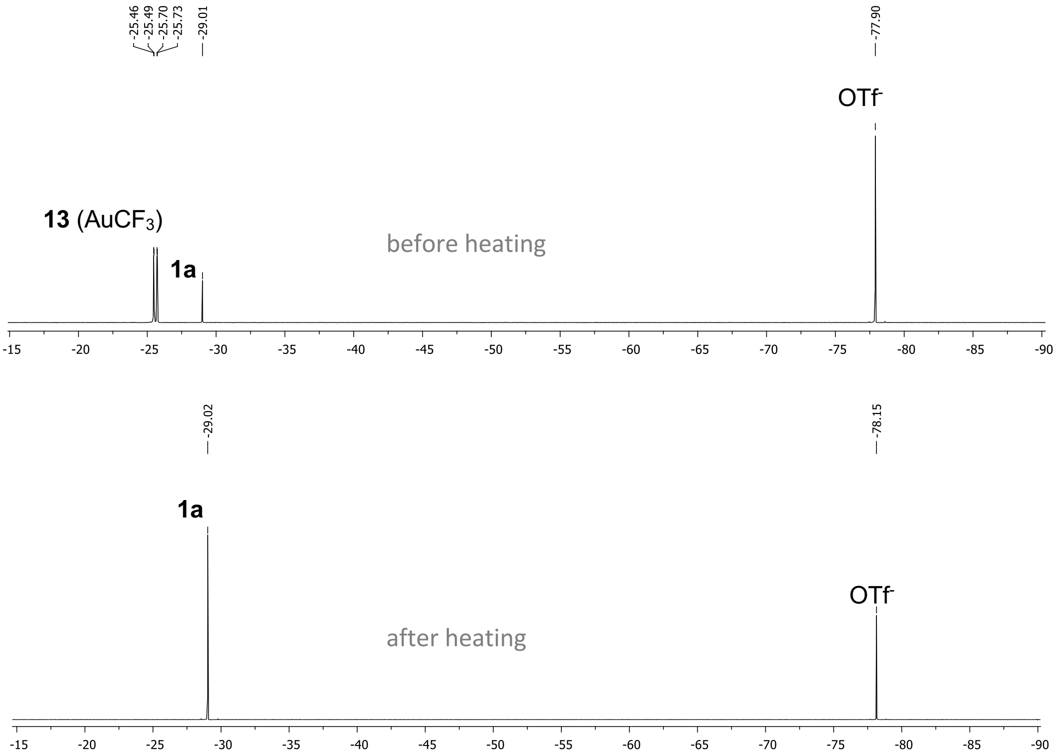


**Figure S102**. $^{19}F$ (282.4 MHz) NMR spectra of a solution of **13** and 5 equiv of $PPh_3$, in $CDCl_3$ before and after heating at 60 ºC for 15 min.

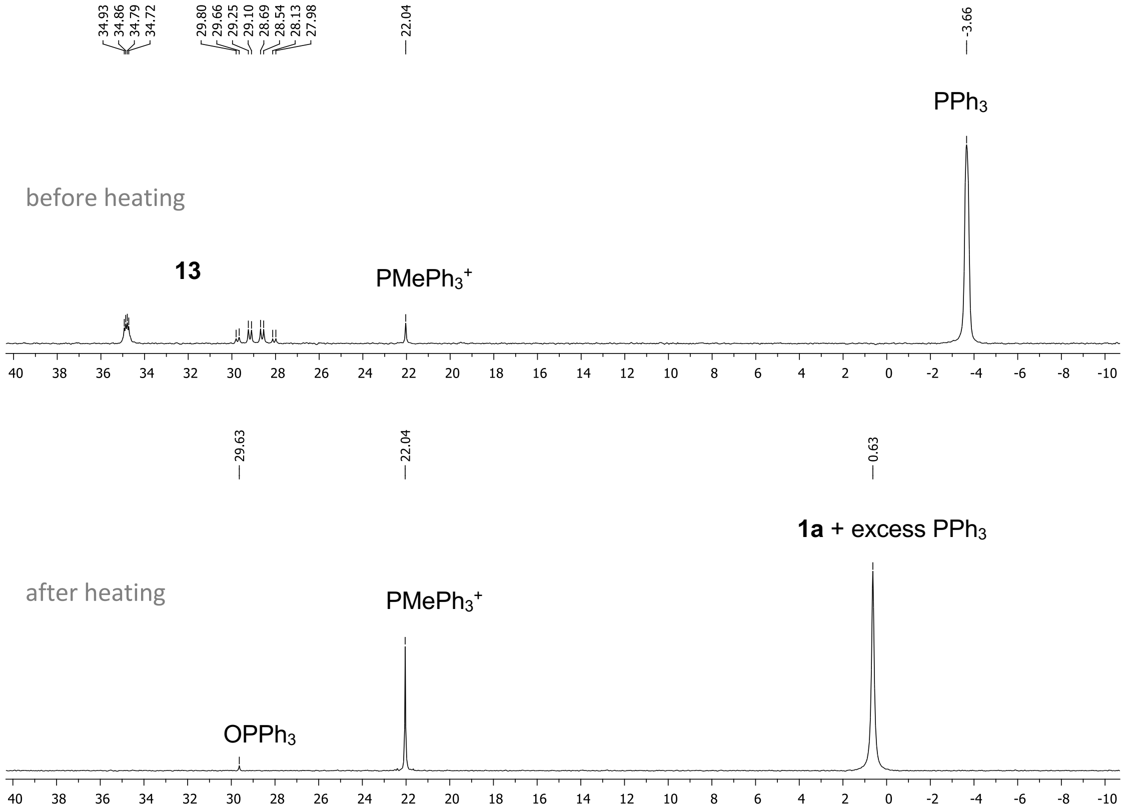


**Figure S103**. $^{31}P\{^1H\}$ (121.5 MHz) NMR spectra of a solution of **13** and 5 equiv of $PPh_3$, in $CDCl_3$ before and after heating at 60 ºC for 15 min.

### 3.11. Reaction of 13 with $NEt_3$. Formation of $(NMeEt_3)^+(OTf)^-$

A mixture of **13** with 2 equiv of $NEt_3$ was heated at 50 ºC for 3 h. The resonances of $(NMeEt_3)^+(OTf)^-$ were assigned by comparison with previously reported $^1$H NMR data.[12]

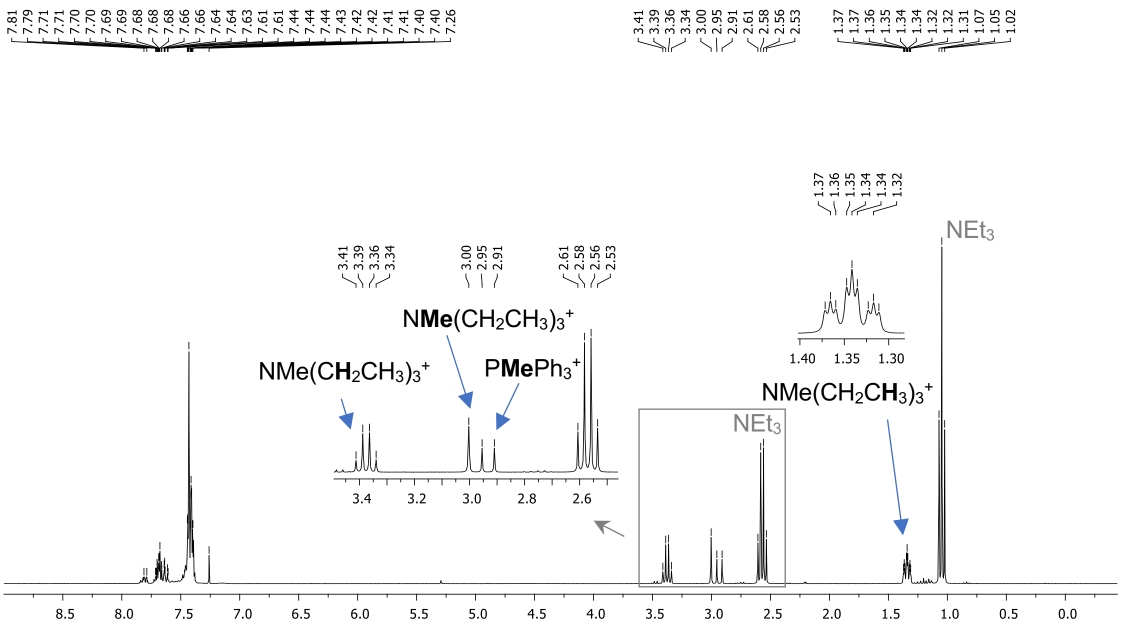


**Figure S104**. $^1$H NMR spectrum (300.1 MHz, $CDCl_3$).

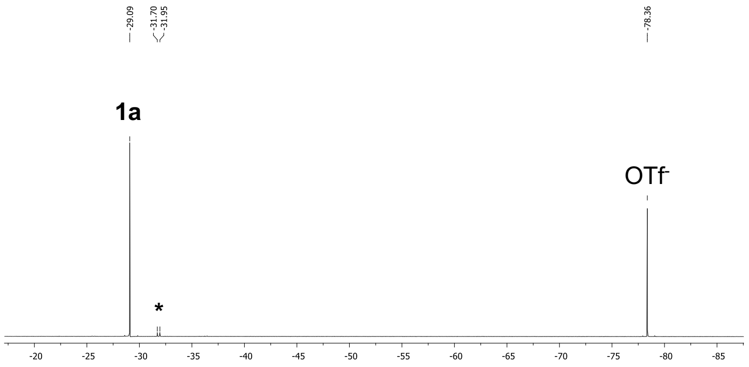


**Figure S105**. $^{19}$F NMR spectrum (282.4 MHz, $CDCl_3$). **1a** does not show P-F coupling because of fast exchange with free phosphine. The signal marked with an asterisk corresponds to an impurity of **9a** present in the starting complex.

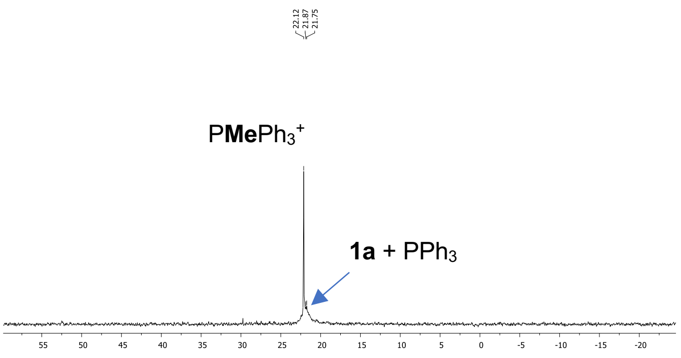


**Figure S106**. $^{31}$P{$^1$H} NMR spectrum (282.4 MHz, $CDCl_3$). The signal of **1a** is broadened and shifted because of fast exchange with free phosphine.

## 4. Crystal structures

4.1 $[Au(OH_2)(CF_3)(Me)(PPh_3)]ClO_4$ (**5a**·$H_2O$)

**Table S2**. Crystal data and structure refinement of **5a**·$H_2O$

| | | |
|---|---|---|
| Empirical formula | $C_{20}H_{20}AuClF_3O_5P$ | |
| Formula weight | 660.75 | |
| Temperature | 100(2) K | |
| Wavelength | 0.71073 Å | |
| Crystal system | Orthorhombic | |
| Space group | Pbca | |
| Unit cell dimensions | *a* = 13.8989(13) Å | α = 90° |
| | *b* = 17.5370(18) Å | β = 90° |
| | *c* = 18.2530(19) Å | γ = 90° |
| Volume | 4449.1(8) $Å^3$ | |
| Z | 8 | |
| Density (calculated) | 1.973 $Mg/m^3$ | |
| Absorption coefficient | 6.859 $mm^{-1}$ | |
| F(000) | 2544 | |
| Crystal size | 0.160 x 0.140 x 0.120 $mm^3$ | |
| Theta range for data collection | 2.177 to 30.534° | |
| Index ranges | $-19 \le h \le 19$, $-25 \le k \le 25$, $-26 \le l \le 26$ | |
| Reflections collected | 106158 | |
| Independent reflections | 6794 [*R*(int) = 0.0596] | |
| Completeness to theta = 30.000° | 100.0 % | |
| Absorption correction | Semi-empirical from equivalents | |
| Max. and min. transmission | 0.7461 and 0.6141 | |
| Refinement method | Full-matrix least-squares on $F^2$ | |
| Data / restraints / parameters | 6794 / 2 / 289 | |
| Goodness-of-fit on $F^2$ | 1.030 | |
| Final *R* indices [*I* > 2sigma(*I*)] | *R1* = 0.0195, *wR2* = 0.0384 | |
| *R* indices (all data) | *R1* = 0.0290, *wR2* = 0.0412 | |
| Largest diff. peak and hole | 0.587 and -0.615 e $Å^{-3}$ | |

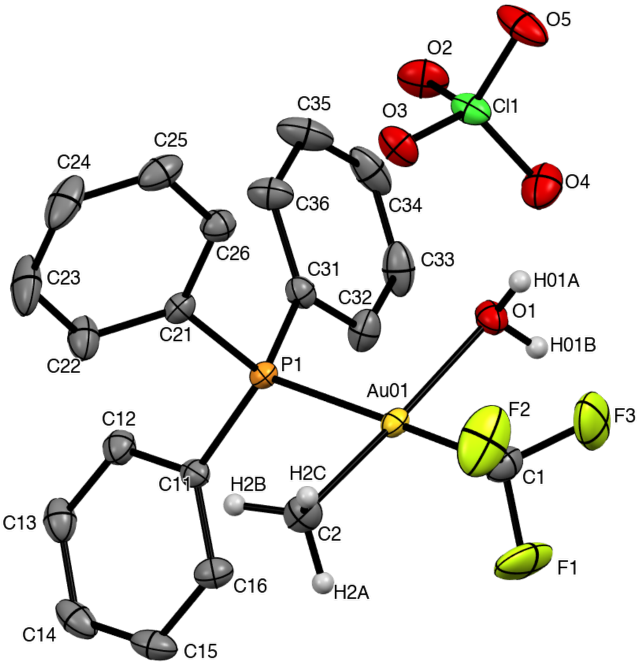


**Figure S107**. ORTEP diagram of the molecular structure of **5a**·$H_2O$ (50 % probability ellipsoids, the phenylic H atoms were omitted for clarity)

**Table S3**. Bond lengths (Å) and angles (°) of **5a**·$H_2O$

| | | | |
|---|---|---|---|
| Au(01)-C(2) | 2.043(2) | F(2)-C(1)-F(3) | 104.4(2) |
| Au(01)-C(1) | 2.070(2) | F(1)-C(1)-Au(01) | 113.46(17) |
| Au(01)-O(1) | 2.1562(17) | F(2)-C(1)-Au(01) | 114.66(17) |
| Au(01)-P(1) | 2.3636(6) | F(3)-C(1)-Au(01) | 112.97(17) |
| C(1)-F(1) | 1.333(3) | C(11)-P(1)-C(21) | 108.15(10) |
| C(1)-F(2) | 1.333(3) | C(11)-P(1)-C(31) | 105.52(10) |
| C(1)-F(3) | 1.348(3) | C(21)-P(1)-C(31) | 107.82(10) |
| P(1)-C(11) | 1.803(2) | C(11)-P(1)-Au(01) | 116.94(7) |
| P(1)-C(21) | 1.804(2) | C(21)-P(1)-Au(01) | 104.20(7) |
| P(1)-C(31) | 1.808(2) | C(31)-P(1)-Au(01) | 113.84(7) |
| C(11)-C(16) | 1.387(3) | C(16)-C(11)-C(12) | 119.5(2) |
| C(11)-C(12) | 1.393(3) | C(16)-C(11)-P(1) | 122.26(18) |
| C(12)-C(13) | 1.389(3) | C(12)-C(11)-P(1) | 118.22(17) |
| C(13)-C(14) | 1.387(4) | C(13)-C(12)-C(11) | 120.3(2) |
| C(14)-C(15) | 1.378(4) | C(14)-C(13)-C(12) | 119.5(2) |
| C(15)-C(16) | 1.383(3) | C(15)-C(14)-C(13) | 120.1(2) |
| C(21)-C(22) | 1.384(3) | C(14)-C(15)-C(16) | 120.6(2) |
| C(21)-C(26) | 1.396(3) | C(15)-C(16)-C(11) | 119.9(2) |
| C(22)-C(23) | 1.384(3) | C(22)-C(21)-C(26) | 119.4(2) |
| C(23)-C(24) | 1.390(4) | C(22)-C(21)-P(1) | 123.55(17) |
| C(24)-C(25) | 1.373(4) | C(26)-C(21)-P(1) | 116.83(17) |
| C(25)-C(26) | 1.377(3) | C(23)-C(22)-C(21) | 119.6(2) |
| C(31)-C(36) | 1.393(3) | C(22)-C(23)-C(24) | 120.5(3) |
| C(31)-C(32) | 1.394(3) | C(25)-C(24)-C(23) | 120.0(2) |
| C(32)-C(33) | 1.389(3) | C(24)-C(25)-C(26) | 119.9(2) |
| C(33)-C(34) | 1.379(4) | C(25)-C(26)-C(21) | 120.6(2) |
| C(34)-C(35) | 1.379(4) | C(36)-C(31)-C(32) | 119.6(2) |
| C(35)-C(36) | 1.382(3) | C(36)-C(31)-P(1) | 123.03(18) |
| Cl(1)-O(2) | 1.4344(18) | C(32)-C(31)-P(1) | 117.34(17) |
| Cl(1)-O(3) | 1.4350(17) | C(33)-C(32)-C(31) | 119.7(2) |
| Cl(1)-O(4) | 1.4414(19) | C(34)-C(33)-C(32) | 120.4(2) |
| Cl(1)-O(5) | 1.4491(18) | C(33)-C(34)-C(35) | 119.7(2) |
| C(2)-Au(01)-C(1) | 85.81(10) | C(34)-C(35)-C(36) | 120.8(3) |
| C(2)-Au(01)-O(1) | 174.86(8) | C(35)-C(36)-C(31) | 119.7(2) |
| C(1)-Au(01)-O(1) | 92.59(9) | O(2)-Cl(1)-O(3) | 109.71(11) |
| C(2)-Au(01)-P(1) | 91.30(7) | O(2)-Cl(1)-O(4) | 109.38(11) |
| C(1)-Au(01)-P(1) | 176.27(7) | O(3)-Cl(1)-O(4) | 109.43(11) |
| O(1)-Au(01)-P(1) | 90.08(5) | O(2)-Cl(1)-O(5) | 109.96(12) |
| F(1)-C(1)-F(2) | 106.2(2) | O(3)-Cl(1)-O(5) | 109.40(10) |
| F(1)-C(1)-F(3) | 104.2(2) | O(4)-Cl(1)-O(5) | 108.94(12) |

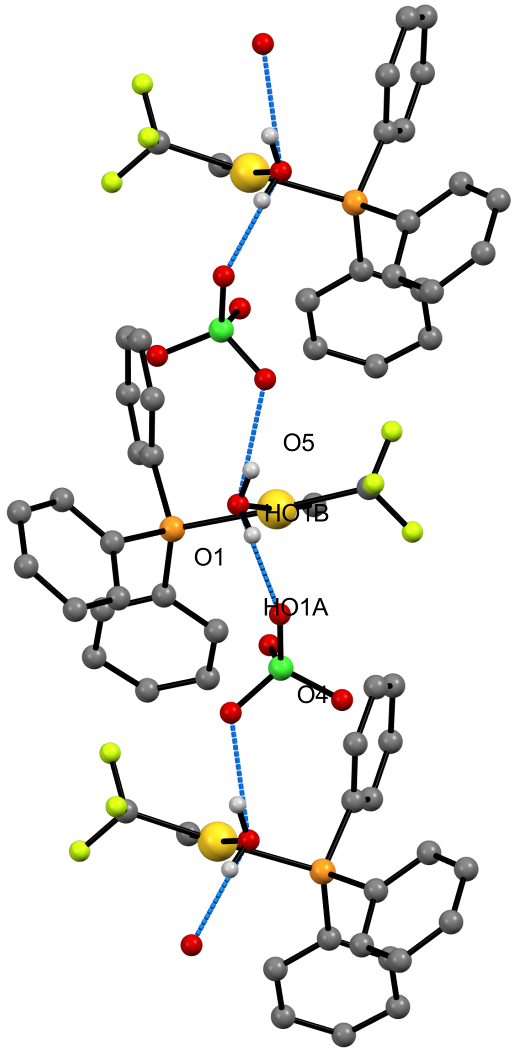


**Figure S108**. Formation of chains by hydrogen bonding between the water hydrogen atoms and the perchlorato oxygen atoms in the structure of **5a**·$H_2O$

**Table S4**. Relevant distances and angles for the hydrogen bonds of **5a**·$H_2O$

| D–H···A | d(D–H) | d(H···A) | d(D···A) | <(DHA) |
|---|---|---|---|---|
| O(1)-H(01A)···O(4) | 0.851(17) | 1.961(19) | 2.783(2) | 162(3) |
| O(1)-H(01B)···O(5)#1 | 0.837(18) | 1.937(19) | 2.768(2) | 172(3) |

Symmetry transformations used to generate equivalent atoms: #1: x+1/2,y,-z+1/2; #2: x,-y+3/2,z+1/2

4.2 Crystal structure of [Au($ONO_2$)($CF_3$)(Me)($PPh_3$)] (**6a**)

**Table S5**. Crystal data and structure refinement of **6a**

| | | |
|---|---|---|
| Empirical formula | $C_{20}H_{18}AuF_3NO_3P$ | |
| Formula weight | 605.29 | |
| Temperature | 100(2) K | |
| Wavelength | 0.71073 Å | |
| Crystal system | Monoclinic | |
| Space group | Cc | |
| Unit cell dimensions | *a* = 19.8305(7) Å | α = 90° |
| | *b* = 7.5696(3) Å | β = = 122.6360(10)° |
| | *c* = 15.2751(5) Å | γ = 90° |
| Volume | 1930.91(12) $Å^3$ | |
| Z | 4 | |
| Density (calculated) | 2.082 $Mg/m^3$ | |
| Absorption coefficient | 7.752 $mm^{-1}$ | |
| F(000) | 1160 | |
| Crystal size | 0.160 x 0.140 x 0.110 $mm^3$ | |
| Theta range for data collection | 2.439 to 30.604°. | |
| Index ranges | $-28 \leq h \leq 28$, $-10 \leq k \leq 10$, $-21 \leq l \leq 21$ | |
| Reflections collected | 41220 | |
| Independent reflections | 5850 [*R*(int) = 0.0240] | |
| Completeness to theta = 30.000° | 100.0 % | |
| Absorption correction | Semi-empirical from equivalents | |
| Max. and min. transmission | 0.7461 and 0.6259 | |
| Refinement method | Full-matrix least-squares on $F^2$ | |
| Data / restraints / parameters | 5850 / 2 / 263 | |
| Goodness-of-fit on $F^2$ | 0.957 | |
| Final *R* indices [*I* > 2sigma(*I*)] | *R1* = 0.0107, *wR2* = 0.0253 | |
| *R* indices (all data) | *R1* = 0.0111, *wR2* = 0.0254 | |
| Absolute structure parameter | 0.0092(18) | |
| Largest diff. peak and hole | 1.294 and -0.464 *e* $Å^{-3}$ | |

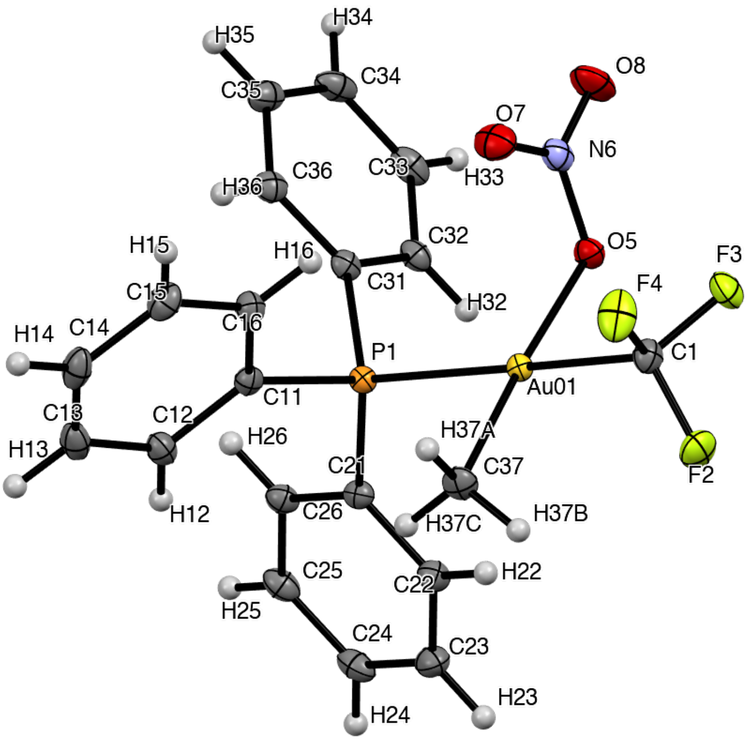


**Figure S109**. ORTEP diagram of the molecular structure of **6a** (50 % probability ellipsoids).

**Table S6**. Bond lengths (Å) and angles (°) of **6a**

| | | | |
|---|---|---|---|
| Au(01)-C(37) | 2.052(3) | F(3)-C(1)-F(4) | 104.8(2) |
| Au(01)-C(1) | 2.075(3) | F(2)-C(1)-Au(01) | 115.69(18) |
| Au(01)-O(5) | 2.133(2) | F(3)-C(1)-Au(01) | 113.91(18) |
| Au(01)-P(1) | 2.3628(7) | F(4)-C(1)-Au(01) | 111.33(18) |
| C(1)-F(2) | 1.349(3) | N(6)-O(5)-Au(01) | 111.73(16) |
| C(1)-F(3) | 1.352(3) | O(8)-N(6)-O(7) | 124.9(3) |
| C(1)-F(4) | 1.362(3) | O(8)-N(6)-O(5) | 116.8(3) |
| O(5)-N(6) | 1.318(3) | O(7)-N(6)-O(5) | 118.4(2) |
| N(6)-O(8) | 1.227(3) | C(11)-P(1)-C(21) | 109.94(13) |
| N(6)-O(7) | 1.232(3) | C(11)-P(1)-C(31) | 106.24(14) |
| P(1)-C(11) | 1.803(3) | C(21)-P(1)-C(31) | 105.13(13) |
| P(1)-C(21) | 1.805(3) | C(11)-P(1)-Au(01) | 108.84(9) |
| P(1)-C(31) | 1.814(3) | C(21)-P(1)-Au(01) | 114.11(9) |
| C(11)-C(12) | 1.394(4) | C(31)-P(1)-Au(01) | 112.26(10) |
| C(11)-C(16) | 1.401(4) | C(12)-C(11)-C(16) | 120.0(3) |
| C(12)-C(13) | 1.400(4) | C(12)-C(11)-P(1) | 123.4(2) |
| C(13)-C(14) | 1.384(4) | C(16)-C(11)-P(1) | 116.6(2) |
| C(14)-C(15) | 1.389(4) | C(11)-C(12)-C(13) | 119.1(3) |
| C(15)-C(16) | 1.389(4) | C(14)-C(13)-C(12) | 120.6(3) |
| C(21)-C(22) | 1.390(4) | C(13)-C(14)-C(15) | 120.3(3) |
| C(21)-C(26) | 1.397(4) | C(16)-C(15)-C(14) | 119.7(3) |
| C(22)-C(23) | 1.389(4) | C(15)-C(16)-C(11) | 120.3(3) |
| C(23)-C(24) | 1.391(5) | C(22)-C(21)-C(26) | 119.8(3) |
| C(24)-C(25) | 1.384(4) | C(22)-C(21)-P(1) | 120.7(2) |
| C(25)-C(26) | 1.393(4) | C(26)-C(21)-P(1) | 119.4(2) |
| C(31)-C(36) | 1.385(4) | C(23)-C(22)-C(21) | 120.1(3) |
| C(31)-C(32) | 1.400(4) | C(22)-C(23)-C(24) | 119.9(3) |
| C(32)-C(33) | 1.392(4) | C(25)-C(24)-C(23) | 120.4(3) |
| C(33)-C(34) | 1.388(5) | C(24)-C(25)-C(26) | 119.9(3) |
| C(34)-C(35) | 1.388(5) | C(25)-C(26)-C(21) | 119.9(3) |
| C(35)-C(36) | 1.399(4) | C(36)-C(31)-C(32) | 120.2(3) |
| C(37)-Au(01)-C(1) | 86.93(11) | C(36)-C(31)-P(1) | 122.2(2) |
| C(37)-Au(01)-O(5) | 176.72(10) | C(32)-C(31)-P(1) | 117.5(2) |
| C(1)-Au(01)-O(5) | 91.66(10) | C(33)-C(32)-C(31) | 119.8(3) |
| C(37)-Au(01)-P(1) | 89.22(8) | C(34)-C(33)-C(32) | 119.9(3) |
| C(1)-Au(01)-P(1) | 174.34(8) | C(35)-C(34)-C(33) | 120.4(3) |
| O(5)-Au(01)-P(1) | 92.39(6) | C(34)-C(35)-C(36) | 119.9(3) |
| F(2)-C(1)-F(3) | 104.9(2) | C(31)-C(36)-C(35) | 119.8(3) |
| F(2)-C(1)-F(4) | 105.2(2) | | |

## 5. Computational methods and data

DFT calculations were carried out with Gaussian 16,[13] using the B3LYP functional[14,15] together with the 6-31G**[16,17] basis set for the C, H, F, and P atoms and the LANL2DZ[18] basis set and effective core potential for the Au atom. Geometry optimizations were performed with no restrictions on symmetry. The solvent effect (chloroform) was accounted for in all cases by using the SMD variation of the Polarizable Continuum Model, as implemented in Gaussian.[19] The optimized structures were confirmed as minima or first-order saddle points (transition states) on the potential energy surface by performing frequency calculations. IRC calculations were performed on transition-state structures to verify that they connect reactant and product.

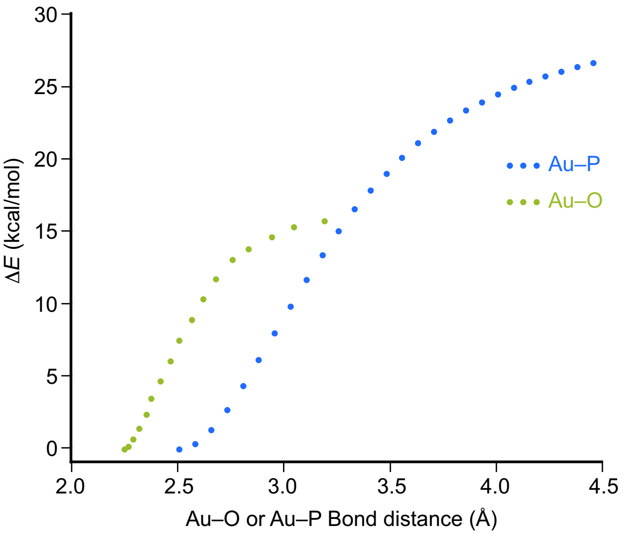


**Fig. S110.** Relaxed potential energy surface (PES) scans along the Au–O or Au–P bond distances of **4a** in $CHCl_3$ solution.

**Table S7.** Energies, free energies, enthalpies and entropies of the optimized structures in $CHCl_3$ solution.[a]

| Structure | $E_0$ [b] | ZPE [c] | G [d] | H [e] | S [f] |
|---|---|---|---|---|---|
| **4a** | -2510.757497 | -2510.402048 | -2510.471925 | -2510.367547 | 219.680 |
| **Int1⁺** | -1549.152898 | -1548.824844 | -1548.882607 | -1548.799417 | 175.089 |
| **OTf⁻** | -961.563223 | -961.536109 | -961.568768 | -961.527953 | 85.902 |
| **Int2⁺** | -1474.397167 | -1474.317649 | -1474.364306 | -1474.300784 | 133.692 |
| **$PPh_3$** | -1036.343082 | -1036.069368 | -1036.116175 | -1036.052464 | 134.091 |
| **TS1A** | -2510.689341 | -2510.336410 | -2510.407275 | -2510.301749 | 222.100 |
| **TS1B** | -2510.728932 | -2510.374924 | -2510.448290 | -2510.340288 | 227.308 |
| **TS2** | -1474.338705 | -1474.260948 | -1474.307870 | -1474.244232 | 133.938 |
| **1a** | -1509.464773 | -1509.176496 | -1509.234508 | -1509.152768 | 172.036 |
| **MeOTf** | -1001.315500 | -1001.248606 | -1001.284486 | -1001.238183 | 97.453 |

[a] Thermal corrections from vibrational calculations at 298.15 K. [b] Electronic energy (Hartrees). [c] Sum of electronic and zero-point energies (Hartrees). [d] Free Energy (Hartrees). [e] Enthalpy (Hartrees). [f] Entropy (cal mol$^{-1}$ K$^{-1}$).

**Table S8.** Cartesian coordinates (Å) of the optimized structures.

**4a**

| | | | |
|---|---|---|---|
| Au | -0.568362316 | 0.069455824 | 0.715887916 |
| C | -0.053499679 | 1.826139928 | -0.290501480 |
| H | -0.931848125 | 2.227593792 | -0.789991654 |
| H | 0.350573215 | 2.516776506 | 0.447468693 |
| H | 0.707843528 | 1.527496043 | -1.011719573 |
| C | 1.442535212 | 0.048596543 | 1.303129393 |
| F | 1.685990000 | -0.928600565 | 2.204282798 |
| F | 1.824522503 | 1.211432908 | 1.898697018 |
| F | 2.293283172 | -0.147967739 | 0.265178239 |
| P | -2.956467294 | 0.296824003 | 0.012517480 |
| C | -4.104767716 | -1.062857558 | 0.444551664 |
| C | -4.895572456 | -1.708065735 | -0.516737543 |
| C | -4.215875517 | -1.429354587 | 1.796490560 |
| C | -5.792487041 | -2.704374201 | -0.125880816 |
| H | -4.816274351 | -1.439801811 | -1.564568921 |
| C | -5.114003976 | -2.424132434 | 2.178234715 |
| H | -3.598555103 | -0.947792204 | 2.548391846 |
| C | -5.903339038 | -3.062858422 | 1.218114475 |
| H | -6.401317320 | -3.200329898 | -0.876208673 |
| H | -5.189872193 | -2.705939438 | 3.224103995 |
| H | -6.598416689 | -3.841800591 | 1.517730336 |
| C | -2.968230793 | 0.476429781 | -1.810549143 |
| C | -3.531330495 | 1.582530329 | -2.462856233 |
| C | -2.318259047 | -0.519139597 | -2.563920774 |
| C | -3.455538162 | 1.685416166 | -3.854393210 |
| H | -4.023894626 | 2.364661367 | -1.895478970 |
| C | -2.255462610 | -0.411793885 | -3.952542567 |
| H | -1.871740433 | -1.375335702 | -2.065326674 |
| C | -2.821572404 | 0.690617088 | -4.599611553 |
| H | -3.892695367 | 2.546159051 | -4.352087800 |
| H | -1.758374050 | -1.187459909 | -4.527658900 |
| H | -2.764459785 | 0.774283996 | -5.681074592 |
| C | -3.726340010 | 1.787618925 | 0.751394773 |
| C | -5.094845786 | 2.050144467 | 0.558884731 |
| C | -2.966962609 | 2.652324342 | 1.553199399 |
| C | -5.680273047 | 3.174139868 | 1.139593615 |
| H | -5.704204612 | 1.375420805 | -0.034693732 |
| C | -3.559954068 | 3.773556343 | 2.137658631 |
| H | -1.916383498 | 2.445129970 | 1.728670770 |
| C | -4.913740821 | 4.037860545 | 1.927630494 |
| H | -6.736810404 | 3.370926335 | 0.983021576 |
| H | -2.963122772 | 4.434159593 | 2.759521069 |
| H | -5.374823790 | 4.909432074 | 2.383166034 |
| O | -0.966169884 | -1.866522465 | 1.773459015 |
| S | -1.040667410 | -3.190693215 | 1.017786341 |
| C | 0.731132110 | -3.759575490 | 1.013066800 |
| F | 1.229256720 | -3.784643050 | 2.252952405 |
| F | 0.799272847 | -4.994161819 | 0.497011021 |
| F | 1.481549593 | -2.943126413 | 0.257866855 |
| O | -1.758280481 | -4.213528130 | 1.784531768 |
| O | -1.364506466 | -3.029079462 | -0.410837492 |

**Int1$^{+}$**

| | | | |
|---|---|---|---|
| Au | -0.691267558 | 0.131327993 | 0.940372012 |
| C | -0.246250173 | 1.917901713 | -0.032893373 |
| C | 1.304163664 | 0.218883262 | 1.623855008 |
| P | -1.393089880 | -1.971225639 | 2.027178482 |
| C | -0.767483000 | -2.105922131 | 3.735608740 |
| C | -0.407814597 | -3.341267728 | 4.296091703 |
| C | -0.656566765 | -0.933468360 | 4.502760364 |
| C | 0.049721852 | -3.398985514 | 5.613441824 |
| H | -0.477400356 | -4.252456414 | 3.711066082 |
| C | -0.204632473 | -1.000508106 | 5.820200107 |
| H | -0.922902426 | 0.028820485 | 4.073736296 |
| C | 0.150130377 | -2.232857047 | 6.375240259 |
| H | 0.329194040 | -4.356571339 | 6.042224676 |
| H | -0.123954125 | -0.091568964 | 6.408677170 |
| H | 0.509049102 | -2.282960676 | 7.398924386 |
| C | -0.857167769 | -3.435915688 | 1.081053635 |
| C | -1.602717171 | -4.626188057 | 1.083318281 |
| C | 0.335838579 | -3.362165678 | 0.343846731 |
| C | -1.147936882 | -5.730552824 | 0.361895069 |
| H | -2.534183040 | -4.690202306 | 1.636231236 |
| C | 0.784662914 | -4.470831745 | -0.373495731 |
| H | 0.906841743 | -2.438241972 | 0.326244971 |
| C | 0.043391035 | -5.654638326 | -0.364192674 |
| H | -1.727132891 | -6.648966292 | 0.364549061 |
| H | 1.707009360 | -4.407775884 | -0.942939707 |
| H | 0.390484169 | -6.516093748 | -0.926999867 |
| C | -3.221439419 | -1.986057694 | 2.082902502 |
| C | -3.931730693 | -2.129277776 | 3.283443884 |
| C | -3.921871406 | -1.811600952 | 0.874476167 |
| C | -5.327909263 | -2.102708257 | 3.271649619 |
| H | -3.404354297 | -2.264808377 | 4.221649593 |
| C | -5.315413166 | -1.787093092 | 0.872058563 |
| H | -3.382881872 | -1.710544301 | -0.064859871 |
| C | -6.019497625 | -1.931461954 | 2.071056129 |
| H | -5.872952411 | -2.219530877 | 4.203484975 |
| H | -5.849909176 | -1.656412882 | -0.063943126 |
| H | -7.105345315 | -1.911749297 | 2.068000574 |
| H | 1.350375962 | 1.160557649 | 2.166893594 |
| H | 1.426306971 | -0.658731767 | 2.251229515 |
| H | 1.900718179 | 0.213103909 | 0.713828686 |
| F | -1.421454177 | 2.347583114 | -0.564972931 |
| F | 0.219145602 | 2.909553667 | 0.755387167 |
| F | 0.625410539 | 1.774090657 | -1.055357979 |

**OTf$^{-}$**

| | | | |
|---|---|---|---|
| C | -1.831602651 | 0.178769254 | -0.049990783 |
| S | -1.211598736 | 1.055453681 | -1.568464612 |
| O | 0.259694890 | 0.965346093 | -1.420136573 |
| O | -1.779192222 | 2.415567441 | -1.418708880 |
| O | -1.786116755 | 0.248357579 | -2.669922682 |
| F | -1.398924620 | -1.097030550 | -0.010423805 |
| F | -1.406764174 | 0.785661926 | 1.075869323 |
| F | -3.178420960 | 0.152098800 | -0.012957521 |

**Int2**

| | | | |
|---|---|---|---|
| Au | -0.718425517 | 1.264928278 | 0.292807480 |
| C | 0.920529643 | 1.221695893 | -0.977591397 |
| H | 0.460149341 | 1.268589909 | -1.965206756 |
| H | 1.521145154 | 2.100997358 | -0.749319409 |
| H | 1.455703661 | 0.289490830 | -0.805912428 |
| C | 0.581582450 | 0.990423417 | 1.815572179 |
| F | -0.156466693 | 1.011007951 | 2.944154604 |
| F | 1.506130776 | 1.958760568 | 1.910178519 |
| F | 1.218660485 | -0.190910600 | 1.762766486 |
| O | -2.415820319 | 1.591486543 | -1.173086322 |
| S | -3.556569572 | 1.526624917 | -0.166097984 |
| C | -4.291212969 | -0.159290572 | -0.462509316 |
| F | -3.343147466 | -1.089013827 | -0.304702982 |
| F | -5.274063833 | -0.377040391 | 0.409829345 |
| F | -4.770931809 | -0.223585520 | -1.703852616 |
| O | -2.919657407 | 1.425326299 | 1.197963061 |
| O | -4.631865690 | 2.493309024 | -0.343215347 |

**$PPh_3$**

| | | | |
|---|---|---|---|
| P | -0.892604118 | 0.571568686 | 0.003294314 |
| C | -0.041847248 | 1.391706240 | 1.431507920 |
| C | -0.752693644 | 1.453761469 | 2.642670371 |
| C | 1.232862385 | 1.975804002 | 1.370602815 |
| C | -0.196712393 | 2.063428076 | 3.767600140 |
| H | -1.749820535 | 1.024479190 | 2.703796419 |
| C | 1.784589274 | 2.596482993 | 2.493849250 |
| H | 1.797823605 | 1.949200780 | 0.444227277 |
| C | 1.073920109 | 2.639033966 | 3.694828410 |
| H | -0.759477490 | 2.098097054 | 4.696277425 |
| H | 2.771890667 | 3.045644519 | 2.428987730 |
| H | 1.504616541 | 3.122959776 | 4.566866335 |
| C | 0.090658483 | 1.148962269 | -1.458337895 |
| C | 1.199049959 | 0.471594580 | -1.989959747 |
| C | -0.338923912 | 2.331419781 | -2.085361430 |
| C | 1.864556042 | 0.969114147 | -3.112748851 |
| H | 1.544211652 | -0.448490868 | -1.529125216 |
| C | 0.332786143 | 2.834204860 | -3.200009211 |
| H | -1.207777225 | 2.859525155 | -1.699951718 |
| C | 1.435882179 | 2.151855065 | -3.718247774 |
| H | 2.719717621 | 0.431324672 | -3.513013543 |
| H | -0.011564312 | 3.751479465 | -3.669461669 |
| H | 1.954114952 | 2.536397726 | -4.592152218 |
| C | -0.382290980 | -1.202348475 | 0.174694520 |
| C | -1.130274428 | -2.150326383 | -0.545139748 |
| C | 0.664437960 | -1.654322155 | 0.993066768 |
| C | -0.827344318 | -3.509749599 | -0.465939560 |
| H | -1.957562177 | -1.821385281 | -1.169435232 |
| C | 0.960130586 | -3.016768719 | 1.081226851 |
| H | 1.250744846 | -0.942816668 | 1.565770497 |
| C | 0.218462973 | -3.946798713 | 0.350492444 |
| H | -1.413906331 | -4.227457541 | -1.032700419 |
| H | 1.772580940 | -3.350020589 | 1.721247710 |
| H | 0.449544258 | -5.005932097 | 0.421216406 |

**TS1A**

Imaginary frequency: 400.6*i* $cm^{-1}$

| | | | |
|---|---|---|---|
| Au | 1.067228578 | -0.334230494 | 0.209221967 |
| P | 0.082473001 | -2.193167033 | 1.388041465 |
| C | -1.340899625 | -1.599375157 | 2.369590489 |
| C | -2.660193986 | -1.902838598 | 2.011032395 |
| C | -1.092625498 | -0.711503561 | 3.429819788 |
| C | -3.721220485 | -1.327346242 | 2.713703696 |
| H | -2.860585118 | -2.567233408 | 1.177946831 |
| C | -2.155971334 | -0.143610346 | 4.130201530 |
| H | -0.070658036 | -0.465923529 | 3.706542145 |
| C | -3.472702090 | -0.449175737 | 3.770829921 |
| H | -4.742273385 | -1.562949612 | 2.428522594 |
| H | -1.957460935 | 0.538661905 | 4.951690217 |
| H | -4.301188981 | -0.001651773 | 4.312173034 |
| C | 1.260919273 | -2.912602940 | 2.590412832 |
| C | 0.844637688 | -3.464424615 | 3.812297314 |
| C | 2.621995485 | -2.940984843 | 2.246400767 |
| C | 1.780134602 | -4.039741341 | 4.673408088 |
| H | -0.202777826 | -3.437159657 | 4.095765485 |
| C | 3.552598938 | -3.520322433 | 3.109531178 |
| H | 2.953553178 | -2.509888105 | 1.304820617 |
| C | 3.132571005 | -4.069550171 | 4.323548556 |
| H | 1.451614772 | -4.462873105 | 5.618250140 |
| H | 4.603513336 | -3.536935016 | 2.836292977 |
| H | 3.857951890 | -4.515692704 | 4.997751852 |
| C | -0.503278517 | -3.585098909 | 0.360915672 |
| C | -0.606898803 | -3.425684845 | -1.027904946 |
| C | -0.861869509 | -4.808777831 | 0.950604453 |
| C | -1.076030891 | -4.476401135 | -1.818073308 |
| H | -0.343912150 | -2.482667936 | -1.487823868 |
| C | -1.327487908 | -5.854825232 | 0.155723455 |
| H | -0.774752863 | -4.946751321 | 2.023960274 |
| C | -1.436418622 | -5.689094009 | -1.228866785 |
| H | -1.165289721 | -4.338780638 | -2.891384382 |
| H | -1.604572360 | -6.798720755 | 0.616151541 |
| H | -1.802047011 | -6.505445062 | -1.845230002 |
| C | -1.020894183 | 1.338832035 | 0.632814153 |
| H | -0.892981279 | 1.896951977 | -0.282717572 |
| H | -2.009555863 | 1.034443857 | 0.936282721 |
| H | -0.278012107 | 1.458520599 | 1.411642892 |
| C | 2.053441194 | 1.275383778 | -0.665019065 |
| F | 3.271867851 | 1.492849311 | -0.065705248 |
| F | 1.416429015 | 2.488956567 | -0.611923743 |
| F | 2.340318066 | 1.073418312 | -1.987320147 |
| O | -1.657637630 | -0.491127114 | -0.580408327 |
| S | -2.371597479 | -0.115951236 | -1.865680099 |
| O | -3.003234699 | -1.276661714 | -2.508145492 |
| O | -3.139548260 | 1.133956766 | -1.752456716 |
| C | -0.952962065 | 0.327716583 | -2.988240864 |
| F | -0.365208220 | 1.458696931 | -2.563756846 |
| F | -1.389973073 | 0.516947400 | -4.237534943 |
| F | -0.028996441 | -0.649791111 | -3.002750999 |

**TS1B**

Imaginary frequency: 192.4*i* $cm^{-1}$

| | | | |
|---|---|---|---|
| Au | -0.566408573 | 0.265655604 | 0.808755205 |
| C | 0.148663364 | 2.094542968 | -0.391603005 |
| H | -0.795690359 | 2.266813213 | -0.883711374 |
| H | 0.422332901 | 2.713496603 | 0.452192197 |
| H | 0.943386587 | 1.590599991 | -0.923805277 |
| C | 1.413323067 | 0.114300601 | 1.393884148 |
| F | 1.902361476 | 1.196728316 | 2.053792869 |
| F | 2.287945589 | -0.149566078 | 0.389123873 |
| F | 1.507893540 | -0.936140277 | 2.266485864 |
| P | -2.969362285 | 0.214766190 | 0.310771679 |
| C | -3.569519201 | -1.509618239 | 0.450244961 |
| C | -4.402812699 | -2.095497159 | -0.512965365 |
| C | -3.153194244 | -2.265235535 | 1.560363295 |
| C | -4.818437305 | -3.420380668 | -0.361564654 |
| H | -4.725421635 | -1.525628598 | -1.378042248 |
| C | -3.574317399 | -3.585976825 | 1.707117619 |
| H | -2.505522281 | -1.820664118 | 2.312784545 |
| C | -4.406694431 | -4.165128324 | 0.745001137 |
| H | -5.463834314 | -3.868430812 | -1.111357722 |
| H | -3.249752571 | -4.162435415 | 2.568276020 |
| H | -4.730535651 | -5.195722898 | 0.857082408 |
| C | -3.415529298 | 0.822360898 | -1.355056799 |
| C | -4.620434943 | 1.499815347 | -1.600030480 |
| C | -2.513495845 | 0.606958023 | -2.410503506 |
| C | -4.918600001 | 1.945719021 | -2.888258500 |
| H | -5.320396269 | 1.685040916 | -0.791736013 |
| C | -2.815505815 | 1.060273607 | -3.694772984 |
| H | -1.571157151 | 0.098816132 | -2.226127875 |
| C | -4.018551761 | 1.728023258 | -3.934129591 |
| H | -5.851202908 | 2.471142387 | -3.071716484 |
| H | -2.102352200 | 0.908416437 | -4.498249440 |
| H | -4.249383148 | 2.087896634 | -4.932496815 |
| C | -3.905578637 | 1.204637785 | 1.533189715 |
| C | -5.175742790 | 0.817505876 | 1.988307740 |
| C | -3.326688672 | 2.389811315 | 2.017279676 |
| C | -5.857673370 | 1.612747842 | 2.910925094 |
| H | -5.629851875 | -0.100963694 | 1.630042995 |
| C | -4.015119317 | 3.181688735 | 2.936079809 |
| H | -2.339087295 | 2.691394430 | 1.678434810 |

| | | | |
|---|---|---|---|
| C | -5.280009965 | 2.793141345 | 3.384148670 |
| H | -6.839212022 | 1.306999589 | 3.261122717 |
| H | -3.560577936 | 4.095984220 | 3.305649299 |
| H | -5.812265041 | 3.406994860 | 4.104934185 |
| O | 0.658541671 | 3.918761431 | -1.620681595 |
| S | 1.721459391 | 3.747594997 | -2.676194443 |
| C | 0.812288464 | 2.826613881 | -4.010098980 |
| F | -0.287605514 | 3.489144521 | -4.397398798 |
| F | 1.596216038 | 2.645154081 | -5.081452792 |
| F | 0.428141767 | 1.609941350 | -3.565416426 |
| O | 2.110828376 | 5.014184065 | -3.316176751 |
| O | 2.799801470 | 2.816445142 | -2.294930808 |

**TS2**

Imaginary frequency: 389.5*i* $cm^{-1}$

| | | | |
|---|---|---|---|
| Au | 0.237581471 | -0.022570270 | -0.138604642 |
| C | -1.301328576 | 1.879381588 | -1.099630392 |
| H | -0.336823317 | 2.360695999 | -0.976228115 |
| H | -1.736646699 | 1.884425955 | -2.090250536 |
| H | -1.965651598 | 1.835844340 | -0.244023972 |
| C | 1.736966952 | 0.441943805 | 1.102605882 |
| F | 1.319637511 | 0.732446386 | 2.363904326 |
| F | 2.456527088 | 1.520320167 | 0.686621472 |
| F | 2.624832970 | -0.582332924 | 1.224305420 |
| O | -1.498270536 | -0.256185817 | -1.424736949 |
| S | -1.680854679 | -0.743290344 | -2.906886397 |
| O | -2.478132231 | -1.965414702 | -2.916989211 |
| O | -2.044509777 | 0.381843294 | -3.770807695 |
| C | 0.035323656 | -1.246883979 | -3.448076679 |
| F | 0.858764884 | -0.193980929 | -3.398386506 |
| F | -0.040080858 | -1.688684745 | -4.701567076 |
| F | 0.509470505 | -2.216227662 | -2.662323692 |

**1a**

| | | | |
|---|---|---|---|
| Au | -0.305298475 | 0.267841649 | 0.300240133 |
| P | -2.691717059 | 0.354583470 | 0.048236557 |
| C | -3.452186174 | 1.751986674 | 0.962181730 |
| C | -2.722047387 | 2.946879814 | 1.073482072 |
| C | -4.725919082 | 1.665952952 | 1.544768764 |
| C | -3.264366219 | 4.041892600 | 1.746486237 |
| H | -1.728786184 | 3.016562261 | 0.638045683 |
| C | -5.261908007 | 2.763249308 | 2.221639653 |
| H | -5.297156921 | 0.745503072 | 1.477396960 |
| C | -4.533989251 | 3.950903610 | 2.322289547 |
| H | -2.691661114 | 4.961105989 | 1.827874221 |
| H | -6.247142375 | 2.686973450 | 2.672647744 |
| H | -4.952419143 | 4.801360126 | 2.852971194 |
| C | -3.519872521 | -1.167806229 | 0.651398979 |
| C | -4.664641474 | -1.696473012 | 0.035921428 |
| C | -2.977293769 | -1.817341356 | 1.772566198 |
| C | -5.260359438 | -2.853799171 | 0.541431925 |
| H | -5.088761587 | -1.212865576 | -0.838305908 |
| C | -3.579148761 | -2.970502625 | 2.276469031 |
| H | -2.082306352 | -1.422815660 | 2.246188308 |
| C | -4.720381332 | -3.490657550 | 1.660831189 |
| H | -6.144375359 | -3.258402370 | 0.056916108 |
| H | -3.151260969 | -3.466074944 | 3.143028178 |
| H | -5.183909757 | -4.393094565 | 2.049064110 |
| C | -3.192455735 | 0.549448394 | -1.706878002 |
| C | -2.445926419 | -0.128043379 | -2.685687925 |
| C | -4.279546944 | 1.344937671 | -2.098136250 |
| C | -2.791802828 | -0.020668886 | -4.032588759 |
| H | -1.592365400 | -0.734135059 | -2.393555948 |
| C | -4.618160174 | 1.453414472 | -3.448713273 |
| H | -4.858887905 | 1.883154442 | -1.354783723 |
| C | -3.877421581 | 0.771242635 | -4.415898755 |
| H | -2.208190185 | -0.547623341 | -4.781828125 |
| H | -5.459616136 | 2.074208215 | -3.742887261 |
| H | -4.140980961 | 0.860762872 | -5.465908106 |
| C | 1.743600691 | 0.196591943 | 0.506298599 |
| F | 2.293967526 | 1.350979912 | 1.012363169 |
| F | 2.416106387 | -0.020145512 | -0.673499644 |
| F | 2.188320389 | -0.796902265 | 1.346651930 |

**MeOTf**

| | | | |
|---|---|---|---|
| C | -1.943275338 | 0.305841585 | -0.327859272 |
| S | -0.583983602 | 1.077013177 | -1.355925206 |
| O | 0.675668969 | 0.653184503 | -0.768375690 |
| O | -0.929088440 | 2.481650482 | -1.536378453 |
| O | -0.754302242 | 0.254565059 | -2.728900656 |
| C | -1.608842133 | 0.806403007 | -3.784892394 |
| H | -1.474315142 | 0.116476840 | -4.616955872 |
| H | -2.650373679 | 0.816560199 | -3.462053880 |
| H | -1.271076259 | 1.806334278 | -4.056034196 |
| F | -1.743456993 | -1.004888403 | -0.229020168 |
| F | -1.922623948 | 0.858470979 | 0.882766904 |
| F | -3.125223591 | 0.536998843 | -0.904741614 |

## 6. References


1 A. Portugués, I. López-García, J. Jiménez-Bernad, D. Bautista and J. Gil-Rubio, *Chem. Eur. J.*, 2019, **25**, 15535–15547.

2 G. R. Fulmer, A. J. M. Miller, N. H. Sherden, H. E. Gottlieb, A. Nudelman, B. M. Stoltz, J. E. Bercaw and K. I. Goldberg, *Organometallics*, 2010, **29**, 2176–2179.

3 A. Johnson and R. J. Puddephatt, *J. Chem. Soc., Dalton Trans.*, 1976, 1360–1363.

4 Y. Usui, J. Noma, M. Hirano and S. Komiya, *Inorg. Chim. Acta*, 2000, **309**, 151–154.

5 R. D. Sanner, J. H. Satcher and M. W. Droege, *Organometallics*, 1989, **8**, 1498–1506.

6 S. Martínez-Salvador, J. Forniés, A. Martín and B. Menjón, *Angew. Chem. Int. Ed.*, 2011, **50**, 6571–6574.

7 O. Crespo, C. Díaz, C. O'Dwyer, M. C. Gimeno, A. Laguna, I. Ospino and M. L. Valenzuela, *Inorg. Chem.*, 2014, **53**, 7260–7269.

8 A. Zhdanko, M. Ströbele and M. E. Maier, *Chem. Eur. J.*, 2012, **18**, 14732–14744.

9 Tolstikova, L. L.; Shainyan, B. A. *Russ. J. Org. Chem.*, 2006, **42**, 1068–1074.

10 Zefirov, N. S.; Koz'min, A. S.; Zhdankin, V. V.; Kirin, V. N.; Yur'eva, N. M.; Sorokin, V. D. *Chem. Scr.* 1983, **22**, 195–200.

11 Reichel, M.; Krumm, B.; Vishnevskiy, Y. V; Blomeyer, S.; Schwabedissen, J.; Stammler, H.-G.; Karaghiosoff, K.; Mitzel, N. W. *Angew. Chem. Int. Ed.* 2019, **58**, 18557–18561.

12 Weiß, R.; Wagner, K.-G.; Hertel, M. *Chem. Ber.* 1984, **117**, 1965–1972.

13 M. J. Frisch, G. W. Trucks, H. B. Schlegel, G. E. Scuseria, M. A. Robb, J. R. Cheeseman, G. Scalmani, V. Barone, G. A. Petersson, H. Nakatsuji, X. Li, M. Caricato, A. V. Marenich, J. Bloino, B. G. Janesko, R. Gomperts, B. Mennucci, H. P. Hratchian, J. V. Ortiz, A. F. Izmaylov, J. L. Sonnenberg, D. Williams-Young, F. Ding, F. Lipparini, F. Egidi, J. Goings, B. Peng, A. Petrone, T. Henderson, D. Ranasinghe, V. G. Zakrzewski, J. Gao, N. Rega, G. Zheng, W. Liang, M. Hada, M. Ehara, K. Toyota, R. Fukuda, J. Hasegawa, M. Ishida, T. Nakajima, Y. Honda, O. Kitao, H. Nakai, T. Vreven, K. Throssell, J. A. J. Montgomery, J. E. Peralta, F. Ogliaro, M. J. Bearpark, J. J. Heyd, E. N. Brothers, K. N. Kudin, V. N. Staroverov, T. A. Keith, R. Kobayashi, J. Normand, K. Raghavachari, A. P. Rendell, J. C. Burant, S. S. Iyengar, J. Tomasi, M. Cossi, J. M. Millam, M. Klene, C. Adamo, R. Cammi, J. W. Ochterski, R. L. Martin, K. Morokuma, O. Farkas, J. B. Foresman and D. J. Fox, *Gaussian 16 (Revision A.03)*, Gaussian Inc., Wallingford CT, 2016.

14 A. Becke, *J. Chem. Phys.*, 1993, **98**, 5648–5652.

15 C. T. Lee, W. T. Yang and R. G. Parr, *Phys. Rev. B*, 1988, **37**, 785–789.

16 P. C. Hariharan and J. A. Pople, *Theor. Chim. Acta*, 1973, **28**, 213–222.

17 M. M. Francl, W. J. Pietro, W. J. Hehre, J. S. Binkley, M. S. Gordon, D. J. Defrees and J. A. Pople, *J. Chem. Phys.*, 1982, **77**, 3654–3665.

18 P. J. Hay and W. R. Wadt, *J. Chem. Phys.*, 1985, **82**, 299–310.

19 A. V. Marenich, C. J. Cramer and D. G. Truhlar, *J. Phys. Chem. B*, 2009, **113**, 6378–6396.
